# By Animal, Water, or Wind: Can Dispersal Mode Predict Genetic Connectivity in Riverine Plant Species?

**DOI:** 10.3389/fpls.2021.626405

**Published:** 2021-02-12

**Authors:** Alison G. Nazareno, L. Lacey Knowles, Christopher W. Dick, Lúcia G. Lohmann

**Affiliations:** ^1^Departamentos de Botânica, Universidade de São Paulo, São Paulo, Brazil; ^2^Department of Genetics, Ecology and Evolution, Federal University of Minas Gerais, Belo Horizonte, Brazil; ^3^Department of Ecology and Evolutionary Biology, University of Michigan, Ann Arbor, MI, United States; ^4^Smithsonian Tropical Research Institute, Panama City, Panama

**Keywords:** Amazon basin, gene flow, ddRADseq, genetic structure, single-nucleotide polymorphisms (SNPs)

## Abstract

Seed dispersal is crucial to gene flow among plant populations. Although the effects of geographic distance and barriers to gene flow are well studied in many systems, it is unclear how seed dispersal mediates gene flow in conjunction with interacting effects of geographic distance and barriers. To test whether distinct seed dispersal modes (i.e., hydrochory, anemochory, and zoochory) have a consistent effect on the level of genetic connectivity (i.e., gene flow) among populations of riverine plant species, we used unlinked single-nucleotide polymorphisms (SNPs) for eight co-distributed plant species sampled across the Rio Branco, a putative biogeographic barrier in the Amazon basin. We found that animal-dispersed plant species exhibited higher levels of genetic diversity and lack of inbreeding as a result of the stronger genetic connectivity than plant species whose seeds are dispersed by water or wind. Interestingly, our results also indicated that the Rio Branco facilitates gene dispersal for all plant species analyzed, irrespective of their mode of dispersal. Even at a small spatial scale, our findings suggest that ecology rather than geography play a key role in shaping the evolutionary history of plants in the Amazon basin. These results may help improve conservation and management policies in Amazonian riparian forests, where degradation and deforestation rates are high.

## Introduction

The proportion of total genetic variability that resides among populations (i.e., population genetic differentiation expressed by *F*_*ST*_; Wright, 1943) provides a measure of the evolutionary processes acting within species (Holsinger and Weir, 2009). This elemental descriptive statistic from population and evolutionary genetic theory (Wright, 1943, 1951, 1965; Malécot, 1948; Nei, 1977; Weir and Cockerham, 1984; Holsinger and Weir, 2009) often seems to relate to species’ life-history traits (e.g., Duminil et al., 2007; Fouquet et al., 2015; Lowe et al., 2018; Medina et al., 2018). As a matter of fact, species-specific traits linked to the spread of genes across space have been used to explain why the magnitude of genetic divergence among populations varies among taxa (Duminil et al., 2007; Fouquet et al., 2015; Ballesteros-Mejia et al., 2016; Hodel et al., 2018; Medina et al., 2018). For instance, the mode of locomotion in vertebrates is predictive of the amount of genetic divergence among populations, with greater genetic differentiation in animal species that walk compared with those that swim or fly (Medina et al., 2018). Likewise, in sessile organisms such as plants, the mode of pollen and seed dispersal (i.e., biotic versus abiotic mediated dispersal) and life-history traits (i.e., growth form and mating system), are thought to predict genetic divergence (Loveless and Hamrick, 1984; Hamrick et al., 1992, 1993; Hamrick and Godt, 1996; Nybom, 2004; Duminil et al., 2007; Ballesteros-Mejia et al., 2016; Lowe et al., 2018).

By extension, life-history traits linked to dispersal ability can also have a direct effect on a species’ response to physical barriers (Fouquet et al., 2015; Papadopoulou and Knowles, 2016), affecting biogeographical and diversification processes (Wallace, 1854; Waters et al., 2020). For example, several studies have shown that large rivers in the Amazon basin are genetic barriers that contribute to allopatric speciation in a plethora of taxa including birds (e.g., Ribas et al., 2012; Naka and Brumfield, 2018), amphibians (e.g., Fouquet et al., 2015), and primates (e.g., Boubli et al., 2015). Despite the strong genetic structure in some plant species imposed by rivers, the river barriers do not appear to be associated with speciation (Zellmer et al., 2012; Nazareno et al., 2019a). Moreover, the effectiveness of rivers as a barrier to gene flow in plants depends on the width of the river separating populations (Moreira and Fernandes, 2013; Nazareno et al., 2017a, 2019a,b), with narrow rivers acting as a more permeable barrier to dispersal than wider ones (Zellmer et al., 2012; Moreira and Fernandes, 2013; Nazareno et al., 2017a, 2019a; but see Collevatti et al., 2009). However, because plants differ in their mechanisms of seed dispersal (Willson and Traveset, 2000), the permeability of a river barrier may differ across species bearing different seed dispersal strategies, especially between species bearing biotic and abiotic-mediated seed dispersal mechanisms. For example, seed dispersal mediated by animals should prevent population divergence, reduce inbreeding, and increase the levels of genetic diversity (e.g., Hamrick et al., 1993; Hamrick and Godt, 1996; Nybom and Bartish, 2000; Nybom, 2004; Vieira et al., 2010; Giombini et al., 2017; but see Thiel-Egenter et al., 2008). However, these general expectations are based on dispersal across a continuous landscape, and it is unclear how those expectations translate into dispersal propensities across river barriers. The relative permeability of a river barrier to gene flow as a function of species-specific traits is even less clear.

**FIGURE 1:**
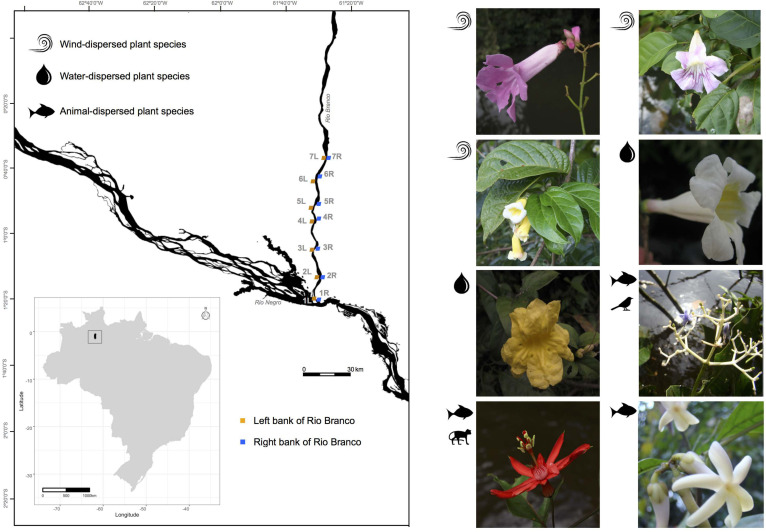
Sampling locations along the banks of the Rio Branco in the Amazon basin, Brazil. Locations labeled 1L-7L and 1R-7R denote the “Left” and “Right” river banks, respectively, of the following species (1) *Tanaecium pyramidatum*, (2) *Bignonia aequinoctialis*, (3) *Adenocalymma schomburgkii*, (4) *Pachyptera kerere*, (5) *Anemopaegma paraense*, (6) *Psychotria lupulina*, (7) *Passiflora spinosa*, and (8) *Amphirrhox longifolia*.

Here we present a riverscape genomics framework in which to assess whether distinct seed dispersal modes (i.e., hydrochory, anemochory, and zoochory) have a consistent effect on the level of historical and contemporary (i.e., past versus present) genetic connectivity among populations of riverine plant species (Figure 1). We also address whether trait-mediated dispersal co-varies among species with shared dispersal modes across a putative river barrier. Because the drainage system of the Amazon and its tributaries is dynamic (Latrubesse et al., 2005), rivers that presently act as dispersal barriers may not have done so in the past (or vice versa). In this context, we quantify the historical genetic differentiation associated with restricted gene flow, as well as the rates of contemporary dispersal. We also tested the Drift Paradox hypothesis (Ritland, 1989) to assess whether intraspecific genetic diversity increases downstream genetic diversity, a general trend observed in several taxa (Blanchet et al., 2020), including riverine plant species (e.g., Boedeltje et al., 2003; Prots et al., 2011).

An adequate understanding of gene flow patterns and genetic diversity in the Amazon can help guide effective conservation and management policies (Van Dyke and Lamb, 2020) of riverine plant species and the riparian forest areas where these species occur. Well thought-out and science-based management plans are urgent considering the rising rates of Amazonian riparian forest degradation and deforestation (Biggs et al., 2019; Nunes et al., 2019). The Brazilian Government plans to build 200 hydroelectric dams through the Amazon basin during the next decades; this represents an additional threat to the Amazon basin (Fearnside, 2015; Naka et al., 2020). To improve our understanding of patterns of genetic diversity and gene flow in this region, we quantified these genetic parameters in eight co-distributed plant species across the Rio Branco (Figure 1) using thousands of unlinked single-nucleotide polymorphisms (SNPs) obtained from a modified high-throughput DNA sequencing methodology (i.e., double-digest Restriction-site associated DNA sequencing – ddRADseq; Peterson et al., 2012).

The Rio Branco is a medium-sized river that has been used as a model system in various studies due to its size. This is due to the fact that narrow and wide rivers tend to affect gene flow in various taxa in the same way, regardless of their dispersal modes. The Rio Branco in particular has been shown to represent an important barrier to gene flow for birds (e.g., Naka et al., 2012) and primates (e.g., Boubli et al., 2015). Because seed dispersal mechanisms impact historical and contemporary gene flow (Duminil et al., 2007; Lowe et al., 2018), we expect a lack of congruence among plant species with distinct dispersal modes. Likewise, because dispersal mode is directly related to genetic connectivity among populations over time, we expect higher levels of genetic diversity and weaker genetic structure for biotically dispersed plant species than abiotically dispersed taxa. Although the dispersal ability (i.e., distinct animal foraging behavior and post-feeding movement) can vary in biotically dispersed species, three of the eight species studied here were placed in the same category because their seed dispersal was mediated by species with foraging behavior that can result in long seed dispersal distances (e.g., fishes; see Table 1). Indeed, previous results have indicated that long-distance dispersal modes, mediated by animals, are responsible for lower levels of genetic differentiation for plant species in forests (e.g., Howe and Smallwood, 1982; Loveless and Hamrick, 1984; Hamrick et al., 1992; Hamrick and Godt, 1996; Duminil et al., 2007), including those in riparian forest areas (e.g., Collevatti et al., 2009; Moreira and Fernandes, 2013). Since the locomotion of animals does not necessarily follow the flow of rivers, a lack of a downstream increase in intraspecific genetic diversity in animal dispersed-plant species is also expected. Comparative population genomics approaches allow us to empirically evaluate the links between dispersal mode and genetic connectivity for riverine plant species in the extremely biodiverse Amazon basin.

**TABLE 1:** Ecological traits of the eight Amazon plant species studied.

**Plant species (Family)**	**Growth form**	**Pollen dispersal vectors**	**Seed dispersal mode**	**References**
*Adenocalymma schomburgkii* (Bignoniaceae)	Liana	Bees^1^	Anemochory^2^	^1^Alcantara and Lohmann, 2010; ^2^Fonseca and Lohmann, 2019
*Amphirrhox longifolia* (Violaceae)	Small, shrub-like treelet (up to 3.0 m in height)^1^	Bees and butterflies^1^	Ballistic dispersal^1^/Zoochory by fishes^2^	^1^Braun et al., 2012; ^2^Correa et al., 2015
*Anemopaegma paraense* (Bignoniaceae)	Liana	Bees^1^	Hydrochory^1^	^1^Lohmann and Taylor, 2014
*Bignonia aequinoctialis* (Bignoniaceae)	Liana	Large to medium-sized bees^1^	Anemochory^2^	^1^Alcantara and Lohmann, 2010; ^2^Zuntini, 2014
*Pachyptera kerere* (Bignoniaceae)	Liana	Large to medium-sized bees^1^	Hydrochory^2^	^1^Alcantara and Lohmann, 2010; ^2^Francisco and Lohmann, 2018
*Passiflora spinosa* (Passifloraceae)	Liana	Humming birds^1^	Zoochory by fishes and primates^2^	^1^Ulmer and MacDougal, 2004; ^2^Nazareno et al., 2019a
*Psychotria lupulina* (Rubiaceae)	Small shrub (up to 1.5 m in height)^1^	Bees^1^	Zoochory by fishes and birds^2,3,4^	^2^Macedo and Prance, 1978; ^3^Valencia, 2002; ^1^Taylor, 2007; ^4^Correa et al., 2016
*Tanaecium pyramidatum* (Bignoniaceae)	Liana	Large to medium-sized bees and wasps^1^	Anemochory^2^	^1^Alcantara and Lohmann, 2010; ^2^Frazão and Lohmann, 2019

## Materials and Methods

### Species Studied

Eight Amazonian plant species were selected for this study: *Adenocalymma schomburgkii* (DC.) L.G. Lohmann (Bignoniaceae), *Amphirrhox longifolia* (A. St.-Hil.) Spreng. (Violaceae), *Anemopaegma paraense* Bureau & K. Schum (Bignoniaceae), *Bignonia aequinoctialis* L. (Bignoniaceae), *Pachyptera kerere* (Aubl.) Sandwith (Bignoniaceae), *Passiflora spinosa* (Poepp. & Endl.) Mast. (Passifloraceae), *Psychotria lupulina* Benth. (Rubiaceae), and *Tanaecium pyramidatum* (Rich.) L.G. Lohmann (Bignoniaceae). These species were chosen due to their varied dispersal modes (Table 1) and high abundance on both banks of the Rio Branco, an ecologically important river barrier for the Amazonian biota (Naka et al., 2012; Boubli et al., 2015; Nazareno et al., 2019a). Assignment to the categories of primary seed dispersal modes, which encompasses all species studied here, was based either on specific morphological features that suggest particular modes of dispersal or on published field observations (Table 1). Although life-history traits such as growth form (Hamrick and Godt, 1996) and pollination mode (Ballesteros-Mejia et al., 2016) may also impact genetic structure, we had insufficient variation in these traits across species, preventing us from including pollination as a variable in our comparative analyses (Table 1). While mating system might also affect the levels of intra- and inter-population genetic diversity, no information about the mating system of the eight focal taxa is available to date.

We tested the effect of riverscape features (i.e., geographic distance along and between river banks) on population structure, while considering distinct dispersal modes. Toward this end, we collected new genomic data for five plant species (i.e., *A. schomburgkii*, *A. paraense*, *B. aequinoctialis*, *P. kerere*, and *T. pyramidatum*) and combined this information with data available from the same location for *A. longifolia*, *P. spinosa*, and *P. lupulina* (Nazareno et al., 2019a). This strategy enabled us to consider multiple dispersal modes in our analyses, allowing us to address the unexplored question of the effects of dispersal mode on the historical and contemporary genetic structure of riverine plants.

### Study Area and Sampling Design

At 750 km long, the Rio Branco (whose drainage basin spans 235,073 km^2^) is the largest tributary of the Rio Negro; it is located on the Guiana Shield, within a sediment-rich “white-water” river ecoregion, and flows southward into the Negro-Amazon Rivers (Figure 1). The Rio Branco is one of several rivers in the Amazon basin threatened by the Brazilian Government’s plan to build hydroelectric dams and associated hydro-ways along its course (Naka et al., 2020). Our sampling of plant populations was restricted to the lower Rio Branco, where vegetation structure is directly related to the hydro-edaphic features, supporting a different type of vegetation and plant community structure than that found along the shores of the clear and nutrient poor black-water rivers (Worbes, 1997).

We sampled seven locations on each river bank of the lower Rio Branco, spanning a distance of 90 km (Figure 1). For each sampling location, we identified a corresponding sampling location on the opposite river bank with distances varying with the width of the river from 1.0 km (7R – 7L, Figure 1) to 3.8 km (3R – 3L, Figure 1). On the same side of the river, distances between pairs of sampling locations varied from 16.2 (1R – 2R, Figure 1) to 89.2 km (1R – 7R, Figure 1) for the right bank, and 8.3 (3L – 4L, Figure 1) to 86.8 km (1L – 7L, Figure 1) for the left bank. To avoid sampling close relatives, reproductive individuals were sampled at intervals of at least 50 m. In total, we collected 622 flowering individuals of *A. schomburgkii* (*n* = 64), *A. longifolia* (*n* = 78), *A. paraense* (*n* = 84), *B. aequinoctialis* (*n* = 78), *P. kerere* (*n* = 84), *P. spinosa* (*n* = 78), *P. lupulina* (*n* = 78), and *T. pyramidatum* (*n* = 78). Those individuals were distributed among 14 sampling locations situated on both banks (left and right) of the Rio Branco during the wet seasons of 2015 and 2016 (Supplementary Table 1). Although we sampled 14 locations for five plant species, one location was left out in the final analyses due to the very small number of individuals collected there (Supplementary Table 1). Vouchers for all species (Supplementary Table 1) were deposited at the Herbarium of the University of São Paulo (SPF), São Paulo, Brazil.

For each sampling location, sample sizes ranged from two to six individuals (Supplementary Table 1). The common limitations of reduced sample sizes are offset by large sets of SNPs (Willing et al., 2012; Nazareno et al., 2017b), allowing for high-resolution identification of genetic structure (Trucchi et al., 2016; Kotsakiozi et al., 2017; Nazareno et al., 2017b), even when just two individuals are sampled per population (Nazareno et al., 2017b). The assumption that small sample sizes are adequate to estimate population genetics parameters has previously been validated for *A. longifolia* (Nazareno et al., 2017b), *P. spinosa*, and *P. lupulina* (Nazareno et al., 2019a). To ensure that this assumption also applied to *A. schomburgkii*, *A. paraense*, *B. aequinoctialis*, *P. kerere*, and *T. pyramidatum*, we performed genetic structure analyses (e.g., pairwise genetic differentiation) while reducing the number of samples randomly, from six to two using a custom script in R (described by Nazareno et al., 2017b). When we compared the mean of the pairwise genetic differentiation between different sample sizes, the results confirmed the robustness of the results to different sample sizes. More specifically, for *A. schomburgkii* an *F*_*ST*_ (*n* = 6) = 0.014, 95% CI (0.010, 0.018) compared with an *F*_*ST*_ (*n* = 2) = 0.013, 95% CI (0.009, 0.017), for *A. paraense* an *F*_*ST*_ (*n* = 6) = 0.015, 95% CI (0.008, 0.022) compared with an *F*_*ST*_ (*n* = 2) = 0.022, 95% CI (0.014, 0.300), for *B. aequinoctialis* an *F*_*ST*_ (*n* = 6) = 0.052, 95% CI (0.029, 0.075) compared with an *F*_*ST*_ (*n* = 2) = 0.051, 95% CI (0.034, 0.067), for *P. kerere* an *F*_*ST*_ (*n* = 6) = 0.022, 95% CI (0.012, 0.032) compared with an *F*_*ST*_ (*n* = 2) = 0.010, 95% CI (0.003, 0.017), and for *T. pyramidatum* an *F*_*ST*_ (*n* = 6) = 0.032, 95% CI (0.029, 0.035) compared with an *F*_*ST*_ (*n* = 2) = 0.033, 95% CI (0.026, 0.040).

### Library Preparation and Sequencing

A total of five new genomic libraries were prepared for *A. schomburgkii*, *A. paraense*, *B. aequinoctialis*, *P. kerere*, and *T. pyramidatum*. The genomic libraries were created using a double digest RADseq (ddRAD) protocol (Peterson et al., 2012), following modifications proposed by Nazareno et al. (2017a) to minimize variance in the number of reads per individual within each pool. Briefly, genomic DNA was extracted using the Macherey-Nagel kit (Macherey-Nagel GmbH & Co. KG). The restriction enzymes *EcoRI* and *MseI* were used to digest genomic DNA, and the DNA fragments obtained were ligated to adaptors with unique barcodes. After that, PCRs were performed on each individual sample and amplicons were pooled for automatic size selection using Pippin Prep (Sage Science, Beverly, MA, United States). The genomic libraries were sequenced on Illumina HiSeq 2500 flow cell (Illumina Inc., San Diego, CA, United States) to generate single-end 100 bp reads at The Centre for Applied Genomics, Toronto, Canada. More details of the protocol can be found in Supplementary Appendix 1 (Supporting information). Raw sequence data from previously published work on *A. longifolia*, *P. spinosa*, and *P. lupulina* (Nazareno et al., 2019a) were analyzed with the same protocols used on the five new genomic libraries generated in this study.

### Identifying and Genotyping SNPs

Files containing the raw sequence reads were analyzed separately for each plant species in Stacks 1.35 (Catchen et al., 2011, 2013; Rochette et al., 2019) using *de novo* assembly. Initially, we used the process_radtags program in Stacks to assign reads to individuals and eliminate poor quality reads (i.e., Phred quality score < 33) and reads missing the expected *EcoRI* cut site (options –barcode_dist 2 -q -e ecoRI). All sequences were processed in ustacks to produce consensus sequences of RAD tags. The program ustacks takes a set of short-read sequences from a single sample as input and aligns them into exactly matching stacks. For each species, a maximum-likelihood framework (Hohenlohe et al., 2010) was applied to estimate the diploid genotype for each individual at each nucleotide position. The optimum minimum depth of coverage (*m*) to create a stack was set at three sequences, the maximum distance allowed between stacks was two nucleotides, and the maximum number of stacks allowed per *de novo* locus was three. The stacks assembly enabled the Deleveraging algorithm (-d), which resolves overmerged tags, and the Removal algorithm (-r), which drops highly repetitive stacks and nearby errors from the algorithm. The alpha value for the SNP model was set at 0.05. As reported by Catchen et al. (2013), low alpha values (i.e., <0.10) avoid underestimating true heterozygous genotypes. Cstacks was used to build a catalog of consensus loci containing all the loci from all the individuals and merging all alleles together. After processing the consensus loci in cstacks, stacks generated from all samples of each plant species were searched against the catalog. The SNPs were called using the software sstacks, tsv2bam, and gstacks (Rochette et al., 2019), with default settings. We ran the software POPULATIONS (Catchen et al., 2011, 2013; Rochette et al., 2019) to obtain the loci that were present in at least 85% of individuals, with ddRAD tags present in all sampling locations. In addition, we used a Minor Allele Frequency (MAF) of 1% (–min_maf 0.01) to filter out allelic types – with a count of one – that may mask population genetic structure (e.g., Rodríguez-Ezpeleta et al., 2016). Only one SNP per locus was included in the final dataset.

### Quality Control of Genomic Data

For each plant species, the numbers of raw sequence reads and unlinked SNPs were characterized for all populations. We used BayeScan 2.1 (Foll and Gaggiotti, 2008) to remove the SNPs potentially under balancing and divergent selection; this software was run with 20 pilot runs of 10,000 iterations, a burn-in of 50,000 iterations, and a final run of 100,000 iterations. In order to minimize false-positives, prior odds of the neutral model were set to 10,000 (i.e., the neutral model is 10,000 times more likely than the model with selection; Foll and Gaggiotti, 2008). Furthermore, deviation from Hardy–Weinberg (H-W) equilibrium was assessed using the exact test based on Monte Carlo permutations of alleles – the most appropriate test when small sample sizes are used (Barnholtz-Sloan, 2003). The H-W equilibrium tests were done using the adegenet package (Jombart, 2008; Jombart and Ahmed, 2011) implemented in R. We tested for linkage disequilibrium (LD) between loci using Arlequin 3.5.2 (Excoffier and Lischer, 2010). Type I error rates for tests of departure from H-W expectations and linkage disequilibrium were corrected for multiple *k* tests using the sequential Bonferroni procedure (Rice, 1989). After the adjustment of the *p*-value, SNPs that failed the H-W equilibrium test and the SNP pairs in LD in at least 50% of the sampling locations were excluded from further analyses.

We used the final dataset to calculate minor allele frequencies for each plant species using the package adegenet (Jombart, 2008; Jombart and Ahmed, 2011) in R 3.3.1 (R Core Team, 2018). We further estimated unbiased expected genetic diversity (*uH*_*E*_; Nei and Roychoudhury, 1974), observed heterozygosity (*H*_*O*_) and the inbreeding coefficient (Wright’s Fixation Index *F*) for each population. Population genetic statistics were averaged across loci using the R package diveRsity (Keenan et al., 2013). For *uH*_*E*_, *H*_*O*_, and *F*, the 95% confidence intervals were obtained to help evaluate differences between means estimated for all plant species. To test the Drift Paradox hypothesis (i.e., if there is a downstream increase of genetic diversity), we performed Pearson product-moment correlation test of *uH*_*E*_ and *H*_*O*_ with the geographic distance along the river course using the package ggpubr implemented in R 3.3.1 (R Core Team, 2018).

### Population Structure Analyses

In order to investigate how the river affects the population structure of plant species with different seed dispersal modes (e.g., anemochory, hydrochory, and zoochory), we assessed the historical and contemporary genetic structure and connectivity of *A. schomburgkii*, *A. paraense*, *B. aequinoctialis*, *P. kerere*, and *T. pyramidatum*. To allow for comparison with the animal-dispersed plant species (*A. longifolia*, *P. spinosa*, and *P. lupulina*), we analyzed the five abiotically dispersed plant species for which new data was obtained using the methods described in Nazareno et al. (2019a), with minor adjustments.

We assessed the genetic structure and the connectivity patterns between sampling locations along and across the river using complementary genetic analyses. For all plant species, we investigated the historical relationships among sampling locations using RAxML 8.2 (GTRGAMMA model; Stamatakis, 2014). The trees based in pairwise nucleotide sequence distances were visualized in FigTree 1.4.4^1^.

We calculated genetic distances among sampling locations (DA: Nei et al., 1983) and visualized the results by applying multidimensional scaling (MDS) in XL-STAT (Addinsoft), using the SMACOF method (Scaling by MAjorizing a COnvex Function). This method minimizes the “Normalized Stress” (De Leeuw, 1977), a measure that determines how well a particular configuration reproduces the observed distance matrix. As an ordination technique, MDS plot locations with similar genetic structure are grouped within the ordination space according to a given stress factor. No assumptions associated with the cause of structure such as HWE and gametic equilibrium are required.

To more precisely characterize the geographic distribution of genetic variation, we applied a Bayesian model that uses a Markov Chain Monte Carlo (MCMC), as implemented in the R package GENELAND 4.0.6 (Guillot, 2012). Bayesian clustering analyses based on Hardy–Weinberg and linkage equilibrium, as implemented in GENELAND, have been broadly used for this purpose. Although other statistical approaches can be employed to identify genetic groups of populations or individuals, we chose to use GENELAND because spatial information increases the power of correctly detecting the underlying population structure (e.g., Bonin et al., 2007). The statistical model implemented in GENELAND can also help to identify genetic discontinuities such as physical barriers among populations. We applied the spatial model with correlated allele frequencies proposed by Guillot et al. (2008), which enables inferences of differentiation even when there is limited gene flow caused by physical barriers. We conducted ten independent runs of 1.0 × 10^6^ in length, discarding the first 5.0 × 10^5^ iterations (burn-in) in post-processing. As the most likely number of *k* populations was unknown, it was treated as a simulated variable along with the MCMC simulations (1 ≤ *k* ≤ *N*), where *N* represents the number of sampling locations for each plant species, which ranged from 13 (*B. aequinoctialis* and *T. pyramidatum*) to 14 (*A. schomburgkii*, *A. paraense*, and *P. kerere*). The modal number of genetic groups of the best run (based on posterior density values) was considered as the number of genetic clusters (K).

To investigate whether species with different dispersal modes displayed congruent patterns of genetic structure, we estimated pairwise genetic differentiation (*F*_*ST*_; Weir and Cockerham, 1984) using the package hierfstat (Goudet, 2005) implemented in R 3.3.1 (R Core Team, 2018). We applied the non-parametric Wilcoxon–Mann–Whitney test to compare the estimates of pairwise genetic differentiation among plant species that are dispersed by animal, wind, or water. Wilcoxon–Mann–Whitney tests were performed using R (R Core Team, 2018), and considering three datasets: (i) all pairs of sampling locations, (ii) pairs of sampling locations across banks, and (iii) pairs of sampling locations along banks of the Rio Branco.

In order to investigate the similarity between genetic and geographic distance, we conducted a test for isolation by distance (IBD) (Mantel, 1967) between a matrix of pairwise genetic distances [*F*_*ST*_/(1 – *F*_*ST*_); Rousset, 1997] and a matrix of Euclidian distances using the package vegan implemented in R (R Core Team, 2018). For this test, we employed a 1.0 × 10^4^ permutation test of significance for the coefficient of correlation. A Mantel test was also applied to pairs of sampling locations situated (1) across river banks and (2) along each bank of the Rio Branco.

Finally, to examine the effect of the Rio Branco on the partitioning of genetic variation among sampling locations, we performed a Molecular Analysis of Variance (AMOVA; Excoffier et al., 1992). We defined two hierarchical levels at which we characterized population differentiation: between sampling locations from opposite river banks, and between sampling locations along the right and left banks. We used Arlequin 3.5.2 (Excoffier and Lischer, 2010) to calculate population differentiation estimates and their statistical significance based on 2.0 × 10^4^ random permutations.

### Contemporary Gene Flow

We obtained estimates of contemporary bidirectional migration rates (*m*) using a Bayesian inference framework implemented in BayesAss3-SNPs 1.1 (Wilson and Rannala, 2003; Mussmann et al., 2019). This program employs a Bayesian approach with MCMC sampling to estimate recent migration rates over the last five to seven generations. This method does not assume that populations are in migration-drift equilibrium or Hardy–Weinberg equilibrium. We conducted the Bayesian analysis with a sample size (*n*) of six individuals per sampling location (except for *A. schomburgkii*, *n* = 4.0 ± 1.3 SD; Supplementary Table 1) using 2.0 × 10^7^ interactions with a burn-in of 1.0 × 10^7^ generations and a sampling frequency of 5.0 × 10^3^. All migration rates whose 95% confidence intervals did not include zero were reported as significant. Estimates of migration rates are accurately obtained even when reduced sample sizes are employed (Nazareno et al., 2019a). To ensure that estimates of migration rates can be accurately obtained when small sample sizes are employed, we performed a preliminary analysis with a subset of *A. schomburgkii* individuals and compared migration rates obtained from sample sizes (n) of six, four, and two individuals in two populations. The results indicated that there are no differences in the migration rates (m) even when a small number of individuals (i.e., two individuals per sampling location) are used in the analysis [e.g., migration rates for *A. schomburgkii*, from population 1R to population 2R: m(*n* = 2) = 0.048, 95% CI (−0.044, 0.139), m(*n* = 4) = 0.059, 95% CI (−0.033, 0.151), and m(*n* = 6) = 0.080, 95% CI (−0.012, 0.172)].

To verify if contemporary migration rates are congruent among plant species displaying different dispersal modes, we applied the non-parametric Wilcoxon–Mann–Whitney test considering either all pairs of sampling locations, or pairs of sampling locations across banks, or pairs of sampling locations along banks of the Rio Branco. To investigate whether migration rates decrease with the increase of geographic distance between sampling locations, we performed Pearson product-moment correlation tests using the package ggpubr implemented in R 3.3.1 (R Core Team, 2018) using the following datasets: (i) all sampling locations, (ii) sampling locations located across banks, (iii) sampling locations located along each river bank, and (iv) sampling locations located along both river banks. To investigate if there is a directional pattern of contemporary gene flow along the river course (i.e., from downstream to upstream, or contrariwise), we also performed Pearson product-moment correlation tests, but considered the following datasets: (i) all sampling locations, (ii) sampling locations along both banks from downstream to upstream and contrariwise, (iii) sampling locations along each river bank from downstream to upstream and contrariwise, (iv) sampling locations across banks from downstream to upstream and contrariwise, and (v) sampling locations along both banks plus across banks from downstream to upstream and contrariwise.

## Results

The numbers of single-end raw reads produced for each lane of HiSeq 2500 Illumina and the mean number of retained reads that passed the default quality filters and which contained an identifiable barcode, are presented in Supplementary Appendix 2 (Supporting information). After additional quality filtering (see Supplementary Appendix 2), the total numbers of unlinked SNPs used in the genomic analyses were 36,768 for *A. schomburgkii*, 39,747 for *A. paraense*, 10,595 for *B. aequinoctialis*, 34,265 for *P. kerere*, and 28,121 for *T. pyramidatum*.

### Genetic Diversity for Biotically and Abiotically Dispersed Plant Species

**FIGURE 2:**
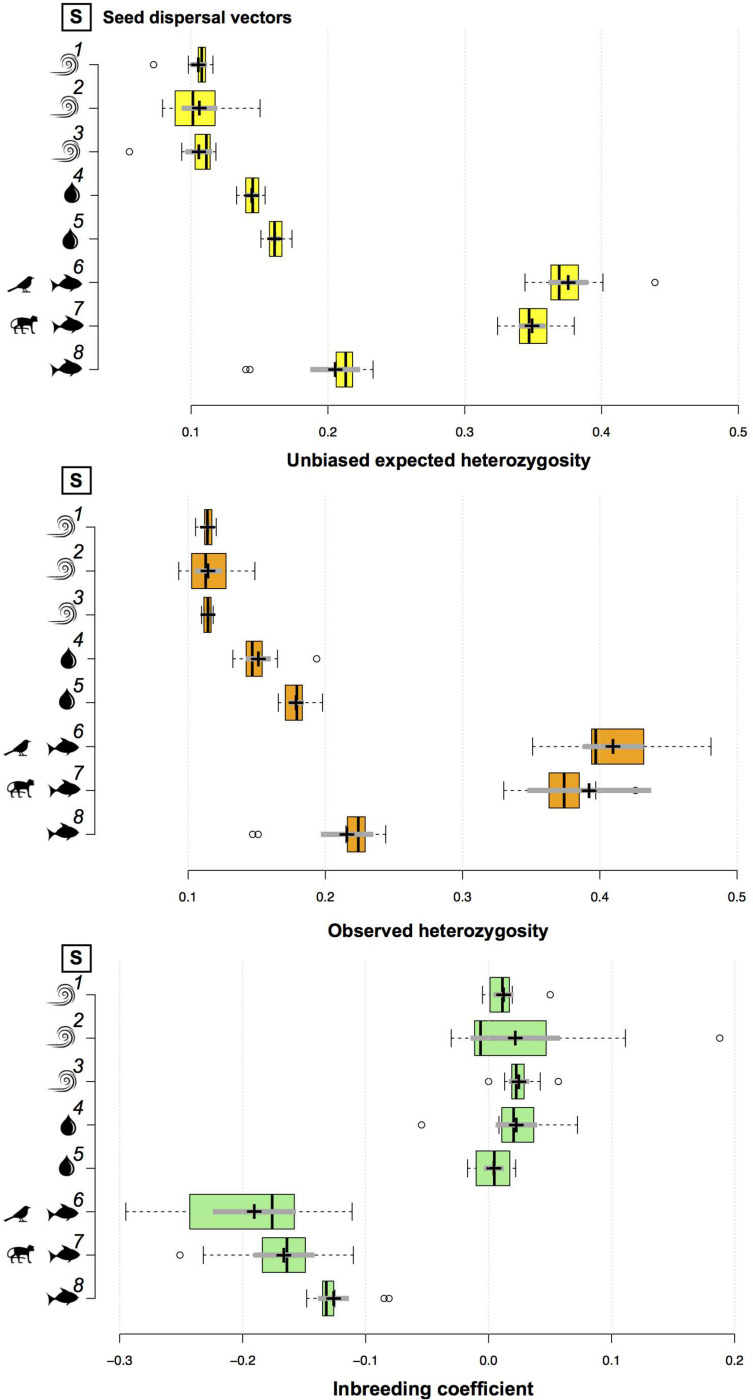
Boxplots comparing the genetic diversity (i.e., unbiased expected genetic diversity and observed heterozygosity) and inbreeding coefficient across species. Center lines show the medians; box limits indicate the 25th and 75th percentiles as determined by R software; whiskers extend 1.5 times the interquartile range from the 25th and 75th percentiles, with outliers represented by dots; crosses represent sample means with bars making the 95% confidence intervals of the means. Species 1–8 are ordered top to bottom as follows: (1) *T. pyramidatum*, (2) *B. aequinoctialis*, (3) *A. schomburgkii*, (4) *P. kerere*, (5) *A. paraense*, (6) *P. lupulina*, (7) *P. spinosa*, and (8) *A. longifolia*.

At the population level, genetic diversity estimates (i.e., *uH*_*E*_ and *H*_*O*_) did not vary much among populations of *A. schomburgkii*, *A. longifolia*, *A. paraense*, *B. aequinoctialis*, *P. kerere*, *P. spinosa*, *P. lupulina*, and *T. pyramidatum* (Supplementary Table 2). At the species level, the mean unbiased expected genetic diversity ranged from 0.104 (*B. aequinoctialis*) to 0.376 (*P. lupulina*), while the mean observed heterozygosity ranged from 0.113 (*B. aequinoctialis*) to 0.410 (*P. lupulina*; Supplementary Table 2). The genetic diversity estimates for animal-dispersed plant species were significantly higher than those estimated for water- or wind-dispersed plant species (Supplementary Table 2 and Figure 2). For wind-dispersed plant species, no significant differences among the genetic diversity estimates were observed, whereas among the water-dispersed plant species, those estimates were significantly higher for *A. paraense* than for *P. kerere*; among the animal-dispersed plant species, the estimates were significantly lower for *A. longifolia* than for *P. spinosa* and *P. lupulina* (Figure 2).

Very low levels of inbreeding were observed for wind- or water-dispersed plant species (Supplementary Table 2). For these species, the mean fixation index (i.e., inbreeding coefficient) ranged from 0.004 ± 0.007 95% CI (*A. paraense*) to 0.025 ± 0.007 95% CI (*A. schomburgkii*). Due to the excess of observed heterozygotes, the mean fixation index was negative for all animal-dispersed plant species (Supplementary Table 2 and Figure 2).

Genetic diversity estimates were not correlated with the distance along the river (Supplementary Table 3), except in *P. kerere*, where values of *uH*_*E*_ and *H*_*O*_ were higher in downstream than upstream sampling locations (*r* = 0.53, *p* = 0.05 for *uH*_*E*_; *r* = 0.55, *p* = 0.04 for *H*_*O*_; Supplementary Figure 2). These data do not find a directional pattern of genetic diversity along the Rio Branco for wind- or animal-dispersed plant species.

### Population Genetic Structure for Plant Species With Distinct Dispersal Mode

Samples did not cluster by river bank or by sampling locations for any of the eight species studied in the RAxML trees (Supplementary Figure 3). In general, geographically close sampling locations are not more closely related (Supplementary Figure 3). We also did not identify any potential barrier to gene flow by the Rio Branco for the abiotically dispersed species (Supplementary Figure 4), when the MDS and Bayesian clustering methods were employed. Using Kruskal’s stress values (i.e., a measure of how well a particular configuration reproduces the observed distance matrix), we inferred that two dimensions were sufficient to explain the genetic patterns estimated by MDS in each species. The sampling locations from each bank of the Rio Branco do not group together in the genetic pattern that emerged from the MDS plots (Supplementary Figure 4). These results closely match those obtained using Bayesian clustering analyses. For example, GENELAND clearly delineated a single group with minimal variance in the posterior probabilities over multiple runs in all water- or wind-dispersed plant species (Supplementary Figure 4). These results agree with those observed for the three animal-dispersed species (Supplementary Figure 4), despite the differences in dispersal mode among taxa (Table 1).

In general, pairwise genetic differentiation among populations was low (Supplementary Table 4). The average pairwise estimate of *F*_*ST*_ was 0.016 (95% CI 0.009, 0.023) for *A. schomburgkii*, 0.015 (95% CI 0.008, 0.022) for *A. paraense*, 0.052 (95% CI 0.029, 0.075) for *B. aequinoctialis*, 0.025 (95% CI 0.015, 0.034) for *P. kerere*, and 0.033 (95% CI 0.026, 0.039) *T. pyramidatum*. For the three animal-dispersed plant species the average pairwise estimate of *F*_*ST*_ was 0.021 (95% CI 0.019, 0.023) for *A. longifolia*, 0.031 (95% CI 0.026, 0.036) for *P. spinosa*, and 0.031 (95% CI 0.025, 0.037) for *P. lupulina* (see Nazareno et al., 2019a). Wilcoxon–Mann–Whitney tests showed incongruent patterns of genetic differentiation among animal-, water-, and wind-dispersed species (Table 2); animal-dispersed species showed an average of pairwise differentiation that was 1.2 to 1.4 times lower than the water- or wind-dispersed species, indicating that animal-dispersed species along the Rio Branco were more connected historically. These results are consistent when different datasets are considered in the analyses (i.e., pairs of sampling locations across river banks, and pairs of sampling locations along the banks of Rio Branco) (Table 2).

**TABLE 2:** Wilcoxon–Mann–Whitney tests for congruence of historical gene flow (i.e., pairwise genetic differentiation; lower diagonal) and contemporary migration rates (above diagonal) among animal- (*Amphirrhox longifolia, Passiflora spinosa*, and *Psychotria lupulina*), water- (*Pachyptera kerere* and *Anemopaegma paraense*), and wind-dispersed plant species (*Adenocalymma schomburgkii, Bignonia aequinoctialis*, and *Tanaecium pyramidatum*) considering all pairs of sampling locations, pairs of sampling locations across and along banks of Rio Branco (Amazon basin, Brazil).

Dispersal mode	All pairs of sampling locations			Pairs of sampling locations across banks			Pairs of sampling locations along banks		
	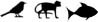	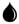	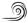	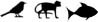	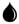	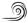	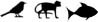	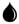	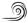
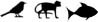	−	**13880**	**19497**	−	**3328**	**4750**	−	**3605**	**4999**
Animal		*p* = 0.040	*p* = 0.003		*p* = 0.032	*p* = 0.018		*p* = 0.004	*p* = 0.001
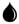	**3074**	−	16052	**637**	−	3656	**865**	−	3932
Water	*p* = 0.017		*p* = 0.605	*p* = 0.006		*p* = 0.499	*p* = 0.032		*p* = 0.609
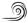	**1932**	**1439**	−	**272**	**1667**	−	**706**	**1508**	−
Wind	*p* < 0.001	*p* < 0.001		*p* < 0.001	*p* < 0.001		*p* < 0.001	*p* < 0.001	

*Lack of congruence is indicated by boldface values (p < 0.05).*

No significant IBD pattern was found in any of the five abiotically-dispersed plant species (*r* = −0.032, *p* = 0.623 in *A. schomburgkii*; *r* = −0.002, *p* = 0.496 in *A. paraense*; *r* = −0.092, *p* = 0.802 in *B. aequinoctialis; r* = 0.102, *p* = 0.148 in *P. kerere*, and *r* = 0.179, *p* = 0.060 in *T. pyramidatum*). Results were also not significant when applied between pairs of sampling locations across river banks (*r* = 0.075, *p* = 0.349 in *A. schomburgkii*; *r* = −0.061, *p* = 0.649 in *A. paraense*; *r* = −0.006, *p* = 0.505 in *B. aequinoctialis; r* = 0.062, *p* = 0.393 in *P. kerere*, and *r* = −0.034, *p* = 0.568 in *T. pyramidatum*). Likewise, results of simple matrix correlation between genetic and geographic distance were insignificant when applied separately along each bank of the Rio Branco (Supplementary Table 5).

**FIGURE 3:**
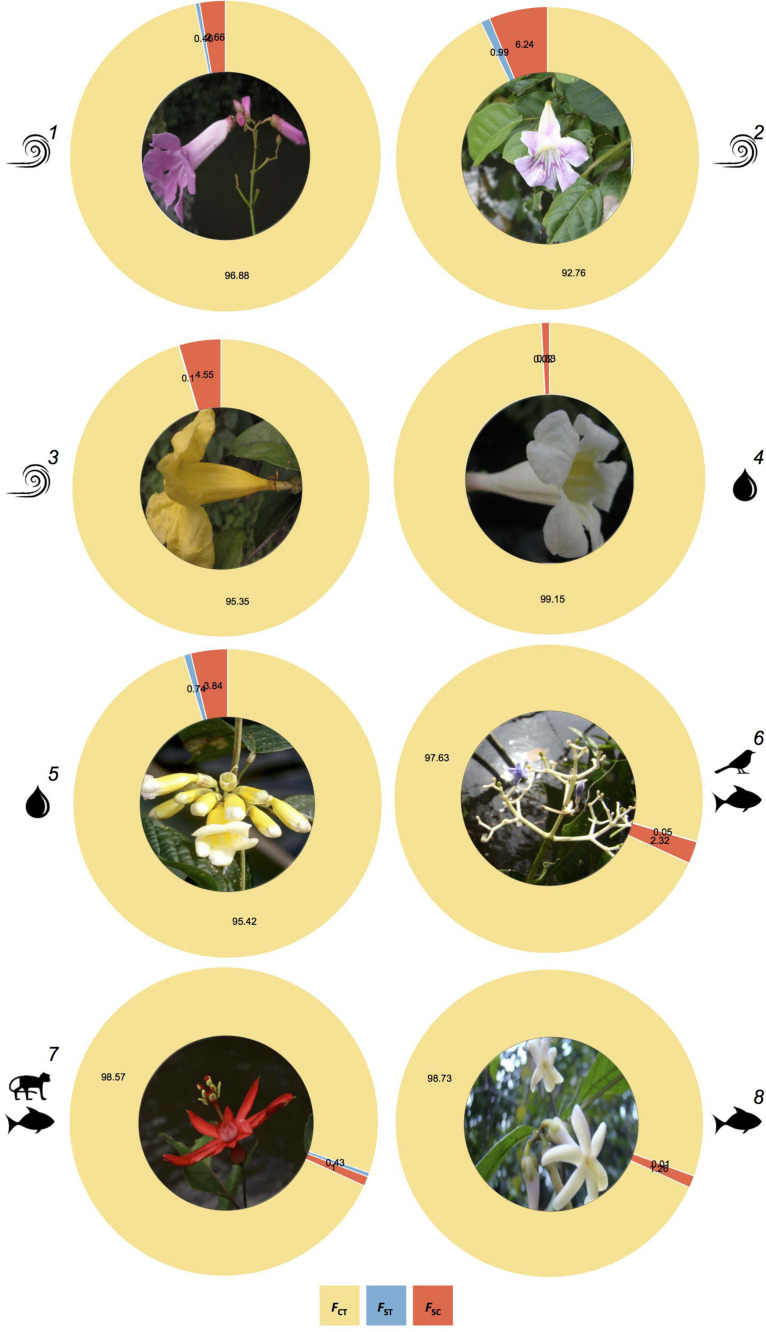
Comparison of the partitioning of genetic structure across plant species dispersed by wind (1-*T. pyramidatum*, 2-*B. aequinoctialis*, and 3-*A. schomburgkii*), water (4-*P. kerere* and 5-*A. paraense*) and animals (6-*P. lupulina*, 7-*P. spinosa*, and 8-*A. longifolia*), as revealed by the Analysis of Molecular Variance (AMOVA), where *F_*ST*_, F*_*SC*_, and *F*_*CT*_ represent the genetic structure between river banks, among sampling locations within banks, and within sampling locations, respectively.

For the abiotically and biotically dispersed species, the hierarchical multi-locus AMOVA revealed that most of the genetic differentiation was attributable to differences observed among sampling locations along each river bank rather than among sampling locations between river banks (Figure 3 and Supplementary Table 6). Taken together, these results indicate that the Rio Branco, a medium-sized river in the Amazon basin, is not a barrier to gene flow for the studied species.

### Contemporary Gene Flow for Plant Species With Distinct Dispersal Mode

When species displaying the same dispersal mode were considered, the average of contemporary gene flow was 1.4 to 1.5 times greater for animal-dispersed plant species than water- or wind-dispersed plant species. In addition, Wilcoxon–Mann–Whitney tests revealed a lack of congruence of contemporary migration rates between biotically and abiotically dispersed species (*W* = 19.497, *p* = 0.003 in animal versus wind; *W* = 13.880, *p = 0.*042 in animal versus water), but significant congruence between water- and wind-dispersed plant species (*W* = 16.052, *p* = 0.610). These results indicated that the biotically dispersed plant species are more connected in the Rio Branco. All these findings were consistent when different datasets (i.e., pairs of sampling locations across river banks, and pairs of sampling locations along the banks of Rio Branco) were considered (Table 2).

For abiotically dispersed plant species, estimates of contemporary gene flow for all species were symmetric among all pairs of sampling locations along Rio Branco (Supplementary Table 7), except from *B. aequinoctialis*, in which asymmetrical migration rates were detected in two pairs of sampling locations (Supplementary Table 7). These estimates were observed from downstream to upstream direction (i.e., from 2L and 3L to 4L). Our data also suggest a source-sink relationship in *B. aequinoctialis* at the sample location 4L, which received a substantial proportion of migrants from the sample locations 2L (*m* = 0.1165, 95% CI 0.0538, 0.1792) and 3L (*m* = 0.1181, 95% CI 0.0507, 0.1855), while the expected proportion of migrants from 4L into 2L (*m* = 0.0162, 95% CI −0.0099, 0.0423), and 3L (*m* = 0.0137, 95% CI −0.0092, 0.0366) were much smaller. Asymmetrical migration rates were also detected among some pairs of sampling locations for the animal-dispersed plant species *A. longifolia*, *P. spinosa*, and *P. lupulina* (Supplementary Table 7).

We found no significant decrease in contemporary gene flow estimates associated with increasing geographic distance for any of the eight species sampled (*r* = −0.034, *p* = 0.642 in *A. schomburgkii*, *r* = −0.036, *p* = 0.623 in *A. paraense*, *r* = −0.112, *p* = 0.164 in *A. longifolia*, *r* = −0.115, *p* = 0.151 in *B. aequinoctialis*, *r* = −0.121, *p* = 0.132 in *P. spinosa*, *r* = −0.141, *p* = 0.079 in *P. lupulina*, *r* = 0.025, *p* = 0.740 in *P. kerere*, and *r* = −0.131, *p* = 0.104 in *T. pyramidatum*). For *P. kerere*, exclusively, we found a significant and positive association between contemporary migration rates and geographic distance, although this association was restricted to the right river bank of the Rio Branco (*r* = 0.44, *p* = 0.003; Supplementary Table 8).

Furthermore, no directional patterns of the contemporary migration rates (i.e., from upstream to downstream or downstream to upstream) were observed for all but one plant species (Supplementary Table 9). However, directional migration from upstream to downstream was observed just for *P. kerere* along the banks of the Rio Branco (Supplementary Table 9). This result is in line with the highest genetic diversity levels observed downstream for this plant species.

## Discussion

Life-history traits, such as dispersal mode/ability can have an effect on the species’ response to geographical and evolutionary processes (e.g., Waters et al., 2020), with contrasting effects on genetic structure (e.g., Bonada et al., 2017). In sessile organisms such as plants, distinct mechanisms of seed dispersal have evolved. Animal-mediated seed dispersal is thought to represent the most important dispersal mode in most tropical forests, in which ca. 50–90% of the plant species are vertebrate-dispersed (e.g., Howe and Smallwood, 1982; Jordano, 2000). In tropical riparian forest, an unknown but large proportion of fruits and seeds is dispersed by animals (e.g., Horn et al., 2011; Parolin et al., 2013; Correa et al., 2016). Although the correlation of dispersal mode with the level of genetic structure is dependent on the geographic scale (e.g., Nazareno et al., 2017a), riverine plant species that are animal-dispersed tend to have lower levels of genetic structure than species dispersed by other modes (e.g., Fér and Hroudová, 2008, 2009; Collevatti et al., 2009; Moreira and Fernandes, 2013; Nazareno et al., 2019a). Indeed, our findings support this expectation, with animal-dispersed plant species exhibiting lower levels of genetic differentiation than abiotically dispersed (i.e., by water or wind) species. It should be noted, however, that the data used to classify the primary seed dispersal mode were based on specific morphological features reported in the scientific literature – a common and relatively straightforward method used to study the influence of seed dispersal on population genetic structure (e.g., Muller-Landau and Hardesty, 2005; Duminil et al., 2007; Lowe et al., 2018; Gamba and Muchhala, 2020). Nonetheless, the categorization of dispersal mode using this approach did not change the outcome; regardless of dispersal mode, connectivity patterns in the eight riverine plant species studied revealed that the Rio Branco, a medium-sized river in the Amazon basin, is permeable, possibly even facilitating gene flow in water- and wind-dispersed plant species. These connectivity patterns seem to have changed historically, suggesting that the dynamic geological history of the Rio Branco may have impacted gene flow mainly in water- and wind-dispersed plant species – a result based on the difference between contemporary and historical dispersal estimates (Table 2). The methods employed here to understand how seed dispersal mode mediates gene flow in conjunction with the expected effects of geographic distance and barriers can be applied to other plant species to assess the impact of genetic connectivity on temporal and spatial patterns of divergence. Below, we discuss the implications of our results for understanding the impact of seed dispersal modes and riverscape on genetic variation within and among riverine plant populations and species.

### The Role of Dispersal Mode and Riverscape to Population Genetic Structure

The population structure observed for the five abiotically dispersed plant species studied here (i.e., *A. schomburgkii*, *A. paraense*, *B. aequinoctialis*, *P. kerere*, and *T. pyramidatum*) and those reported for three animal-dispersed plant species (i.e., *A. longifolia*, *P. spinosa*, and *P. lupulina*; Nazareno et al., 2019a) consistently showed a lack of genetic structure throughout the Rio Branco. This population genetic pattern is consistent with the island model proposed by Tero et al. (2003) to explain how dispersal shapes population differentiation in riverine plant populations. Indeed, multiple sources of evidence supported the island model of population structure (i.e., subpopulations exhibiting a genetic uniformity over the space; Tero et al., 2003) for the eight Amazonian riverine plants species studied. For example, the phylogenetic relationships among individual samples (Supplementary Figure 3), and the Bayesian and genetic distance-based clustering analyses (Supplementary Figure 4), grouped sampling locations separated by the river. Furthermore, when we tested for the presence of hierarchical population genetic structure, the low and non-significant proportion of the total variance that was attributed to the variance across river banks (Figure 3 and Supplementary Table 6) reinforces that the Rio Branco has facilitated dispersal even for riverine plant species that are not adapted to hydrochory. Furthermore, we also did not find evidence of isolation by distance (Supplementary Table 8), indicating fairly extensive historical and contemporary gene flow via seeds and/or pollen along and across banks of the Rio Branco. Although the population genetic studies in riverscapes differ from each other in landscape-scale sampling design, our results mirror the observed pattern of river systems promoting genetic similarity among subpopulations in other riverine plant species (e.g., Kitamoto et al., 2005; Collevatti et al., 2009; Moreira and Fernandes, 2013; Hopley and Byrne, 2018; Lee et al., 2018; Murray et al., 2019). This genetic pattern can vary among taxa though (e.g., Tero et al., 2003; Prentis et al., 2004; Liu et al., 2006; Honnay et al., 2010; Wei et al., 2015; Hopley and Byrne, 2018; Hernández-Leal et al., 2019). For example, in a river in the southwest of Australia, Hopley and Byrne (2018) reported distinct patterns of genetic differentiation and gene flow in *Callistachys lanceolata* and *Astartea leptophylla*, showing how abiotic factors (e.g., geographic distance and distribution range) shape the genetic structure of shrub plant species with different seed dispersal modes.

The link between dispersal mode and degree of genetic structure for riverine plant species (e.g., Fér and Hroudová, 2008; Wei et al., 2015; Nazareno et al., 2017a, 2019a) is still less clear than those observed for plant species across continuous landscapes (e.g., Hamrick et al., 1993; Duminil et al., 2007; Lowe et al., 2018; Gamba and Muchhala, 2020). Despite that, riverine plant species that are animal-dispersed tend to show lower levels of genetic structure than species dispersed by other modes (e.g., Fér and Hroudová, 2008, 2009; Collevatti et al., 2009; Moreira and Fernandes, 2013; Nazareno et al., 2019a; but see Nazareno et al., 2019b), slowing down population genetic differentiation (e.g., Collevatti et al., 2009; Moreira and Fernandes, 2013). While few plant studies employed a sampling scheme to test the effect of geographic distance across river banks on genetic structure (e.g., Collevatti et al., 2009; Moreira and Fernandes, 2013; Nazareno et al., 2017a, 2019a,b), patterns of genetic differentiation have been assessed along river banks (e.g., Tero et al., 2003; Prentis et al., 2004; Liu et al., 2006; Honnay et al., 2010; Rogalski et al., 2017), as well as among different river systems or basins (e.g., Kitamoto et al., 2005; Fér and Hroudová, 2008; Love et al., 2013; Hevroy et al., 2018; Hopley and Byrne, 2018; Lee et al., 2018; Hernández-Leal et al., 2019; Murray et al., 2019). However, riverscape features such as the geographic distance across river banks may also contribute to shaping the spatial distribution of the genetic diversity, shedding light on the role of physical barriers to dispersal of riverine plant species. For example, Nazareno et al. (2019b) reported a strong genetic differentiation among populations of the primate-dispersed and bee-pollinated tree species *Buchenavia oxycarpa* on opposite banks of the Rio Negro in the Amazon basin. This trend is not consistent with what has been documented in two animal-dispersed tree species (e.g., *Caryocar villosum* and *C. microcarpum*) in this large-sized river (Collevatti et al., 2009). The long-distance of pollen gene flow mediated by bat species, coupled with the seed dispersal by strong swimming tapirs and fish (Collevatti et al., 2009), helps to explain those findings in the *Caryocar* species. While geographic distances up to 12.0 km seem to prevent gene flow between *B. oxycarpa* populations across river banks, Nazareno et al. (2019b) reported that pollen and seeds of *B. oxycarpa* were dispersed at long-distances along river banks, with gene flow occurring up to 84 km along banks of the Rio Negro. This lack of isolation by distance and extensive gene flow within a river bank was also documented for *A. longifolia* in the Rio Negro (Nazareno et al., 2017a), as well as for a few other riverine plant species growing along different river systems (e.g., Kitamoto et al., 2005; Honnay et al., 2010; Hopley and Byrne, 2018). However, in the Rio Branco, a medium-sized Amazon river, *A. longifolia* appears to constitute a panmictic population (Nazareno et al., 2017a, 2019a). These findings suggest that large-sized Amazon rivers can contribute to structuring plant populations, while small- to medium-sized rivers can contribute to the maintenance of the levels of genetic diversity in riverine plant species. Pollination mode has been shown to represent another important factor shaping genetic structure for different plant populations (Gamba and Muchhala, 2020), indicating that other life-history traits such as pollination mode should also be considered in studies of this nature (Collevatti et al., 2009). Despite the importance of pollination mode, seed dispersal mode did not unequivocally determine genetic structure within our study system. The link between gene dispersal by pollen and seeds and the genetic structure of riverine plant species remains to be documented.

### The Role of Dispersal Mode to Intrapopulation Genetic Diversity

Although the population genetic structure observed for the eight plant species was similar in the Rio Branco, the levels of intrapopulation genetic diversity differed significantly among animal-, wind-, and water-dispersed plant species (Figure 2). This result corroborates the intra-population genetic diversity patterns reported for other Neotropical plant species with distinct seed dispersal modes (e.g., Lowe et al., 2018).

Because the levels of genetic diversity are species-specific, comparisons across species need to be interpreted with care. As far as within-population genetic diversity is concerned, higher levels of unbiased *H*_*E*_ were observed for animal-dispersed than for water- and wind-dispersed species. Although water-dispersed plant species have been neglected in studies investigating links between seed dispersal mode and the levels of genetic diversity (e.g., Duminil et al., 2007; Gamba and Muchhala, 2020), our results are in line with the findings reported by animal-dispersed plant species in continuous landscapes (e.g., Hamrick et al., 1992, 1993; Hamrick and Godt, 1996; Vieira et al., 2010; Bustamante et al., 2016; but see Thiel-Egenter et al., 2008), and riverscapes (e.g., Collevatti et al., 2009; Moreira and Fernandes, 2013). These trends imply that plant-animal interactions can help to keep high levels of intrapopulation diversity and to prevent the loss of genetic diversity (e.g., Nazareno and Carvalho, 2009; Vieira et al., 2010; Bustamante et al., 2016), mainly for outcrossed plant species (e.g., Ellegren and Galtier, 2016). Besides the high levels of genetic diversity observed, the three animal-dispersed plant species showed a lack of (biparental) inbreeding. These results contrast with those observed for abiotically dispersed riverine plant species (e.g., Lundqvist and Andersson, 2001; Liu et al., 2006; Hmeljevski et al., 2011; Hopley and Byrne, 2018), and the findings documented for the five inbreeding abiotically dispersed liana species, all of which showed low levels of genetic diversity (Figure 2), suggesting that the plant species studied here likely share similar mating systems. However, our understanding of how genes are recombined and maintained by these plant species would greatly benefit from future mating system studies (e.g., estimating the multilocus and single-locus outcrossing rates, selfing rates, and biparental inbreeding rates).

Two of the animal-dispersed plant species studied, *P. spinosa* (i.e., a liana likely pollinated by hummingbirds and potentially dispersed by fishes and mammals) and *P. lupulina* (i.e., a shrub pollinated by small bees and dispersed by fishes and birds) showed higher levels of genetic variation (i.e., *H*_*E*_ and *H*_*O*_) than *A. longifolia*. The effectiveness of seed dispersion in *A. longifolia* – a plant species with a mixed seed dispersal mode (i.e., animal and ballistic dispersal) – can be influenced by flood dynamics throughout the year, which may be more restricted in periods of non-flooding, especially if the plant is located far from the river banks. Thus, the contribution of ballistic dispersal may have a negative effect on the levels of intrapopulation genetic diversity in *A. longifolia*. Indeed, animal-dispersed plant species tend to have higher levels of genetic diversity than ballistic-dispersed plant species (e.g., Hamrick and Godt, 1996). However, based on genetic data generated for 23 Neotropical tree species (Lowe et al., 2018), no differences of the average intrapopulation genetic diversity were observed between animal- and ballistic-dispersed plant species neither between animal-dispersed plant species and plant species with a mixed mode of seed dispersal (e.g., animal and ballistic). While these results were obtained in plant species sampled across continuous landscapes (Lowe et al., 2018), a multispecies-scale study is still needed to better elucidate the intrapopulation genetic diversity in riverine plant species.

Contrary to what is expected for species closely-related phylogenetically (e.g., Carvalho et al., 2019), the levels of genetic diversity for the five riverine plant species of the Bignoniaceae were different between water- and wind-dispersed plant species, indicating that the mode of seed dispersal seems to impact the patterns of intrapopulation genetic diversity. For the three wind-dispersed plant species studied here, *A. schomburgkii*, *B. aequinoctialis*, and *T. pyramidatum*, no significant differences among the levels of genetic diversity were detected (Figure 2). All these species share the same growth form (i.e., climbing habitat) and pollination mode (i.e., melittophily). However, for the two water-dispersed plant species studied (i.e., *A. paraense* and *P. kerere*), life-history traits such as pollination mode and growth form (Table 1) do not explain the differences observed in their levels of genetic diversity, suggesting that other factors (e.g., population size, plant density, phenological pattern, successional stage, and range size) or a combination of these factors might impact the intrapopulation genetic diversity levels. Considering the range size of the two water-dispersed plant species studied, *P. kerere* which has a more restricted range size than *A. paraense* (Meyer et al., 2018), presented lower levels of genetic diversity than *A. paraense* – a pattern reported in other plant species with contrasting range sizes (e.g., Loveless and Hamrick, 1984; Lowe et al., 2018).

### Spatial Patterns of Genetic Diversity in Riverine Plant Species

Finally, we explored the Drift Paradox hypothesis (Ritland, 1989) in order to understand how riverscape features (e.g., the unidirectional river water flow) can affect the distribution of the genetic diversity in riverine plant species with distinct dispersal modes. This hypothesis predicts that intraspecific genetic diversity increases downward in a river system, with less diverse upstream populations. This pattern can be expected even for plant species that are not adapted to hydrochory as these taxa can be dispersed secondarily by water (Boedeltje et al., 2003; Prots et al., 2011). Paradoxically to what has been observed in other taxa (e.g., fish, arthropods, mollusks, and amphibians; Blanchet et al., 2020), our results indicate that all but one riverine plant species studied, including the water-dispersed plant species *A. paraense*, do not fit with the Drift Paradox expectation, an outcome also reported for a plethora of plant species (e.g., Ritland, 1989; Tero et al., 2003; Honnay et al., 2010; Hmeljevski et al., 2011; Wei et al., 2015; Hevroy et al., 2018; Nazareno et al., 2019a; Murray et al., 2019; Blanchet et al., 2020). This might be due to the fact that gene flow in riverine plant species is not exclusively dependent on water (Blanchet et al., 2020), with contributions of dispersal by seeds and pollen varying among plant species (Dick et al., 2008). However, the water-dispersed plant species *P. kerere* has provided evidence of downstream accumulation of genetic diversity (Supplementary Figure 2), mirroring the pattern found in a few other plant species (e.g., Gornall et al., 1998; Lundqvist and Andersson, 2001; Liu et al., 2006; Fér and Hroudová, 2008; Love et al., 2013; Rogalski et al., 2017), including an annual grass species in the Amazon basin (Akimoto et al., 1998). The result observed for *P. kerere* is in line with their directional pattern of contemporary migration from upstream to downstream (Supplementary Table 9), suggesting that seed dispersal and not pollen-mediated gene flow is shaping the pattern of gene flow and genetic diversity in *P. kerere*. However, the contrasting results observed for the two water-dispersed plant species suggests that the seeds of *A. paraense* might be dispersed upstream by secondary dispersal through animals, a diplochory seed dispersal process also reported for other water-dispersed plant species (e.g., Honnay et al., 2010; Horn et al., 2011; Murray et al., 2019). These distinct patterns of seed dispersal in *P. kerere* and *A. paraense* can also explain the differences observed in their intrapopulation genetic diversity estimates (Figure 2).

On the other hand, since the locomotion of animals and wind currents do not necessarily follow the river water flow, the lack of downstream accumulation of genetic diversity for the other six riverine plant species suggests that water can play a secondary role in the dispersal process for animal- and wind-dispersed plant species. Yet, the lack of accumulation of genetic diversity in downstream sampling locations can be due to the higher seed recruitment opportunities in upstream habitats due to the density dependence of recruitment, although this ecological pattern remains to be tested. Nonetheless, most estimates of contemporary gene flow were symmetrical between pairs of sampling locations, even for one water-dispersed plant species (Supplementary Table 4), suggesting that pollen-mediated gene flow across and along the river banks may have homogenized genetic diversity in these riverine plant species. Indeed, it is important to take into account the fact that the distribution of the genetic variation in plant populations might also be determined by distinct factors, especially pollen-mediated gene flow (e.g., Ghazoul, 2007; Dick et al., 2008). Although pollen-mediated gene flow across continuous landscapes seems to be effective only within hundreds of meters decreasing with an increase of geographical distance (e.g., Richards et al., 1999; Tero et al., 2003; but see Bain et al., 2016), additional studies using molecular markers with different modes of inheritance are still needed for an improved understanding of the role of seed dispersal and pollen-mediated gene flow on genetic patterns in riverine plant species.

### Concluding Remarks

Even at a small spatial scale, the results of our intraspecific population genomic structure study in plant species displaying distinct dispersal modes corroborates previous findings that ecology rather than geography play a key role in shaping the evolutionary history of plants in the Amazon basin (e.g., Lavin, 2006; Pennington and Dick, 2010; Dexter et al., 2017). A clear lack of geographic phylogenetic structure due to dispersal throughout the Amazonian rainforest was reported in large trees belonging to different plant clades (Dexter et al., 2017; but see Monro, 2006). Taking into account that all the eight plant species studied here have ranges extending far beyond the Rio Branco (Taylor, 2007; Braun et al., 2012; Lohmann and Taylor, 2014), putative dispersal barriers such as rivers do not seem to have prevented the successful dispersion of these taxa across the Amazon basin. We argue, however, that wide geographic distribution patterns do not necessarily imply a lack of population structure or a lack of phylogeographic breaks (e.g., Dexter et al., 2012; Nazareno et al., 2017a; Honorio Coronado et al., 2019; Nazareno et al., 2019b) in Amazonian plant species.

As noticed before, we showed that medium-sized rivers in the Amazon basin, such as the Rio Branco, can act as permeable barriers facilitating – rather than preventing – gene flow in riverine plant species with distinct seed dispersal modes. This pattern may have arisen due to trade-offs between extinction and recolonization. However, further studies based on data derived from multiple taxa sampled at the community level throughout the Amazon river system are needed to further understand how macroevolutionary processes influence the population structure of riverine plant species. The findings reported here can help establish better management plans to safeguard riparian forest areas. Considering the relevance of such areas for the provision of essential ecosystem services (e.g., conservation of biological diversity, protection of hydrological flows and soils, maintenance of water quality) these results can help guide conservation and restoration policies in Amazonian riparian forests areas, where degradation and deforestation rates are rising (Biggs et al., 2019; Nunes et al., 2019). Yet, since the Rio Branco does not actually act as a barrier, local adaptation is less likely and seed sources may not correspond to areas defined by rivers (e.g., Leimu and Fischer, 2008; De Kort et al., 2014; Hopley and Byrne, 2018). Further comparative population genomic approaches that take different geographic scales into account, including Amazon waterways with distinct environmental conditions, are needed to consolidate our knowledge on the degree to which seed dispersal and pollination mode mediate gene flow in conjunction with the generalized expected effects of spatial distance and barriers. Such studies would allow us to better understand how biotic and abiotic factors mediate gene flow in riparian forests in the Amazon basin and, other riverscapes worldwide.

## Data Availability Statement

SNP datasets for the *Amphirrhox longifolia*, *Passiflora spinosa*, and *Psychotria lupulina* populations are available for download from the Dryad Digital Repository (https://doi.org/10.5061/dryad.f53j0dk). For *Adenocalymma schomburgkii*, *Anemopaegma paraense*, *Bignonia aequinoctialis*, *Pachyptera kerere*, and *Tanaecium pyramidatum* populations, the SNP datasets are available for download at https://doi.org/10.5061/dryad.573n5tb6c.

## Author Contributions

AGN and LGL designed the study and coordinated sample collection. AGN conducted molecular work, performed analyses, and led the writing of the manuscript with input from all co-authors. LLK provided analytical support and troubleshooting. CWD provided laboratory assistance. All authors contributed to the article and approved the submitted version.

## Conflict of Interest

The authors declare that the research was conducted in the absence of any commercial or financial relationships that could be construed as a potential conflict of interest.

## Footnotes

1 http://tree.bio.ed.ac.uk/software/figtree/

## References

[B1] Akimoto M. Shimamoto Y. Morishima H. (1998). Population genetic structure of wild rice *Oryza glumaepatula* distributed in the Amazon flood area influenced by its life-history traits. *Mol. Ecol.* 7 1371–1381. 10.1046/j.1365-294x.1998.00485.x

[B2] Alcantara S. Lohmann L. G. (2010). Evolution of floral morphology and pollination system in Bignonieae (Bignoniaceae). *Am. J. Bot.* 97 782–796. 10.3732/ajb.0900182 21622444

[B3] Bain A. Borges R. M. Chevallier M. H. Vignes H. Kobmoo N. Peng Y. Q. (2016). Geographic structuring into vicariant species-pairs in a wide-ranging, high dispersal plant-insect mutualism: the case of Ficus racemosa and its pollinating wasps. *Evol. Ecol.* 30 663–684. 10.1007/s10682-016-9836-5

[B4] Ballesteros-Mejia L. Lima N. E. Lima-Ribeiro M. S. Collevatti R. G. (2016). Pollination mode and mating system explain patterns in genetic differentiation in Neotropical plants. *PLoS One* 11:e0158660. 10.1371/journal.pone.0158660 27472384 PMC4966973

[B5] Barnholtz-Sloan J. S. (2003). “Population genetics,” in *Introduction to Bioinformatics*, eds Krawetz S. A. Womble D. D. (Totowa, NJ: Humana Press), 207–227.

[B6] Biggs T. W. Santiago T. M. O. Sills E. Caviglia-Harris J. (2019). The Brazilian Forest Code and riparian preservation areas: spatiotemporal analysis and implications for hydrological ecosystem services. *Reg. Environ. Change* 19 2381–2394. 10.1007/s10113-019-01549-w

[B7] Blanchet S. Prunier J. G. Paz-Vinas I. Saint-Pé K. Rey O. Raffard A. (2020). A river runs through it: the causes, consequences, and management of intraspecific diversity in river networks. *Evol. App.* 13 1195–1213. 10.1111/eva.12941 PMC7359825 32684955

[B8] Boedeltje G. Bakker J. P. Bekker R. M. Van Groenendael J. M. Soesbergen M. (2003). Plant dispersal in a lowland stream in relation to occurrence and three specific life-history traits of the species in the species pool. *J. Ecol.* 91 855–866. 10.1046/j.1365-2745.2003.00820.x

[B9] Bonada N. Carlson S. M. Datry T. Finn D. S. Leigh C. Lytle D. A. (2017). Genetic, evolutionary, and biogeographical processes in intermittent rivers and ephemeral streams. *Int. Rivers Ephemeral Streams* 2017 405–431. 10.1016/b978-0-12-803835-2.00015-2

[B10] Bonin A. Ehrich D. Manel S. (2007). Statistical analysis of amplified fragment length polymorphism data: a toolbox for molecular ecologists and evolutionists. *Mol. Ecol.* 16 3737–3758. 10.1111/j.1365-294x.2007.03435.x 17850542

[B11] Boubli J. P. Ribas C. Alfaro J. W. L. Alfaro M. E. da Silva M. N. Pinho G. M. (2015). Spatial and temporal patterns of diversification on the Amazon: a test of the riverine hypothesis for all diurnal primates of Rio Negro and Rio Branco in Brazil. *Mol. Phylogenet. Evol.* 82 400–412. 10.1016/j.ympev.2014.09.005 25285613

[B12] Braun M. Dotter S. Schlindwein C. Gottsberger G. (2012). Can nectar be a disadvantage? Contrasting pollination natural histories of two woody Violaceae from the Neotropics. *Int. J. Plant Sci.* 173 161–171. 10.1086/663167

[B13] Bustamante E. Búrquez A. Scheinvar E. Eguiarte L. E. (2016). Population genetic structure of a widespread bat-pollinated colummar cactus. *PLoS One* 11:e0152329. 10.1371/journal.pone.0152329 27015281 PMC4820105

[B14] Carvalho Y. G. S. Vitorino L. C. Souza U. J. B. Bessa L. A. (2019). Recent trends in research on the genetic diversity of plants: implications for conservation. *Diversity* 11:62. 10.3390/d11040062

[B15] Catchen J. M. Amores A. Hohenlohe P. Cresko W. Postlethwait J. H. (2011). Stacks: building and genotyping loci de novo from short-read sequences. *G3* 1 171–182. 10.1534/g3.111.000240 22384329 PMC3276136

[B16] Catchen J. M. Hohenlohe P. A. Bassham S. Amores A. Cresko W. A. (2013). Stacks: an analysis tool set for population genomics. *Mol. Ecol.* 22 3124–3140. 10.1111/mec.12354 23701397 PMC3936987

[B17] Collevatti R. G. Leoi L. C. T. Leite S. A. Gribel R. (2009). Contrasting patterns of genetic structure in Caryocar (Caryocaraceae) congeners from flooded and upland Amazonian forests. *Biol. J. Linn. Soc.* 98 278–290. 10.1111/j.1095-8312.2009.01287.x

[B18] Correa S. B. Araujo J. K. Penha J. Cunha C. N. Bobier K. E. Anderson J. T. (2016). Stability and generalization in seed dispersal networks: a case study of frugivorous fish in Neotropical wetlands. *Proc. R. Soc. B* 283 20161267. 10.1098/rspb.2016.1267 27581879 PMC5013796

[B19] Correa S. B. Costa-Pereira R. Fleming T. Goulding M. Anderson J. T. (2015). Neotropical fish-fruit interactions: Eco-evolutionary dynamics and conservation. *Biol. Rev. Camb. Philos. Soc.* 90 1263–1278. 10.1111/brv.12153 25599800

[B20] De Kort H. Mergeay J. Mijnsbrugge K. V. Decocq G. Maccherini S. Bruun H. H. K. (2014). An evaluation of seed zone delineation using phenotypic and population genomic data on black alder *Alnus glutinosa*. *J. App. Ecol.* 51 1218–1227. 10.1111/1365-2664.12305

[B21] De Leeuw J. (1977). Correctness of Kruskal’s algorithms for monotone regression with ties. *Psychometrika* 42 141–144. 10.1007/bf02293750

[B22] Dexter K. G. Lavin M. Torke B. M. Twyford A. D. Kursar T. A. Coley P. D. (2017). Dispersal assembly of rain forest tree communities across the Amazon basin. *Proc. Natl. Acad. Sci. U.S.A.* 114 2645–2650. 10.1073/pnas.1613655114 28213498 PMC5347625

[B23] Dexter K. G. Terborgh J. W. Cunningham C. W. (2012). Historical effects on beta diversity and community assembly in Amazonian trees. *Proc. Natl. Acad. Sci. U.S.A.* 109 7787–7792. 10.1073/pnas.1203523109 22547831 PMC3356654

[B24] Dick C. W. Hardy O. J. Jones F. A. Petit R. J. (2008). Spatial scales of pollen and seed-mediated gene flow in tropical rain forest trees. *Trop. Plant Biol.* 1 20–33. 10.1007/s12042-007-9006-6

[B25] Duminil J. Fineschi S. Hampe A. Jordano P. Salvini D. Vendramin G. G. (2007). Can population genetic structure be predicted from life-history traits? *Am. Nat.* 169 662–672. 10.2307/4137044 17427136

[B26] Ellegren H. Galtier N. (2016). Determinants of genetic diversity. *Nature Rev. Genet.* 17 422–433. 27265362 10.1038/nrg.2016.58

[B27] Excoffier L. Lischer H. E. L. (2010). Arlequin suite ver 3.5: a new series of programs to perform population genetics analyses under Linux and Windows. *Mol. Ecol. Res.* 10 564–567. 10.1111/j.1755-0998.2010.02847.x 21565059

[B28] Excoffier L. Smouse P. Quattro J. (1992). Analysis of molecular variance inferred from metric distances among DNA haplotypes: Application to human mitochondrial DNA restriction data. *Genetics* 131 479–491. 1644282 10.1093/genetics/131.2.479 PMC1205020

[B29] Fearnside P. M. (2015). *Hidrelétricas na Amazônia: Impactos Ambientais e Sociais na Tomada de Decisñes Sobre Grandes obras.* Manaus: Editora do INPA.

[B30] Fér T. Hroudová Z. (2008). Detecting dispersal of Nuphar lutea in river corridors using microsatellite markers. *Freshw. Biol.* 53 1409–1422. 10.1111/j.1365-2427.2008.01973.x

[B31] Fér T. Hroudová Z. (2009). Genetic diversity and dispersal of *Phragmites australis* in a small river system. *Aquat. Bot.* 90 165–171. 10.1016/j.aquabot.2008.09.001

[B32] Foll M. Gaggiotti O. E. (2008). A genome scan method to identify selected loci appropriate for both dominant and codominant markers: a Bayesian perspective. *Genetics* 180 977–993. 10.1534/genetics.108.092221 18780740 PMC2567396

[B33] Fonseca L. H. M. Lohmann L. G. (2019). An updated synopsis of Adenocalymma (Bignonieae, Bignoniaceae): new combinations, synonyms, and lectotypifications. *Syst. Bot.* 44 893–912. 10.1600/036364419x15710776741341

[B34] Fouquet A. Courtois E. A. Baudain D. Lima J. D. (2015). The trans-riverine genetic structure of 28 Amazonian frog species is dependent on life history. *J. Trop. Ecol.* 31 361–373. 10.1017/s0266467415000206

[B35] Francisco J. N. C. Lohmann L. G. (2018). Taxonomic revision of Pachyptera (Bignonieae, Bignoniaceae). *PhytoKeys* 92 89–131. 10.3897/phytokeys.92.20987 29416412 PMC5799743

[B36] Frazão A. Lohmann L. G. (2019). An updated synopsis of Tanaecium (Bignonieae, Bignoniaceae). *PhytoKeys* 132 31–52. 10.3897/phytokeys.132.37538 31598067 PMC6776558

[B37] Gamba D. Muchhala N. (2020). Global patterns of population genetic differentiation in seed plants. *Mol. Ecol.* 29 3413–3428. 10.1111/mec.15575 32743850

[B38] Ghazoul J. (2007). Pollen and seed dispersal among dispersed plants. *Biol. Rev.* 80 413–443. 10.1017/s1464793105006731 16094807

[B39] Giombini M. I. Bravo S. P. Sica Y. V. Tosto D. S. (2017). Early genetic consequences of defaunation in a large-seeded vertebrate-dispersed palm (*Syagrus romanzoffiana*). *Heredity* 118 568–577. 10.1038/hdy.2016.130 28121308 PMC5436022

[B40] Gornall R. J. Hollingsworth P. M. Preston C. D. (1998). Evidence for spatial and directional gene flow in a population of an aquatic plant, *Potamogeton coloratus*. *Heredity* 80 414–421. 10.1046/j.1365-2540.1998.00270.x

[B41] Goudet J. (2005). HIERFSTAT, a package for R to compute and test hierarchical F-statistics. *Mol. Ecol. Notes* 5 184–186. 10.1111/j.1471-8286.2004.00828.x

[B42] Guillot G. (2012). *Population Genetic and Morphometric Data Analysis Using R and the Geneland Program.* Available online at: http://www2.imm.dtu.dk/~gigu/Geneland/Geneland-979 (accessed January 20, 2020).

[B43] Guillot G. Santos F. Estoup A. (2008). Analyzing georeferenced population genetics data with Geneland: a new algorithm to deal with null alleles and a friendly graphical user interface. *Bioinformatics* 24 1406–1407. 10.1093/bioinformatics/btn136 18413327

[B44] Hamrick J. L. Godt M. J. W. (1996). Effects of life history traits on genetic diversity in plant species. *Philos. Trans. R. Soc. B* 351 1291–1298. 10.1098/rstb.1996.0112

[B45] Hamrick J. L. Godt M. J. W. Sherman-Broyles S. L. (1992). Factors influencing levels of genetic diversity in woody plant species. *New Forests* 6 95–124. 10.1007/bf00120641

[B46] Hamrick J. L. Murawski D. A. Nason J. D. (1993). The influence of seed dispersal mechanisms on the genetic structure of tropical tree populations. *Vegetatio* 108 281–297. 10.1007/978-94-011-1749-4_20

[B47] Hernández-Leal M. Suárez-Atilano M. Piñero D. González-Rodríguez A. (2019). Regional patterns of genetic structure and environmental differentiation in willow populations (*Salix humboldtiana* Willd.) from Central Mexico. *Ecol. Evol.* 9 9564–9579. 10.1002/ece3.5475 31534675 PMC6745842

[B48] Hevroy T. H. Moody M. L. Krauss S. L. (2018). Population genetic analysis reveals barriers and corridors for gene flow within and among riparian populations of a rare plant. *AoB Plants* 10 lx065. 10.1093/aobpla/plx065 PMC5751030 29308125

[B49] Hmeljevski K. V. Reis A. Montagna T. Reis M. S. (2011). Genetic diversity, genetic drift and mixed mating system in small subpopulations of *Dyckia ibiramensis* a rare endemic bromeliad from Southern Brazil. *Conserv. Genet.* 12, 761–769. 10.1007/s10592-011-0183-3

[B50] Hodel R. G. J. Knowles L. L. Dunaway J. F. Payton A. C. McDaniel S. F. Soltis P. S. (2018). Terrestrial species adapted to sea dispersal: differences in propagule dispersal of two Caribbean mangroves. *Mol. Ecol.* 27 4612–4626. 10.1111/mec.14894 30308703

[B51] Hohenlohe P. A. Bassham S. Etter P. D. Stiffler N. Johnson E. A. Cresko W. A. (2010). Population genomics of parallel adaptation in three-spine stickleback using sequenced RAD tags. *PLoS Genet.* 6:e1000862. 10.1371/journal.pgen.1000862 20195501 PMC2829049

[B52] Holsinger K. E. Weir B. S. (2009). Genetics in geographically structured populations: defining, estimating and interpreting FST. *Nat. Rev. Genet.* 10 639–650. 10.1038/nrg2611 19687804 PMC4687486

[B53] Honnay O. Jacquemyn H. Nackaerts K. Breyne P. Van Looy K. (2010). Patterns of population genetic diversity in riparian and aquatic plant species along rivers. *J. Biog.* 37 1730–1739. 10.1111/j.1365-2699.2010.02331.x

[B54] Honorio Coronado E. N. Dexter K. G. Hart M. L. Phillips O. L. Pennington R. T. (2019). Comparative phylogeography of five widespread tree species: insights into the history of western Amazonia. *Ecol. Evol.* 9 7333–7345. 10.1002/ece3.5306 31380054 PMC6662334

[B55] Hopley T. Byrne M. (2018). Connectivity in riparian plants: influence of vegetation type and habitat fragmentation overrides water flow. *Oecologia* 188 465–478. 10.1007/s00442-018-4226-z 30039200

[B56] Horn M. H. Correa S. B. Parolin P. Pollux B. J. A. Anderson J. T. Lucas C. (2011). Seed dispersal by fishes in tropical and temperate fresh waters: the growing evidence. *Acta Oecol.* 37 561–577. 10.1016/j.actao.2011.06.004

[B57] Howe H. F. Smallwood J. (1982). Ecology of seed dispersal. *Ann. Rev. Ecol. Evol. Syst.* 13 201–228.

[B58] Jombart T. (2008). Adegenet: a R package for the multivariate analysis of genetic markers. *Bioinformatics* 24 1403–1405. 10.1093/bioinformatics/btn129 18397895

[B59] Jombart T. Ahmed I. (2011). Adegenet 1.3-1: new tools for the analysis of genome-wide SNP data. *Bioinformatics* 27 3070–3071. 10.1093/bioinformatics/btr521 21926124 PMC3198581

[B60] Jordano P. (2000). *Seeds: The Ecology of Regeneration in Plant Communities*, ed. Fenner M. (Wallingford, Oxfordshire: CAB International), 125–166.

[B61] Keenan K. McGinnity P. Cross T. F. Crozier W. W. Prodohl P. A. (2013). diveRsity: an R package for the estimation and exploration of population genetics parameters and their associated errors. *Meth. Ecol. Evol.* 4 782–788. 10.1111/2041-210x.12067

[B62] Kitamoto N. Honjo M. Ueno S. Takenaka A. Tsumura Y. Washitani I. (2005). Spatial genetic structure among and within populations of *Primula sieboldii* growing beside separate streams. *Mol. Ecol.* 14 149–157. 10.1111/j.1365-294x.2004.02398.x 15643958

[B63] Kotsakiozi P. Richardson J. B. Pichler V. Favia G. Martins A. J. Urbanelli S. (2017). Population genomics of the Asian tiger mosquito, *Aedes albopictus*: insights into the recent worldwide invasion. *Ecol. Evol.* 7 10143–10157. 10.1002/ece3.3514 29238544 PMC5723592

[B64] Latrubesse E. M. Stevaux J. C. Sinha R. (2005). Tropical rivers. *Geomorphology* 70 187–206.

[B65] Lavin M. (2006). “Floristic and geographic stability of discontinuous seasonally dry tropical forests explains patterns of plant phylogeny and endemism,” in *Neotropical Savannas and Seasonally Dry Forests: Plant Biodiversity, Biogeography and Conservation*, eds Pennington R. T. Ratter J. A. Lewis G. P. (Boca Raton, FL: CRC Press), 433–447. 10.1201/9781420004496-19

[B66] Lee S.-R. Jo Y.-S. Park C.-H. Friedman J. M. Olson M. S. (2018). Population genomic analysis suggests strong influence of river network on spatial distribution of genetic variation in invasive saltcedar across the southwestern United States. *Mol. Ecol.* 27 636–646. 10.1111/mec.14468 29274176

[B67] Leimu R. Fischer M. (2008). A meta-analysis of local adaptation in plants. *PLoS One* 3:e4010. 10.1371/journal.pone.0004010 19104660 PMC2602971

[B68] Liu Y. Wang Y. Huang H. (2006). High interpopulation genetic differentiation and unidirectional linear migration patterns in *Myricaria laxiflora* (Tamaricaceae), an endemic riparian plant in the Three Gorges Valley of the Yangtze River. *Am. J. Bot.* 93 206–215. 10.3732/ajb.93.2.206 21646181

[B69] Lohmann L. G. Taylor C. M. (2014). A new generic classification of Tribe Bignonieae (Bignoniaceae). *Ann. Miss. Bot. Gard.* 99 348–489. 10.3417/2003187

[B70] Love H. M. Maggs C. A. Murray T. E. Provana J. (2013). Genetic evidence for predominantly hydrochoric gene flow in the invasive riparian plant *Impatiens glandulifera* (Himalayan balsam). *Ann. Bot.* 112 1743–1750. 10.1093/aob/mct227 24169594 PMC3838552

[B71] Loveless M. D. Hamrick J. L. (1984). Ecological determinants of genetic structure in plant populations. *Ann. Rev. Ecol. Syst.* 15 65–95. 10.1146/annurev.es.15.110184.000433

[B72] Lowe A. J. Breed M. F. Caron H. Colpaert N. Dick C. W. Finegan B. (2018). Standardised genetic diversity-life history correlates for improved genetic resource management of Neotropical trees. *Divers. Dist.* 24 730–741. 10.1111/ddi.12716

[B73] Lundqvist E. Andersson E. (2001). Genetic diversity in populations of plants with different breeding and dispersal strategies in a free-flowing boreal river system. *Hereditas* 135 75–83. 10.1111/j.1601-5223.2001.00075.x 12035618

[B74] Macedo M. Prance G. T. (1978). Notes on the vegetation of Amazonia II. The dispersal of plants in Amazonian white sand campinas: the campinas as functional islands. *Brittonia* 30 203–215. 10.2307/2806654

[B75] Malécot G. (1948). *Les Mathématiques de l’Hérédié.* Paris: Masson.

[B76] Mantel N. (1967). The detection of disease clustering and a generalized regression approach. *Cancer Res.* 27 209–220. 6018555

[B77] Medina I. Cooke G. M. Ord T. J. (2018). Walk, swim or fly? Locomotor mode predicts genetic differentiation in vertebrates. *Ecol. Lett.* 21 638–645. 10.1111/ele.12930 29527800

[B78] Meyer L. Diniz-Filho J. A. F. Lohmann L. G. (2018). A comparison of hull methods for estimating species ranges and richness maps. *Plant Ecol. Diversity* 10 389–401. 10.1080/17550874.2018.1425505

[B79] Monro A. K. (2006). The revision of species-rich genera: a phylogenetic framework for the strategic revision of Pilea (Urticaceae) based on cpDNA, nrDNA, and morphology. *Am. J. Bot.* 93 426–441. 10.3732/ajb.93.3.426 21646202

[B80] Moreira P. A. Fernandes G. (2013). Is the São Francisco River a geographic barrier to gene flow in trees of Handroanthus ochraceus? *J. Trop. Ecol.* 29 243–250. 10.1017/s0266467413000217

[B81] Muller-Landau H. C. Hardesty B. D. (2005). “Seed dispersal of woody plants in tropical forests: concepts, examples and future directions,” in *Biotic Interactions in the Tropics*, eds Burslem D. Pinard M. Hartley S. (Cambridge: Cambridge University Press), 267–309. 10.1017/cbo9780511541971.012

[B82] Murray B. F. Reid A. M. Capon S. J. Thoms M. Wu S.-B. (2019). Gene flow and genetic structure in *Acacia stenophylla* (Fabaceae): effects of hydrological connectivity. *J. Biog.* 46 1138–1151. 10.1111/jbi.13566

[B83] Mussmann S. M. Douglas M. R. Chafin T. K. Douglas M. E. (2019). BA3-SNPs: contemporary migration reconfigured in BayesAss for next-generation sequence data. *Methods Ecol. Evol.* 10, 1808–1813. 10.1111/2041-210X.13252

[B84] Naka L. N. Bechtoldt C. L. Henriques L. M. Brumfield R. T. (2012). The role of physical barriers in the location of avian suture zones in the Guiana Shield, northern Amazonia. *Am. Nat.* 179 E115–E132. 22437185 10.1086/664627

[B85] Naka L. N. Brumfield R. T. (2018). The dual role of Amazonian rivers in the generation and maintenance of avian diversity. *Sci. Adv.* 4 eaar8575. 10.1126/sciadv.aar8575 30083603 PMC6070317

[B86] Naka L. N. Laranjeiras T. O. Lima G. R. Plaskievicz A. C. Mariz D. Costa B. M. (2020). The avifauna of the Rio Branco, an Amazonian evolutionary and ecological hotspot in peril. *Bird Conserv. Int.* 30 21–39. 10.1017/s0959270919000133

[B87] Nazareno A. G. Bemmels J. B. Dick C. W. Lohmann L. G. (2017b). Minimum sample sizes for population genomics: an empirical study from an Amazonian plant species. *Mol. Ecol. Res.* 17 1136–1147. 10.1111/1755-0998.12654 28078808

[B88] Nazareno A. G. Carvalho D. (2009). What the reasons for no inbreeding and high genetic diversity of the Neotropical fig tree *Ficus arpazusa*? *Conserv. Genet.* 10 1789–1793. 10.1007/s10592-008-9776-x

[B89] Nazareno A. G. Dick C. W. Lohmann L. G. (2017a). Wide but not impermeable: testing the riverine barrier hypothesis for an Amazonian plant species. *Mol. Ecol.* 26 3636–3648. 10.1111/mec.14142 28393442

[B90] Nazareno A. G. Dick C. W. Lohmann L. G. (2019a). Tangled banks: a landscape genomic evaluation of Wallace’s riverine barrier hypothesis for three amazon plant species. *Mol. Ecol.* 5 980–997. 10.1111/mec.14948 30450714

[B91] Nazareno A. G. Dick C. W. Lohmann L. G. (2019b). A biogeographic barrier test reveals a strong genetic structure for a canopy-emergent Amazon tree species. *Sci. Rep.* 9 18602. 10.1038/s41598-019-55147-1 PMC6901565 31819128

[B92] Nei M. (1977). F-statistics and analysis of gene diversity in subdivided populations. *Ann. Hum. Genet.* 41 225–233. 10.1111/j.1469-1809.1977.tb01918.x 596830

[B93] Nei M. Roychoudhury A. K. (1974). Sampling variances of heterozygosity and genetic distance. *Genetics* 76 379–390. 10.1093/genetics/76.2.379 4822472 PMC1213072

[B94] Nei M. Tajima F. Tateno Y. (1983). Accuracy of estimated phylogenetic trees from molecular data. *J. Mol. Evol.* 19 153–170. 10.1007/bf02300753 6571220

[B95] Nunes S. Barlow J. Gardner T. Sales M. Monteiro D. Souza C. Jr. (2019). Uncertainties in assessing the extent and legal compliance status of riparian forests in the eastern Brazilian Amazon. *Land Use Policy* 82 37–47. 10.1016/j.landusepol.2018.11.051

[B96] Nybom H. (2004). Comparison of different nuclear DNA markers for estimating intraspecific genetic diversity in plants. *Mol. Ecol.* 13 1143–1155. 10.1111/j.1365-294x.2004.02141.x 15078452

[B97] Nybom H. Bartish I. V. (2000). Effects of life history traits and sampling strategies on genetic diversity estimates obtained with RAPD markers in plants. *Perspect. Plant Ecol. Evol. Syst.* 3 93–114. 10.1078/1433-8319-00006

[B98] Papadopoulou A. Knowles L. L. (2016). Towards a paradigm shift in comparative phylogeography driven by trait-based hypotheses. *Proc. Natl. Acad. Sci. U.S.A.* 113 8018–8024. 10.1073/pnas.1601069113 27432974 PMC4961141

[B99] Parolin P. Wittmann F. Ferreira L. V. (2013). Fruit and seed dispersal in Amazonian floodplain trees–a review. *Ecotropica* 19 15–32.

[B100] Pennington R. T. Dick C. W. (2010). “Diversification of the Amazonian flora and its relation to key geological and environmental events: a molecular perspective,” in *Amazonia, Landscape and Species Evolution: A Look into the Past*, eds Hoorn C. Vonhof H. Wesselingh F. (Hoboken, NJ: Wiley-Blackwell), 373–385. 10.1002/9781444306408.ch23

[B101] Peterson B. K. Weber J. N. Kay E. H. Fisher H. S. Hoekstra H. E. (2012). Double digest RADseq: an inexpensive method for de novo SNP discovery and genotyping in model and non-model species. *PLoS One* 7:e37135. 10.1371/journal.pone.0037135 22675423 PMC3365034

[B102] Prentis P. J. Vesey A. Meyers N. M. Mather P. B. (2004). Genetic structuring of the stream lily *Helmholtzia glaberrima* (Philydraceae) within Toolona Creek, south-eastern Queensland. *Aust. J. Bot.* 52 201–207. 10.1071/bt03064

[B103] Prots B. Omelchuk O. Van Bodegom P. V. (2011). The role of river corridors for plants dispersal. *J. Biol. Syst.* 3 150–154.

[B104] R Core Team (2018). *R: A Language and Environment for Statistical Computing.* Vienna: R Foundation for Statistical Computing.

[B105] Ribas C. C. Aleixo A. Nogueira A. C. R. Miyaki C. Y. Cracraft J. (2012). A palaeobiogeographic model for biotic diversification within Amazonia over the past three million years. *Proc. R. Soc. London B Biol. Sci.* 279 681–689. 10.1098/rspb.2011.1120 21795268 PMC3248724

[B106] Rice W. R. (1989). Analyzing tables of statistical tests. *Evolution* 43 223–225. 10.2307/2409177 28568501

[B107] Richards C. M. Church S. McCauley D. E. (1999). The influence of population size and isolation on gene flow by pollen in *Silene alba.* *Evolution* 53, 63–73. 10.1111/j.1558-5646.1999.tb05333.x 28565199

[B108] Ritland K. (1989). Genetic differentiation, diversity, and inbreeding in the mountain monkeyflower (Mimulus caespitosus) of the Washington Cascades. *Canadian J. Bot.* 67 2017–2024. 10.1139/b89-255

[B109] Rochette N. C. Rivera-Colón A. G. Catchen J. M. (2019). Stacks 2: analytical methods for paired-end sequencing improve RADseq-based population genomics. *Mol. Ecol.* 28 4737–4754. 10.1111/mec.15253 31550391

[B110] Rodríguez-Ezpeleta N. Bradbury I. R. Mendibil I. Álvarez P. Cotano U. Irigoien X. (2016). Population structure of Atlantic mackerel inferred from RAD-seq-derived SNP markers: effects of sequence clustering parameters and hierarchical SNP selection. *Mol. Ecol. Res.* 16 991–1001. 10.1111/1755-0998.12518 26936210

[B111] Rogalski J. M. Reis A. Rogalski M. Montagna T. Reis M. S. (2017). Mating system and genetic structure across all known populations of *Dyckia brevifolia*: a clonal, endemic, and endangered rheophyte bromeliad. *J. Heredity* 108 299–307. 10.1093/jhered/esx011 28199659

[B112] Rousset F. (1997). Genetic differentiation and estimation of gene flow from F-statistics under isolation by distance. *Genetics* 145 1219–1228. 9093870 10.1093/genetics/145.4.1219 PMC1207888

[B113] Stamatakis A. (2014). RAxML version 8: a tool for phylogenetic analysis and post-analysis of large phylogenies. *Bioinformatics* 30 1312–1313. 10.1093/bioinformatics/btu033 24451623 PMC3998144

[B114] Taylor C. M. (2007). “Psychotria L,” in *Flora Fanerogâmica do Estado de São Paulo*, eds Wanderley M. G. L. Shepherd G. J. Melhem T. S. Giulietti A. M. (São Paulo: FAPESP), 389–412.

[B115] Tero N. Aspi J. Siikamäki P. Jäkäläniemi A. Tuomi J. (2003). Genetic structure and gene flow in a metapopulation of an endangered plant species, *Silene tatarica*. *Mol. Ecol.* 12 2073–2085. 10.1046/j.1365-294x.2003.01898.x 12859630

[B116] Thiel-Egenter C. Gugerli F. Alvarez N. Brodbeck S. Cieslak E. Colli L. (2008). Effects of species traits on the genetic diversity of high-mountain plants: a multi-species study across the Alps and Carpathians. *Glob. Ecol. Biog.* 18 78–87. 10.1111/j.1466-8238.2008.00421.x

[B117] Trucchi E. Facon B. Gratton P. Mori E. Stenseth N. C. Jentoft S. (2016). Long live the alien: Is high genetic diversity a pivotal aspect of crested porcupine (*Hystrix cristala*) long-lasting and successful invasion? *Mol. Ecol.* 25 3527–3539. 10.1111/mec.13698 27171527

[B118] Ulmer T. MacDougal J. M. (2004). *Passiflora: Passionflowers of the World.* Portland: Timber Press, 1–430.

[B119] Valencia S. B. C. (2002). *Trophic Ecology of Frugivorous Fishes in Floodplain Forests of the Colombian Amazon.* College Station, TX: AandM University, 1–154.

[B120] Van Dyke F. Lamb R. L. (2020). “The conservation of populations: theory, analysis, application,” in *Conservation Biology*, (Cham: Springer).

[B121] Vieira F. A. Fajardo C. G. Souza A. M. Carvalho D. (2010). Landscape-level and fine-scale genetic structure of the Neotropical tree *Protium spruceanum* (Burseraceae). *Int. J. Forestry Res.* 2010 120979.

[B122] Wallace A. R. (1854). On the monkeys of the Amazon. *Ann. Mag. Nat. Hist.* 14 451–454. 10.1080/037454809494374

[B123] Waters J. M. Emerson B. C. Arribas P. McCulloch G. A. (2020). Dispersal reduction: causes, genomic mechanisms, and evolutionary consequences. *Trends Ecol. Evol.* 35 512–522. 10.1016/j.tree.2020.01.012 32396818

[B124] Wei X. Meng H. Bao D. Jiang M. (2015). Gene flow and genetic structure of a mountain riparian tree species, Euptelea pleiospermum (Eupteleaceae): how important is the stream dendritic network? *Tree Genet. Gen.* 11 64.

[B125] Weir B. S. Cockerham C. C. (1984). Estimating F-statistics for the analysis of population structure. *Evolution* 38 1358–1370. 28563791 10.1111/j.1558-5646.1984.tb05657.x

[B126] Willing E.-M. Dreyer C. van Oosterhout C. (2012). Estimates of genetic differentiation measured by FST do not necessarily require large sample sizes when using many SNP markers. *PLoS One* 7:e42649. 10.1371/journal.pone.0042649 22905157 PMC3419229

[B127] Willson M. F. Traveset A. (2000). “The ecology of seed dispersal,” in *Seeds: The Ecology of Regeneration in Plant Communities*, ed. Fenner M. (New York, NY: CAB), 85–111.

[B128] Wilson G. A. Rannala B. (2003). Bayesian inference of recent migration rates using multilocus genotypes. *Genetics* 163 1177–1191. 12663554 10.1093/genetics/163.3.1177 PMC1462502

[B129] Worbes M. (1997). “The forest ecosystem of the floodplains,” in *The Central Amazon floodplain*, ed. Junk W. J. (Berlin: Springer), 223–265.

[B130] Wright S. (1943). Isolation by distance. *Genetics* 28 114–138. 17247074 10.1093/genetics/28.2.114 PMC1209196

[B131] Wright S. (1951). The genetical structure of populations. *Ann. Eugenics* 15 323–354. 10.1111/j.1469-1809.1949.tb02451.x 24540312

[B132] Wright S. (1965). The interpretation of population structure by F-statistics with special regards to systems of mating. *Evolution* 19 395–420.

[B133] Zellmer A. J. Hanes M. M. Hird S. M. Carstens B. C. (2012). Deep phylogeographic structure and environmental differentiation in the carnivorous plant *Sarracenia alata*. *Syst. Biol.* 61 763–777. 22556200 10.1093/sysbio/sys048

[B134] Zuntini A. R. (2014). *Taxonomic Recision and Phylogeny of Bignonia L. (Bignonieae, Bignoniaceae.* São Paulo: Universidade de São Paulo, 1–309.

