# Supplementary material for: By Animal, Water, or Wind: Can Dispersal Mode Predict Genetic Connectivity in Riverine Plant Species?
Source: Front Plant Sci. 2021 Feb 12;12:626405. doi: 10.3389/fpls.2021.626405 (PMC7907645; doi:10.3389/fpls.2021.626405)
Supplement: Supplementary file 1 [file Table_1.DOCX]

**BY ANIMAL, WATER, OR WIND: CAN DISPERSAL MODE PREDICT GENETIC CONNECTIVITY IN RIVERINE PLANT SPECIES?**

**Supporting Information**

Alison G. Nazareno^1,2,3^, LAURA LACEY KNOWLES^3^, Christopher W. Dick^3,4^, Lúcia G. Lohmann^1,^

^1^ Departmento de Botânica, Universidade de São Paulo, São Paulo, SP, Brazil.

^2^ Department of Genetics, Ecology and Evolution, Federal University of Minas Gerais, Belo Horizonte, MG, Brazil.

^3^ Department of Ecology and Evolutionary Biology, University of Michigan, Ann Arbor, MI, USA.

^4^ Smithsonian Tropical Research Institute, Panama City, Republic of Panama

E-mail: [alison_nazareno@yahoo.com.br](mailto:alison_nazareno@yahoo.com.br) (AGN), [llohmann@usp.br](mailto:llohmann@usp.br) (LGL)

**APPENDIX S1** *Genomic library and sequencing*

Five genomic libraries were created using a double digest RADseq protocol (Peterson *et al*. 2012), with the modifications proposed by Nazareno *et al*. (2017b) to minimize variance in the number of reads per individual within each pool. Double-stranded DNA concentrations were quantified before digestion reactions using the Qubit dsDNA Assay Kit (Invitrogen). Samples were adjusted to equal molar concentration and the final DNA concentration varied from 200 ng.µL^-1^ (*Bignonia aequinoctialis*) to 450 ng.µL^-1^ (*Anemopaegma paraense*). Each sample was digested with two high-fidelity restriction enzymes EcoRI and MseI (New England Biolabs). Digestion reactions were carried out in a total volume of 20 µL, using 17 µL of resuspended DNA, 5 units of EcoRI, 5 units of MseI and 1X CutSmart buffer (New England Biolabs) for 3 hours at 37 ºC, ending with a 20 min deactivation step at 65 ºC. Reactions were then purified with the Agencourt AMPure XP system (Beckman Coulter), following the manufacturer’s instructions, with elution in 40 µL TE buffer. In order to standardize the initial DNA mass to be added into an adapter ligation, the cleaned digests were quantified using Qubit. Adapter ligations were carried out in a total volume of 30 µL, combining 42 ng DNA, 0.22 µM of a non-sample specific MseI adaptor (common for all samples), 0.33 µM of a sample specific EcoRI double-strand adaptor for each DNA sample, 1U of T4 DNA ligase (New England BioLabs), and 1.3 X T4 ligase buffer which were incubated at 23 ºC for 30 min. Reactions were then heat-killed at 65 ºC for 10 min following a slow cooling to room temperature (23 ºC). A total of 96 EcoRI double-stranded barcodes with a unique 10 base pair sequence were created using Python; for further details on the barcodes and the MseI oligos sequences see Nazareno *et al*. (2017b).

After cleaning the reactions with the Agencourt AMPure XP system, ligation products were amplified in 20 µL PCRs, each containing 13.5 µL of the ligation product, 0.2 µM of Illumina PCR primers, 0.2 mM dNTPs, 1.0 mM MgCl_2_, 0.5 U of iProof^TM^ High-Fidelity DNA polymerase (BIO-RAD), and 2X of iProof buffer. The PCR protocol (98°C for 30s, 20 cycles of 98 °C for 20s, 60 ºC for 30s and 72 °C for 40s, followed by a final extension at 72 °C for 10 min) was carried out in an Eppendorf PCR System. Before pooling samples at each library, each sample was purified using the Agencourt AMPure XP system and the DNA was quantified using Qubit. Multiplexed libraries were prepared with approximately equal amounts of DNA among samples. Automated size-selection was performed using a 2% agarose cartridge (Pippin Prep; Sage Science, Beverly, MA) to select genomic fragments at a target range size of 375-475 bp. Size, quantity, and quality of each individual library were measured on the Agilent 2100 Bioanalyzer (Agilent Technologies) using the Agilent DNA 1000 Kit. Each library was sequenced (100-bp single-end reads) in a single lane of an Illumina HiSeq 2500 flow cell (Illumina Inc., San Diego, CA) at The Centre for Applied Genomics in Toronto, Canada (each lane was pooled with 64-84 samples).

**APPENDIX S2** *Data quality control for abiotically dispersed plant species*

The number of single-end raw reads of 101 bp produced for each lane of HiSeq 2000 Illumina ranged from 179 million (library with 84 *Tanaecium pyramidatum*) to 426 million (library with 84 *Anemopaegma paraense* samples). Each read starts with a barcode sequence identifying a sample (up to 10 bp long) and the 6 bp restriction site, followed by 85 bp of usable data. The mean number of retained reads that passed the default quality filters, including a Phred quality score > 33, contained an identifiable barcode as follows: 3,502,045 ± 169,327 SE for *Adenocalymma schomburgkii*, 4,874,419 ± 223,086 SE for *Anemopaegma paraense*, 2,820,519 ± 141,773 SE for *Bignonia aequinoctialis*, 2,561,979 ± 133,424 SE for *Pachyptera kerere*, and 2,082,463 ± 55,676 SE for *Tanaecium pyramidatum*.

For each abiotically dispersed plant species sampled, further filtering (i.e., 10-fold coverage; presence in at least 85% of the individuals; MAF > 0.01) resulted in 36,792 (mean coverage depth = 23.4 ± 9.5 SD) unlinked polymorphic SNP markers within the RAD tag sequences for all sampling locations for *Adenocalymma schomburgkii*, 39,796 (mean coverage depth = 17.0 ± 7.8 SD) for *Anemopaegma paraense*, 10,618 (mean coverage depth = 12.6 ± 4.3 SD) for *Bignonia aequinoctialis*, 34,348 (mean coverage depth = 19.3 ± 8.6 SD) for *Pachyptera kerere*, and 28,142 (mean coverage depth = 18.6 ± 4.1 SD) for *Tanaecium pyramidatum*. After a Bonferroni adjustment, no significant departures from H-W equilibrium were observed in any sampling location or species (*p* > 1.36 × 10^-6^ for *Adenocalymma schomburgkii*, *p* > 1.26 × 10^-6^ for *Anemopaegma paraense*, *p* > 4.71 × 10^-6^ for *Bignonia aequinoctialis*, *p* > 1.46 × 10^-6^ for *Pachyptera kerere*, and *p* > 1.78 × 10^-6^ for *Tanaecium pyramidatum*). In addition, considering each species, no linkage disequilibrium was observed after a sequential Bonferroni correction for k tests (k = 6.76 × 10*^8^*, *p* < 7.39 × 10^-11^ for *Adenocalymma schomburgkii*, k = 7.91 × 10*^8^*, *p* < 6.31 × 10^-11^ for *Anemopaegma paraense*, k = 5.63 × 10*^7^*, *p* < 8.87 × 10^-10^ for *Bignonia aequinoctialis*, k = 5.89 × 10*^8^*, *p* < 8.47 × 10^-11^ for *Pachyptera kerere*, and k = 3.95 × 10*^8^*, *p* < 1.26 × 10^-10^).

We detected potential loci that were under diversifying selection with the false discovery rate (FDR) set to 0.05: 24 loci for *Adenocalymma schomburgkii*, 49 loci for *Anemopaegma paraense*, 23 loci for *Bignonia aequinoctialis*, 83 loci for *Pachyptera kerere*, and 21 loci for *Tanaecium pyramidatum* (Fig. S1). Thus, the total of filtered SNPs used in the genomic analyses were 36,768 for *Adenocalymma schomburgkii*, 39,747 for *Anemopaegma paraense*, 10,595 for *Bignonia aequinoctialis*, 34,265 for *Pachyptera kerere*, and 28,121 for *Tanaecium pyramidatum*. Considering the final dataset, minor allele frequency (MAF) averaged 0.0823 ± 0.1055 SD for *Adenocalymma schomburgkii*, 0.1251 ± 0.1388 SD for *Anemopaegma paraense*, 0.0888 ± 0.0998 SD for *Bignonia aequinoctialis*, 0.1106 ± 0.1293 SD for *Pachyptera kerere*, and 0.0776 ± 0.1011 SD for *Tanaecium pyramidatum*. Although the ploidy levels for the studied plant species are unknown, our SNPs analyses showed a pattern compatible with diploid since all individuals of *Adenocalymma schomburgkii, Anemopaegma paraense*, *Bignonia aequinoctialis*, *Pachyptera kerere*, and *Tanaecium pyramidatum* displayed just two alleles per locus. Results from the HiSeq 2000 Illumina sequencing and data quality control for animal-dispersed plant species (i.e., *Amphirrhox longifolia*, *Passiflora spinosa*, and *Psychotria lupulina*) are reported in Nazareno et al. (2019a).

**REFERENCES**

Nazareno AG, Bemmels JB, Dick CW, Lohmann LG. 2017b. Minimum sample sizes for population genomics: An empirical study from an Amazonian plant species. *Molecular Ecology Resources* **17**, 1136–1147.

Nazareno AG, Dick CW, Lohmann LG. 2019a. Tangled banks: a landscape genomic evaluation of Wallace's riverine barrier hypothesis for three amazon plant species. *Molecular Ecology* **5**: 980–997.

Peterson BK, Weber JN, Kay EH, Fisher HS, Hoekstra HE. 2012. Double digest RADseq: An inexpensive method for *de novo* SNP discovery and genotyping in model and non-model species. *PLoS One* **7**: e37135.

**TABLE S1** Collection information and number of individuals sampled in each location along the right (R) and left (L) banks of the Rio Branco (Amazon Basin, Brazil) for (A) *Adenocalymma schomburgkii*, (B) *Amphirrhox longifolia*, (C) *Anemopaegma paraense*, (D) *Bignonia aequinoctialis*, (E) *Pachyptera kerere*, (F) *Passiflora spinosa*, (G) *Psychotria lupulina*, and (H) *Tanaecium pyramidatum*. The vouchers are deposited at São Paulo University Herbarium (SPF), SP, Brazil.

| Location | Latitude | Longitude | A | B | C | D | E | F | G | H | Vouchers  A | Vouchers  B | Vouchers  C | Vouchers  D | Vouchers  E | Vouchers  F | Vouchers  G | Vouchers  H |
| --- | --- | --- | --- | --- | --- | --- | --- | --- | --- | --- | --- | --- | --- | --- | --- | --- | --- | --- |
| 1R | 01°23 27.1" S | 61°50'31.6" W | 6 | 6 | 6 | 6 | 6 | 6 | 6 | 6 |  | AF131 |  |  |  | EK259 | AF130 |  |
| 2R | 01°14'40.0" S | 61°49'52.0" W | 6 | 6 | 6 | - | 6 | 6 | 6 | 6 |  | AF132 | BMG716 | - |  | EK323 | AF133 |  |
| 3R | 01°05'25.2" S | 61°52'06.5" W | 4 | - | 6 | 6 | 6 | 6 | 6 | 6 |  | - |  |  |  | EK325 | MB339 |  |
| 4R | 00°56'44.1" S | 61°50'58.0" W | 2 | 6 | 6 | 6 | 6 | 6 | 6 | - |  | VT416 |  |  |  | EK280 | AF139 | - |
| 5R | 00°50'55.7" S | 61°51'33.1" W | 4 | 6 | 6 | 6 | 6 | 6 | 6 | 6 |  | VT417 | EYK327 |  |  | EK296 | VT418 |  |
| 6R | 00°43'46.3" S | 61°51'24.0" W | 3 | 6 | 6 | 6 | 6 | 6 | 6 | 6 |  | VT419 |  |  |  | EK298 | VT420 |  |
| 7R | 00°35'07.0" S | 61°48'15.3" W | 4 | 6 | 6 | 6 | 6 | - | 6 | 6 |  | VT425 |  |  |  | - | VT426 |  |
| 7L | 00°35'25.3" S | 61°48'41.4" W | 3 | 6 | 6 | 6 | 6 | 6 | 6 | 6 | AF295 | VT427 | AF293 | AF292 | AF294 | AF296 | VT428 | AF291 |
| 6L | 00°43'34.5" S | 61°52'05.7" W | 6 | 6 | 6 | 6 | 6 | 6 | 6 | 6 | AF287 | VT429 | AF297 | AF286 | AF301 | AF285 | VT430 | AF289 |
| 5L | 00°52'2.05" S | 61°52'51.3" W | 4 | 6 | 6 | 6 | 6 | 6 | 6 | 6 | AF279 | VT432 | AF283 | AF280 | AF284 | AF282 | VT433 | AF281 |
| 4L | 00°56'46.4" S | 61°52'32.1" W | 5 | 6 | 6 | 6 | 6 | 6 | 6 | 6 | AF274 | VT434 | AF290 | AF277 | AF278 | AF300 | VT435 | AF273 |
| 3L | 01°05'46.5" S | 61°52'53.0" W | 6 | 6 | 6 | 6 | 6 | 6 | 6 | 6 | AF266 | VT436 |  | AF268 | AF272 | AF269 | VT437 | AF270 |
| 2L | 01°14'42.7" S | 61°50'56.2" W | 5 | 6 | 6 | 6 | 6 | 6 | 6 | 6 |  | VT442 |  |  |  | EK273 | VT443 |  |
| 1L | 01°22'24.5" S | 61°51'59.7" W | 6 | 6 | 6 | 6 | 6 | 6 | - | 6 | AF256 | VT447 | AF302 | AF303 | AF255 | AF304 | - | AF259 |

**TABLE S2** Genetic diversity statistics estimated from ddRADseq data for eight plant species from locations sampled along the Rio Branco (Amazon Basin, Brazil); mean observed heterozygosity (*H*_O_), mean unbiased expected genetic diversity (*uH*_E_), and inbreeding coefficient (*F*_IS_). Values in parentheses are 95% confidence intervals.

| **Plant species** | ***H*_O_** | ***uH*_E_** | ***F*_IS_** |
| --- | --- | --- | --- |
| 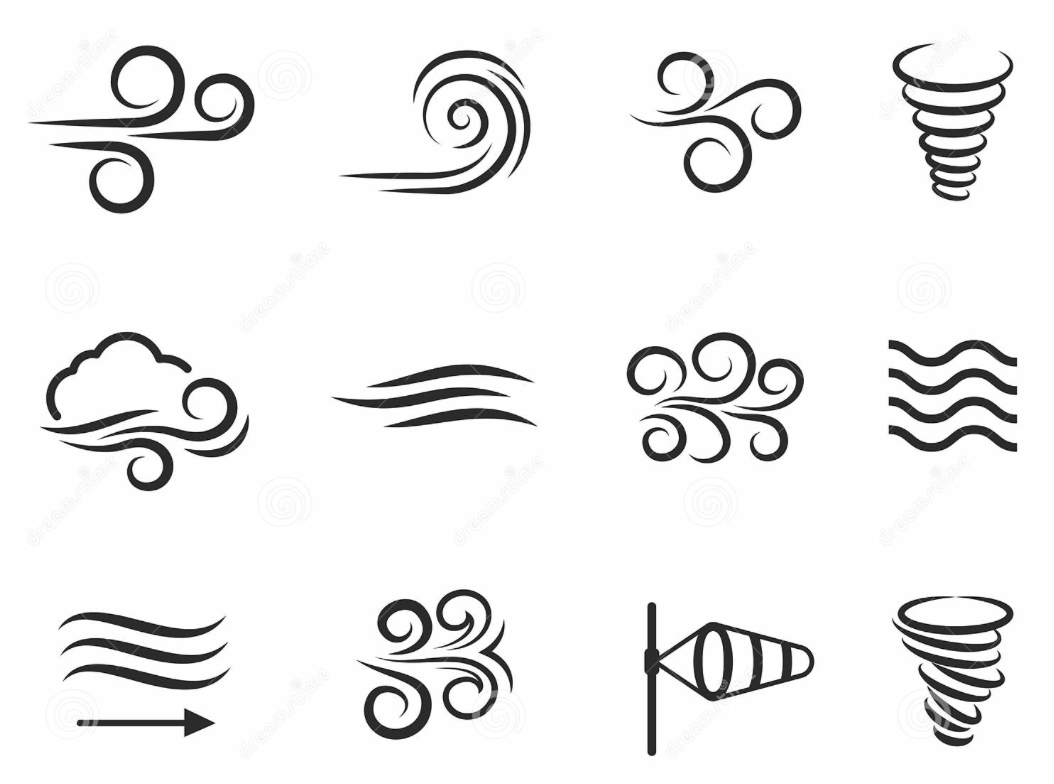  *Adenocalymma schomburgkii* | 0.114 ± (0.001) | 0.106 ± (0.008) | 0.025 ± (0.007) |
| 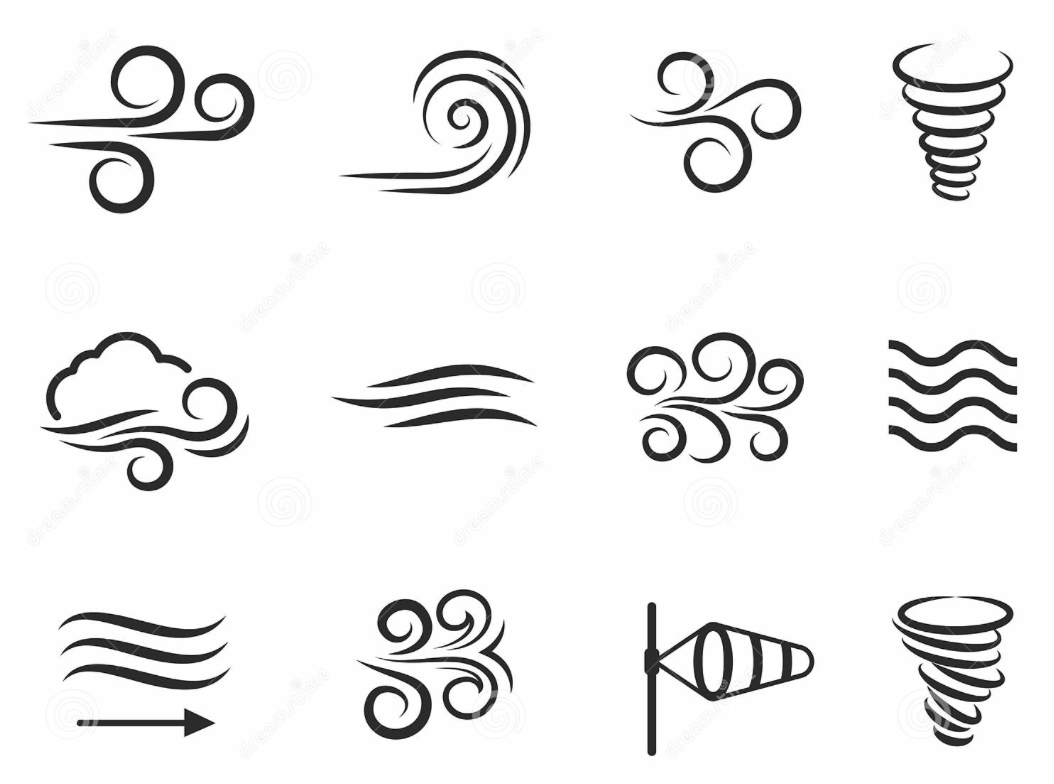*Bignonia aequinoctialis* | 0.113 ± (0.008) | 0.104 ± (0.011) | 0.009 ± (0.022) |
| 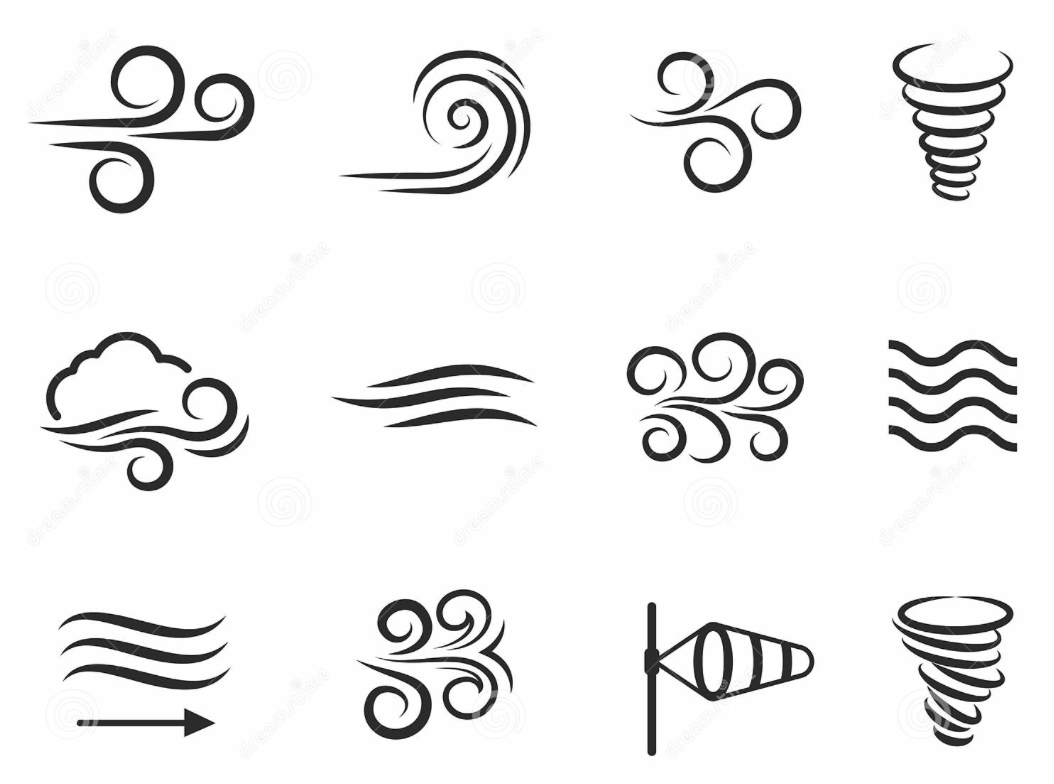*Tanaecium pyramidatum* | 0.115 ± (0.002) | 0.108 ± (0.005) | 0.012 ± (0.007) |
| 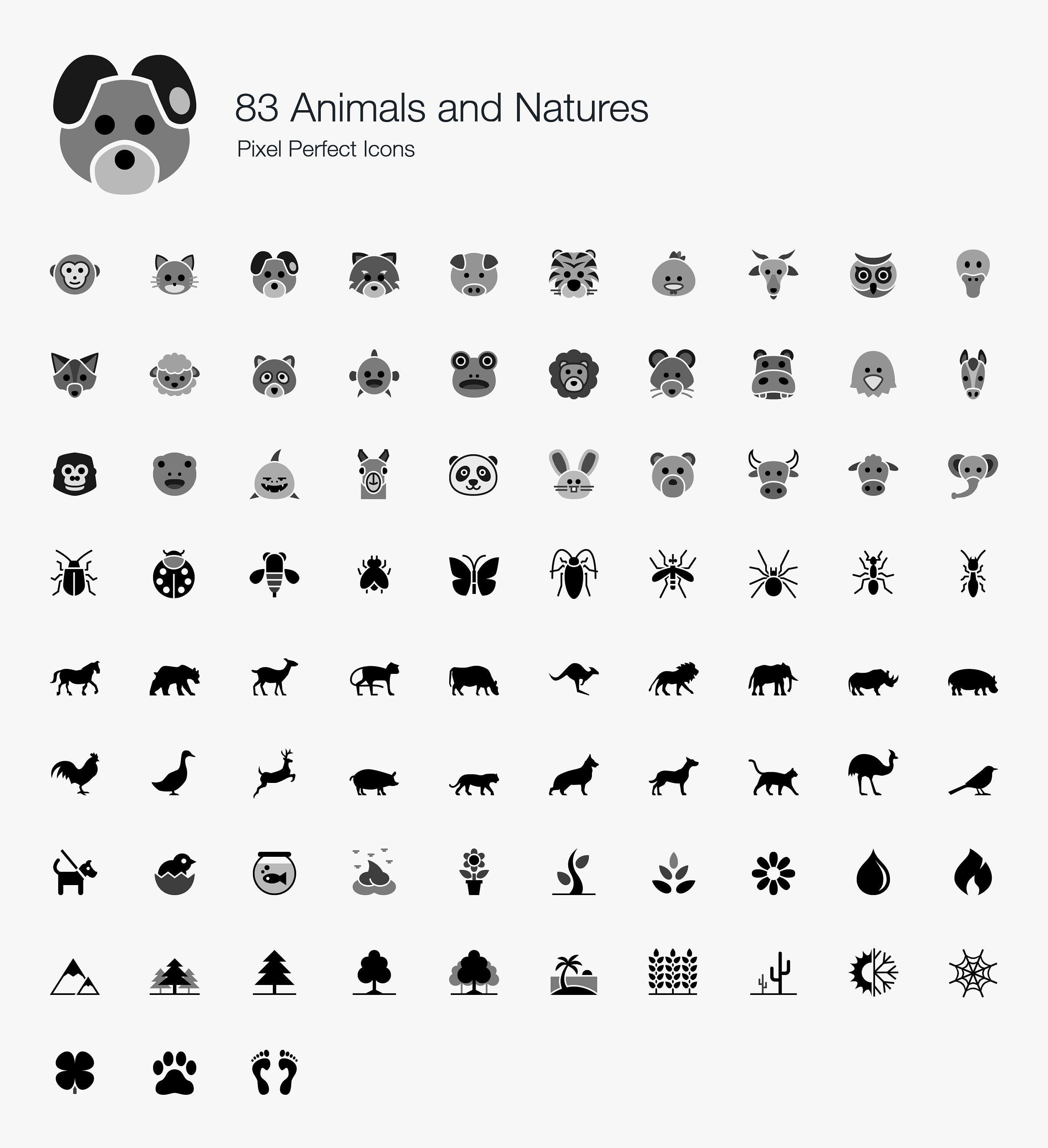  *Anemopaegma paraense* | 0.179 ± (0.005) | 0.161 ± (0.003) | 0.004 ± (0.007) |
| 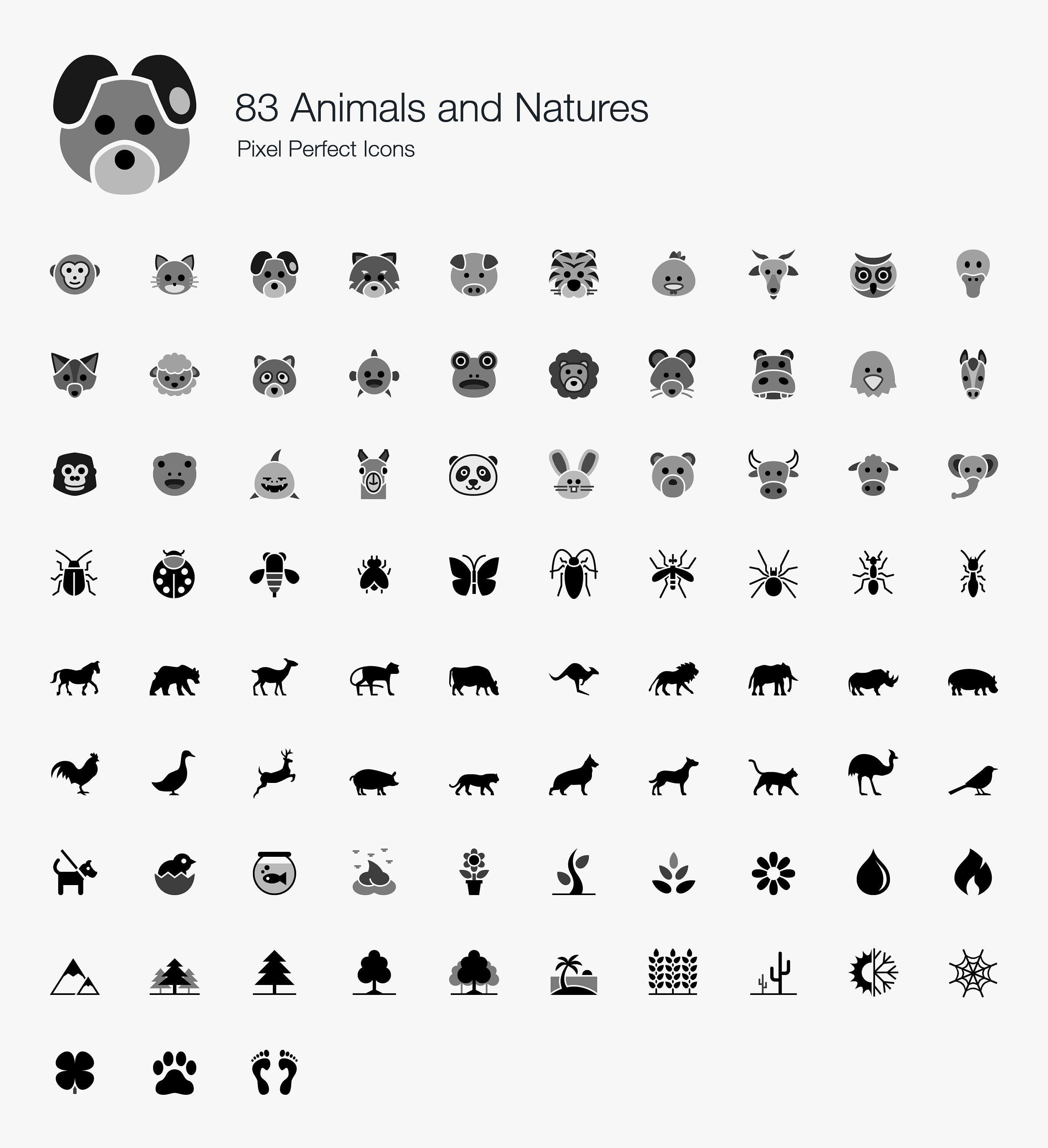  *Pachypetra kerere* | 0.151 ± (0.008) | 0.144 ± (0.003) | 0.023 ± (0.014) |
| *Amphirrhox longifolia* | 0.216 ± (0.016) | 0.205 ± (0.016) | -0.126 ± (0.011) |
| 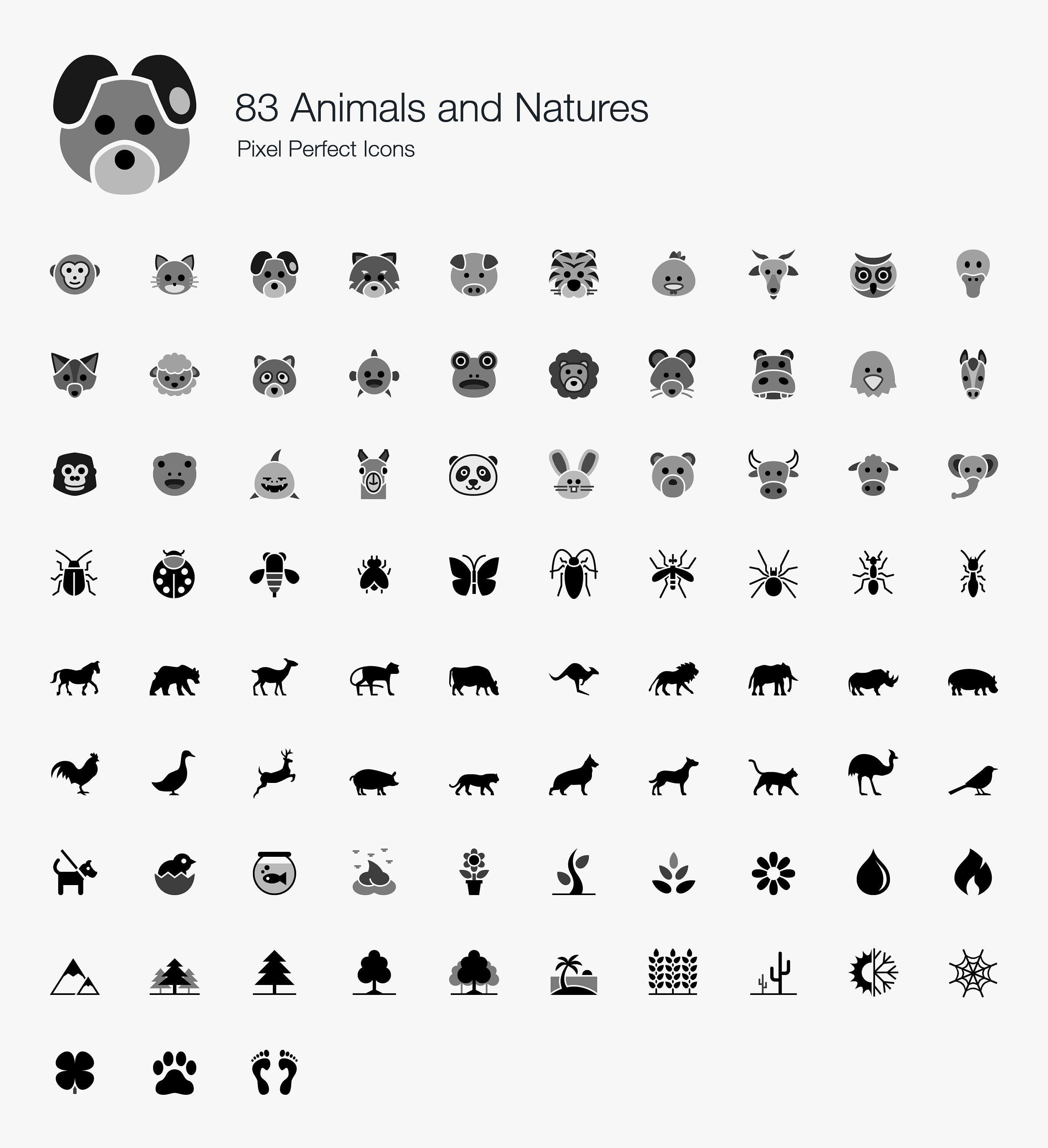*Passiflora spinosa* | 0.392 ± (0.039) | 0.349 ± (0.008) | -0.166 ± (0.021) |
| 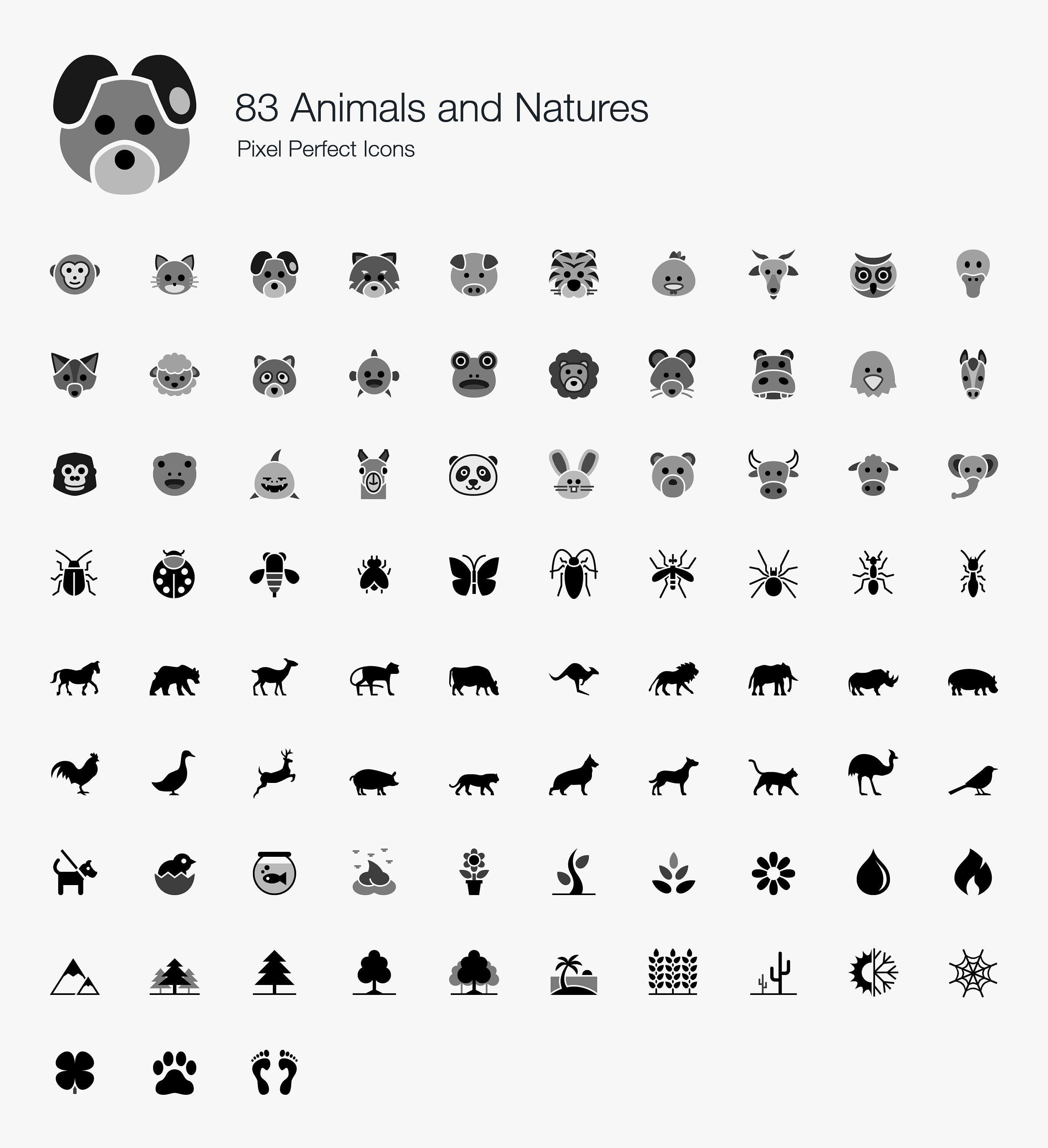  *Psychotria lupulina* | 0.410 ± (0.019) | 0.376 ± (0.012) | -0.190 ± (0.029) |

**TABLE S3** Values correspond to Pearson's correlation coefficient between genetic diversity estimates (*uH*_E_: unbiased expected genetic diversity; *H*_O_: observed heterozygosity) and distance between sampling locations from upstream to downstream along the Rio Branco (Amazon Basin, Brazil).

| **Plant species** | ***uH*_E_** | | ***H*_O_** | |
| --- | --- | --- | --- | --- |
|  | ***r*** | ***P-value*** | ***r*** | ***P-value*** |
| 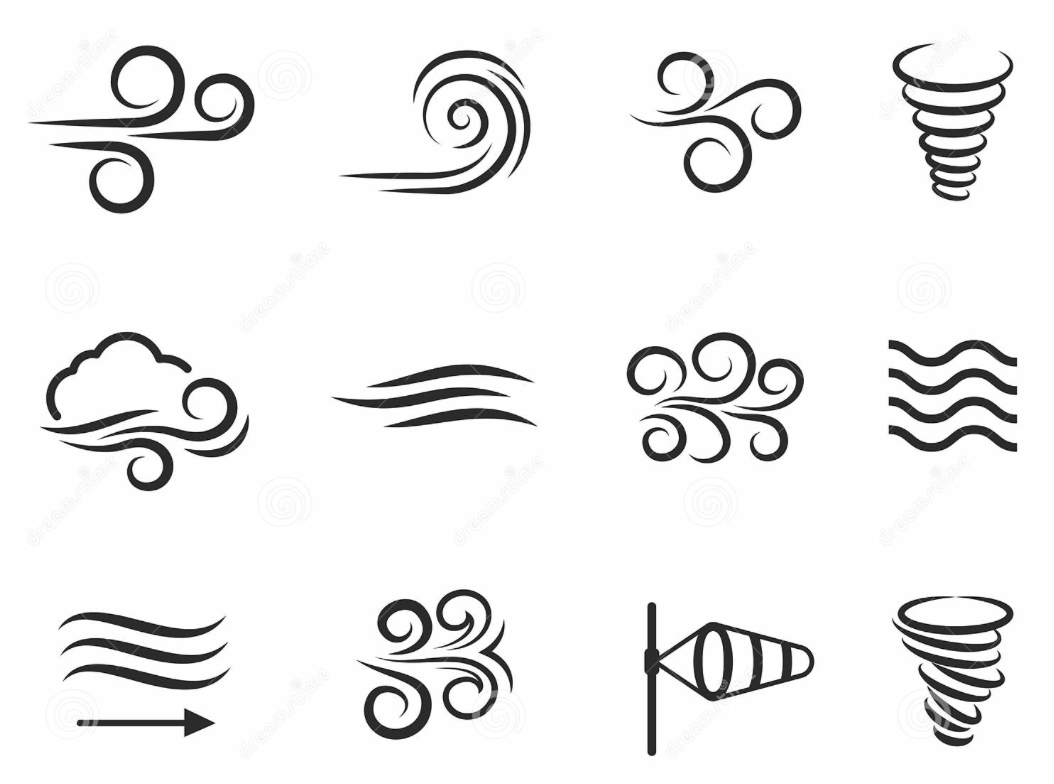  *Adenocalymma schomburgkii* | 0.05 | 0.85 | 0.14 | 0.63 |
| 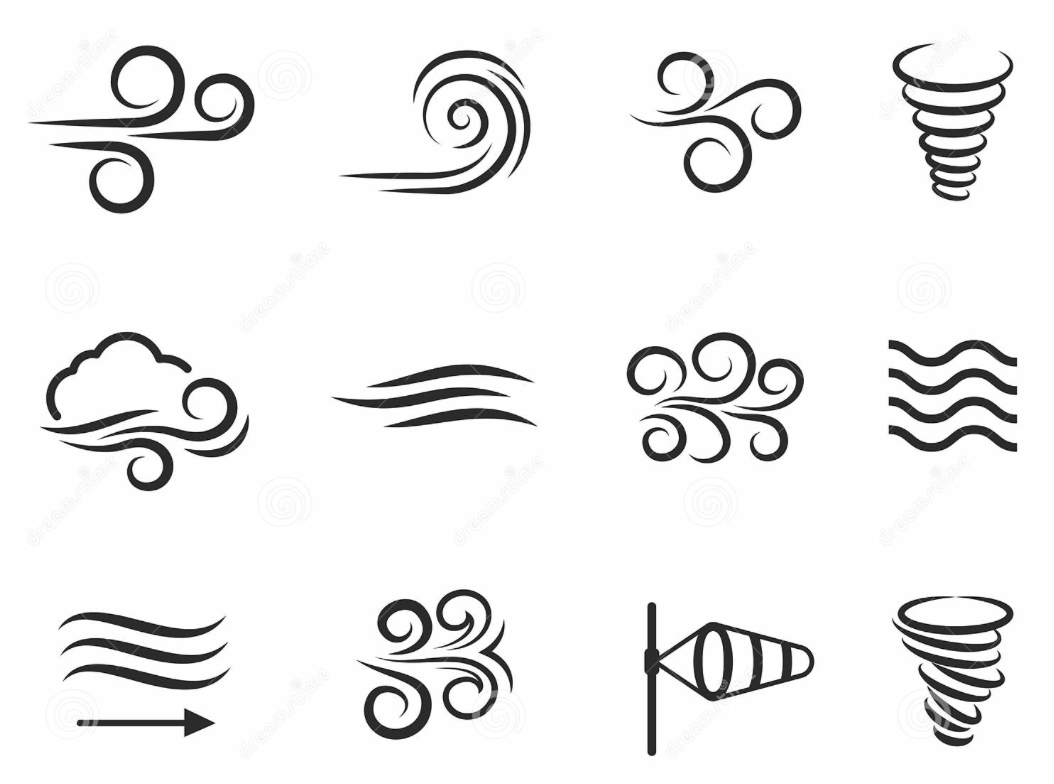*Bignonia aequinoctialis* | 0.17 | 0.56 | 0.04 | 0.89 |
| 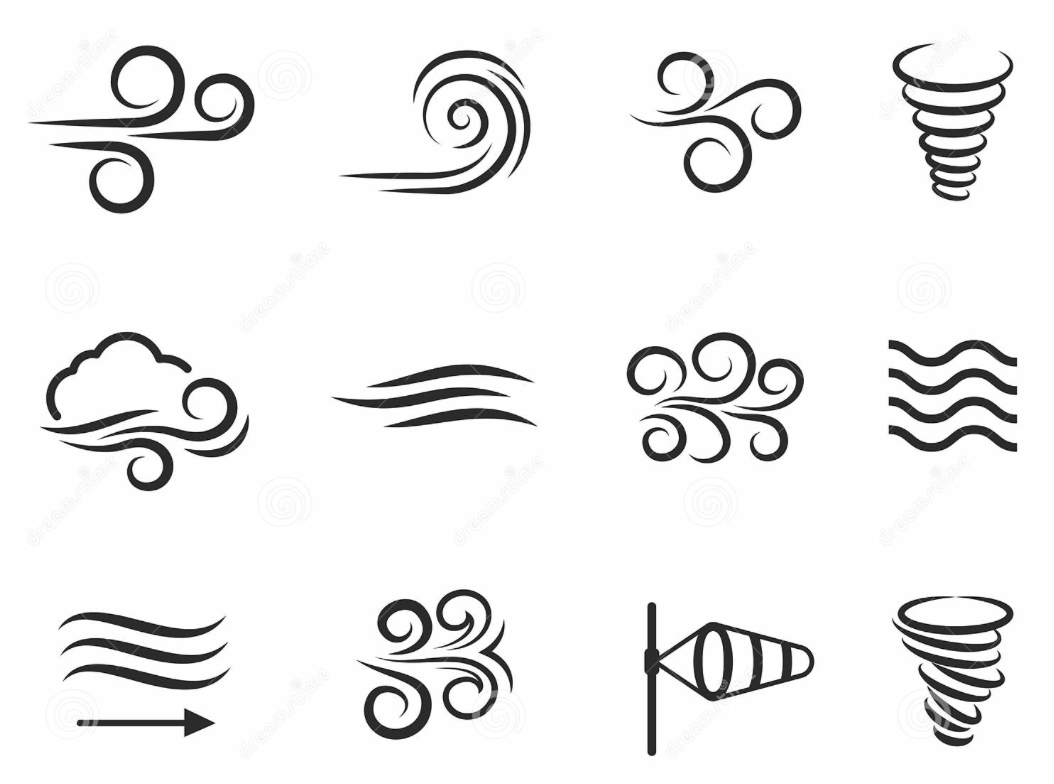*Tanaecium pyramidatum* | 0.29 | 0.32 | -0.21 | 0.46 |
| 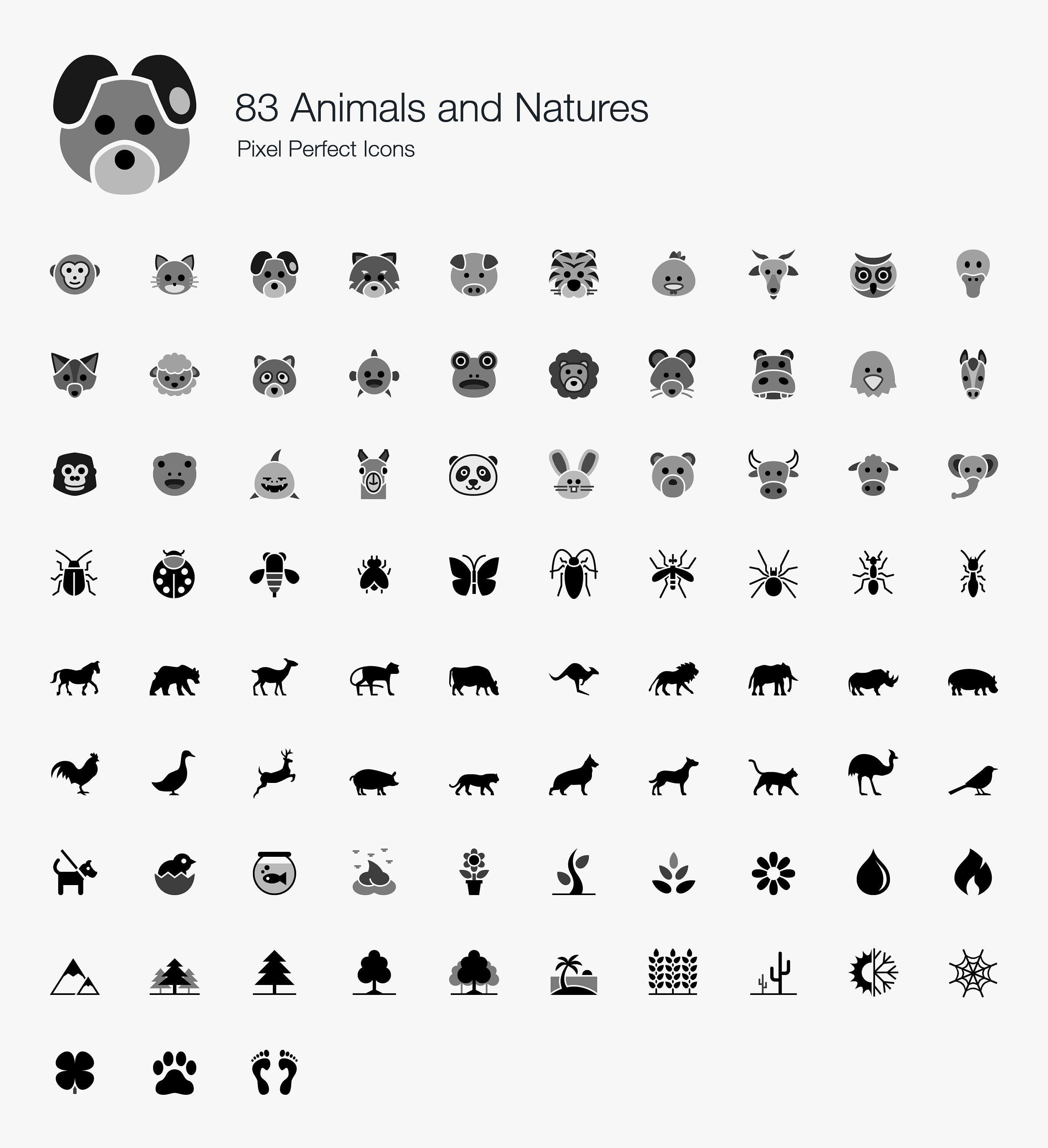  *Anemopaegma paraense* | 0.34 | 0.23 | 0.19 | 0.52 |
| 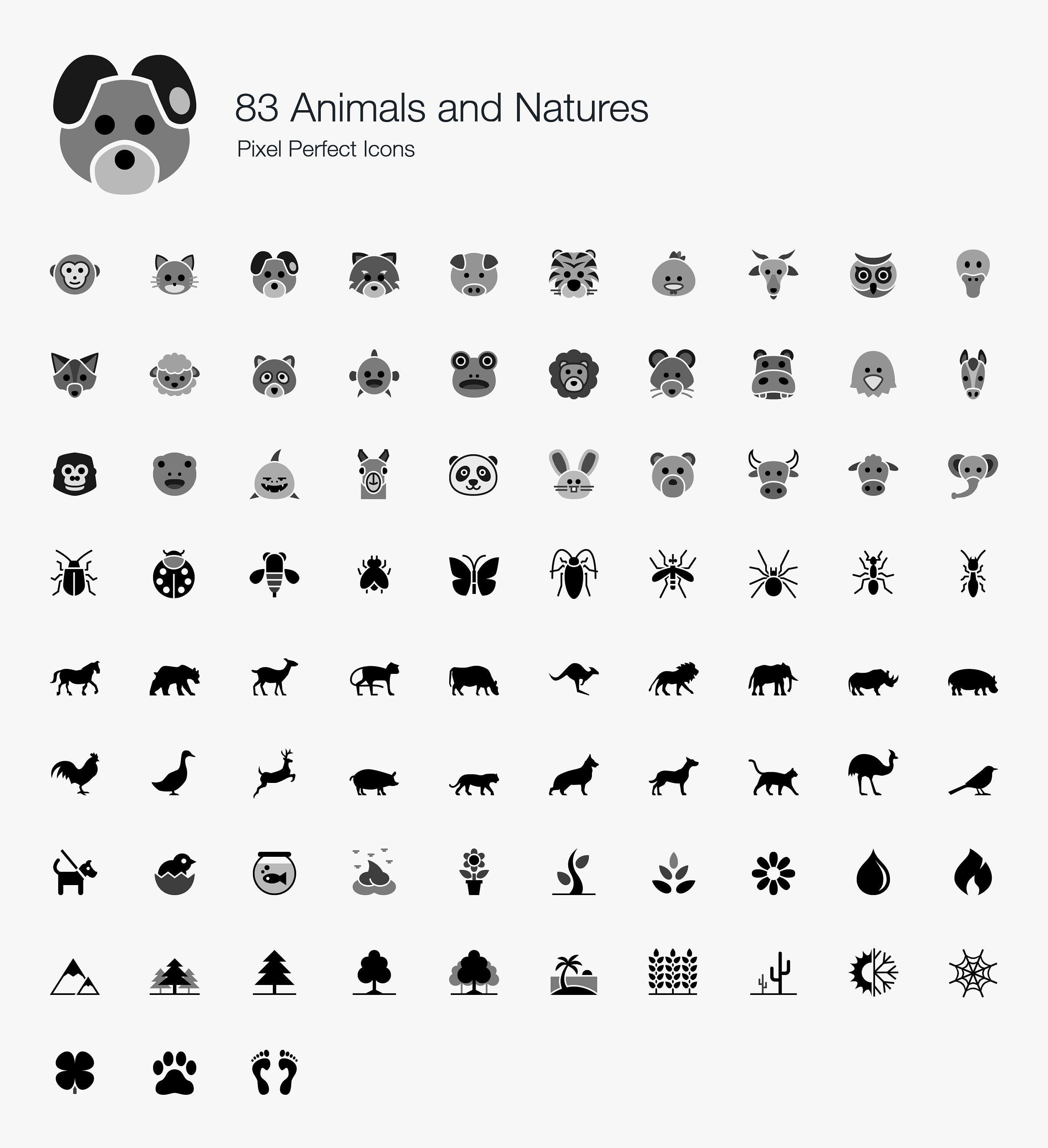  *Pachypetra kerere* | 0.53 | 0.05 | 0.55 | 0.04 |
| *Amphirrhox longifolia* | -0.52 | 0.07 | 0.49 | 0.08 |
| 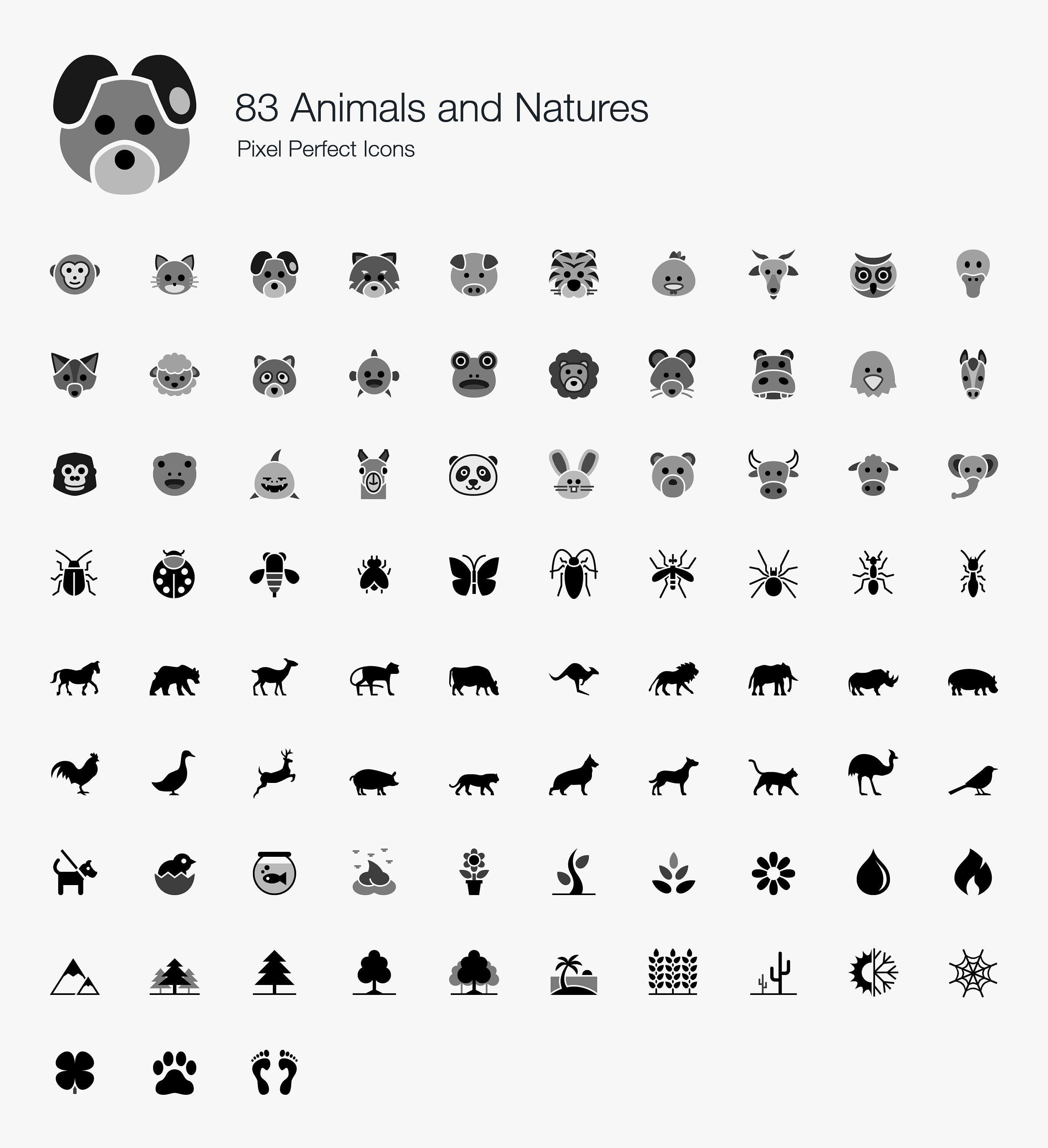*Passiflora spinosa* | 0.27 | 0.37 | 0.29 | 0.33 |
| 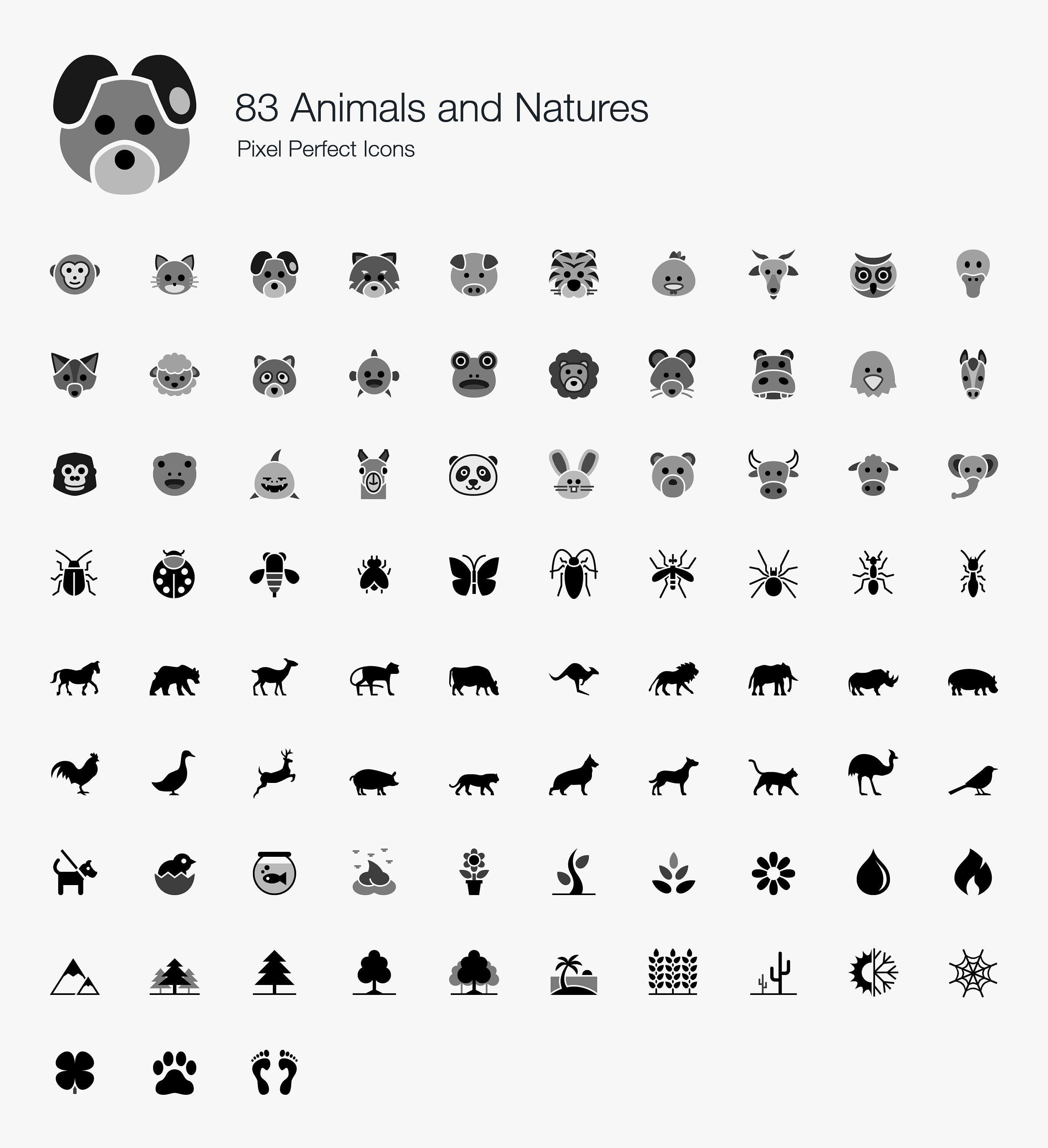  *Psychotria lupulina* | -0.09 | 0.75 | -0.05 | 0.86 |

**TABLE S4** Matrix of the geographic distances (km; above diagonal) and the genetic differentiation (*F*_ST_; below diagonal) between sampling locations along the Rio Branco (Amazon Basin, Brazil) for each species.

(A) *Adenocalymma schomburgkii*

|  | **1R** | **2R** | **3R** | **4R** | **5R** | **6R** | **7R** | **1L** | **2L** | **3L** | **4L** | **5L** | **6L** | **7L** |
| --- | --- | --- | --- | --- | --- | --- | --- | --- | --- | --- | --- | --- | --- | --- |
| **1R** |  | 16.2 | 33.4 | 49.2 | 60.0 | 73.1 | 89.2 | 3.3 | 14.8 | 31.3 | 47.3 | 58.0 | 71.2 | 88.6 |
| **2R** | 0.021 |  | 17.5 | 33.1 | 43.9 | 57.0 | 72.9 | 16.1 | 2.0 | 17.3 | 33.1 | 43.8 | 57.0 | 72.3 |
| **3R** | 0.012 | 0.003 |  | 16.1 | 26.7 | 39.9 | 56.3 | 41.1 | 25.3 | 7.8 | 9.1 | 19.2 | 32.4 | 55.6 |
| **4R** | 0.122 | 0.029 | 0.025 |  | 10.8 | 23.9 | 40.1 | 49.3 | 33.3 | 16.0 | 2.9 | 10.9 | 24.0 | 39.5 |
| **5R** | 0.007 | 0.018 | 0.014 | 0.024 |  | 13.2 | 29.8 | 58.0 | 42.1 | 24.7 | 9.3 | 3.2 | 15.5 | 29.1 |
| **6R** | 0.026 | 0.019 | 0.002 | 0.057 | 0.013 |  | 17.0 | 73.5 | 57.4 | 40.2 | 24.3 | 13.6 | 1.3 | 16.2 |
| **7R** | 0.006 | 0.014 | 0.013 | 0.042 | 0.006 | 0.010 |  | 88.6 | 72.3 | 55.6 | 39.5 | 29.1 | 16.2 | 1.1 |
| **1L** | 0.022 | 0.009 | 0.004 | 0.039 | 0.004 | 0.011 | 0.008 |  | 14.3 | 39.0 | 47.2 | 56.0 | 71.6 | 86.8 |
| **2L** | 0.013 | 0.009 | 0.013 | 0.052 | 0.019 | 0.014 | 0.025 | 0.035 |  | 25.0 | 33.2 | 41.9 | 57.4 | 72.5 |
| **3L** | 0.012 | 0.011 | 0.007 | 0.054 | 0.009 | 0.017 | 0.003 | 0.002 | 0.020 |  | 8.3 | 17.0 | 32.6 | 48.3 |
| **4L** | 0.021 | 0.013 | 0.008 | 0.054 | 0.006 | 0.017 | 0.019 | 0.014 | 0.012 | 0.010 |  | 8.8 | 24.3 | 40.0 |
| **5L** | 0.024 | 0.005 | 0.009 | 0.054 | 0.023 | 0.022 | 0.003 | 0.013 | 0.018 | 0.004 | 0.009 |  | 15.6 | 31.6 |
| **6L** | 0.016 | 0.007 | 0.004 | 0.035 | 0.004 | 0.009 | 0.006 | 0.005 | 0.004 | 0.004 | 0.009 | 0.002 |  | 16.3 |
| **7L** | 0.012 | 0.002 | 0.002 | 0.039 | 0.013 | 0.004 | 0.000 | 0.013 | 0.008 | 0.002 | 0.003 | 0.007 | 0.015 |  |

(B) *Anemopaegma paraense*

|  | **1R** | **2R** | **3R** | **4R** | **5R** | **6R** | **7R** | **1L** | **2L** | **3L** | **4L** | **5L** | **6L** | **7L** |
| --- | --- | --- | --- | --- | --- | --- | --- | --- | --- | --- | --- | --- | --- | --- |
| **1R** |  | 16.2 | 33.4 | 49.2 | 60.0 | 73.1 | 89.2 | 3.3 | 14.8 | 31.3 | 47.3 | 58.0 | 71.2 | 88.6 |
| **2R** | 0.001 |  | 17.5 | 33.1 | 43.9 | 57.0 | 72.9 | 16.1 | 2.0 | 17.3 | 33.1 | 43.8 | 57.0 | 72.3 |
| **3R** | 0.030 | 0.012 |  | 16.1 | 26.7 | 39.9 | 56.3 | 41.1 | 25.3 | 7.8 | 9.1 | 19.2 | 32.4 | 55.6 |
| **4R** | 0.000 | 0.009 | 0.020 |  | 10.8 | 23.9 | 40.1 | 49.3 | 33.3 | 16.0 | 2.9 | 10.9 | 24.0 | 39.5 |
| **5R** | 0.027 | 0.015 | 0.033 | 0.018 |  | 13.2 | 29.8 | 58.0 | 42.1 | 24.7 | 9.3 | 3.2 | 15.5 | 29.1 |
| **6R** | 0.001 | 0.003 | 0.021 | 0.004 | 0.024 |  | 17.0 | 73.5 | 57.4 | 40.2 | 24.3 | 13.6 | 1.3 | 16.2 |
| **7R** | 0.016 | 0.013 | 0.036 | 0.008 | 0.033 | 0.003 |  | 88.6 | 72.3 | 55.6 | 39.5 | 29.1 | 16.2 | 1.1 |
| **1L** | 0.004 | 0.007 | 0.009 | 0.005 | 0.015 | 0.008 | 0.001 |  | 14.3 | 39.0 | 47.2 | 56.0 | 71.6 | 86.8 |
| **2L** | 0.015 | 0.018 | 0.052 | 0.023 | 0.046 | 0.022 | 0.028 | 0.014 |  | 25.0 | 33.2 | 41.9 | 57.4 | 72.5 |
| **3L** | 0.003 | 0.015 | 0.051 | 0.004 | 0.029 | 0.005 | 0.009 | 0.005 | 0.020 |  | 8.3 | 17.0 | 32.6 | 48.3 |
| **4L** | 0.005 | 0.003 | 0.039 | 0.006 | 0.024 | 0.002 | 0.010 | 0.002 | 0.003 | 0.010 |  | 8.8 | 24.3 | 40.0 |
| **5L** | 0.005 | 0.001 | 0.034 | 0.003 | 0.020 | 0.005 | 0.010 | 0.004 | 0.003 | 0.012 | 0.013 |  | 15.6 | 31.6 |
| **6L** | 0.011 | 0.021 | 0.031 | 0.008 | 0.034 | 0.003 | 0.029 | 0.005 | 0.002 | 0.013 | 0.012 | 0.047 |  | 16.3 |
| **7L** | 0.001 | 0.017 | 0.059 | 0.011 | 0.043 | 0.010 | 0.016 | 0.010 | 0.019 | 0.012 | 0.003 | 0.002 | 0.000 |  |

(C) *Bignonia aequinoctialis*

|  | **1R** | **3R** | **4R** | **5R** | **6R** | **7R** | **1L** | **2L** | **3L** | **4L** | **5L** | **6L** | **7L** |
| --- | --- | --- | --- | --- | --- | --- | --- | --- | --- | --- | --- | --- | --- |
| **1R** |  | 33.4 | 49.2 | 60.0 | 73.1 | 89.2 | 3.3 | 14.8 | 31.3 | 47.3 | 58.0 | 71.2 | 88.6 |
| **3R** | 0.073 |  | 16.1 | 26.7 | 39.9 | 56.3 | 41.1 | 25.3 | 7.8 | 9.1 | 19.2 | 32.4 | 55.6 |
| **4R** | 0.062 | 0.104 |  | 10.8 | 23.9 | 40.1 | 49.3 | 33.3 | 16.0 | 2.9 | 10.9 | 24.0 | 39.5 |
| **5R** | 0.060 | 0.074 | 0.079 |  | 13.2 | 29.8 | 58.0 | 42.1 | 24.7 | 9.3 | 3.2 | 15.5 | 29.1 |
| **6R** | 0.058 | 0.085 | 0.066 | 0.071 |  | 17.0 | 73.5 | 57.4 | 40.2 | 24.3 | 13.6 | 1.3 | 16.2 |
| **7R** | 0.049 | 0.067 | 0.065 | 0.054 | 0.059 |  | 88.6 | 72.3 | 55.6 | 39.5 | 29.1 | 16.2 | 1.1 |
| **1L** | 0.072 | 0.095 | 0.078 | 0.085 | 0.085 | 0.075 |  | 14.3 | 39.0 | 47.2 | 56.0 | 71.6 | 86.8 |
| **2L** | 0.065 | 0.089 | 0.076 | 0.084 | 0.072 | 0.071 | 0.092 |  | 25.0 | 33.2 | 41.9 | 57.4 | 72.5 |
| **3L** | 0.071 | 0.078 | 0.085 | 0.093 | 0.077 | 0.079 | 0.092 | 0.065 |  | 8.3 | 17.0 | 32.6 | 48.3 |
| **4L** | 0.056 | 0.080 | 0.055 | 0.068 | 0.062 | 0.055 | 0.086 | 0.072 | 0.074 |  | 8.8 | 24.3 | 40.0 |
| **5L** | 0.087 | 0.078 | 0.108 | 0.087 | 0.092 | 0.081 | 0.085 | 0.067 | 0.081 | 0.089 |  | 15.6 | 31.6 |
| **6L** | 0.084 | 0.087 | 0.098 | 0.105 | 0.087 | 0.094 | 0.104 | 0.081 | 0.079 | 0.089 | 0.092 |  | 16.3 |
| **7L** | 0.068 | 0.099 | 0.074 | 0.083 | 0.074 | 0.070 | 0.093 | 0.077 | 0.077 | 0.077 | 0.090 | 0.089 |  |

(D) *Pachyptera kerere*

|  | **1R** | **2R** | **3R** | **4R** | **5R** | **6R** | **7R** | **1L** | **2L** | **3L** | **4L** | **5L** | **6L** | **7L** |
| --- | --- | --- | --- | --- | --- | --- | --- | --- | --- | --- | --- | --- | --- | --- |
| **1R** |  | 16.2 | 33.4 | 49.2 | 60.0 | 73.1 | 89.2 | 3.3 | 14.8 | 31.3 | 47.3 | 58.0 | 71.2 | 88.6 |
| **2R** | 0.012 |  | 17.5 | 33.1 | 43.9 | 57.0 | 72.9 | 16.1 | 2.0 | 17.3 | 33.1 | 43.8 | 57.0 | 72.3 |
| **3R** | 0.003 | 0.005 |  | 16.1 | 26.7 | 39.9 | 56.3 | 41.1 | 25.3 | 7.8 | 9.1 | 19.2 | 32.4 | 55.6 |
| **4R** | 0.030 | 0.003 | 0.033 |  | 10.8 | 23.9 | 40.1 | 49.3 | 33.3 | 16.0 | 2.9 | 10.9 | 24.0 | 39.5 |
| **5R** | 0.008 | 0.019 | 0.010 | 0.025 |  | 13.2 | 29.8 | 58.0 | 42.1 | 24.7 | 9.3 | 3.2 | 15.5 | 29.1 |
| **6R** | 0.004 | 0.004 | 0.015 | 0.010 | 0.001 |  | 17.0 | 73.5 | 57.4 | 40.2 | 24.3 | 13.6 | 1.3 | 16.2 |
| **7R** | 0.017 | 0.002 | 0.007 | 0.021 | 0.011 | 0.006 |  | 88.6 | 72.3 | 55.6 | 39.5 | 29.1 | 16.2 | 1.1 |
| **1L** | 0.006 | 0.030 | 0.009 | 0.071 | 0.030 | 0.031 | 0.020 |  | 14.3 | 39.0 | 47.2 | 56.0 | 71.6 | 86.8 |
| **2L** | 0.004 | 0.057 | 0.022 | 0.077 | 0.023 | 0.026 | 0.031 | 0.017 |  | 25.0 | 33.2 | 41.9 | 57.4 | 72.5 |
| **3L** | 0.029 | 0.003 | 0.013 | 0.012 | 0.038 | 0.015 | 0.020 | 0.066 | 0.079 |  | 8.3 | 17.0 | 32.6 | 48.3 |
| **4L** | 0.020 | 0.005 | 0.015 | 0.007 | 0.006 | 0.008 | 0.020 | 0.001 | 0.018 | 0.005 |  | 8.8 | 24.3 | 40.0 |
| **5L** | 0.048 | 0.049 | 0.070 | 0.050 | 0.050 | 0.005 | 0.046 | 0.093 | 0.080 | 0.057 | 0.044 |  | 15.6 | 31.6 |
| **6L** | 0.007 | 0.006 | 0.010 | 0.005 | 0.003 | 0.010 | 0.005 | 0.035 | 0.032 | 0.006 | 0.015 | 0.033 |  | 16.3 |
| **7L** | 0.034 | 0.018 | 0.037 | 0.001 | 0.033 | 0.012 | 0.025 | 0.074 | 0.074 | 0.008 | 0.011 | 0.075 | 0.013 |  |

(E) *Tanaecium pyramidatum*

|  | **1R** | **2R** | **3R** | **5R** | **6R** | **7R** | **1L** | **2L** | **3L** | **4L** | **5L** | **6L** | **7L** |
| --- | --- | --- | --- | --- | --- | --- | --- | --- | --- | --- | --- | --- | --- |
| **1R** |  | 16.2 | 33.4 | 60.0 | 73.1 | 89.2 | 3.3 | 14.8 | 31.3 | 47.3 | 58.0 | 71.2 | 88.6 |
| **2R** | 0.053 |  | 17.5 | 43.9 | 57.0 | 72.9 | 16.1 | 2.0 | 17.3 | 33.1 | 43.8 | 57.0 | 72.3 |
| **3R** | 0.044 | 0.043 |  | 26.7 | 39.9 | 56.3 | 41.1 | 25.3 | 7.8 | 9.1 | 19.2 | 32.4 | 55.6 |
| **5R** | 0.039 | 0.043 | 0.032 |  | 13.2 | 29.8 | 58.0 | 42.1 | 24.7 | 9.3 | 3.2 | 15.5 | 29.1 |
| **6R** | 0.032 | 0.027 | 0.019 | 0.004 |  | 17.0 | 73.5 | 57.4 | 40.2 | 24.3 | 13.6 | 1.3 | 16.2 |
| **7R** | 0.039 | 0.037 | 0.027 | 0.016 | 0.006 |  | 88.6 | 72.3 | 55.6 | 39.5 | 29.1 | 16.2 | 1.1 |
| **1L** | 0.049 | 0.021 | 0.041 | 0.038 | 0.013 | 0.030 |  | 14.3 | 39.0 | 47.2 | 56.0 | 71.6 | 86.8 |
| **2L** | 0.031 | 0.037 | 0.024 | 0.024 | 0.002 | 0.021 | 0.030 |  | 25.0 | 33.2 | 41.9 | 57.4 | 72.5 |
| **3L** | 0.059 | 0.061 | 0.047 | 0.045 | 0.029 | 0.039 | 0.055 | 0.046 |  | 8.3 | 17.0 | 32.6 | 48.3 |
| **4L** | 0.038 | 0.042 | 0.032 | 0.011 | 0.001 | 0.013 | 0.032 | 0.019 | 0.036 |  | 8.8 | 24.3 | 40.0 |
| **5L** | 0.039 | 0.040 | 0.030 | 0.010 | 0.022 | 0.011 | 0.028 | 0.017 | 0.042 | 0.004 |  | 15.6 | 31.6 |
| **6L** | 0.053 | 0.055 | 0.046 | 0.028 | 0.003 | 0.020 | 0.045 | 0.035 | 0.056 | 0.022 | 0.025 |  | 16.3 |
| **7L** | 0.068 | 0.060 | 0.050 | 0.044 | 0.015 | 0.025 | 0.052 | 0.045 | 0.062 | 0.033 | 0.038 | 0.030 |  |

(F) *Amphirrhox longifolia**

|  | 1R | 2R | 4R | 5R | 6R | 7R | 7L | 6L | 5L | 4L | 3L | 2L | 1L |
| --- | --- | --- | --- | --- | --- | --- | --- | --- | --- | --- | --- | --- | --- |
| 1R |  | 16.2 | 49.2 | 60.0 | 73.1 | 89.2 | 88.6 | 73.5 | 58.0 | 49.3 | 41.1 | 16.1 | 3.3 |
| 2R | 0.027 |  | 33.1 | 43.9 | 57.0 | 72.9 | 72.3 | 57.4 | 42.1 | 33.3 | 25.3 | 2.0 | 14.8 |
| 4R | 0.029 | 0.013 |  | 10.8 | 23.9 | 40.1 | 39.5 | 24.3 | 9.3 | 2.9 | 9.1 | 33.1 | 47.3 |
| 5R | 0.019 | 0.021 | 0.024 |  | 13.2 | 29.8 | 29.1 | 13.6 | 3.2 | 10.9 | 19.2 | 43.8 | 58.0 |
| 6R | 0.024 | 0.024 | 0.025 | 0.020 |  | 17.0 | 16.2 | 1.3 | 15.5 | 24.0 | 32.4 | 57.0 | 71.2 |
| 7R | 0.025 | 0.016 | 0.021 | 0.014 | 0.013 |  | 1.0 | 17.1 | 32.3 | 40.7 | 49.0 | 73.1 | 87.4 |
| 7L | 0.027 | 0.024 | 0.026 | 0.021 | 0.025 | 0.017 |  | 16.3 | 31.6 | 40.0 | 48.3 | 72.5 | 86.8 |
| 6L | 0.023 | 0.035 | 0.031 | 0.019 | 0.024 | 0.021 | 0.023 |  | 15.6 | 24.3 | 32.6 | 57.4 | 71.6 |
| 5L | 0.026 | 0.024 | 0.030 | 0.015 | 0.023 | 0.020 | 0.020 | 0.024 |  | 8.8 | 17.0 | 41.9 | 56.0 |
| 4L | 0.042 | 0.034 | 0.037 | 0.032 | 0.029 | 0.025 | 0.034 | 0.027 | 0.034 |  | 8.3 | 33.2 | 47.2 |
| 3L | 0.019 | 0.010 | 0.012 | 0.015 | 0.018 | 0.006 | 0.016 | 0.017 | 0.017 | 0.027 |  | 25.0 | 39.0 |
| 2L | 0.020 | 0.014 | 0.018 | 0.017 | 0.017 | 0.009 | 0.024 | 0.024 | 0.019 | 0.031 | 0.009 |  | 14.3 |
| 1L | 0.016 | 0.006 | 0.012 | 0.009 | 0.014 | 0.005 | 0.014 | 0.018 | 0.014 | 0.023 | 0.004 | 0.012 |  |

*From Nazareno et al. 2019a

(G) *Passiflora spinosa**

|  | 1L | 2L | 3L | 4L | 5L | 6L | 7L | 1R | 2R | 3R | 4R | 5R | 6R |
| --- | --- | --- | --- | --- | --- | --- | --- | --- | --- | --- | --- | --- | --- |
| 1L |  | 14.3 | 39.0 | 47.2 | 56.0 | 71.6 | 86.8 | 3.3 | 14.8 | 31.3 | 47.3 | 58.0 | 71.2 |
| 2L | 0.039 |  | 25.0 | 33.2 | 41.9 | 57.4 | 72.5 | 16.1 | 2.0 | 17.3 | 33.1 | 43.8 | 57.0 |
| 3L | 0.051 | 0.010 |  | 8.3 | 17.0 | 32.6 | 48.3 | 41.1 | 25.3 | 3.8 | 9.1 | 19.2 | 32.4 |
| 4L | 0.054 | 0.009 | 0.005 |  | 8.8 | 24.3 | 40.0 | 49.3 | 33.3 | 16.0 | 2.9 | 10.9 | 24.0 |
| 5L | 0.110 | 0.081 | 0.048 | 0.067 |  | 15.6 | 31.6 | 58.0 | 42.1 | 24.7 | 9.3 | 3.2 | 15.5 |
| 6L | 0.067 | 0.040 | 0.038 | 0.031 | 0.081 |  | 16.3 | 73.5 | 57.4 | 40.2 | 24.3 | 13.6 | 1.3 |
| 7L | 0.081 | 0.078 | 0.058 | 0.056 | 0.080 | 0.054 |  | 88.6 | 72.3 | 55.6 | 39.5 | 29.1 | 16.2 |
| 1R | 0.047 | 0.024 | 0.004 | 0.009 | 0.043 | 0.014 | 0.027 |  | 16.2 | 33.4 | 49.2 | 60.0 | 73.1 |
| 2R | 0.032 | 0.020 | 0.014 | 0.006 | 0.032 | 0.022 | 0.030 | 0.017 |  | 17.5 | 33.1 | 43.9 | 57.0 |
| 3R | 0.038 | 0.024 | 0.010 | 0.021 | 0.055 | 0.038 | 0.053 | 0.007 | 0.001 |  | 16.1 | 26.7 | 39.9 |
| 4R | 0.060 | 0.028 | 0.003 | 0.005 | 0.058 | 0.037 | 0.054 | 0.006 | 0.004 | 0.005 |  | 10.8 | 23.9 |
| 5R | 0.029 | 0.015 | 0.006 | 0.002 | 0.049 | 0.019 | 0.042 | 0.003 | 0.019 | 0.008 | 0.006 |  | 13.2 |
| 6R | 0.048 | 0.017 | 0.010 | 0.011 | 0.042 | 0.027 | 0.046 | 0.007 | 0.018 | 0.005 | 0.008 | 0.008 |  |

*From Nazareno et al. 2019a

(H) *Psychotria lupulina**

|  | 1R | 2R | 3R | 4R | 5R | 6R | 7R | 7L | 6L | 5L | 4L | 3L | 2L |
| --- | --- | --- | --- | --- | --- | --- | --- | --- | --- | --- | --- | --- | --- |
| 1R |  | 16.2 | 33.4 | 49.2 | 60.0 | 73.1 | 89.2 | 88.6 | 73.5 | 58.0 | 49.3 | 41.1 | 16.1 |
| 2R | 0.047 |  | 17.5 | 33.1 | 43.9 | 57.0 | 72.9 | 72.3 | 57.4 | 42.1 | 33.3 | 25.3 | 2.0 |
| 3R | 0.056 | 0.013 |  | 16.1 | 26.7 | 39.9 | 56.3 | 55.6 | 40.2 | 24.7 | 16.0 | 3.8 | 17.3 |
| 4R | 0.052 | 0.007 | 0.001 |  | 10.8 | 23.9 | 40.1 | 39.5 | 24.3 | 9.3 | 2.9 | 9.1 | 33.1 |
| 5R | 0.121 | 0.088 | 0.056 | 0.069 |  | 13.2 | 29.8 | 29.1 | 13.6 | 3.2 | 10.9 | 19.2 | 43.8 |
| 6R | 0.078 | 0.042 | 0.039 | 0.024 | 0.088 |  | 17.0 | 16.2 | 1.3 | 15.5 | 24.0 | 32.4 | 57.0 |
| 7R | 0.072 | 0.068 | 0.047 | 0.033 | 0.061 | 0.030 |  | 1.1 | 17.1 | 32.3 | 40.7 | 49.0 | 73.1 |
| 7L | 0.060 | 0.026 | 0.016 | 0.012 | 0.059 | 0.030 | 0.039 |  | 16.3 | 31.6 | 40.0 | 48.3 | 72.5 |
| 6L | 0.040 | 0.028 | 0.010 | 0.015 | 0.042 | 0.034 | 0.019 | 0.013 |  | 15.6 | 24.3 | 32.6 | 57.4 |
| 5L | 0.033 | 0.019 | 0.008 | 0.002 | 0.067 | 0.039 | 0.045 | 0.017 | 0.001 |  | 8.8 | 17.0 | 41.9 |
| 4L | 0.038 | 0.014 | 0.013 | 0.011 | 0.043 | 0.021 | 0.028 | 0.010 | 0.014 | 0.015 |  | 8.3 | 33.2 |
| 3L | 0.048 | 0.017 | 0.001 | 0.002 | 0.061 | 0.021 | 0.031 | 0.004 | 0.001 | 0.001 | 0.022 |  | 25.0 |
| 2L | 0.034 | 0.005 | 0.028 | 0.012 | 0.042 | 0.012 | 0.002 | 0.013 | 0.025 | 0.013 | 0.025 | 0.026 |  |

*From Nazareno et al. 2019a

**TABLE S5** Values of the Pearson’s correlation coefficient and *P*-values for the correlation between pairwise genetic differentiation (*F*_ST_) and geographic distance sampled along both banks of the Rio Branco (Amazon Basin, Brazil).

| **Plant species** | **Right river bank** | | **Left river bank** | |
| --- | --- | --- | --- | --- |
|  | ***r*** | ***P-value*** | ***r*** | ***P-value*** |
| 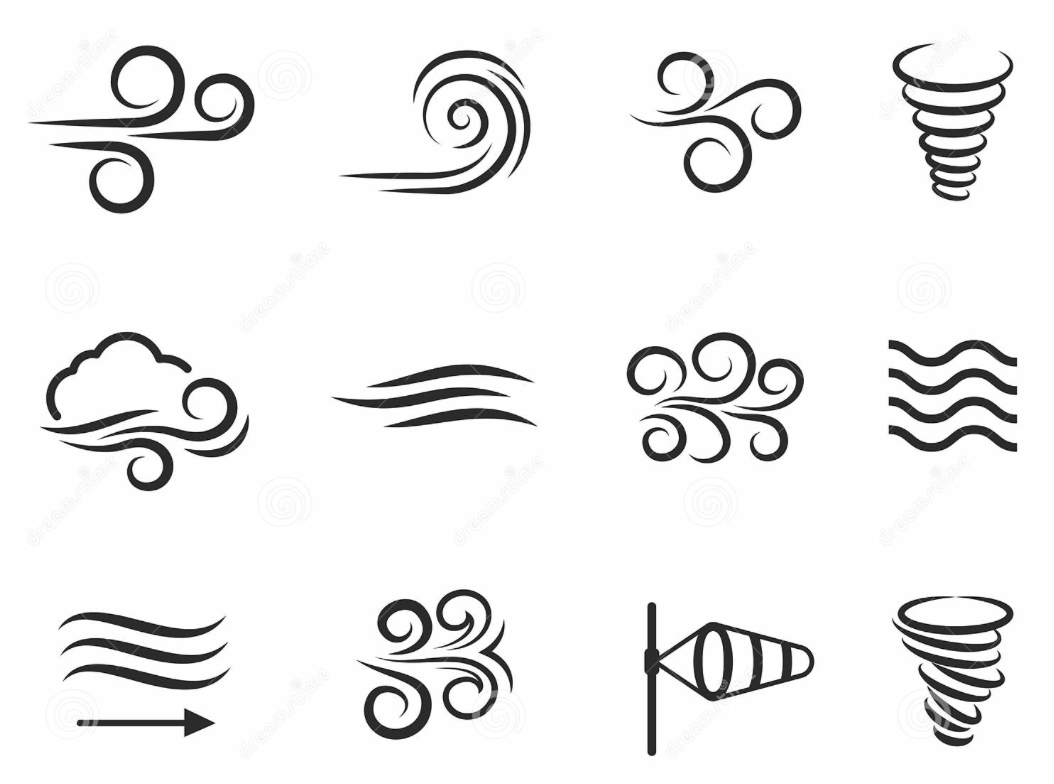  *Adenocalymma schomburgkii* | -0.340 | 0.908 | 0.059 | 0.417 |
| 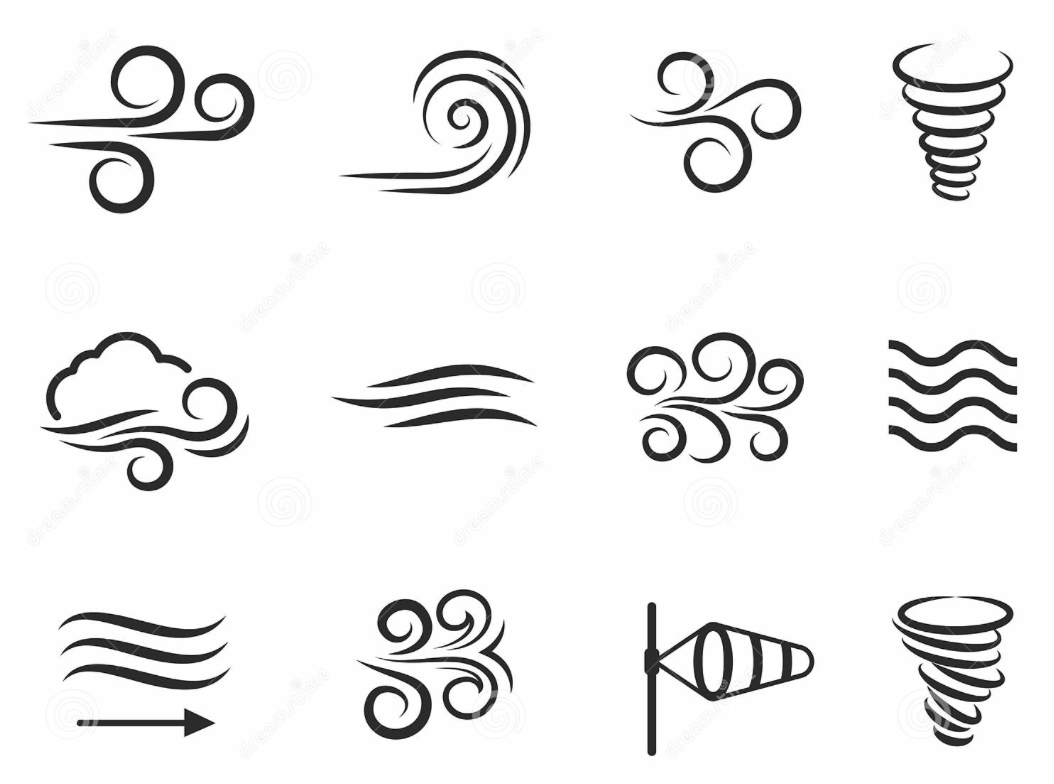*Bignonia aequinoctialis* | 0.073 | 0.335 | -0.112 | 0.648 |
| 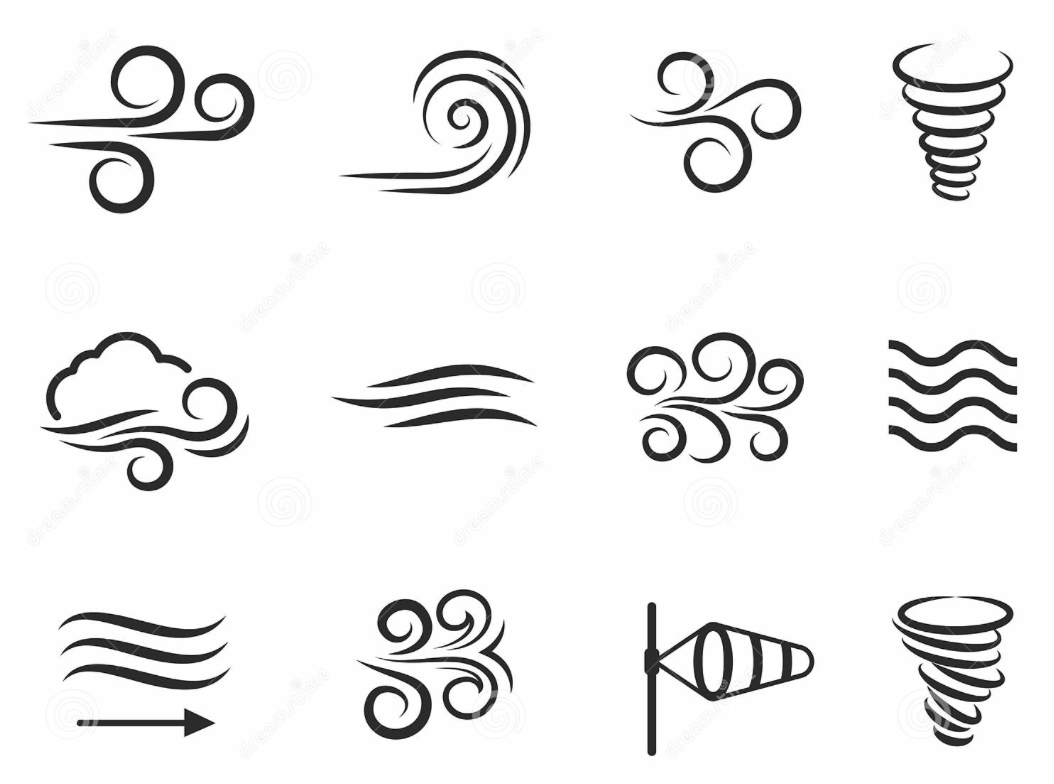*Tanaecium pyramidatum* | -0.245 | 0.793 | 0.240 | 0.196 |
| 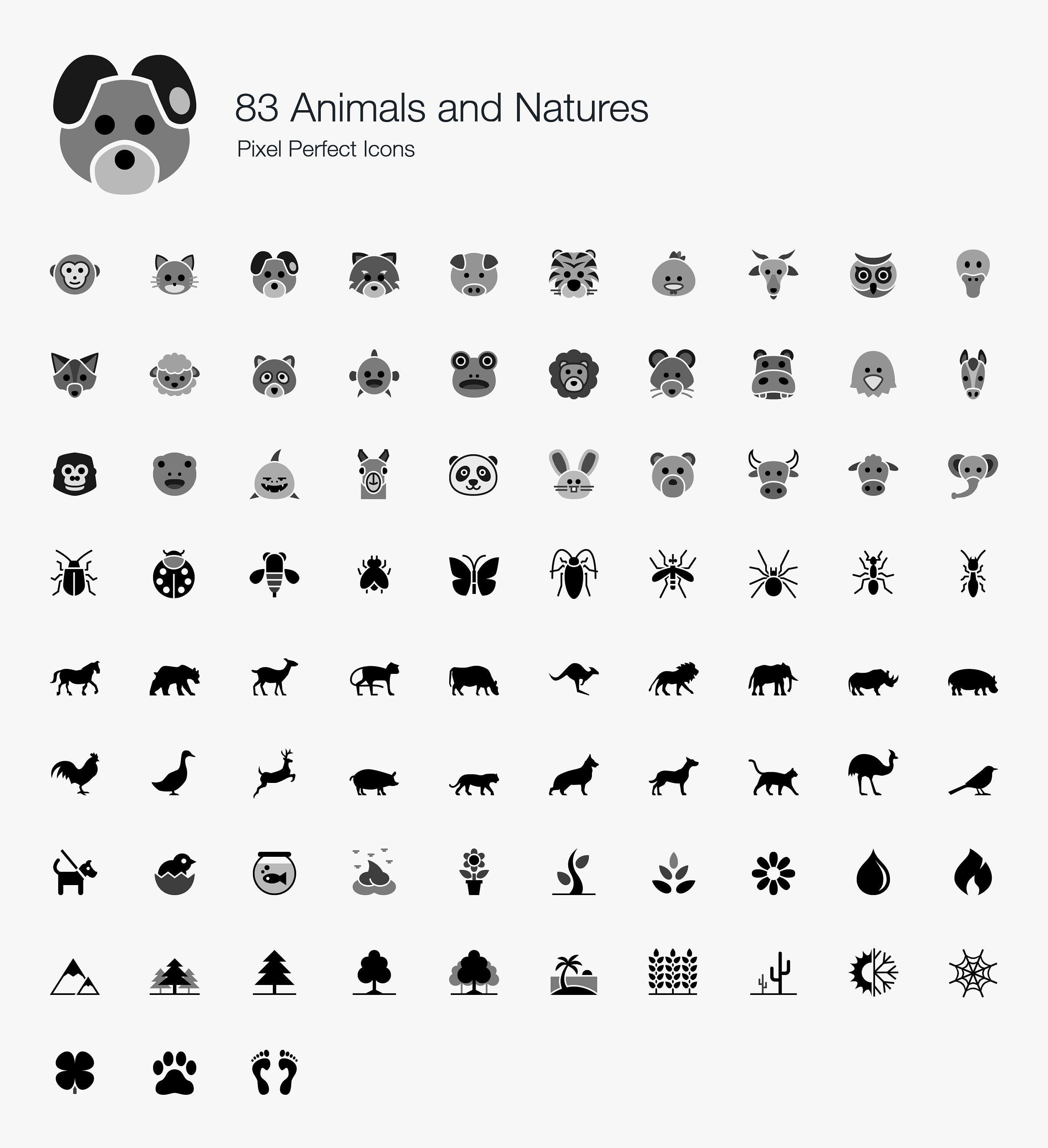  *Anemopaegma paraense* | -0.009 | 0.516 | 0.467 | 0.073 |
| 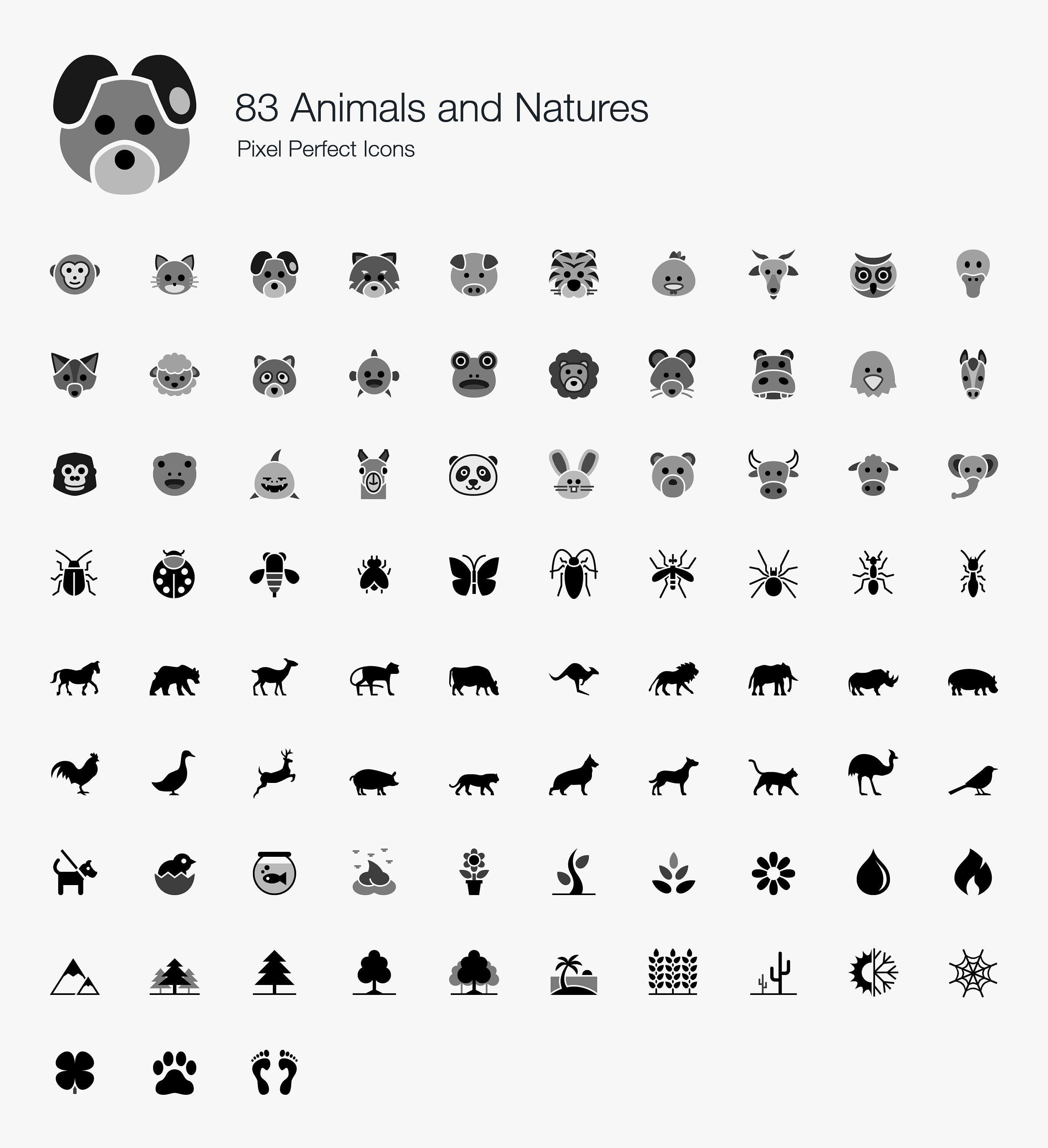  *Pachypetra kerere* | -0.236 | 0.843 | 0.335 | 0.086 |
| *Amphirrhox longifolia* | -0.053 | 0.624 | -0.123 | 0.702 |
| 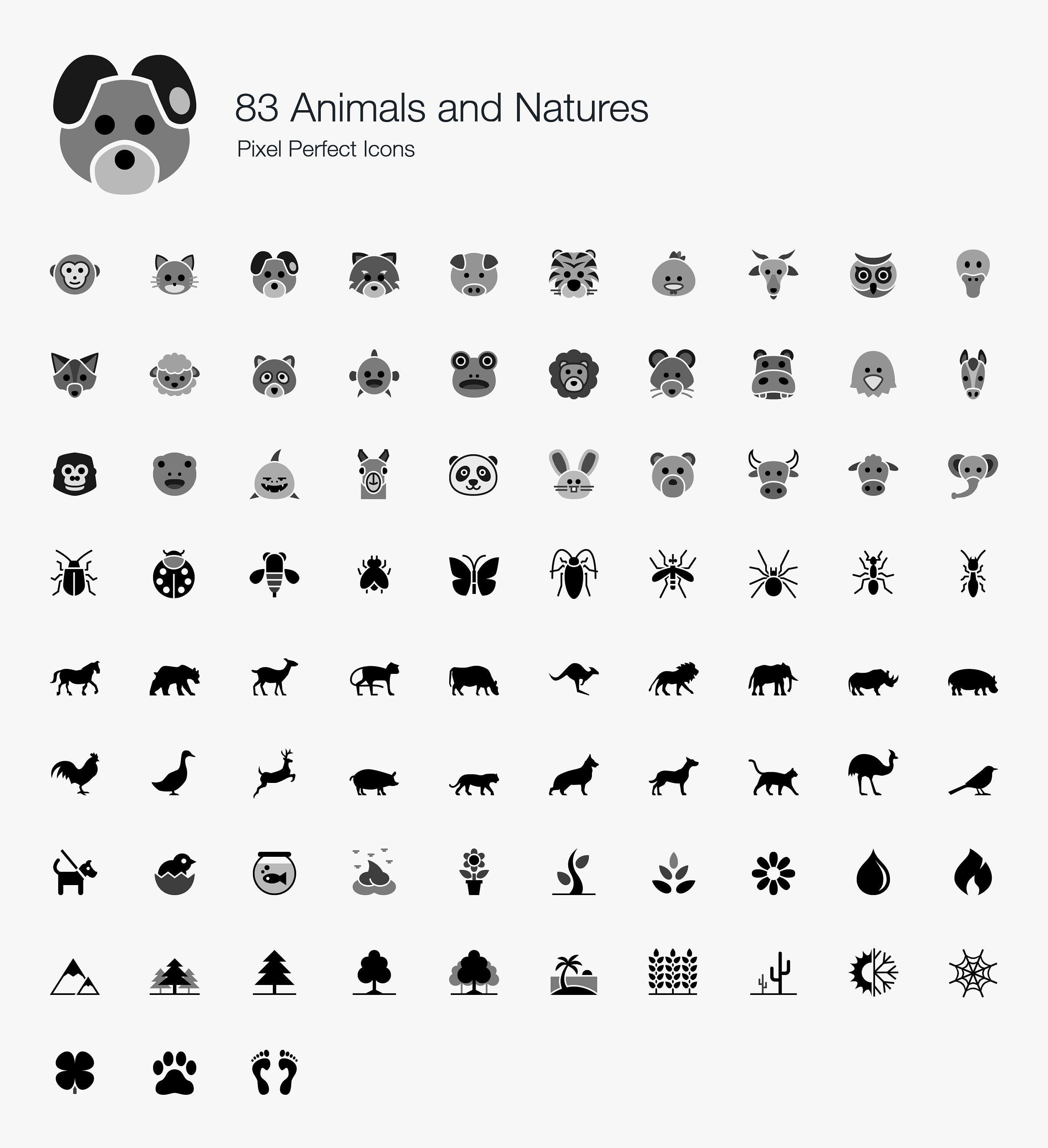*Passiflora spinosa* | 0.213 | 0.486 | 0.458 | 0.091 |
| 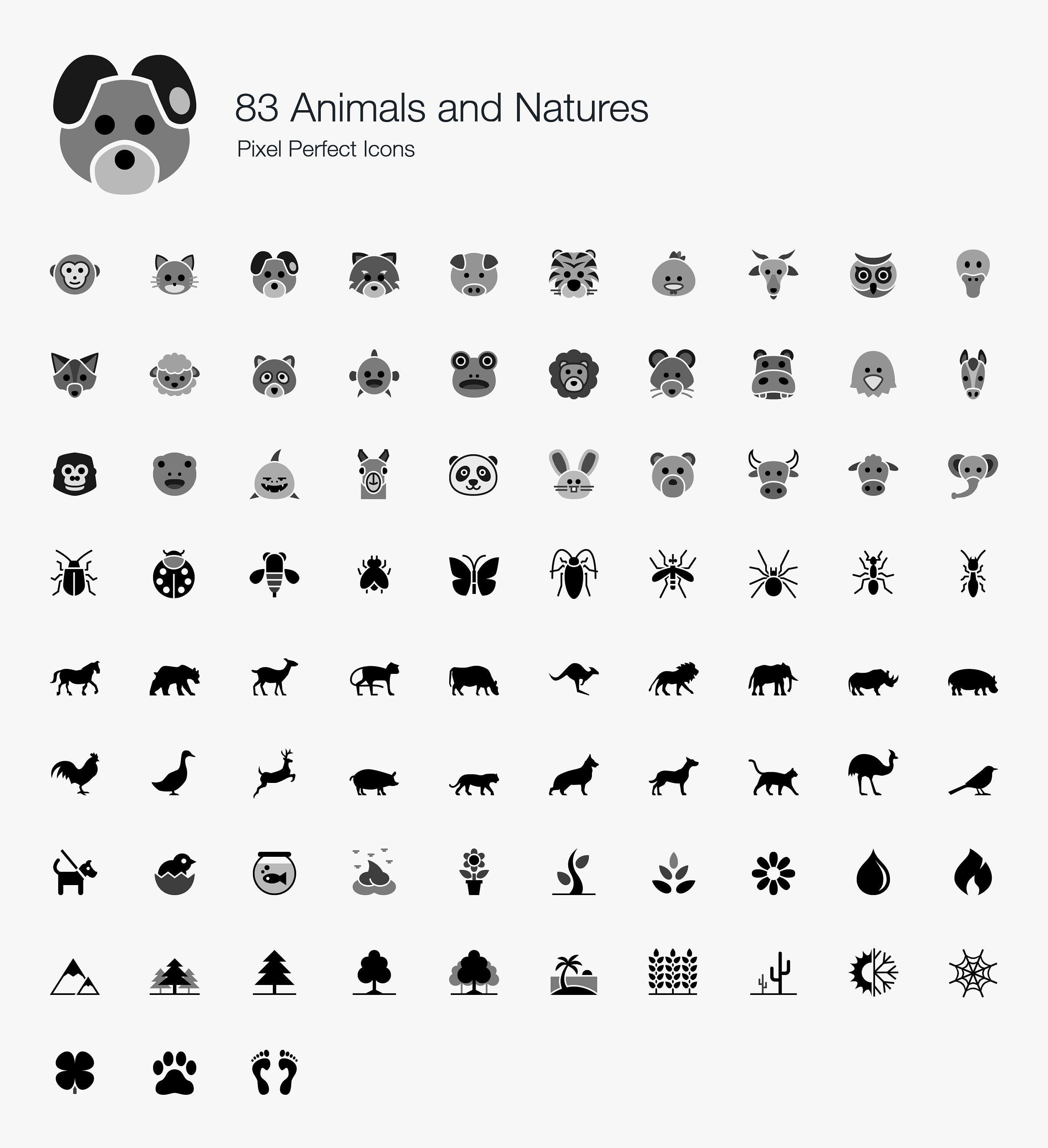  *Psychotria lupulina* | 0.418 | 0.103 | 0.052 | 0.899 |

**TABLE S6** Analysis of Molecular Variance (AMOVA) for each plant species along the Rio Branco, Amazon Basin, Brazil.

| **Plant species** | |  | | Sum of  squares | Variance  components | | | % of Variation | *P-*value |  |
| --- | --- | --- | --- | --- | --- | --- | --- | --- | --- | --- |
| *Adenocalymma schomburgkii* | | *F*_ST_ | | 262.59 | 0.23 | | | 0.10 | 1.000 |  |
| 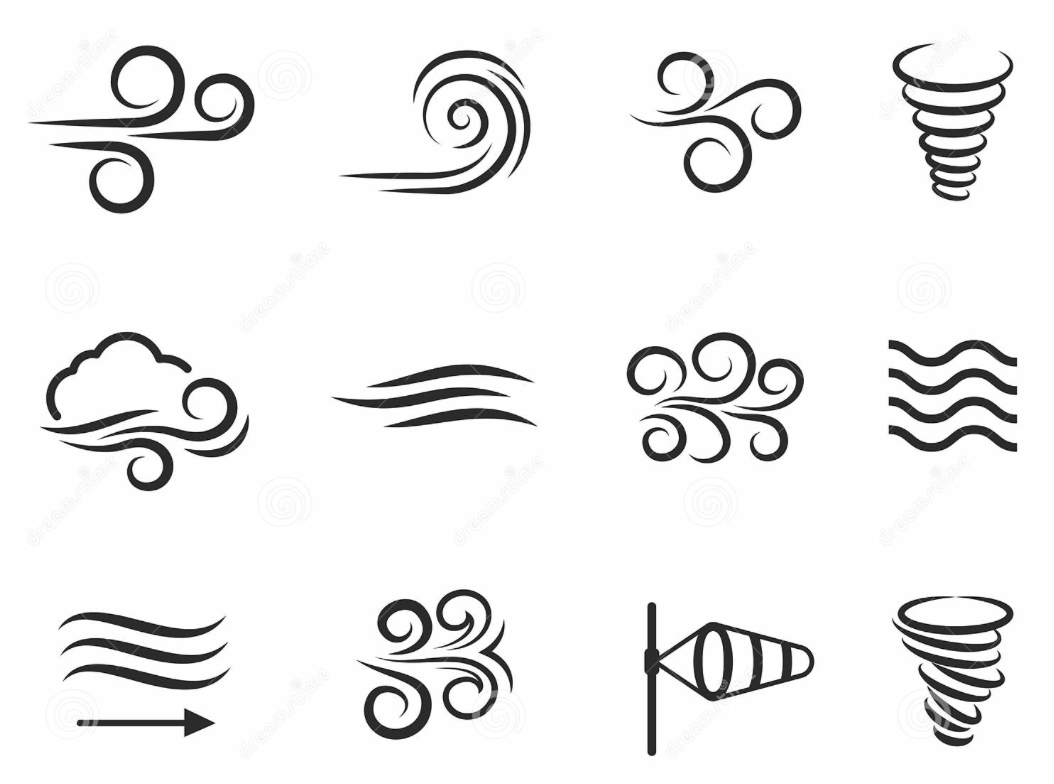 | | *F*_SC_ | | 14133.02 | 10.85 | | | 4.55 | 0.000 |  |
|  | | *F*_CT_ | | 13367.50 | 227.05 | | | 95.34 | 0.000 |  |
|  | |  | |  |  | | |  |  |  |
| *Bignonia aequinoctialis* | | *F*_ST_ | | 537.83 | 3.08 | | | 0.99 | 1.000 |  |
| 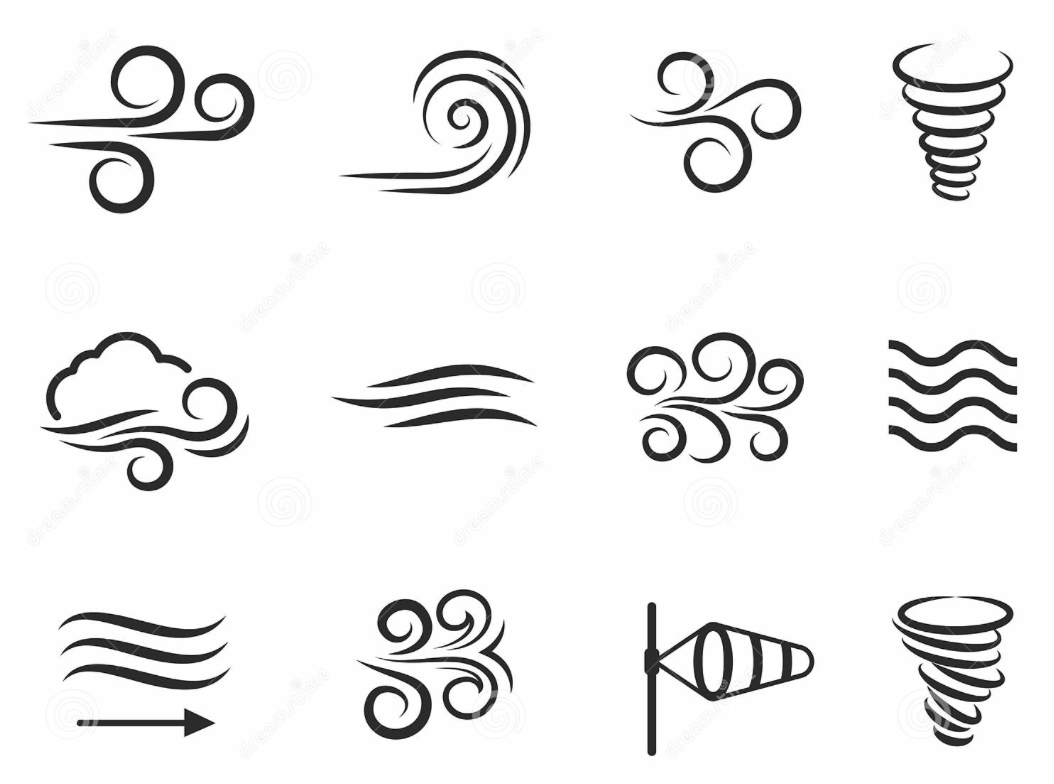 | | *F*_SC_ | | 21688.61 | 19.42 | | | 6.24 | 0.000 |  |
|  | | *F*_CT_ | | 19776.50 | 288.54 | | | 92.76 | 0.000 |  |
|  | |  | |  |  | | |  |  |  |
| *Tanaecium pyramidatum* | | *F*_ST_ | | 491.49 | 1.68 | | | 0.46 | 1.000 |  |
| 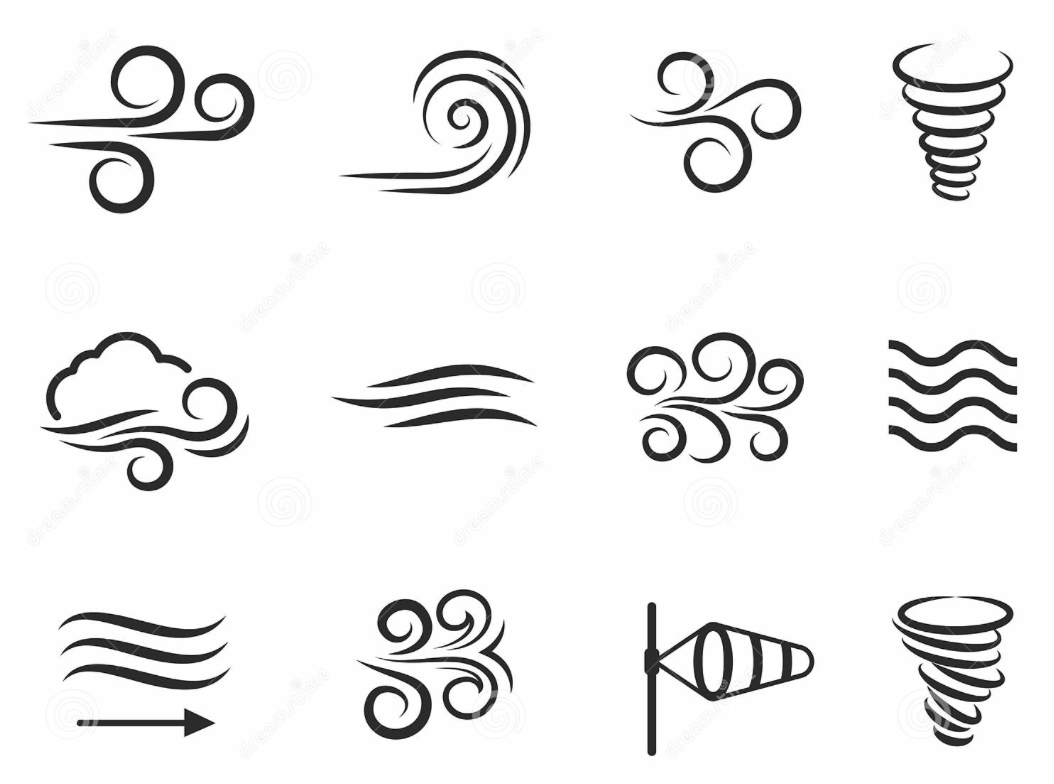 | | *F*_SC_ | | 26711.69 | 9.54 | | | 2.66 | 0.000 |  |
|  | | *F*_CT_ | | 26037.00 | 347.59 | | | 96.88 | 0.000 |  |
|  | |  | |  |  | | |  |  |  |
| *Anemopaegma paraense* | | *F*_ST_ | | 6736.10 | 31.43 | | | 0.74 | 1.000 |  |
| 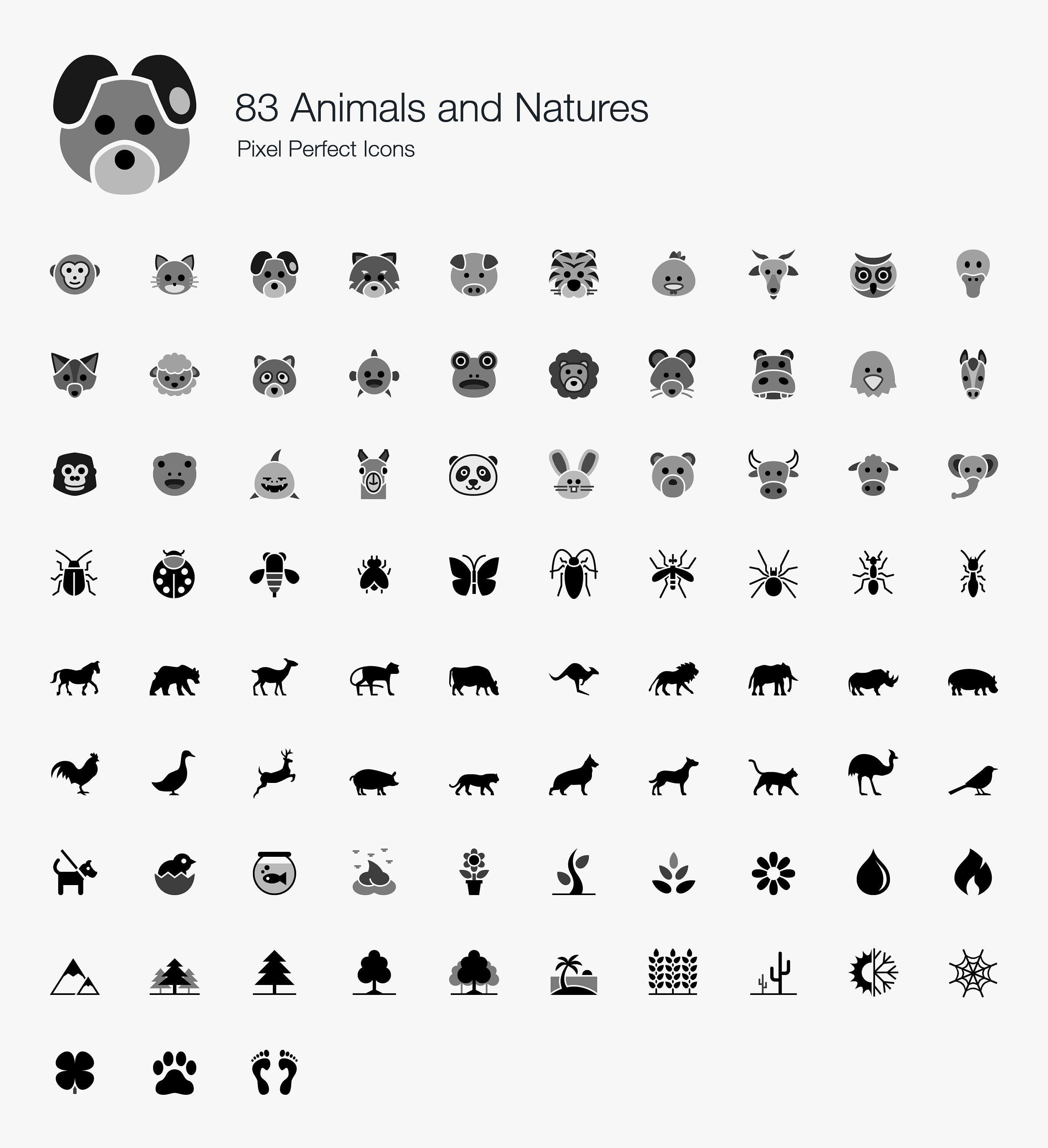 | | *F*_SC_ | | 318824.83 | 162.22 | | | 3.84 | 0.000 |  |
|  | | *F*_CT_ | | 304016.00 | 4030.68 | | | 95.42 | 0.000 |  |
|  | |  | |  |  | | |  |  |  |
| *Pachyptera kerere* | | *F*_ST_ | | 376.55 | 0.09 | | | 0.02 | 1.000 |  |
| 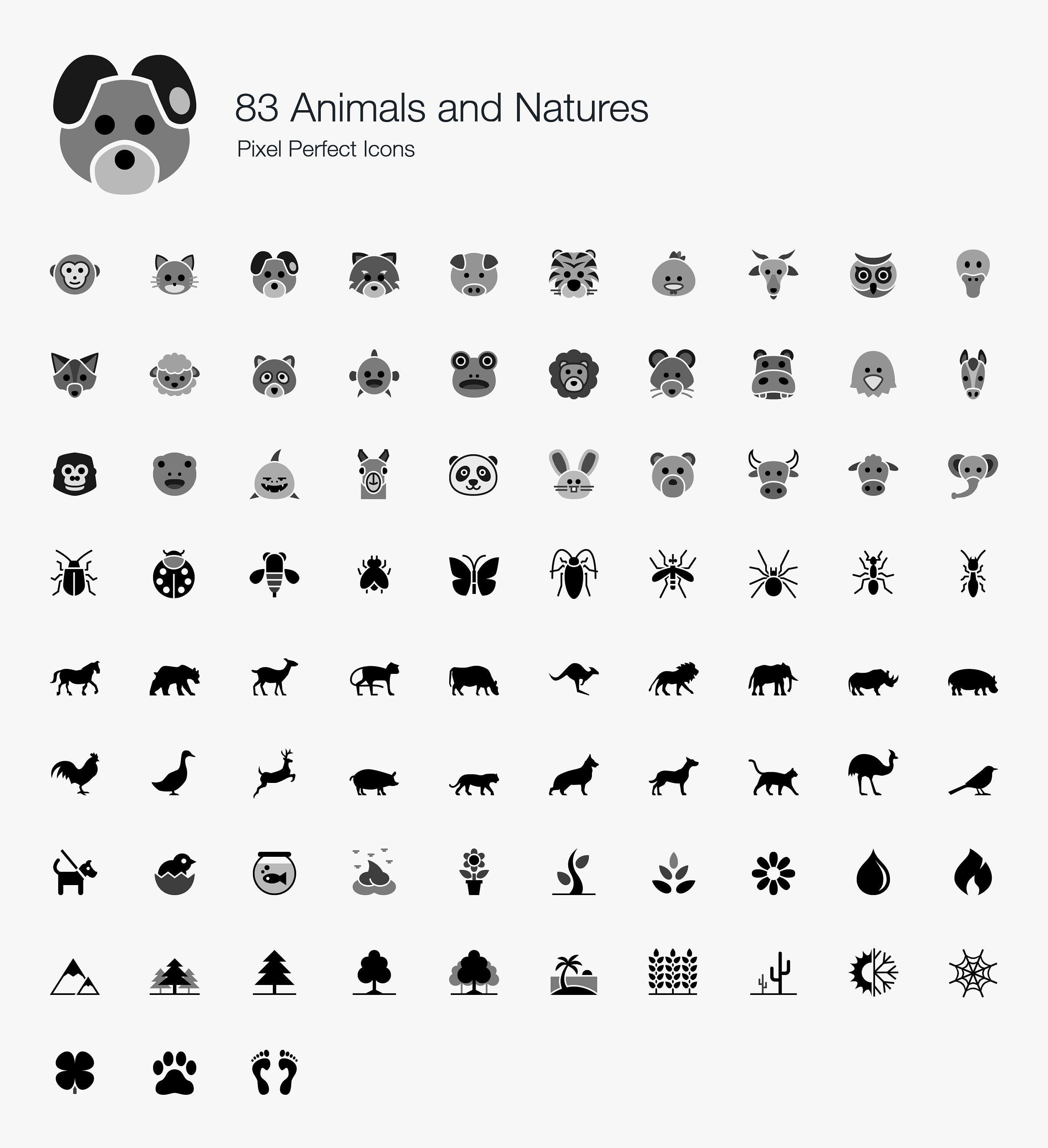 | | *F*_SC_ | | 30373.63 | 3.18 | | | 0.83 | 0.000 |  |
|  | | *F*_CT_ | | 30641.00 | 377.27 | | | 99.15 | 0.000 |  |
|  | |  | |  |  | | |  |  |  |
| *Amphirrhox longifolia** | | *F*_ST_ | | 77.71 | 0.01 | | | 0.01 | 0.365 |  |
|  | | *F*_SC_ | | 842.41 | 0.086 | | | 1.26 | <0.001 |  |
|  | | *F*_CT_ | | 8135.12 | 67.32 | | | 98.73 | <0.001 |  |
|  | |  | |  |  | | |  |  |  |
| *Passiflora spinosa** | | *F*_ST_ | | 1164.15 | 0.01 | | | 0.43 | 0.322 |  |
| 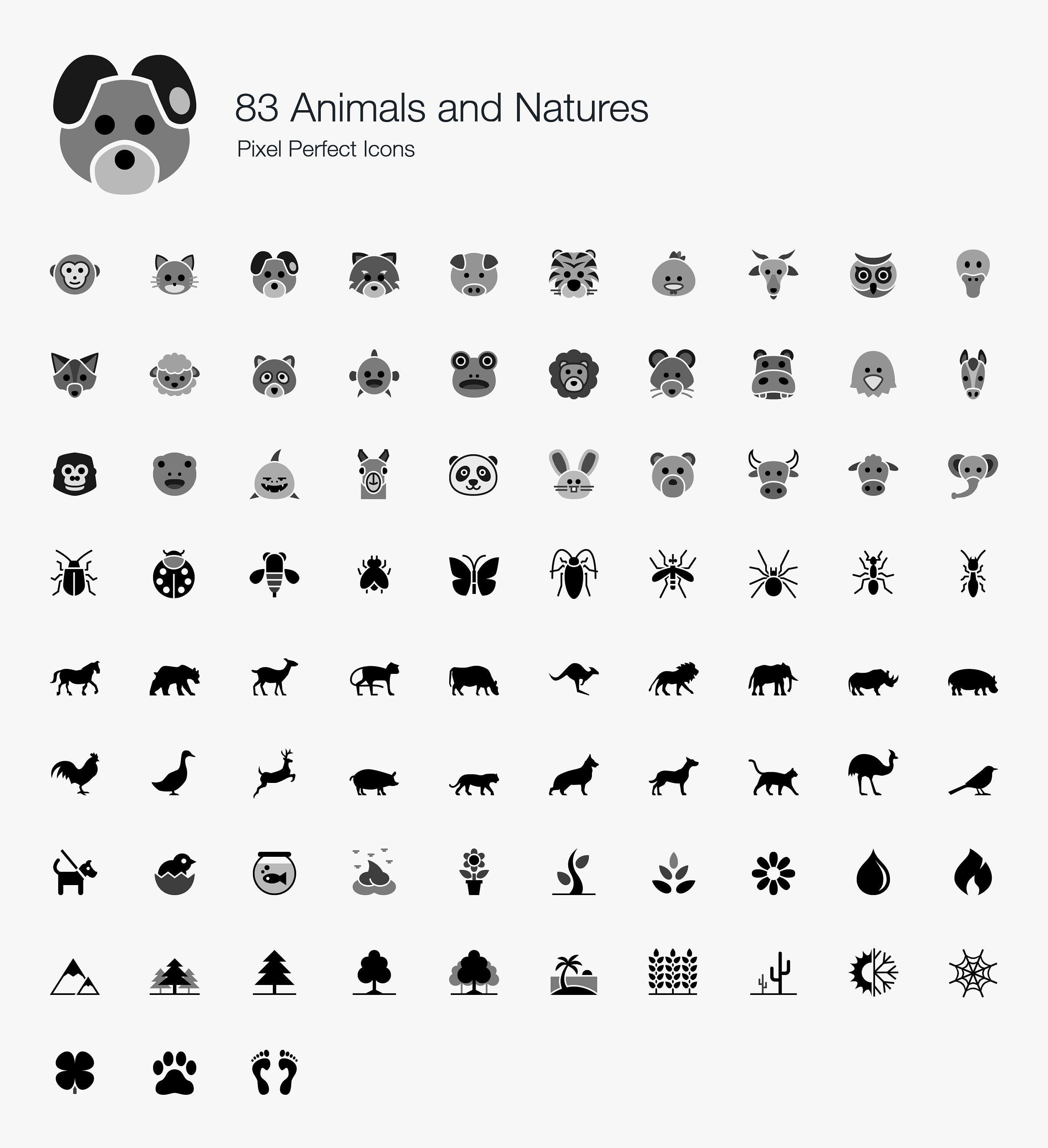 | | *F*_SC_ | | 21742.68 | 10.09 | | | 1.00 | 0.012 |  |
|  | | *F*_CT_ | | 152112.42 | 1159.71 | | | 98.57 | 0.007 |  |
|  | |  | |  |  | | |  |  |  |
| *Psychotria lupulina** | | *F*_ST_ | | 847.08 | 0.35 | | | 0.05 | 0.422 |  |
| 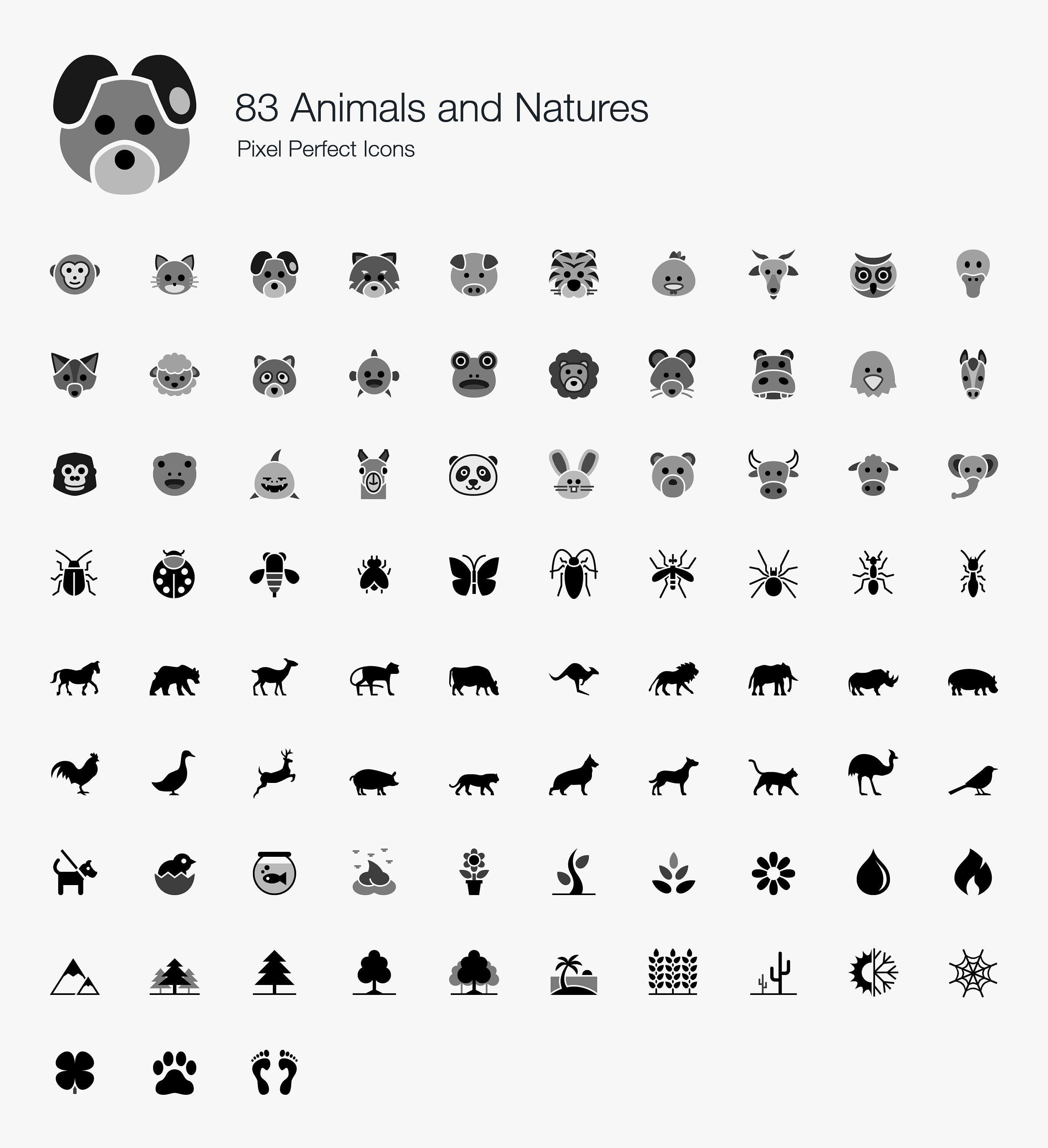 | | *F*_SC_ | | 9062.39 | 15.70 | | | 2.32 | 0.000 |  |
|  | | *F*_CT_ | | 80725.85 | 661.84 | | | 97.63 | <0.001 |  |
|  |  | |  | | |  |  | |  | |

Genetic differentiation between river banks (*F*_ST_), among sampling locations within river banks (*F*_SC_), and within sampling locations (*F*_CT_).

*From Nazareno et al. 2019a

**TABLE S7** Directional gene flow estimates (m) for pairs of sampling locations along the Rio Branco (Amazon Basin, Brazil) for each plant species. The 95% confidence interval (CI) are also presented. All migration rates whose 95% confidence intervals did not include zero are reported as significant. Pairs of sampling locations with asymmetric short-term estimates of gene flow (i.e., non-overlapping 95% CI) are in bold.

(A) *Adenocalymma schomburgkii*

| **Site pairs** | **m** | **CI95%-** | **CI95%+** | **Site pairs** | **m** | **CI95%-** | **CI95%+** |
| --- | --- | --- | --- | --- | --- | --- | --- |
| **m[2R][1R]** | 0.0166 | -0.0144 | 0.0476 | **m[1L][4R]** | 0.0176 | -0.0143 | 0.0495 |
| **m[3R][1R]** | 0.0204 | -0.0155 | 0.0563 | **m[2L][4R]** | 0.0172 | -0.0136 | 0.0480 |
| **m[4R][1R]** | 0.0247 | -0.0174 | 0.0668 | **m[3L][4R]** | 0.0200 | -0.0200 | 0.0600 |
| **m[5R][1R]** | 0.0197 | -0.0122 | 0.0516 | **m[4L][4R]** | 0.0208 | -0.0157 | 0.0573 |
| **m[6R][1R]** | 0.0227 | -0.0189 | 0.0643 | **m[5L][4R]** | 0.0180 | -0.0149 | 0.0509 |
| **m[7R][1R]** | 0.0202 | -0.0157 | 0.0561 | **m[6L][4R]** | 0.0158 | -0.0132 | 0.0448 |
| **m[1L][1R]** | 0.0204 | -0.0168 | 0.0576 | **m[7L][4R]** | 0.0200 | -0.0159 | 0.0559 |
| **m[2L][1R]** | 0.0153 | -0.0170 | 0.0476 | **m[1R][5R]** | 0.0167 | -0.0143 | 0.0477 |
| **m[3L][1R]** | 0.0176 | -0.0159 | 0.0511 | **m[2R][5R]** | 0.0158 | -0.0165 | 0.0481 |
| **m[4L][1R]** | 0.0183 | -0.0097 | 0.0463 | **m[3R][5R]** | 0.0200 | -0.0221 | 0.0621 |
| **m[5L][1R]** | 0.0165 | -0.0154 | 0.0484 | **m[4R][5R]** | 0.0214 | -0.0178 | 0.0606 |
| **m[6L][1R]** | 0.0184 | -0.0122 | 0.0490 | **m[6R][5R]** | 0.0226 | -0.0164 | 0.0616 |
| **m[7L][1R]** | 0.0155 | -0.0184 | 0.0494 | **m[7R][5R]** | 0.0216 | -0.0156 | 0.0588 |
| **m[1R][2R]** | 0.0415 | 0.0019 | 0.0811 | **m[1L][5R]** | 0.0225 | -0.0196 | 0.0646 |
| **m[3R][2R]** | 0.0193 | -0.0179 | 0.0565 | **m[2L][5R]** | 0.0185 | -0.0109 | 0.0479 |
| **m[4R][2R]** | 0.0239 | -0.0218 | 0.0696 | **m[3L][5R]** | 0.0171 | -0.0196 | 0.0538 |
| **m[5R][2R]** | 0.0209 | -0.0185 | 0.0603 | **m[4L][5R]** | 0.0187 | -0.0132 | 0.0506 |
| **m[6R][2R]** | 0.0186 | -0.0167 | 0.0539 | **m[5L][5R]** | 0.0320 | -0.0117 | 0.0757 |
| **m[7R][2R]** | 0.0157 | -0.0127 | 0.0441 | **m[6L][5R]** | 0.0153 | -0.0125 | 0.0431 |
| **m[1L][2R]** | 0.0212 | -0.0170 | 0.0594 | **m[7L][5R]** | 0.0165 | -0.0162 | 0.0492 |
| **m[2L][2R]** | 0.0166 | -0.0103 | 0.0435 | **m[1R][6R]** | 0.0154 | -0.0120 | 0.0428 |
| **m[3L][2R]** | 0.0167 | -0.0105 | 0.0439 | **m[2R][6R]** | 0.0138 | -0.0117 | 0.0393 |
| **m[4L][2R]** | 0.0628 | 0.0081 | 0.1175 | **m[3R][6R]** | 0.0192 | -0.0216 | 0.0600 |
| **m[5L][2R]** | 0.0251 | -0.0204 | 0.0706 | **m[4R][6R]** | 0.0201 | -0.0209 | 0.0611 |
| **m[6L][2R]** | 0.0165 | -0.0145 | 0.0475 | **m[5R][6R]** | 0.0189 | -0.0142 | 0.0520 |
| **m[7L][2R]** | 0.0185 | -0.0207 | 0.0577 | **m[7R][6R]** | 0.0155 | -0.0153 | 0.0463 |
| **m[1R][3R]** | 0.0178 | -0.0173 | 0.0529 | **m[1L][6R]** | 0.0222 | -0.0129 | 0.0573 |
| **m[2R][3R]** | 0.0155 | -0.0114 | 0.0424 | **m[2L][6R]** | 0.0186 | -0.0096 | 0.0468 |
| **m[4R][3R]** | 0.0245 | -0.0292 | 0.0782 | **m[3L][6R]** | 0.0177 | -0.0154 | 0.0508 |
| **m[5R][3R]** | 0.0182 | -0.0173 | 0.0537 | **m[4L][6R]** | 0.0216 | -0.0211 | 0.0643 |
| **m[6R][3R]** | 0.0205 | -0.0126 | 0.0536 | **m[5L][6R]** | 0.0178 | -0.0173 | 0.0529 |
| **m[7R][3R]** | 0.0213 | -0.0193 | 0.0619 | **m[6L][6R]** | 0.0196 | -0.0172 | 0.0564 |
| **m[1L][3R]** | 0.0215 | -0.0187 | 0.0617 | **m[7L][6R]** | 0.0186 | -0.0182 | 0.0554 |
| **m[2L][3R]** | 0.0186 | -0.0116 | 0.0488 | **m[1R][7R]** | 0.0151 | -0.0159 | 0.0461 |
| **m[3L][3R]** | 0.0190 | -0.0092 | 0.0472 | **m[2R][7R]** | 0.0147 | -0.0145 | 0.0439 |

| **Site pairs** | **m** | **CI95%-** | **CI95%+** | **Site pairs** | **m** | **CI95%-** | **CI95%+** |
| --- | --- | --- | --- | --- | --- | --- | --- |
| **m[4L][3R]** | 0.0189 | -0.0134 | 0.0512 | **m[3R][7R]** | 0.0192 | -0.0169 | 0.0553 |
| **m[5L][3R]** | 0.0172 | -0.0161 | 0.0505 | **m[4R][7R]** | 0.0215 | -0.0214 | 0.0644 |
| **m[6L][3R]** | 0.0143 | -0.0139 | 0.0425 | **m[5R][7R]** | 0.0183 | -0.0154 | 0.0520 |
| **m[7L][3R]** | 0.0169 | -0.0139 | 0.0477 | **m[6R][7R]** | 0.0190 | -0.0135 | 0.0515 |
| **m[1R][4R]** | 0.0184 | -0.0159 | 0.0527 | **m[1L][7R]** | 0.0216 | -0.0186 | 0.0618 |
| **m[2R][4R]** | 0.0168 | -0.0093 | 0.0429 | **m[2L][7R]** | 0.0170 | -0.0159 | 0.0499 |
| **m[3R][4R]** | 0.0155 | -0.0125 | 0.0435 | **m[3L][7R]** | 0.0221 | -0.0149 | 0.0591 |
| **m[5R][4R]** | 0.0240 | -0.0146 | 0.0626 | **m[4L][7R]** | 0.0174 | -0.0193 | 0.0541 |
| **m[6R][4R]** | 0.0188 | -0.0137 | 0.0513 | **m[5L][7R]** | 0.0139 | -0.0131 | 0.0409 |
| **m[7R][4R]** | 0.0165 | -0.0139 | 0.0469 | **m[6L][7R]** | 0.0179 | -0.0097 | 0.0455 |
| **m[7L][7R]** | 0.0170 | -0.0100 | 0.0440 | **m[6R][4L]** | 0.0189 | -0.0166 | 0.0544 |
| **m[1R][1L]** | 0.0151 | -0.0086 | 0.0388 | **m[7R][4L]** | 0.0194 | -0.0112 | 0.0500 |
| **m[2R][1L]** | 0.0158 | -0.0140 | 0.0456 | **m[1L][4L]** | 0.0195 | -0.0148 | 0.0538 |
| **m[3R][1L]** | 0.0152 | -0.0097 | 0.0401 | **m[2L][4L]** | 0.0155 | -0.0149 | 0.0459 |
| **m[4R][1L]** | 0.0218 | -0.0235 | 0.0671 | **m[3L][4L]** | 0.0151 | -0.0151 | 0.0453 |
| **m[5R][1L]** | 0.0187 | -0.0123 | 0.0497 | **m[5L][4L]** | 0.0152 | -0.0101 | 0.0405 |
| **m[6R][1L]** | 0.0192 | -0.0169 | 0.0553 | **m[6L][4L]** | 0.0175 | -0.0129 | 0.0479 |
| **m[7R][1L]** | 0.0191 | -0.0156 | 0.0538 | **m[7L][4L]** | 0.0140 | -0.0125 | 0.0405 |
| **m[2L][1L]** | 0.0189 | -0.0140 | 0.0518 | **m[1R][5L]** | 0.0153 | -0.0159 | 0.0465 |
| **m[3L][1L]** | 0.0195 | -0.0154 | 0.0544 | **m[2R][5L]** | 0.0202 | -0.0141 | 0.0545 |
| **m[4L][1L]** | 0.0142 | -0.0101 | 0.0385 | **m[3R][5L]** | 0.0206 | -0.0157 | 0.0569 |
| **m[5L][1L]** | 0.0177 | -0.0133 | 0.0487 | **m[4R][5L]** | 0.0213 | -0.0238 | 0.0664 |
| **m[6L][1L]** | 0.0203 | -0.0138 | 0.0544 | **m[5R][5L]** | 0.0182 | -0.0200 | 0.0564 |
| **m[7L][1L]** | 0.0171 | -0.0223 | 0.0565 | **m[6R][5L]** | 0.0224 | -0.0168 | 0.0616 |
| **m[1R][2L]** | 0.0164 | -0.0173 | 0.0501 | **m[7R][5L]** | 0.0198 | -0.0159 | 0.0555 |
| **m[2R][2L]** | 0.0163 | -0.0117 | 0.0443 | **m[1L][5L]** | 0.0190 | -0.0169 | 0.0549 |
| **m[3R][2L]** | 0.0186 | -0.0151 | 0.0523 | **m[2L][5L]** | 0.0161 | -0.0100 | 0.0422 |
| **m[4R][2L]** | 0.0275 | -0.0217 | 0.0767 | **m[3L][5L]** | 0.0184 | -0.0134 | 0.0502 |
| **m[5R][2L]** | 0.0160 | -0.0142 | 0.0462 | **m[4L][5L]** | 0.0182 | -0.0159 | 0.0523 |
| **m[6R][2L]** | 0.0189 | -0.0207 | 0.0585 | **m[6L][5L]** | 0.0166 | -0.0183 | 0.0515 |
| **m[7R][2L]** | 0.0183 | -0.0121 | 0.0487 | **m[7L][5L]** | 0.0156 | -0.0150 | 0.0462 |
| **m[1L][2L]** | 0.0192 | -0.0135 | 0.0519 | **m[1R][6L]** | 0.0211 | -0.0165 | 0.0587 |
| **m[3L][2L]** | 0.0193 | -0.0121 | 0.0507 | **m[2R][6L]** | 0.0171 | -0.0084 | 0.0426 |
| **m[4L][2L]** | 0.0194 | -0.0155 | 0.0543 | **m[3R][6L]** | 0.0171 | -0.0131 | 0.0473 |
| **m[5L][2L]** | 0.0151 | -0.0155 | 0.0457 | **m[4R][6L]** | 0.0218 | -0.0135 | 0.0571 |
| **m[6L][2L]** | 0.0176 | -0.0151 | 0.0503 | **m[5R][6L]** | 0.0164 | -0.0150 | 0.0478 |
| **m[7L][2L]** | 0.0170 | -0.0226 | 0.0566 | **m[6R][6L]** | 0.0193 | -0.0174 | 0.0560 |
| **m[1R][3L]** | 0.0180 | -0.0120 | 0.0480 | **m[7R][6L]** | 0.0187 | -0.0138 | 0.0512 |
| **m[2R][3L]** | 0.0166 | -0.0157 | 0.0489 | **m[1L][6L]** | 0.0169 | -0.0127 | 0.0465 |
| **m[3R][3L]** | 0.0194 | -0.0141 | 0.0529 | **m[2L][6L]** | 0.0156 | -0.0126 | 0.0438 |
| **m[4R][3L]** | 0.0236 | -0.0252 | 0.0724 | **m[3L][6L]** | 0.0185 | -0.0185 | 0.0555 |
| **m[5R][3L]** | 0.0188 | -0.0112 | 0.0488 | **m[4L][6L]** | 0.0320 | -0.0090 | 0.0730 |

| **Site pairs** | **m** | **CI95%-** | **CI95%+** | **Site pairs** | **m** | **CI95%-** | **CI95%+** |
| --- | --- | --- | --- | --- | --- | --- | --- |
| **m[6R][3L]** | 0.0180 | -0.0181 | 0.0541 | **m[5L][6L]** | 0.0183 | -0.0166 | 0.0532 |
| **m[7R][3L]** | 0.0186 | -0.0102 | 0.0474 | **m[7L][6L]** | 0.0152 | -0.0101 | 0.0405 |
| **m[1L][3L]** | 0.0197 | -0.0240 | 0.0634 | **m[1R][7L]** | 0.0360 | -0.0124 | 0.0844 |
| **m[2L][3L]** | 0.0149 | -0.0141 | 0.0439 | **m[2R][7L]** | 0.0147 | -0.0143 | 0.0437 |
| **m[4L][3L]** | 0.0180 | -0.0145 | 0.0505 | **m[3R][7L]** | 0.0164 | -0.0091 | 0.0419 |
| **m[5L][3L]** | 0.0174 | -0.0096 | 0.0444 | **m[4R][7L]** | 0.0217 | -0.0232 | 0.0666 |
| **m[6L][3L]** | 0.0181 | -0.0199 | 0.0561 | **m[5R][7L]** | 0.0214 | -0.0166 | 0.0594 |
| **m[7L][3L]** | 0.0172 | -0.0085 | 0.0429 | **m[6R][7L]** | 0.0206 | -0.0210 | 0.0622 |
| **m[1R][4L]** | 0.0188 | -0.0131 | 0.0507 | **m[7R][7L]** | 0.0205 | -0.0163 | 0.0573 |
| **m[2R][4L]** | 0.0178 | -0.0192 | 0.0548 | **m[1L][7L]** | 0.0158 | -0.0134 | 0.0450 |
| **m[3R][4L]** | 0.0194 | -0.0145 | 0.0533 | **m[2L][7L]** | 0.0185 | -0.0127 | 0.0497 |
| **m[4R][4L]** | 0.0161 | -0.0149 | 0.0471 | **m[3L][7L]** | 0.0196 | -0.0155 | 0.0547 |
| **m[5R][4L]** | 0.0150 | -0.0144 | 0.0444 | **m[4L][7L]** | 0.0163 | -0.0155 | 0.0481 |
|  |  |  |  | **m[5L][7L]** | 0.0342 | -0.0068 | 0.0752 |
|  |  |  |  | **m[6L][7L]** | 0.0186 | -0.0214 | 0.0586 |

(B) *Anemopaegma paraense*

| **Site pairs** | **m** | **CI95%-** | **CI95%+** | **Site pairs** | **m** | **CI95%-** | **CI95%+** |
| --- | --- | --- | --- | --- | --- | --- | --- |
| **m[2R][1R]** | 0.0321 | -0.0063 | 0.0705 | **m[1L][4R]** | 0.0181 | -0.0199 | 0.0561 |
| **m[3R][1R]** | 0.0156 | -0.0118 | 0.0430 | **m[2L][4R]** | 0.0215 | -0.0177 | 0.0607 |
| **m[4R][1R]** | 0.0153 | -0.0104 | 0.0410 | **m[3L][4R]** | 0.0145 | -0.0108 | 0.0398 |
| **m[5R][1R]** | 0.0148 | -0.0095 | 0.0391 | **m[4L][4R]** | 0.0192 | -0.0182 | 0.0566 |
| **m[6R][1R]** | 0.0164 | -0.0144 | 0.0472 | **m[5L][4R]** | 0.0191 | -0.0130 | 0.0512 |
| **m[7R][1R]** | 0.0154 | -0.0124 | 0.0432 | **m[6L][4R]** | 0.0137 | -0.0104 | 0.0378 |
| **m[1L][1R]** | 0.0142 | -0.0128 | 0.0412 | **m[7L][4R]** | 0.0190 | -0.0147 | 0.0527 |
| **m[2L][1R]** | 0.0187 | -0.0132 | 0.0506 | **m[1R][5R]** | 0.0147 | -0.0102 | 0.0396 |
| **m[3L][1R]** | 0.0168 | -0.0150 | 0.0486 | **m[2R][5R]** | 0.0147 | -0.0141 | 0.0435 |
| **m[4L][1R]** | 0.0201 | -0.0203 | 0.0605 | **m[3R][5R]** | 0.0188 | -0.0177 | 0.0553 |
| **m[5L][1R]** | 0.0192 | -0.0114 | 0.0498 | **m[4R][5R]** | 0.0164 | -0.0138 | 0.0466 |
| **m[6L][1R]** | 0.0150 | -0.0138 | 0.0438 | **m[6R][5R]** | 0.0185 | -0.0199 | 0.0569 |
| **m[7L][1R]** | 0.0164 | -0.0175 | 0.0503 | **m[7R][5R]** | 0.0170 | -0.0161 | 0.0501 |
| **m[1R][2R]** | 0.0144 | -0.0113 | 0.0401 | **m[1L][5R]** | 0.0193 | -0.0150 | 0.0536 |
| **m[3R][2R]** | 0.0158 | -0.0130 | 0.0446 | **m[2L][5R]** | 0.0251 | -0.0176 | 0.0678 |
| **m[4R][2R]** | 0.0176 | -0.0116 | 0.0468 | **m[3L][5R]** | 0.0176 | -0.0138 | 0.0490 |
| **m[5R][2R]** | 0.0188 | -0.0133 | 0.0509 | **m[4L][5R]** | 0.0139 | -0.0112 | 0.0390 |
| **m[6R][2R]** | 0.0181 | -0.0125 | 0.0487 | **m[5L][5R]** | 0.0177 | -0.0154 | 0.0508 |
| **m[7R][2R]** | 0.0165 | -0.0158 | 0.0488 | **m[6L][5R]** | 0.0145 | -0.0125 | 0.0415 |
| **m[1L][2R]** | 0.0155 | -0.0145 | 0.0455 | **m[7L][5R]** | 0.0157 | -0.0172 | 0.0486 |
| **m[2L][2R]** | 0.0134 | -0.0174 | 0.0442 | **m[1R][6R]** | 0.0202 | -0.0196 | 0.0600 |
| **m[3L][2R]** | 0.0171 | -0.0186 | 0.0528 | **m[2R][6R]** | 0.0346 | -0.0160 | 0.0852 |
| **m[4L][2R]** | 0.0175 | -0.0188 | 0.0538 | **m[3R][6R]** | 0.0149 | -0.0145 | 0.0443 |
| **m[5L][2R]** | 0.0156 | -0.0128 | 0.0440 | **m[4R][6R]** | 0.0175 | -0.0162 | 0.0512 |
| **m[6L][2R]** | 0.0170 | -0.0151 | 0.0491 | **m[5R][6R]** | 0.0156 | -0.0109 | 0.0421 |
| **m[7L][2R]** | 0.0153 | -0.0143 | 0.0449 | **m[7R][6R]** | 0.0316 | -0.0135 | 0.0767 |
| **m[1R][3R]** | 0.0180 | -0.0155 | 0.0515 | **m[1L][6R]** | 0.0192 | -0.0186 | 0.0570 |
| **m[2R][3R]** | 0.0166 | -0.0142 | 0.0474 | **m[2L][6R]** | 0.0180 | -0.0198 | 0.0558 |
| **m[4R][3R]** | 0.0174 | -0.0140 | 0.0488 | **m[3L][6R]** | 0.0141 | -0.0108 | 0.0390 |
| **m[5R][3R]** | 0.0154 | -0.0111 | 0.0419 | **m[4L][6R]** | 0.0159 | -0.0162 | 0.0480 |
| **m[6R][3R]** | 0.0144 | -0.0132 | 0.0420 | **m[5L][6R]** | 0.0173 | -0.0194 | 0.0540 |
| **m[7R][3R]** | 0.0169 | -0.0141 | 0.0479 | **m[6L][6R]** | 0.0198 | -0.0165 | 0.0561 |
| **m[1L][3R]** | 0.0175 | -0.0141 | 0.0491 | **m[7L][6R]** | 0.0171 | -0.0135 | 0.0477 |
| **m[2L][3R]** | 0.0155 | -0.0108 | 0.0418 | **m[1R][7R]** | 0.0162 | -0.0114 | 0.0438 |
| **m[3L][3R]** | 0.0163 | -0.0184 | 0.0510 | **m[2R][7R]** | 0.0188 | -0.0141 | 0.0517 |
| **m[4L][3R]** | 0.0171 | -0.0105 | 0.0447 | **m[3R][7R]** | 0.0182 | -0.0147 | 0.0511 |
| **m[5L][3R]** | 0.0155 | -0.0123 | 0.0433 | **m[4R][7R]** | 0.0150 | -0.0144 | 0.0444 |
| **m[6L][3R]** | 0.0190 | -0.0139 | 0.0519 | **m[5R][7R]** | 0.0173 | -0.0205 | 0.0551 |
| **m[7L][3R]** | 0.0171 | -0.0125 | 0.0467 | **m[6R][7R]** | 0.0168 | -0.0124 | 0.0460 |
| **m[1R][4R]** | 0.0162 | -0.0165 | 0.0489 | **m[1L][7R]** | 0.0153 | -0.0125 | 0.0431 |
| **m[2R][4R]** | 0.0308 | -0.0090 | 0.0706 | **m[2L][7R]** | 0.0190 | -0.0159 | 0.0539 |

| **Site pairs** | **m** | **CI95%-** | **CI95%+** | **Site pairs** | **m** | **CI95%-** | **CI95%+** |
| --- | --- | --- | --- | --- | --- | --- | --- |
| **m[3R][4R]** | 0.0138 | -0.0125 | 0.0401 | **m[3L][7R]** | 0.0193 | -0.0152 | 0.0538 |
| **m[5R][4R]** | 0.0132 | -0.0107 | 0.0371 | **m[4L][7R]** | 0.0139 | -0.0090 | 0.0368 |
| **m[6R][4R]** | 0.0160 | -0.0103 | 0.0423 | **m[5L][7R]** | 0.0150 | -0.0124 | 0.0424 |
| **m[7R][4R]** | 0.0159 | -0.0111 | 0.0429 | **m[6L][7R]** | 0.0147 | -0.0141 | 0.0435 |
| **m[7L][7R]** | 0.0190 | -0.0120 | 0.0500 | **m[7R][4L]** | 0.0180 | -0.0161 | 0.0521 |
| **m[1R][1L]** | 0.0179 | -0.0172 | 0.0530 | **m[1L][4L]** | 0.0165 | -0.0129 | 0.0459 |
| **m[2R][1L]** | 0.0384 | -0.0075 | 0.0843 | **m[2L][4L]** | 0.0163 | -0.0143 | 0.0469 |
| **m[3R][1L]** | 0.0168 | -0.0104 | 0.0440 | **m[3L][4L]** | 0.0167 | -0.0131 | 0.0465 |
| **m[4R][1L]** | 0.0185 | -0.0140 | 0.0510 | **m[5L][4L]** | 0.0189 | -0.0209 | 0.0587 |
| **m[5R][1L]** | 0.0177 | -0.0174 | 0.0528 | **m[6L][4L]** | 0.0168 | -0.0148 | 0.0484 |
| **m[6R][1L]** | 0.0149 | -0.0163 | 0.0461 | **m[7L][4L]** | 0.0144 | -0.0117 | 0.0405 |
| **m[7R][1L]** | 0.0166 | -0.0122 | 0.0454 | **m[1R][5L]** | 0.0173 | -0.0137 | 0.0483 |
| **m[2L][1L]** | 0.0245 | -0.0063 | 0.0553 | **m[2R][5L]** | 0.0181 | -0.0162 | 0.0524 |
| **m[3L][1L]** | 0.0231 | -0.0149 | 0.0611 | **m[3R][5L]** | 0.0164 | -0.0163 | 0.0491 |
| **m[4L][1L]** | 0.0159 | -0.0121 | 0.0439 | **m[4R][5L]** | 0.0159 | -0.0159 | 0.0477 |
| **m[5L][1L]** | 0.0179 | -0.0115 | 0.0473 | **m[5R][5L]** | 0.0159 | -0.0119 | 0.0437 |
| **m[6L][1L]** | 0.0340 | -0.0093 | 0.0773 | **m[6R][5L]** | 0.0168 | -0.0132 | 0.0468 |
| **m[7L][1L]** | 0.0150 | -0.0162 | 0.0462 | **m[7R][5L]** | 0.0141 | -0.0143 | 0.0425 |
| **m[1R][2L]** | 0.0176 | -0.0177 | 0.0529 | **m[1L][5L]** | 0.0160 | -0.0167 | 0.0487 |
| **m[2R][2L]** | 0.0157 | -0.0096 | 0.0410 | **m[2L][5L]** | 0.0330 | -0.0095 | 0.0755 |
| **m[3R][2L]** | 0.0162 | -0.0157 | 0.0481 | **m[3L][5L]** | 0.0196 | -0.0155 | 0.0547 |
| **m[4R][2L]** | 0.0172 | -0.0149 | 0.0493 | **m[4L][5L]** | 0.0161 | -0.0084 | 0.0406 |
| **m[5R][2L]** | 0.0187 | -0.0168 | 0.0542 | **m[6L][5L]** | 0.0498 | 0.0047 | 0.0949 |
| **m[6R][2L]** | 0.0159 | -0.0131 | 0.0449 | **m[7L][5L]** | 0.0171 | -0.0141 | 0.0483 |
| **m[7R][2L]** | 0.0164 | -0.0103 | 0.0431 | **m[1R][6L]** | 0.0174 | -0.0132 | 0.0480 |
| **m[1L][2L]** | 0.0139 | -0.0135 | 0.0413 | **m[2R][6L]** | 0.0148 | -0.0095 | 0.0391 |
| **m[3L][2L]** | 0.0166 | -0.0161 | 0.0493 | **m[3R][6L]** | 0.0221 | -0.0226 | 0.0668 |
| **m[4L][2L]** | 0.0118 | -0.0107 | 0.0343 | **m[4R][6L]** | 0.0173 | -0.0139 | 0.0485 |
| **m[5L][2L]** | 0.0145 | -0.0135 | 0.0425 | **m[5R][6L]** | 0.0177 | -0.0205 | 0.0559 |
| **m[6L][2L]** | 0.0186 | -0.0126 | 0.0498 | **m[6R][6L]** | 0.0151 | -0.0125 | 0.0427 |
| **m[7L][2L]** | 0.0166 | -0.0116 | 0.0448 | **m[7R][6L]** | 0.0165 | -0.0127 | 0.0457 |
| **m[1R][3L]** | 0.0173 | -0.0182 | 0.0528 | **m[1L][6L]** | 0.0171 | -0.0172 | 0.0514 |
| **m[2R][3L]** | 0.0326 | -0.0113 | 0.0765 | **m[2L][6L]** | 0.0165 | -0.0127 | 0.0457 |
| **m[3R][3L]** | 0.0162 | -0.0118 | 0.0442 | **m[3L][6L]** | 0.0180 | -0.0126 | 0.0486 |
| **m[4R][3L]** | 0.0189 | -0.0197 | 0.0575 | **m[4L][6L]** | 0.0148 | -0.0121 | 0.0417 |
| **m[5R][3L]** | 0.0321 | -0.0091 | 0.0733 | **m[5L][6L]** | 0.0328 | -0.0097 | 0.0753 |
| **m[6R][3L]** | 0.0199 | -0.0117 | 0.0515 | **m[7L][6L]** | 0.0162 | -0.0126 | 0.0450 |
| **m[7R][3L]** | 0.0504 | -0.0029 | 0.1037 | **m[1R][7L]** | 0.0139 | -0.0114 | 0.0392 |
| **m[1L][3L]** | 0.0173 | -0.0125 | 0.0471 | **m[2R][7L]** | 0.0156 | -0.0138 | 0.0450 |
| **m[2L][3L]** | 0.0153 | -0.0145 | 0.0451 | **m[3R][7L]** | 0.0173 | -0.0139 | 0.0485 |
| **m[4L][3L]** | 0.0169 | -0.0143 | 0.0481 | **m[4R][7L]** | 0.0338 | -0.0130 | 0.0806 |
| **m[5L][3L]** | 0.0323 | -0.0087 | 0.0733 | **m[5R][7L]** | 0.0174 | -0.0130 | 0.0478 |

| **Site pairs** | **m** | **CI95%-** | **CI95%+** | **Site pairs** | **m** | **CI95%-** | **CI95%+** |
| --- | --- | --- | --- | --- | --- | --- | --- |
| **m[6L][3L]** | 0.0521 | 0.0013 | 0.1029 | **m[6R][7L]** | 0.0314 | -0.0064 | 0.0692 |
| **m[7L][3L]** | 0.0171 | -0.0139 | 0.0481 | **m[7R][7L]** | 0.0151 | -0.0118 | 0.0420 |
| **m[1R][4L]** | 0.0163 | -0.0172 | 0.0498 | **m[1L][7L]** | 0.0184 | -0.0161 | 0.0529 |
| **m[2R][4L]** | 0.0180 | -0.0151 | 0.0511 | **m[2L][7L]** | 0.0172 | -0.0144 | 0.0488 |
| **m[3R][4L]** | 0.0161 | -0.0135 | 0.0457 | **m[3L][7L]** | 0.0173 | -0.0145 | 0.0491 |
| **m[4R][4L]** | 0.0162 | -0.0165 | 0.0489 | **m[4L][7L]** | 0.0209 | -0.0181 | 0.0599 |
| **m[5R][4L]** | 0.0178 | -0.0169 | 0.0525 | **m[5L][7L]** | 0.0183 | -0.0166 | 0.0532 |
| **m[6R][4L]** | 0.0157 | -0.0110 | 0.0424 | **m[6L][7L]** | 0.0154 | -0.0115 | 0.0423 |

(C) *Bignonia aequinoctialis*

| **Site pairs** | **m** | **CI95%-** | **CI95%+** | **Site pairs** | **m** | **CI95%-** | **CI95%+** |
| --- | --- | --- | --- | --- | --- | --- | --- |
| **m[2R][1R]** | NA | NA | NA | **m[1L][4R]** | 0.0146 | -0.0121 | 0.0413 |
| **m[3R][1R]** | 0.0172 | -0.0124 | 0.0468 | **m[2L][4R]** | 0.0157 | -0.0129 | 0.0443 |
| **m[4R][1R]** | 0.0162 | -0.0165 | 0.0489 | **m[3L][4R]** | 0.0166 | -0.0150 | 0.0482 |
| **m[5R][1R]** | 0.0173 | -0.0109 | 0.0455 | **m[4L][4R]** | 0.0169 | -0.0158 | 0.0496 |
| **m[6R][1R]** | 0.0184 | -0.0137 | 0.0505 | **m[5L][4R]** | 0.0193 | -0.0177 | 0.0563 |
| **m[7R][1R]** | 0.0122 | -0.0084 | 0.0328 | **m[6L][4R]** | 0.0176 | -0.0177 | 0.0529 |
| **m[1L][1R]** | 0.0188 | -0.0159 | 0.0535 | **m[7L][4R]** | 0.0182 | -0.0139 | 0.0503 |
| **m[2L][1R]** | 0.0186 | -0.0184 | 0.0556 | **m[1R][5R]** | 0.0172 | -0.0128 | 0.0472 |
| **m[3L][1R]** | 0.0140 | -0.0136 | 0.0416 | **m[2R][5R]** | NA | NA | NA |
| **m[4L][1R]** | 0.0160 | -0.0191 | 0.0511 | **m[3R][5R]** | 0.0186 | -0.0141 | 0.0513 |
| **m[5L][1R]** | 0.0161 | -0.0121 | 0.0443 | **m[4R][5R]** | 0.0180 | -0.0130 | 0.0490 |
| **m[6L][1R]** | 0.0175 | -0.0141 | 0.0491 | **m[6R][5R]** | 0.0158 | -0.0097 | 0.0413 |
| **m[7L][1R]** | 0.0193 | -0.0125 | 0.0511 | **m[7R][5R]** | 0.0191 | -0.0160 | 0.0542 |
| **m[1R][2R]** | NA | NA | NA | **m[1L][5R]** | 0.0138 | -0.0117 | 0.0393 |
| **m[3R][2R]** | NA | NA | NA | **m[2L][5R]** | 0.0164 | -0.0163 | 0.0491 |
| **m[4R][2R]** | NA | NA | NA | **m[3L][5R]** | 0.0200 | -0.0149 | 0.0549 |
| **m[5R][2R]** | NA | NA | NA | **m[4L][5R]** | 0.0151 | -0.0143 | 0.0445 |
| **m[6R][2R]** | NA | NA | NA | **m[5L][5R]** | 0.0142 | -0.0097 | 0.0381 |
| **m[7R][2R]** | NA | NA | NA | **m[6L][5R]** | 0.0158 | -0.0171 | 0.0487 |
| **m[1L][2R]** | NA | NA | NA | **m[7L][5R]** | 0.0171 | -0.0154 | 0.0496 |
| **m[2L][2R]** | NA | NA | NA | **m[1R][6R]** | 0.0166 | -0.0155 | 0.0487 |
| **m[3L][2R]** | NA | NA | NA | **m[2R][6R]** | NA | NA | NA |
| **m[4L][2R]** | NA | NA | NA | **m[3R][6R]** | 0.0166 | -0.0104 | 0.0436 |
| **m[5L][2R]** | NA | NA | NA | **m[4R][6R]** | 0.0174 | -0.0149 | 0.0497 |
| **m[6L][2R]** | NA | NA | NA | **m[5R][6R]** | 0.0200 | -0.0176 | 0.0576 |
| **m[7L][2R]** | NA | NA | NA | **m[7R][6R]** | 0.0156 | -0.0118 | 0.0430 |
| **m[1R][3R]** | 0.0175 | -0.0082 | 0.0432 | **m[1L][6R]** | 0.0166 | -0.0159 | 0.0491 |
| **m[2R][3R]** | NA | NA | NA | **m[2L][6R]** | 0.0140 | -0.0117 | 0.0397 |
| **m[4R][3R]** | 0.0160 | -0.0163 | 0.0483 | **m[3L][6R]** | 0.0163 | -0.0164 | 0.0490 |
| **m[5R][3R]** | 0.0164 | -0.0157 | 0.0485 | **m[4L][6R]** | 0.0156 | -0.0130 | 0.0442 |
| **m[6R][3R]** | 0.0162 | -0.0118 | 0.0442 | **m[5L][6R]** | 0.0186 | -0.0106 | 0.0478 |
| **m[7R][3R]** | 0.0161 | -0.0164 | 0.0486 | **m[6L][6R]** | 0.0134 | -0.0105 | 0.0373 |
| **m[1L][3R]** | 0.0183 | -0.0236 | 0.0602 | **m[7L][6R]** | 0.0180 | -0.0149 | 0.0509 |
| **m[2L][3R]** | 0.0184 | -0.0128 | 0.0496 | **m[1R][7R]** | 0.0158 | -0.0150 | 0.0466 |
| **m[3L][3R]** | 0.0201 | -0.0162 | 0.0564 | **m[2R][7R]** | NA | NA | NA |
| **m[4L][3R]** | 0.0148 | -0.0177 | 0.0473 | **m[3R][7R]** | 0.0163 | -0.0100 | 0.0426 |
| **m[5L][3R]** | 0.0184 | -0.0108 | 0.0476 | **m[4R][7R]** | 0.0132 | -0.0137 | 0.0401 |
| **m[6L][3R]** | 0.0168 | -0.0128 | 0.0464 | **m[5R][7R]** | 0.0177 | -0.0172 | 0.0526 |
| **m[7L][3R]** | 0.0168 | -0.0138 | 0.0474 | **m[6R][7R]** | 0.0166 | -0.0128 | 0.0460 |
| **m[1R][4R]** | 0.0173 | -0.0133 | 0.0479 | **m[1L][7R]** | 0.0198 | -0.0116 | 0.0512 |
| **m[2R][4R]** | NA | NA | NA | **m[2L][7R]** | 0.0174 | -0.0106 | 0.0454 |

| **Site pairs** | **m** | **CI95%-** | **CI95%+** | **Site pairs** | **m** | **CI95%-** | **CI95%+** |
| --- | --- | --- | --- | --- | --- | --- | --- |
| **m[3R][4R]** | 0.0149 | -0.0100 | 0.0398 | **m[3L][7R]** | 0.0138 | -0.0134 | 0.0410 |
| **m[5R][4R]** | 0.0170 | -0.0187 | 0.0527 | **m[4L][7R]** | 0.0181 | -0.0125 | 0.0487 |
| **m[6R][4R]** | 0.0166 | -0.0181 | 0.0513 | **m[5L][7R]** | 0.0173 | -0.0131 | 0.0477 |
| **m[7R][4R]** | 0.0150 | -0.0130 | 0.0430 | **m[6L][7R]** | 0.0187 | -0.0091 | 0.0465 |
| **m[7L][7R]** | 0.0139 | -0.0116 | 0.0394 | **m[7R][4L]** | 0.1104 | 0.0316 | 0.1892 |
| **m[1R][1L]** | 0.0137 | -0.0155 | 0.0429 | **m[1L][4L]** | 0.1118 | 0.0516 | 0.1720 |
| **m[2R][1L]** | NA | NA | NA | **m[2L][4L]** | **0.1165** | **0.0538** | **0.1792** |
| **m[3R][1L]** | 0.0173 | -0.0074 | 0.0420 | **m[3L][4L]** | **0.1181** | **0.0507** | **0.1855** |
| **m[4R][1L]** | 0.0159 | -0.0166 | 0.0484 | **m[5L][4L]** | 0.0964 | 0.0319 | 0.1609 |
| **m[5R][1L]** | 0.0168 | -0.0136 | 0.0472 | **m[6L][4L]** | 0.1004 | 0.0340 | 0.1668 |
| **m[6R][1L]** | 0.0166 | -0.0191 | 0.0523 | **m[7L][4L]** | 0.1163 | 0.0424 | 0.1902 |
| **m[7R][1L]** | 0.0176 | -0.0149 | 0.0501 | **m[1R][5L]** | 0.0169 | -0.0180 | 0.0518 |
| **m[2L][1L]** | 0.0162 | -0.0134 | 0.0458 | **m[2R][5L]** | NA | NA | NA |
| **m[3L][1L]** | 0.0156 | -0.0163 | 0.0475 | **m[3R][5L]** | 0.0161 | -0.0086 | 0.0408 |
| **m[4L][1L]** | 0.0187 | -0.0215 | 0.0589 | **m[4R][5L]** | 0.0182 | -0.0249 | 0.0613 |
| **m[5L][1L]** | 0.0156 | -0.0156 | 0.0468 | **m[5R][5L]** | 0.0168 | -0.0189 | 0.0525 |
| **m[6L][1L]** | 0.0168 | -0.0148 | 0.0484 | **m[6R][5L]** | 0.0160 | -0.0152 | 0.0472 |
| **m[7L][1L]** | 0.0152 | -0.0144 | 0.0448 | **m[7R][5L]** | 0.0173 | -0.0174 | 0.0520 |
| **m[1R][2L]** | 0.0164 | -0.0163 | 0.0491 | **m[1L][5L]** | 0.0179 | -0.0162 | 0.0520 |
| **m[2R][2L]** | NA | NA | NA | **m[2L][5L]** | 0.0178 | -0.0161 | 0.0517 |
| **m[3R][2L]** | 0.0163 | -0.0106 | 0.0432 | **m[3L][5L]** | 0.0157 | -0.0098 | 0.0412 |
| **m[4R][2L]** | 0.0176 | -0.0138 | 0.0490 | **m[4L][5L]** | 0.0180 | -0.0149 | 0.0509 |
| **m[5R][2L]** | 0.0162 | -0.0114 | 0.0438 | **m[6L][5L]** | 0.0173 | -0.0105 | 0.0451 |
| **m[6R][2L]** | 0.0165 | -0.0147 | 0.0477 | **m[7L][5L]** | 0.0157 | -0.0094 | 0.0408 |
| **m[7R][2L]** | 0.0149 | -0.0123 | 0.0421 | **m[1R][6L]** | 0.0128 | -0.0142 | 0.0398 |
| **m[1L][2L]** | 0.0164 | -0.0128 | 0.0456 | **m[2R][6L]** | NA | NA | NA |
| **m[3L][2L]** | 0.0193 | -0.0164 | 0.0550 | **m[3R][6L]** | 0.0195 | -0.0152 | 0.0542 |
| **m[4L][2L]** | **0.0162** | **-0.0099** | **0.0423** | **m[4R][6L]** | 0.0148 | -0.0079 | 0.0375 |
| **m[5L][2L]** | 0.0146 | -0.0140 | 0.0432 | **m[5R][6L]** | 0.0159 | -0.0127 | 0.0445 |
| **m[6L][2L]** | 0.0165 | -0.0141 | 0.0471 | **m[6R][6L]** | 0.0154 | -0.0142 | 0.0450 |
| **m[7L][2L]** | 0.0155 | -0.0115 | 0.0425 | **m[7R][6L]** | 0.0145 | -0.0133 | 0.0423 |
| **m[1R][3L]** | 0.0171 | -0.0174 | 0.0516 | **m[1L][6L]** | 0.0197 | -0.0144 | 0.0538 |
| **m[2R][3L]** | NA | NA | NA | **m[2L][6L]** | 0.0161 | -0.0102 | 0.0424 |
| **m[3R][3L]** | 0.0163 | -0.0156 | 0.0482 | **m[3L][6L]** | 0.0152 | -0.0120 | 0.0424 |
| **m[4R][3L]** | 0.0150 | -0.0138 | 0.0438 | **m[4L][6L]** | 0.0152 | -0.0095 | 0.0399 |
| **m[5R][3L]** | 0.0185 | -0.0166 | 0.0536 | **m[5L][6L]** | 0.0158 | -0.0120 | 0.0436 |
| **m[6R][3L]** | 0.0171 | -0.0096 | 0.0438 | **m[7L][6L]** | 0.0137 | -0.0106 | 0.0380 |
| **m[7R][3L]** | 0.0153 | -0.0141 | 0.0447 | **m[1R][7L]** | 0.0149 | -0.0167 | 0.0465 |
| **m[1L][3L]** | 0.0153 | -0.0155 | 0.0461 | **m[2R][7L]** | NA | NA | NA |
| **m[2L][3L]** | 0.0167 | -0.0152 | 0.0486 | **m[3R][7L]** | 0.0187 | -0.0170 | 0.0544 |
| **m[4L][3L]** | **0.0137** | **-0.0092** | **0.0366** | **m[4R][7L]** | 0.0180 | -0.0149 | 0.0509 |
| **m[5L][3L]** | 0.0177 | -0.0152 | 0.0506 | **m[5R][7L]** | 0.0188 | -0.0165 | 0.0541 |

| **Site pairs** | **m** | **CI95%-** | **CI95%+** | **Site pairs** | **m** | **CI95%-** | **CI95%+** |
| --- | --- | --- | --- | --- | --- | --- | --- |
| **m[6L][3L]** | 0.0158 | -0.0126 | 0.0442 | **m[6R][7L]** | 0.0150 | -0.0132 | 0.0432 |
| **m[7L][3L]** | 0.0166 | -0.0148 | 0.0480 | **m[7R][7L]** | 0.0133 | -0.0087 | 0.0353 |
| **m[1R][4L]** | 0.1219 | 0.0539 | 0.1899 | **m[1L][7L]** | 0.0151 | -0.0149 | 0.0451 |
| **m[2R][4L]** | NA | NA | NA | **m[2L][7L]** | 0.0163 | -0.0133 | 0.0459 |
| **m[3R][4L]** | 0.0824 | 0.0218 | 0.1430 | **m[3L][7L]** | 0.0176 | -0.0173 | 0.0525 |
| **m[4R][4L]** | 0.0873 | 0.0299 | 0.1447 | **m[4L][7L]** | 0.0188 | -0.0143 | 0.0519 |
| **m[5R][4L]** | 0.0980 | 0.0351 | 0.1609 | **m[5L][7L]** | 0.0168 | -0.0144 | 0.0480 |
| **m[6R][4L]** | 0.1199 | 0.0540 | 0.1858 | **m[6L][7L]** | 0.0162 | -0.0157 | 0.0481 |

(D) *Pachyptera kerere*

| **Site pairs** | **m** | **CI95%-** | **CI95%+** | **Site pairs** | **m** | **CI95%-** | **CI95%+** |
| --- | --- | --- | --- | --- | --- | --- | --- |
| **m[2R][1R]** | 0.0254 | -0.0212 | 0.0720 | **m[1L][4R]** | 0.0164 | -0.0132 | 0.0460 |
| **m[3R][1R]** | 0.0172 | -0.0120 | 0.0464 | **m[2L][4R]** | 0.0154 | -0.0132 | 0.0440 |
| **m[4R][1R]** | 0.0148 | -0.0115 | 0.0411 | **m[3L][4R]** | 0.0360 | -0.0093 | 0.0813 |
| **m[5R][1R]** | 0.0161 | -0.0170 | 0.0492 | **m[4L][4R]** | 0.0159 | -0.0127 | 0.0445 |
| **m[6R][1R]** | 0.0181 | -0.0176 | 0.0538 | **m[5L][4R]** | 0.0252 | -0.0146 | 0.0650 |
| **m[7R][1R]** | 0.0321 | -0.0106 | 0.0748 | **m[6L][4R]** | 0.0179 | -0.0195 | 0.0553 |
| **m[1L][1R]** | 0.0199 | -0.0128 | 0.0526 | **m[7L][4R]** | 0.0195 | -0.0119 | 0.0509 |
| **m[2L][1R]** | 0.0160 | -0.0107 | 0.0427 | **m[1R][5R]** | 0.0170 | -0.0167 | 0.0507 |
| **m[3L][1R]** | 0.0164 | -0.0120 | 0.0448 | **m[2R][5R]** | 0.0182 | -0.0173 | 0.0537 |
| **m[4L][1R]** | 0.0337 | -0.0124 | 0.0798 | **m[3R][5R]** | 0.0161 | -0.0109 | 0.0431 |
| **m[5L][1R]** | 0.0150 | -0.0101 | 0.0401 | **m[4R][5R]** | 0.0165 | -0.0131 | 0.0461 |
| **m[6L][1R]** | 0.0171 | -0.0150 | 0.0492 | **m[6R][5R]** | 0.0145 | -0.0118 | 0.0408 |
| **m[7L][1R]** | 0.0147 | -0.0084 | 0.0378 | **m[7R][5R]** | 0.0158 | -0.0152 | 0.0468 |
| **m[1R][2R]** | 0.0188 | -0.0163 | 0.0539 | **m[1L][5R]** | 0.0167 | -0.0203 | 0.0537 |
| **m[3R][2R]** | 0.0187 | -0.0134 | 0.0508 | **m[2L][5R]** | 0.0182 | -0.0173 | 0.0537 |
| **m[4R][2R]** | 0.0175 | -0.0164 | 0.0514 | **m[3L][5R]** | 0.0160 | -0.0154 | 0.0474 |
| **m[5R][2R]** | 0.0164 | -0.0128 | 0.0456 | **m[4L][5R]** | 0.0302 | -0.0084 | 0.0688 |
| **m[6R][2R]** | 0.0170 | -0.0142 | 0.0482 | **m[5L][5R]** | 0.0193 | -0.0156 | 0.0542 |
| **m[7R][2R]** | 0.0171 | -0.0141 | 0.0483 | **m[6L][5R]** | 0.0156 | -0.0136 | 0.0448 |
| **m[1L][2R]** | 0.0324 | -0.0025 | 0.0673 | **m[7L][5R]** | 0.0316 | -0.0015 | 0.0647 |
| **m[2L][2R]** | 0.0192 | -0.0106 | 0.0490 | **m[1R][6R]** | 0.0169 | -0.0125 | 0.0463 |
| **m[3L][2R]** | 0.0170 | -0.0148 | 0.0488 | **m[2R][6R]** | 0.0194 | -0.0157 | 0.0545 |
| **m[4L][2R]** | 0.0164 | -0.0126 | 0.0454 | **m[3R][6R]** | 0.0161 | -0.0157 | 0.0479 |
| **m[5L][2R]** | 0.0143 | -0.0120 | 0.0406 | **m[4R][6R]** | 0.0176 | -0.0196 | 0.0548 |
| **m[6L][2R]** | 0.0164 | -0.0095 | 0.0423 | **m[5R][6R]** | 0.0158 | -0.0181 | 0.0497 |
| **m[7L][2R]** | 0.0153 | -0.0159 | 0.0465 | **m[7R][6R]** | 0.0177 | -0.0119 | 0.0473 |
| **m[1R][3R]** | 0.0131 | -0.0071 | 0.0333 | **m[1L][6R]** | 0.0152 | -0.0117 | 0.0421 |
| **m[2R][3R]** | 0.0163 | -0.0141 | 0.0467 | **m[2L][6R]** | 0.0173 | -0.0176 | 0.0522 |
| **m[4R][3R]** | 0.0170 | -0.0116 | 0.0456 | **m[3L][6R]** | 0.0166 | -0.0114 | 0.0446 |
| **m[5R][3R]** | 0.0183 | -0.0182 | 0.0548 | **m[4L][6R]** | 0.0158 | -0.0167 | 0.0483 |
| **m[6R][3R]** | 0.0175 | -0.0154 | 0.0504 | **m[5L][6R]** | 0.0147 | -0.0086 | 0.0380 |
| **m[7R][3R]** | 0.0159 | -0.0190 | 0.0508 | **m[6L][6R]** | 0.0152 | -0.0128 | 0.0432 |
| **m[1L][3R]** | 0.0175 | -0.0182 | 0.0532 | **m[7L][6R]** | 0.0174 | -0.0163 | 0.0511 |
| **m[2L][3R]** | 0.0142 | -0.0136 | 0.0420 | **m[1R][7R]** | 0.0365 | -0.0064 | 0.0794 |
| **m[3L][3R]** | 0.0339 | -0.0175 | 0.0853 | **m[2R][7R]** | 0.0166 | -0.0130 | 0.0462 |
| **m[4L][3R]** | 0.0202 | -0.0137 | 0.0541 | **m[3R][7R]** | 0.0187 | -0.0131 | 0.0505 |
| **m[5L][3R]** | 0.0177 | -0.0180 | 0.0534 | **m[4R][7R]** | 0.0174 | -0.0140 | 0.0488 |
| **m[6L][3R]** | 0.0192 | -0.0182 | 0.0566 | **m[5R][7R]** | 0.0167 | -0.0162 | 0.0496 |
| **m[7L][3R]** | 0.0175 | -0.0125 | 0.0475 | **m[6R][7R]** | 0.0178 | -0.0185 | 0.0541 |
| **m[1R][4R]** | 0.0196 | -0.0194 | 0.0586 | **m[1L][7R]** | 0.0166 | -0.0150 | 0.0482 |
| **m[2R][4R]** | 0.0156 | -0.0146 | 0.0458 | **m[2L][7R]** | 0.0147 | -0.0116 | 0.0410 |

| **Site pairs** | **m** | **CI95%-** | **CI95%+** | **Site pairs** | **m** | **CI95%-** | **CI95%+** |
| --- | --- | --- | --- | --- | --- | --- | --- |
| **m[3R][4R]** | 0.0150 | -0.0087 | 0.0387 | **m[3L][7R]** | 0.0196 | -0.0167 | 0.0559 |
| **m[5R][4R]** | 0.0160 | -0.0136 | 0.0456 | **m[4L][7R]** | 0.0194 | -0.0159 | 0.0547 |
| **m[6R][4R]** | 0.0207 | -0.0183 | 0.0597 | **m[5L][7R]** | 0.0185 | -0.0160 | 0.0530 |
| **m[7R][4R]** | 0.0180 | -0.0128 | 0.0488 | **m[6L][7R]** | 0.0158 | -0.0103 | 0.0419 |
| **m[7L][7R]** | 0.0140 | -0.0121 | 0.0401 | **m[7R][4L]** | 0.0175 | -0.0199 | 0.0549 |
| **m[1R][1L]** | 0.0148 | -0.0132 | 0.0428 | **m[1L][4L]** | 0.0183 | -0.0144 | 0.0510 |
| **m[2R][1L]** | 0.0358 | -0.0087 | 0.0803 | **m[2L][4L]** | 0.0186 | -0.0165 | 0.0537 |
| **m[3R][1L]** | 0.0147 | -0.0141 | 0.0435 | **m[3L][4L]** | 0.0139 | -0.0155 | 0.0433 |
| **m[4R][1L]** | 0.0166 | -0.0108 | 0.0440 | **m[5L][4L]** | 0.0164 | -0.0118 | 0.0446 |
| **m[5R][1L]** | 0.0185 | -0.0127 | 0.0497 | **m[6L][4L]** | 0.0152 | -0.0126 | 0.0430 |
| **m[6R][1L]** | 0.0347 | -0.0108 | 0.0802 | **m[7L][4L]** | 0.0161 | -0.0184 | 0.0506 |
| **m[7R][1L]** | 0.0352 | -0.0073 | 0.0777 | **m[1R][5L]** | 0.0128 | -0.0139 | 0.0395 |
| **m[2L][1L]** | 0.0185 | -0.0168 | 0.0538 | **m[2R][5L]** | 0.0152 | -0.0166 | 0.0470 |
| **m[3L][1L]** | 0.0139 | -0.0137 | 0.0415 | **m[3R][5L]** | 0.0169 | -0.0109 | 0.0447 |
| **m[4L][1L]** | 0.0318 | -0.0060 | 0.0696 | **m[4R][5L]** | 0.0146 | -0.0130 | 0.0422 |
| **m[5L][1L]** | 0.0170 | -0.0106 | 0.0446 | **m[5R][5L]** | 0.0166 | -0.0155 | 0.0487 |
| **m[6L][1L]** | 0.0172 | -0.0179 | 0.0523 | **m[6R][5L]** | 0.0305 | -0.0056 | 0.0666 |
| **m[7L][1L]** | 0.0185 | -0.0148 | 0.0518 | **m[7R][5L]** | 0.0174 | -0.0193 | 0.0541 |
| **m[1R][2L]** | 0.0166 | -0.0165 | 0.0497 | **m[1L][5L]** | 0.0162 | -0.0169 | 0.0493 |
| **m[2R][2L]** | 0.0328 | -0.0072 | 0.0728 | **m[2L][5L]** | 0.0152 | -0.0089 | 0.0393 |
| **m[3R][2L]** | 0.0159 | -0.0149 | 0.0467 | **m[3L][5L]** | 0.0172 | -0.0183 | 0.0527 |
| **m[4R][2L]** | 0.0168 | -0.0153 | 0.0489 | **m[4L][5L]** | 0.0155 | -0.0145 | 0.0455 |
| **m[5R][2L]** | 0.0174 | -0.0163 | 0.0511 | **m[6L][5L]** | 0.0173 | -0.0107 | 0.0453 |
| **m[6R][2L]** | 0.0169 | -0.0121 | 0.0459 | **m[7L][5L]** | 0.0179 | -0.0144 | 0.0502 |
| **m[7R][2L]** | 0.0197 | -0.0113 | 0.0507 | **m[1R][6L]** | 0.0182 | -0.0165 | 0.0529 |
| **m[1L][2L]** | 0.0150 | -0.0173 | 0.0473 | **m[2R][6L]** | 0.0144 | -0.0138 | 0.0426 |
| **m[3L][2L]** | 0.0308 | -0.0086 | 0.0702 | **m[3R][6L]** | 0.0379 | -0.0117 | 0.0875 |
| **m[4L][2L]** | 0.0204 | -0.0208 | 0.0616 | **m[4R][6L]** | 0.0170 | -0.0151 | 0.0491 |
| **m[5L][2L]** | 0.0182 | -0.0157 | 0.0521 | **m[5R][6L]** | 0.0174 | -0.0108 | 0.0456 |
| **m[6L][2L]** | 0.0148 | -0.0103 | 0.0399 | **m[6R][6L]** | 0.0153 | -0.0114 | 0.0420 |
| **m[7L][2L]** | 0.0182 | -0.0177 | 0.0541 | **m[7R][6L]** | 0.0157 | -0.0117 | 0.0431 |
| **m[1R][3L]** | 0.0263 | -0.0164 | 0.0690 | **m[1L][6L]** | 0.0190 | -0.0120 | 0.0500 |
| **m[2R][3L]** | 0.0136 | -0.0111 | 0.0383 | **m[2L][6L]** | 0.0189 | -0.0146 | 0.0524 |
| **m[3R][3L]** | 0.0346 | -0.0026 | 0.0718 | **m[3L][6L]** | 0.0167 | -0.0149 | 0.0483 |
| **m[4R][3L]** | 0.0179 | -0.0209 | 0.0567 | **m[4L][6L]** | 0.0141 | -0.0155 | 0.0437 |
| **m[5R][3L]** | 0.0160 | -0.0120 | 0.0440 | **m[5L][6L]** | 0.0171 | -0.0129 | 0.0471 |
| **m[6R][3L]** | 0.0149 | -0.0086 | 0.0384 | **m[7L][6L]** | 0.0182 | -0.0165 | 0.0529 |
| **m[7R][3L]** | 0.0339 | -0.0078 | 0.0756 | **m[1R][7L]** | 0.0158 | -0.0099 | 0.0415 |
| **m[1L][3L]** | 0.0281 | -0.0156 | 0.0718 | **m[2R][7L]** | 0.0156 | -0.0099 | 0.0411 |
| **m[2L][3L]** | 0.0175 | -0.0164 | 0.0514 | **m[3R][7L]** | 0.0144 | -0.0121 | 0.0409 |
| **m[4L][3L]** | 0.0364 | -0.0136 | 0.0864 | **m[4R][7L]** | 0.0305 | -0.0081 | 0.0691 |
| **m[5L][3L]** | 0.0185 | -0.0166 | 0.0536 | **m[5R][7L]** | 0.0168 | -0.0155 | 0.0491 |

| **Site pairs** | **m** | **CI95%-** | **CI95%+** | **Site pairs** | **m** | **CI95%-** | **CI95%+** |
| --- | --- | --- | --- | --- | --- | --- | --- |
| **m[6L][3L]** | 0.0169 | -0.0180 | 0.0518 | **m[6R][7L]** | 0.0177 | -0.0180 | 0.0534 |
| **m[7L][3L]** | 0.0157 | -0.0125 | 0.0439 | **m[7R][7L]** | 0.0165 | -0.0115 | 0.0445 |
| **m[1R][4L]** | 0.0165 | -0.0156 | 0.0486 | **m[1L][7L]** | 0.0142 | -0.0101 | 0.0385 |
| **m[2R][4L]** | 0.0159 | -0.0147 | 0.0465 | **m[2L][7L]** | 0.0362 | -0.0099 | 0.0823 |
| **m[3R][4L]** | 0.0165 | -0.0127 | 0.0457 | **m[3L][7L]** | 0.0200 | -0.0118 | 0.0518 |
| **m[4R][4L]** | 0.0189 | -0.0179 | 0.0557 | **m[4L][7L]** | 0.0149 | -0.0133 | 0.0431 |
| **m[5R][4L]** | 0.0195 | -0.0160 | 0.0550 | **m[5L][7L]** | 0.0141 | -0.0124 | 0.0406 |
| **m[6R][4L]** | 0.0152 | -0.0144 | 0.0448 | **m[6L][7L]** | 0.0149 | -0.0096 | 0.0394 |

(E) *Tanaecium pyramidatum*

| **Site pairs** | **m** | **CI95%-** | **CI95%+** | **Site pairs** | **m** | **CI95%-** | **CI95%+** |
| --- | --- | --- | --- | --- | --- | --- | --- |
| **m[2R][1R]** | 0.0151 | -0.0125 | 0.0427 | **m[1L][4R]** | NA | NA | NA |
| **m[3R][1R]** | 0.0182 | -0.0106 | 0.0470 | **m[2L][4R]** | NA | NA | NA |
| **m[4R][1R]** | NA | NA | NA | **m[3L][4R]** | NA | NA | NA |
| **m[5R][1R]** | 0.0168 | -0.0150 | 0.0486 | **m[4L][4R]** | NA | NA | NA |
| **m[6R][1R]** | 0.0175 | -0.0154 | 0.0504 | **m[5L][4R]** | NA | NA | NA |
| **m[7R][1R]** | 0.0169 | -0.0188 | 0.0526 | **m[6L][4R]** | NA | NA | NA |
| **m[1L][1R]** | 0.0191 | -0.0209 | 0.0591 | **m[7L][4R]** | NA | NA | NA |
| **m[2L][1R]** | 0.0158 | -0.0103 | 0.0419 | **m[1R][5R]** | 0.0168 | -0.0116 | 0.0452 |
| **m[3L][1R]** | 0.0189 | -0.0183 | 0.0561 | **m[2R][5R]** | 0.0144 | -0.0138 | 0.0426 |
| **m[4L][1R]** | 0.0150 | -0.0120 | 0.0420 | **m[3R][5R]** | 0.0173 | -0.0170 | 0.0516 |
| **m[5L][1R]** | 0.0169 | -0.0139 | 0.0477 | **m[4R][5R]** | NA | NA | NA |
| **m[6L][1R]** | 0.0182 | -0.0147 | 0.0511 | **m[6R][5R]** | 0.0165 | -0.0137 | 0.0467 |
| **m[7L][1R]** | 0.0161 | -0.0160 | 0.0482 | **m[7R][5R]** | 0.0168 | -0.0128 | 0.0464 |
| **m[1R][2R]** | 0.0154 | -0.0146 | 0.0454 | **m[1L][5R]** | 0.0139 | -0.0167 | 0.0445 |
| **m[3R][2R]** | 0.0166 | -0.0152 | 0.0484 | **m[2L][5R]** | 0.0163 | -0.0151 | 0.0477 |
| **m[4R][2R]** | NA | NA | NA | **m[3L][5R]** | 0.0167 | -0.0182 | 0.0516 |
| **m[5R][2R]** | 0.0143 | -0.0116 | 0.0402 | **m[4L][5R]** | 0.0162 | -0.0136 | 0.0460 |
| **m[6R][2R]** | 0.0154 | -0.0130 | 0.0438 | **m[5L][5R]** | 0.0198 | -0.0178 | 0.0574 |
| **m[7R][2R]** | 0.0160 | -0.0116 | 0.0436 | **m[6L][5R]** | 0.0129 | -0.0108 | 0.0366 |
| **m[1L][2R]** | 0.0168 | -0.0148 | 0.0484 | **m[7L][5R]** | 0.0176 | -0.0151 | 0.0503 |
| **m[2L][2R]** | 0.0191 | -0.0156 | 0.0538 | **m[1R][6R]** | 0.0153 | -0.0092 | 0.0398 |
| **m[3L][2R]** | 0.0168 | -0.0161 | 0.0497 | **m[2R][6R]** | 0.0165 | -0.0143 | 0.0473 |
| **m[4L][2R]** | 0.0148 | -0.0156 | 0.0452 | **m[3R][6R]** | 0.0159 | -0.0123 | 0.0441 |
| **m[5L][2R]** | 0.0143 | -0.0122 | 0.0408 | **m[4R][6R]** | NA | NA | NA |
| **m[6L][2R]** | 0.0161 | -0.0088 | 0.0410 | **m[5R][6R]** | 0.0154 | -0.0181 | 0.0489 |
| **m[7L][2R]** | 0.0157 | -0.0110 | 0.0424 | **m[7R][6R]** | 0.0135 | -0.0118 | 0.0388 |
| **m[1R][3R]** | 0.0155 | -0.0096 | 0.0406 | **m[1L][6R]** | 0.0178 | -0.0153 | 0.0509 |
| **m[2R][3R]** | 0.0177 | -0.0195 | 0.0549 | **m[2L][6R]** | 0.0199 | -0.0179 | 0.0577 |
| **m[4R][3R]** | NA | NA | NA | **m[3L][6R]** | 0.0150 | -0.0107 | 0.0407 |
| **m[5R][3R]** | 0.0170 | -0.0114 | 0.0454 | **m[4L][6R]** | 0.0186 | -0.0132 | 0.0504 |
| **m[6R][3R]** | 0.0175 | -0.0170 | 0.0520 | **m[5L][6R]** | 0.0162 | -0.0140 | 0.0464 |
| **m[7R][3R]** | 0.0152 | -0.0150 | 0.0454 | **m[6L][6R]** | 0.0171 | -0.0170 | 0.0512 |
| **m[1L][3R]** | 0.0174 | -0.0142 | 0.0490 | **m[7L][6R]** | 0.0155 | -0.0127 | 0.0437 |
| **m[2L][3R]** | 0.0143 | -0.0137 | 0.0423 | **m[1R][7R]** | 0.0175 | -0.0174 | 0.0524 |
| **m[3L][3R]** | 0.0180 | -0.0187 | 0.0547 | **m[2R][7R]** | 0.0181 | -0.0135 | 0.0497 |
| **m[4L][3R]** | 0.0169 | -0.0101 | 0.0439 | **m[3R][7R]** | 0.0168 | -0.0126 | 0.0462 |
| **m[5L][3R]** | 0.0175 | -0.0131 | 0.0481 | **m[4R][7R]** | NA | NA | NA |
| **m[6L][3R]** | 0.0167 | -0.0145 | 0.0479 | **m[5R][7R]** | 0.0176 | -0.0138 | 0.0490 |
| **m[7L][3R]** | 0.0158 | -0.0140 | 0.0456 | **m[6R][7R]** | 0.0490 | 0.0035 | 0.0945 |
| **m[1R][4R]** | NA | NA | NA | **m[1L][7R]** | 0.0132 | -0.0152 | 0.0416 |
| **m[2R][4R]** | NA | NA | NA | **m[2L][7R]** | 0.0151 | -0.0133 | 0.0435 |

| **Site pairs** | **m** | **CI95%-** | **CI95%+** | **Site pairs** | **m** | **CI95%-** | **CI95%+** |
| --- | --- | --- | --- | --- | --- | --- | --- |
| **m[3R][4R]** | NA | NA | NA | **m[3L][7R]** | 0.0158 | -0.0158 | 0.0474 |
| **m[5R][4R]** | NA | NA | NA | **m[4L][7R]** | 0.0172 | -0.0142 | 0.0486 |
| **m[6R][4R]** | NA | NA | NA | **m[5L][7R]** | 0.0328 | -0.0133 | 0.0789 |
| **m[7R][4R]** | NA | NA | NA | **m[6L][7R]** | 0.0180 | -0.0161 | 0.0521 |
| **m[7L][7R]** | 0.0171 | -0.0117 | 0.0459 | **m[7R][4L]** | 0.0180 | -0.0236 | 0.0596 |
| **m[1R][1L]** | 0.0161 | -0.0145 | 0.0467 | **m[1L][4L]** | 0.0183 | -0.0178 | 0.0544 |
| **m[2R][1L]** | 0.0329 | -0.0151 | 0.0809 | **m[2L][4L]** | 0.0146 | -0.0152 | 0.0444 |
| **m[3R][1L]** | 0.0189 | -0.0197 | 0.0575 | **m[3L][4L]** | 0.0186 | -0.0139 | 0.0511 |
| **m[4R][1L]** | NA | NA | NA | **m[5L][4L]** | 0.0152 | -0.0148 | 0.0452 |
| **m[5R][1L]** | 0.0154 | -0.0128 | 0.0436 | **m[6L][4L]** | 0.0193 | -0.0205 | 0.0591 |
| **m[6R][1L]** | 0.0170 | -0.0122 | 0.0462 | **m[7L][4L]** | 0.0173 | -0.0248 | 0.0594 |
| **m[7R][1L]** | 0.0183 | -0.0111 | 0.0477 | **m[1R][5L]** | 0.0198 | -0.0163 | 0.0559 |
| **m[2L][1L]** | 0.0170 | -0.0124 | 0.0464 | **m[2R][5L]** | 0.0167 | -0.0145 | 0.0479 |
| **m[3L][1L]** | 0.0177 | -0.0125 | 0.0479 | **m[3R][5L]** | 0.0194 | -0.0188 | 0.0576 |
| **m[4L][1L]** | 0.0150 | -0.0158 | 0.0458 | **m[4R][5L]** | NA | NA | NA |
| **m[5L][1L]** | 0.0143 | -0.0120 | 0.0406 | **m[5R][5L]** | 0.0170 | -0.0142 | 0.0482 |
| **m[6L][1L]** | 0.0155 | -0.0155 | 0.0465 | **m[6R][5L]** | 0.0185 | -0.0117 | 0.0487 |
| **m[7L][1L]** | 0.0158 | -0.0144 | 0.0460 | **m[7R][5L]** | 0.0160 | -0.0105 | 0.0425 |
| **m[1R][2L]** | 0.0182 | -0.0149 | 0.0513 | **m[1L][5L]** | 0.0158 | -0.0156 | 0.0472 |
| **m[2R][2L]** | 0.0157 | -0.0155 | 0.0469 | **m[2L][5L]** | 0.0159 | -0.0123 | 0.0441 |
| **m[3R][2L]** | 0.0151 | -0.0125 | 0.0427 | **m[3L][5L]** | 0.0155 | -0.0153 | 0.0463 |
| **m[4R][2L]** | NA | NA | NA | **m[4L][5L]** | 0.0155 | -0.0129 | 0.0439 |
| **m[5R][2L]** | 0.0176 | -0.0204 | 0.0556 | **m[6L][5L]** | 0.0155 | -0.0086 | 0.0396 |
| **m[6R][2L]** | 0.0161 | -0.0106 | 0.0428 | **m[7L][5L]** | 0.0178 | -0.0189 | 0.0545 |
| **m[7R][2L]** | 0.0137 | -0.0196 | 0.0470 | **m[1R][6L]** | 0.0154 | -0.0109 | 0.0417 |
| **m[1L][2L]** | 0.0162 | -0.0191 | 0.0515 | **m[2R][6L]** | 0.0151 | -0.0131 | 0.0433 |
| **m[3L][2L]** | 0.0173 | -0.0174 | 0.0520 | **m[3R][6L]** | 0.0160 | -0.0120 | 0.0440 |
| **m[4L][2L]** | 0.0142 | -0.0130 | 0.0414 | **m[4R][6L]** | NA | NA | NA |
| **m[5L][2L]** | 0.0163 | -0.0127 | 0.0453 | **m[5R][6L]** | 0.0168 | -0.0126 | 0.0462 |
| **m[6L][2L]** | 0.0173 | -0.0139 | 0.0485 | **m[6R][6L]** | 0.0215 | -0.0179 | 0.0609 |
| **m[7L][2L]** | 0.0187 | -0.0187 | 0.0561 | **m[7R][6L]** | 0.0158 | -0.0138 | 0.0454 |
| **m[1R][3L]** | 0.0149 | -0.0135 | 0.0433 | **m[1L][6L]** | 0.0156 | -0.0169 | 0.0481 |
| **m[2R][3L]** | 0.0158 | -0.0148 | 0.0464 | **m[2L][6L]** | 0.0185 | -0.0140 | 0.0510 |
| **m[3R][3L]** | 0.0126 | -0.0141 | 0.0393 | **m[3L][6L]** | 0.0159 | -0.0151 | 0.0469 |
| **m[4R][3L]** | NA | NA | NA | **m[4L][6L]** | 0.0175 | -0.0166 | 0.0516 |
| **m[5R][3L]** | 0.0158 | -0.0142 | 0.0458 | **m[5L][6L]** | 0.0168 | -0.0132 | 0.0468 |
| **m[6R][3L]** | 0.0124 | -0.0129 | 0.0377 | **m[7L][6L]** | 0.0151 | -0.0125 | 0.0427 |
| **m[7R][3L]** | 0.0167 | -0.0141 | 0.0475 | **m[1R][7L]** | 0.0200 | -0.0184 | 0.0584 |
| **m[1L][3L]** | 0.0198 | -0.0155 | 0.0551 | **m[2R][7L]** | 0.0197 | -0.0130 | 0.0524 |
| **m[2L][3L]** | 0.0164 | -0.0154 | 0.0482 | **m[3R][7L]** | 0.0162 | -0.0095 | 0.0419 |
| **m[4L][3L]** | 0.0182 | -0.0143 | 0.0507 | **m[4R][7L]** | NA | NA | NA |
| **m[5L][3L]** | 0.0150 | -0.0117 | 0.0417 | **m[5R][7L]** | 0.0155 | -0.0135 | 0.0445 |

| **Site pairs** | **m** | **CI95%-** | **CI95%+** | **Site pairs** | **m** | **CI95%-** | **CI95%+** |
| --- | --- | --- | --- | --- | --- | --- | --- |
| **m[6L][3L]** | 0.0153 | -0.0098 | 0.0404 | **m[6R][7L]** | 0.0177 | -0.0107 | 0.0461 |
| **m[7L][3L]** | 0.0157 | -0.0133 | 0.0447 | **m[7R][7L]** | 0.0144 | -0.0107 | 0.0395 |
| **m[1R][4L]** | 0.0162 | -0.0124 | 0.0448 | **m[1L][7L]** | 0.0166 | -0.0138 | 0.0470 |
| **m[2R][4L]** | 0.0160 | -0.0101 | 0.0421 | **m[2L][7L]** | 0.0156 | -0.0122 | 0.0434 |
| **m[3R][4L]** | 0.0167 | -0.0105 | 0.0439 | **m[3L][7L]** | 0.0179 | -0.0178 | 0.0536 |
| **m[4R][4L]** | NA | NA | NA | **m[4L][7L]** | 0.0182 | -0.0222 | 0.0586 |
| **m[5R][4L]** | 0.0161 | -0.0131 | 0.0453 | **m[5L][7L]** | 0.0192 | -0.0114 | 0.0498 |
| **m[6R][4L]** | 0.0362 | -0.0144 | 0.0868 | **m[6L][7L]** | 0.0370 | -0.0034 | 0.0774 |

(F) *Amphirrhox longifolia**

| **Site pairs** | **m** | **CI95%-** | **CI95%+** | **Site pairs** | **m** | **CI95%-** | **CI95%+** |
| --- | --- | --- | --- | --- | --- | --- | --- |
| **m[2R][1R]** | 0.0151 | 0.0008 | 0.0294 | **m[6L][7L]** | 0.0152 | 0.0006 | 0.0298 |
| **m[4R][1R]** | 0.0151 | 0.0010 | 0.0292 | **m[5L][7L]** | **0.0173** | **0.0011** | **0.0335** |
| **m[5R][1R]** | 0.0152 | 0.0008 | 0.0296 | **m[4L][7L]** | 0.0148 | 0.0008 | 0.0288 |
| **m[6R][1R]** | 0.0160 | 0.0010 | 0.0310 | **m[3L][7L]** | 0.0149 | 0.0009 | 0.0289 |
| **m[7R][1R]** | 0.0161 | 0.0008 | 0.0314 | **m[2L][7L]** | 0.0149 | 0.0008 | 0.0290 |
| **m[7L][1R]** | 0.0150 | 0.0008 | 0.0292 | **m[1L][7L]** | 0.0152 | 0.0006 | 0.0298 |
| **m[6L][1R]** | 0.0151 | 0.0005 | 0.0297 | **m[1R][6L]** | 0.0152 | 0.0010 | 0.0294 |
| **m[5L][1R]** | **0.0172** | **0.0008** | **0.0336** | **m[2R][6L]** | 0.0151 | 0.0006 | 0.0296 |
| **m[4L][1R]** | 0.0152 | 0.0009 | 0.0295 | **m[4R][6L]** | 0.0152 | 0.0007 | 0.0297 |
| **m[3L][1R]** | 0.0152 | 0.0008 | 0.0296 | **m[5R][6L]** | 0.0152 | 0.0006 | 0.0298 |
| **m[2L][1R]** | 0.0149 | 0.0003 | 0.0295 | **m[6R][6L]** | 0.0161 | 0.0008 | 0.0314 |
| **m[1L][1R]** | 0.0152 | 0.0008 | 0.0296 | **m[7R][6L]** | 0.0163 | 0.0011 | 0.0315 |
| **m[1R][2R]** | 0.0150 | 0.0006 | 0.0294 | **m[7L][6L]** | 0.0153 | 0.0011 | 0.0295 |
| **m[4R][2R]** | 0.0148 | 0.0010 | 0.0286 | **m[5L][6L]** | **0.0176** | **0.0009** | **0.0343** |
| **m[5R][2R]** | 0.0148 | 0.0008 | 0.0288 | **m[4L][6L]** | 0.0153 | 0.0010 | 0.0296 |
| **m[6R][2R]** | 0.0163 | 0.0010 | 0.0316 | **m[3L][6L]** | 0.0150 | 0.0010 | 0.0290 |
| **m[7R][2R]** | 0.0160 | 0.0010 | 0.0310 | **m[2L][6L]** | 0.0152 | 0.0011 | 0.0293 |
| **m[7L][2R]** | 0.0152 | 0.0008 | 0.0296 | **m[1L][6L]** | 0.0154 | 0.0010 | 0.0298 |
| **m[6L][2R]** | 0.0152 | 0.0005 | 0.0299 | **m[1R][5L]** | **0.1459** | **0.1088** | **0.1830** |
| **m[5L][2R]** | **0.0173** | **0.0011** | **0.0335** | **m[2R][5L]** | **0.1468** | **0.1087** | **0.1849** |
| **m[4L][2R]** | 0.0148 | 0.0010 | 0.0286 | **m[4R][5L]** | **0.1460** | **0.1085** | **0.1835** |
| **m[3L][2R]** | 0.0152 | 0.0009 | 0.0295 | **m[5R][5L]** | **0.1460** | **0.1074** | **0.1846** |
| **m[2L][2R]** | 0.0153 | 0.0008 | 0.0298 | **m[6R][5L]** | **0.1345** | **0.0953** | **0.1737** |
| **m[1L][2R]** | 0.0151 | 0.0009 | 0.0293 | **m[7R][5L]** | **0.1340** | **0.0950** | **0.1730** |
| **m[1R][4R]** | 0.0153 | 0.0009 | 0.0297 | **m[7L][5L]** | **0.1457** | **0.1077** | **0.1837** |
| **m[2R][4R]** | 0.0154 | 0.0007 | 0.0301 | **m[6L][5L]** | **0.1461** | **0.1087** | **0.1835** |
| **m[5R][4R]** | 0.0153 | 0.0005 | 0.0301 | **m[4L][5L]** | **0.1458** | **0.1080** | **0.1836** |
| **m[6R][4R]** | 0.0157 | 0.0009 | 0.0305 | **m[3L][5L]** | **0.1462** | **0.1094** | **0.1830** |
| **m[7R][4R]** | 0.0164 | 0.0006 | 0.0322 | **m[2L][5L]** | **0.1464** | **0.1080** | **0.1848** |
| **m[7L][4R]** | 0.0150 | 0.0008 | 0.0292 | **m[1L][5L]** | **0.1456** | **0.1072** | **0.1840** |
| **m[6L][4R]** | 0.0148 | 0.0007 | 0.0289 | **m[1R][4L]** | 0.0146 | 0.0009 | 0.0283 |
| **m[5L][4R]** | **0.0178** | **0.0007** | **0.0349** | **m[2R][4L]** | 0.0148 | 0.0008 | 0.0288 |
| **m[4L][4R]** | 0.0152 | 0.0009 | 0.0295 | **m[4R][4L]** | 0.0150 | 0.0004 | 0.0296 |
| **m[3L][4R]** | 0.0148 | 0.0008 | 0.0288 | **m[5R][4L]** | 0.0150 | 0.0009 | 0.0291 |
| **m[2L][4R]** | 0.0151 | 0.0009 | 0.0293 | **m[6R][4L]** | 0.0163 | 0.0009 | 0.0317 |
| **m[1L][4R]** | 0.0151 | 0.0008 | 0.0294 | **m[7R][4L]** | 0.0162 | 0.0013 | 0.0311 |
| **m[1R][5R]** | 0.0154 | 0.0012 | 0.0296 | **m[7L][4L]** | 0.0151 | 0.0008 | 0.0294 |
| **m[2R][5R]** | 0.0150 | 0.0010 | 0.0290 | **m[6L][4L]** | 0.0153 | 0.0010 | 0.0296 |
| **m[4R][5R]** | 0.0155 | 0.0008 | 0.0302 | **m[5L][4L]** | **0.0173** | **0.0007** | **0.0339** |
| **m[6R][5R]** | 0.0161 | 0.0010 | 0.0312 | **m[3L][4L]** | 0.0152 | 0.0008 | 0.0296 |
| **m[7R][5R]** | 0.0164 | 0.0009 | 0.0319 | **m[2L][4L]** | 0.0150 | 0.0008 | 0.0292 |
| **m[7L][5R]** | 0.0152 | 0.0006 | 0.0298 | **m[1L][4L]** | 0.0151 | 0.0010 | 0.0292 |
| **m[6L][5R]** | 0.0150 | 0.0010 | 0.0290 | **m[1R][3L]** | 0.0150 | 0.0007 | 0.0293 |
| **m[5L][5R]** | **0.0171** | **0.0008** | **0.0334** | **m[2R][3L]** | 0.0149 | 0.0008 | 0.0290 |

| **Site pairs** | **m** | **CI95%-** | **CI95%+** | **Site pairs** | **m** | **CI95%-** | **CI95%+** |
| --- | --- | --- | --- | --- | --- | --- | --- |
| **m[4L][5R]** | 0.0155 | 0.0007 | 0.0303 | **m[4R][3L]** | 0.0152 | 0.0010 | 0.0294 |
| **m[3L][5R]** | 0.0156 | 0.0005 | 0.0307 | **m[5R][3L]** | 0.0149 | 0.0008 | 0.0290 |
| **m[2L][5R]** | 0.0153 | 0.0005 | 0.0301 | **m[6R][3L]** | 0.0159 | 0.0006 | 0.0312 |
| **m[1L][5R]** | 0.0153 | 0.0005 | 0.0301 | **m[7R][3L]** | 0.0160 | 0.0005 | 0.0315 |
| **m[1R][6R]** | 0.0153 | 0.0010 | 0.0296 | **m[7L][3L]** | 0.0153 | 0.0007 | 0.0299 |
| **m[2R][6R]** | 0.0150 | 0.0010 | 0.0290 | **m[6L][3L]** | 0.0151 | 0.0009 | 0.0293 |
| **m[4R][6R]** | 0.0153 | 0.0006 | 0.0300 | **m[5L][3L]** | **0.0173** | **0.0010** | **0.0336** |
| **m[5R][6R]** | 0.0151 | 0.0009 | 0.0293 | **m[4L][3L]** | 0.0152 | 0.0010 | 0.0294 |
| **m[7R][6R]** | 0.0164 | 0.0009 | 0.0319 | **m[2L][3L]** | 0.0151 | 0.0009 | 0.0293 |
| m[7L][6R] | 0.0154 | 0.0011 | 0.0297 | m[1L][3L] | 0.0150 | 0.0009 | 0.0291 |
| m[6L][6R] | 0.0152 | 0.0009 | 0.0295 | m[1R][2L] | 0.0152 | 0.0009 | 0.0295 |
| **m[5L][6R]** | **0.0172** | **0.0008** | **0.0336** | m[2R][2L] | 0.0150 | 0.0005 | 0.0295 |
| m[4L][6R] | 0.0152 | 0.0012 | 0.0292 | m[4R][2L] | 0.0151 | 0.0010 | 0.0292 |
| m[3L][6R] | 0.0149 | 0.0007 | 0.0291 | m[5R][2L] | 0.0154 | 0.0012 | 0.0296 |
| m[2L][6R] | 0.0150 | 0.0010 | 0.0290 | m[6R][2L] | 0.0160 | 0.0015 | 0.0305 |
| m[1L][6R] | 0.0151 | 0.0008 | 0.0294 | m[7R][2L] | 0.0155 | 0.0007 | 0.0303 |
| m[1R][7R] | 0.0154 | 0.0010 | 0.0298 | m[7L][2L] | 0.0153 | 0.0009 | 0.0297 |
| m[2R][7R] | 0.0150 | 0.0008 | 0.0292 | m[6L][2L] | 0.0153 | 0.0007 | 0.0299 |
| m[4R][7R] | 0.0153 | 0.0010 | 0.0296 | **m[5L][2L]** | **0.0181** | **0.0009** | **0.0353** |
| m[5R][7R] | 0.0153 | 0.0009 | 0.0297 | m[4L][2L] | 0.0154 | 0.0009 | 0.0299 |
| m[6R][7R] | 0.0160 | 0.0010 | 0.0310 | m[3L][2L] | 0.0151 | 0.0008 | 0.0294 |
| m[7L][7R] | 0.0152 | 0.0011 | 0.0293 | m[1L][2L] | 0.0154 | 0.0008 | 0.0300 |
| m[6L][7R] | 0.0155 | 0.0008 | 0.0302 | m[1R][1L] | 0.0148 | 0.0008 | 0.0288 |
| **m[5L][7R]** | **0.0172** | **0.0008** | **0.0336** | m[2R][1L] | 0.0152 | 0.0007 | 0.0297 |
| m[4L][7R] | 0.0153 | 0.0010 | 0.0296 | m[4R][1L] | 0.0152 | 0.0012 | 0.0292 |
| m[3L][7R] | 0.0150 | 0.0009 | 0.0291 | m[5R][1L] | 0.0152 | 0.0010 | 0.0294 |
| m[2L][7R] | 0.0154 | 0.0009 | 0.0299 | m[6R][1L] | 0.0159 | 0.0007 | 0.0311 |
| m[1L][7R] | 0.0151 | 0.0008 | 0.0294 | m[7R][1L] | 0.0161 | 0.0009 | 0.0313 |
| m[1R][7L] | 0.0153 | 0.0008 | 0.0298 | m[7L][1L] | 0.0150 | 0.0012 | 0.0288 |
| m[2R][7L] | 0.0153 | 0.0011 | 0.0295 | m[6L][1L] | 0.0150 | 0.0011 | 0.0289 |
| m[4R][7L] | 0.0152 | 0.0007 | 0.0297 | **m[5L][1L]** | **0.0176** | **0.0008** | **0.0344** |
| m[5R][7L] | 0.0153 | 0.0009 | 0.0297 | m[4L][1L] | 0.0151 | 0.0012 | 0.0290 |
| m[6R][7L] | 0.0161 | 0.0010 | 0.0312 | m[3L][1L] | 0.0151 | 0.0009 | 0.0293 |
| m[7R][7L] | 0.0159 | 0.0009 | 0.0309 | m[2L][1L] | 0.0151 | 0.0006 | 0.0296 |

*From Nazareno et al. 2019a

(G) *Passiflora spinosa**

| **Site pairs** | **Mean** | **95%CI-** | **95%CI+** | **Site pairs** | **Mean** | **95%CI-** | **95%CI+** |
| --- | --- | --- | --- | --- | --- | --- | --- |
| **m[2L][1L]** | 0.0155 | 0.0009 | 0.0301 | **m[1R][7L]** | 0.0153 | 0.0007 | 0.0299 |
| **m[3L][1L]** | 0.0157 | 0.0012 | 0.0302 | **m[2R][7L]** | 0.0214 | -0.0013 | 0.0441 |
| **m[4L][1L]** | 0.0155 | 0.0012 | 0.0298 | **m[3R][7L]** | 0.0152 | 0.0005 | 0.0299 |
| **m[5L][1L]** | 0.0152 | 0.0008 | 0.0296 | **m[4R][7L]** | 0.0152 | 0.0009 | 0.0295 |
| **m[6L][1L]** | 0.0152 | 0.0007 | 0.0297 | **m[5R][7L]** | **0.0166** | **-0.0003** | **0.0335** |
| **m[7L][1L]** | 0.0172 | 0.0008 | 0.0336 | **m[6R][7L]** | 0.0155 | 0.0011 | 0.0299 |
| **m[1R][1L]** | 0.0154 | 0.0007 | 0.0301 | **m[1L][1R]** | 0.0151 | 0.0005 | 0.0297 |
| **m[2R][1L]** | 0.0190 | 0.0042 | 0.0338 | **m[2L][1R]** | 0.0155 | 0.0013 | 0.0297 |
| **m[3R][1L]** | 0.0152 | 0.0007 | 0.0297 | **m[3L][1R]** | 0.0159 | 0.0012 | 0.0306 |
| **m[4R][1L]** | 0.0154 | 0.0010 | 0.0298 | **m[4L][1R]** | 0.0219 | 0.0001 | 0.0437 |
| **m[5R][1L]** | **0.0167** | **-0.0004** | **0.0338** | **m[5L][1R]** | 0.0152 | 0.0007 | 0.0297 |
| **m[6R][1L]** | 0.0156 | 0.0013 | 0.0299 | **m[6L][1R]** | 0.0153 | 0.0006 | 0.0300 |
| **m[1L][2L]** | 0.0152 | 0.0008 | 0.0296 | **m[7L][1R]** | 0.0171 | 0.0012 | 0.0330 |
| **m[3L][2L]** | 0.0156 | 0.0011 | 0.0301 | **m[2R][1R]** | 0.0190 | 0.0045 | 0.0335 |
| **m[4L][2L]** | 0.0154 | 0.0004 | 0.0304 | **m[3R][1R]** | 0.0154 | 0.0006 | 0.0302 |
| **m[5L][2L]** | 0.0150 | 0.0008 | 0.0292 | **m[4R][1R]** | 0.0154 | 0.0007 | 0.0301 |
| **m[6L][2L]** | 0.0154 | 0.0006 | 0.0302 | **m[5R][1R]** | **0.0166** | **0.0005** | **0.0327** |
| **m[7L][2L]** | 0.0173 | 0.0008 | 0.0338 | **m[6R][1R]** | 0.0156 | 0.0013 | 0.0299 |
| **m[1R][2L]** | 0.0153 | 0.0003 | 0.0303 | **m[1L][2R]** | 0.0153 | 0.0009 | 0.0297 |
| **m[2R][2L]** | 0.0269 | 0.0039 | 0.0499 | **m[2L][2R]** | 0.0313 | 0.0088 | 0.0538 |
| **m[3R][2L]** | 0.0152 | 0.0006 | 0.0298 | **m[3L][2R]** | 0.0159 | 0.0013 | 0.0305 |
| **m[4R][2L]** | 0.0155 | 0.0009 | 0.0301 | **m[4L][2R]** | 0.0253 | 0.0107 | 0.0399 |
| **m[5R][2L]** | **0.0168** | **-0.0001** | **0.0337** | **m[5L][2R]** | 0.0152 | 0.0006 | 0.0298 |
| **m[6R][2L]** | 0.0157 | 0.0012 | 0.0302 | **m[6L][2R]** | 0.0152 | 0.0010 | 0.0294 |
| **m[1L][3L]** | 0.0413 | 0.0270 | 0.0556 | **m[7L][2R]** | 0.0172 | 0.0011 | 0.0333 |
| **m[2L][3L]** | 0.0374 | 0.0229 | 0.0519 | **m[1R][2R]** | 0.0300 | 0.0085 | 0.0515 |
| **m[4L][3L]** | 0.0375 | 0.0226 | 0.0524 | **m[3R][2R]** | 0.0152 | 0.0011 | 0.0293 |
| **m[5L][3L]** | 0.0410 | 0.0262 | 0.0558 | **m[4R][2R]** | 0.0153 | 0.0011 | 0.0295 |
| **m[6L][3L]** | 0.0412 | 0.0270 | 0.0554 | **m[5R][2R]** | **0.0162** | **0.0002** | **0.0322** |
| **m[7L][3L]** | 0.0374 | 0.0214 | 0.0534 | **m[6R][2R]** | 0.0158 | 0.0014 | 0.0302 |
| **m[1R][3L]** | 0.0376 | 0.0230 | 0.0522 | **m[1L][3R]** | 0.0153 | 0.0010 | 0.0296 |
| **m[2R][3L]** | 0.0373 | 0.0223 | 0.0523 | **m[2L][3R]** | 0.0155 | 0.0007 | 0.0303 |
| **m[3R][3L]** | 0.0412 | 0.0267 | 0.0557 | **m[3L][3R]** | 0.0159 | 0.0012 | 0.0306 |
| **m[4R][3L]** | 0.0412 | 0.0268 | 0.0556 | **m[4L][3R]** | 0.0154 | 0.0007 | 0.0301 |
| **m[5R][3L]** | 0.0426 | 0.0261 | 0.0591 | **m[5L][3R]** | 0.0152 | 0.0007 | 0.0297 |
| **m[6R][3L]** | 0.0417 | 0.0273 | 0.0561 | **m[6L][3R]** | 0.0152 | 0.0007 | 0.0297 |
| **m[1L][4L]** | 0.0152 | 0.0006 | 0.0298 | **m[7L][3R]** | 0.0171 | 0.0010 | 0.0332 |
| **m[2L][4L]** | 0.0155 | 0.0006 | 0.0304 | **m[1R][3R]** | 0.0155 | 0.0008 | 0.0302 |
| **m[3L][4L]** | 0.0156 | 0.0012 | 0.0300 | **m[2R][3R]** | 0.0157 | 0.0010 | 0.0304 |
| **m[5L][4L]** | 0.0153 | 0.0013 | 0.0293 | **m[4R][3R]** | 0.0151 | 0.0010 | 0.0292 |
| **m[6L][4L]** | 0.0152 | 0.0008 | 0.0296 | **m[5R][3R]** | **0.0166** | **-0.0001** | **0.0333** |
| **m[7L][4L]** | 0.0170 | 0.0007 | 0.0333 | **m[6R][3R]** | 0.0156 | 0.0015 | 0.0297 |
| **m[1R][4L]** | 0.0155 | 0.0006 | 0.0304 | **m[1L][4R]** | 0.0152 | 0.0011 | 0.0293 |
| **m[2R][4L]** | 0.0158 | 0.0005 | 0.0311 | **m[2L][4R]** | 0.0157 | 0.0006 | 0.0308 |

| **Site pairs** | **Mean** | **95%CI-** | **95%CI+** | **Site pairs** | **Mean** | **95%CI-** | **95%CI+** |
| --- | --- | --- | --- | --- | --- | --- | --- |
| **m[3R][4L]** | 0.0153 | 0.0012 | 0.0294 | **m[3L][4R]** | 0.0155 | 0.0009 | 0.0301 |
| **m[4R][4L]** | 0.0152 | 0.0011 | 0.0293 | **m[4L][4R]** | 0.0154 | 0.0010 | 0.0298 |
| **m[5R][4L]** | **0.0167** | **-0.0001** | **0.0335** | **m[5L][4R]** | 0.0152 | 0.0008 | 0.0296 |
| **m[6R][4L]** | 0.0156 | 0.0013 | 0.0299 | **m[6L][4R]** | 0.0153 | 0.0011 | 0.0295 |
| **m[1L][5L]** | 0.0152 | 0.0008 | 0.0296 | **m[7L][4R]** | 0.0170 | 0.0009 | 0.0331 |
| **m[2L][5L]** | 0.0156 | 0.0008 | 0.0304 | **m[1R][4R]** | 0.0167 | 0.0022 | 0.0312 |
| **m[3L][5L]** | 0.0156 | 0.0015 | 0.0297 | **m[2R][4R]** | 0.0158 | 0.0004 | 0.0312 |
| **m[4L][5L]** | 0.0155 | 0.0001 | 0.0309 | **m[3R][4R]** | 0.0153 | 0.0006 | 0.0300 |
| **m[6L][5L]** | 0.0153 | 0.0008 | 0.0298 | **m[5R][4R]** | **0.0166** | **-0.0005** | **0.0337** |
| **m[7L][5L]** | 0.0170 | 0.0006 | 0.0334 | **m[6R][4R]** | 0.0157 | 0.0015 | 0.0299 |
| **m[1R][5L]** | 0.0154 | 0.0010 | 0.0298 | **m[1L][5R]** | **0.0935** | **0.0550** | **0.1320** |
| **m[2R][5L]** | 0.0157 | 0.0004 | 0.0310 | **m[2L][5R]** | **0.0814** | **0.0440** | **0.1188** |
| **m[3R][5L]** | 0.0153 | 0.0008 | 0.0298 | **m[3L][5R]** | 0.0934 | 0.0561 | 0.1307 |
| **m[4R][5L]** | 0.0153 | 0.0012 | 0.0294 | **m[4L][5R]** | **0.0819** | **0.0449** | **0.1189** |
| **m[5R][5L]** | **0.0166** | **0.0000** | **0.0332** | **m[5L][5R]** | **0.0937** | **0.0552** | **0.1322** |
| **m[6R][5L]** | 0.0158 | 0.0012 | 0.0304 | **m[6L][5R]** | **0.0929** | **0.0550** | **0.1308** |
| **m[1L][6L]** | 0.0152 | 0.0009 | 0.0295 | **m[7L][5R]** | **0.0795** | **0.0395** | **0.1195** |
| **m[2L][6L]** | 0.0154 | 0.0009 | 0.0299 | **m[1R][5R]** | **0.0819** | **0.0435** | **0.1203** |
| **m[3L][6L]** | 0.0159 | 0.0015 | 0.0303 | **m[2R][5R]** | **0.0723** | **0.0340** | **0.1106** |
| **m[4L][6L]** | 0.0155 | 0.0008 | 0.0302 | **m[3R][5R]** | **0.0928** | **0.0546** | **0.1310** |
| **m[5L][6L]** | 0.0153 | 0.0011 | 0.0295 | **m[4R][5R]** | **0.0931** | **0.0546** | **0.1316** |
| **m[7L][6L]** | 0.0173 | 0.0013 | 0.0333 | **m[6R][5R]** | 0.0940 | 0.0559 | 0.1321 |
| **m[1R][6L]** | 0.0156 | 0.0007 | 0.0305 | **m[1L][6R]** | 0.0411 | 0.0268 | 0.0554 |
| **m[2R][6L]** | 0.0190 | 0.0043 | 0.0337 | **m[2L][6R]** | 0.0380 | 0.0231 | 0.0529 |
| **m[3R][6L]** | 0.0153 | 0.0009 | 0.0297 | **m[3L][6R]** | 0.0414 | 0.0271 | 0.0557 |
| **m[4R][6L]** | 0.0154 | 0.0004 | 0.0304 | **m[4L][6R]** | 0.0373 | 0.0227 | 0.0519 |
| **m[5R][6L]** | **0.0165** | **-0.0003** | **0.0333** | **m[5L][6R]** | 0.0413 | 0.0270 | 0.0556 |
| **m[6R][6L]** | 0.0157 | 0.0012 | 0.0302 | **m[6L][6R]** | 0.0413 | 0.0269 | 0.0557 |
| **m[1L][7L]** | 0.0151 | 0.0013 | 0.0289 | **m[7L][6R]** | 0.0381 | 0.0219 | 0.0543 |
| **m[2L][7L]** | 0.0155 | 0.0006 | 0.0304 | **m[1R][6R]** | 0.0379 | 0.0236 | 0.0522 |
| **m[3L][7L]** | 0.0156 | 0.0015 | 0.0297 | **m[2R][6R]** | 0.0340 | 0.0192 | 0.0488 |
| **m[4L][7L]** | 0.0154 | 0.0004 | 0.0304 | **m[3R][6R]** | 0.0411 | 0.0267 | 0.0555 |
| **m[5L][7L]** | 0.0151 | 0.0008 | 0.0294 | **m[4R][6R]** | 0.0409 | 0.0267 | 0.0551 |
| **m[6L][7L]** | 0.0154 | 0.0009 | 0.0299 | **m[5R][6R]** | 0.0428 | 0.0267 | 0.0589 |

*From Nazareno et al. 2019a

(H) *Psychotria lupulina**

| **Site pairs** | **m** | **95%CI-** | **95%CI+** | **Site pairs** | **m** | **95%CI-** | **95%CI+** |
| --- | --- | --- | --- | --- | --- | --- | --- |
| **m[2R][1R]** | 0.0155 | 0.0005 | 0.0305 | **m[7L][5R]** | 0.0157 | 0.0013 | 0.0301 |
| **m[3R][1R]** | **0.0159** | **0.0019** | **0.0299** | **m[6L][5R]** | 0.0155 | 0.0009 | 0.0301 |
| **m[4R][1R]** | 0.0154 | 0.0008 | 0.0300 | **m[5L][5R]** | 0.0166 | 0.0013 | 0.0319 |
| **m[5R][1R]** | 0.0153 | 0.0007 | 0.0299 | **m[4L][5R]** | 0.0153 | 0.0006 | 0.0300 |
| **m[6R][1R]** | 0.0152 | 0.0012 | 0.0292 | **m[3L][5R]** | **0.0163** | **-0.0004** | **0.0330** |
| **m[7R][1R]** | 0.0151 | 0.0012 | 0.0290 | **m[2L][5R]** | 0.0187 | 0.0014 | 0.0360 |
| **m[7L][1R]** | 0.0157 | 0.0011 | 0.0303 | **m[1R][6R]** | 0.0151 | 0.0005 | 0.0297 |
| **m[6L][1R]** | 0.0157 | 0.0006 | 0.0308 | **m[2R][6R]** | 0.0156 | 0.0013 | 0.0299 |
| **m[5L][1R]** | 0.0168 | 0.0016 | 0.0320 | **m[3R][6R]** | **0.0158** | **0.0015** | **0.0301** |
| **m[4L][1R]** | 0.0154 | 0.0007 | 0.0301 | **m[4R][6R]** | 0.0187 | 0.0041 | 0.0333 |
| **m[3L][1R]** | **0.0163** | **-0.0002** | **0.0328** | **m[5R][6R]** | 0.0151 | 0.0012 | 0.0290 |
| **m[2L][1R]** | 0.0186 | 0.0016 | 0.0356 | **m[7R][6R]** | 0.0153 | 0.0005 | 0.0301 |
| **m[1R][2R]** | 0.0155 | 0.0008 | 0.0302 | **m[7L][6R]** | 0.0156 | 0.0005 | 0.0307 |
| **m[3R][2R]** | **0.0159** | **0.0017** | **0.0301** | **m[6L][6R]** | 0.0157 | 0.0010 | 0.0304 |
| **m[4R][2R]** | 0.0154 | 0.0008 | 0.0300 | **m[5L][6R]** | 0.0165 | 0.0014 | 0.0316 |
| **m[5R][2R]** | 0.0152 | 0.0010 | 0.0294 | **m[4L][6R]** | 0.0153 | 0.0007 | 0.0299 |
| **m[6R][2R]** | 0.0152 | 0.0009 | 0.0295 | **m[3L][6R]** | **0.0165** | **0.0000** | **0.0329** |
| **m[7R][2R]** | 0.0153 | 0.0009 | 0.0297 | **m[2L][6R]** | 0.0189 | 0.0010 | 0.0368 |
| **m[7L][2R]** | 0.0159 | 0.0014 | 0.0304 | **m[1R][7R]** | 0.0154 | 0.0007 | 0.0301 |
| **m[6L][2R]** | 0.0155 | 0.0010 | 0.0300 | **m[2R][7R]** | 0.0155 | 0.0005 | 0.0305 |
| **m[5L][2R]** | 0.0166 | 0.0014 | 0.0318 | **m[3R][7R]** | 0.0162 | 0.0019 | 0.0305 |
| **m[4L][2R]** | 0.0153 | 0.0009 | 0.0297 | **m[4R][7R]** | 0.0154 | 0.0010 | 0.0298 |
| **m[3L][2R]** | 0.0164 | -0.0002 | 0.0330 | **m[5R][7R]** | 0.0150 | 0.0008 | 0.0292 |
| **m[2L][2R]** | 0.0186 | 0.0011 | 0.0361 | **m[6R][7R]** | 0.0153 | 0.0009 | 0.0297 |
| **m[1R][3R]** | **0.0443** | **0.0301** | **0.0585** | **m[7L][7R]** | 0.0157 | 0.0015 | 0.0299 |
| **m[2R][3R]** | **0.0509** | **0.0360** | **0.0658** | **m[6L][7R]** | 0.0153 | 0.0012 | 0.0294 |
| **m[4R][3R]** | 0.0409 | 0.0262 | 0.0556 | **m[5L][7R]** | 0.0166 | 0.0019 | 0.0313 |
| **m[5R][3R]** | 0.0416 | 0.0268 | 0.0564 | **m[4L][7R]** | 0.0151 | 0.0009 | 0.0293 |
| **m[6R][3R]** | **0.0484** | **0.0342** | **0.0626** | **m[3L][7R]** | **0.0164** | **0.0001** | **0.0327** |
| **m[7R][3R]** | 0.0411 | 0.0264 | 0.0558 | **m[2L][7R]** | 0.0188 | 0.0014 | 0.0362 |
| **m[7L][3R]** | 0.0471 | 0.0250 | 0.0692 | **m[1R][7L]** | 0.0153 | 0.0009 | 0.0297 |
| **m[6L][3R]** | **0.0455** | **0.0306** | **0.0604** | **m[2R][7L]** | 0.0318 | 0.0098 | 0.0538 |
| **m[5L][3R]** | 0.0433 | 0.0279 | 0.0587 | **m[3R][7L]** | 0.0160 | 0.0015 | 0.0305 |
| **m[4L][3R]** | **0.0486** | **0.0340** | **0.0632** | **m[4R][7L]** | 0.0284 | 0.0062 | 0.0506 |
| **m[3L][3R]** | 0.0563 | 0.0395 | 0.0731 | **m[5R][7L]** | 0.0153 | 0.0006 | 0.0300 |
| **m[2L][3R]** | 0.0399 | 0.0220 | 0.0578 | **m[6R][7L]** | 0.0153 | 0.0006 | 0.0300 |
| **m[1R][4R]** | 0.0152 | 0.0011 | 0.0293 | **m[7R][7L]** | 0.0155 | 0.0006 | 0.0304 |
| **m[2R][4R]** | 0.0156 | 0.0009 | 0.0303 | **m[6L][7L]** | 0.0321 | 0.0097 | 0.0545 |
| **m[3R][4R]** | 0.0158 | 0.0014 | 0.0302 | **m[5L][7L]** | 0.0168 | 0.0015 | 0.0321 |
| **m[5R][4R]** | 0.0152 | 0.0005 | 0.0299 | **m[4L][7L]** | 0.0152 | 0.0011 | 0.0293 |
| **m[6R][4R]** | 0.0152 | 0.0006 | 0.0298 | **m[3L][7L]** | 0.0163 | -0.0001 | 0.0327 |
| **m[7R][4R]** | 0.0152 | 0.0010 | 0.0294 | **m[2L][7L]** | 0.0189 | 0.0017 | 0.0361 |
| **m[7L][4R]** | 0.0302 | 0.0158 | 0.0446 | **m[1R][6L]** | 0.0154 | 0.0009 | 0.0299 |
| **m[6L][4R]** | 0.0154 | 0.0014 | 0.0294 | **m[2R][6L]** | 0.0154 | 0.0010 | 0.0298 |

| **Site pairs** | **m** | **95%CI-** | **95%CI+** | **Site pairs** | **m** | **95%CI-** | **95%CI+** |
| --- | --- | --- | --- | --- | --- | --- | --- |
| **m[5L][4R]** | 0.0166 | 0.0013 | 0.0319 | **m[3R][6L]** | **0.016** | **0.0017** | **0.0303** |
| **m[4L][4R]** | 0.0152 | 0.0011 | 0.0293 | **m[4R][6L]** | 0.0155 | 0.0006 | 0.0304 |
| **m[3L][4R]** | **0.0164** | **0.0002** | **0.0326** | **m[5R][6L]** | 0.0152 | 0.0011 | 0.0293 |
| **m[2L][4R]** | 0.0185 | 0.0012 | 0.0358 | **m[6R][6L]** | 0.0153 | 0.0010 | 0.0296 |
| **m[1R][5R]** | 0.0154 | 0.0013 | 0.0295 | **m[7R][6L]** | 0.0153 | 0.0008 | 0.0298 |
| **m[2R][5R]** | 0.0155 | 0.0007 | 0.0303 | **m[7L][6L]** | 0.0189 | 0.0042 | 0.0336 |
| **m[3R][5R]** | 0.0160 | 0.0016 | 0.0304 | **m[5L][6L]** | 0.0168 | 0.0018 | 0.0318 |
| **m[4R][5R]** | 0.0156 | 0.0014 | 0.0298 | **m[4L][6L]** | 0.0151 | 0.0010 | 0.0292 |
| **m[6R][5R]** | 0.0152 | 0.0010 | 0.0294 | **m[3L][6L]** | **0.0165** | **0.0000** | **0.0330** |
| **m[7R][5R]** | 0.0153 | 0.0008 | 0.0298 | **m[2L][6L]** | 0.0187 | 0.0010 | 0.0364 |
| **m[1R][5L]** | 0.0414 | 0.0270 | 0.0558 | **m[1R][3L]** | **0.0891** | **0.0510** | **0.1272** |
| **m[2R][5L]** | 0.0380 | 0.0228 | 0.0532 | **m[2R][3L]** | 0.0671 | 0.0285 | 0.1057 |
| **m[3R][5L]** | 0.0418 | 0.0273 | 0.0563 | **m[3R][3L]** | 0.0839 | 0.0465 | 0.1213 |
| **m[4R][5L]** | 0.0381 | 0.0236 | 0.0526 | **m[4R][3L]** | **0.0777** | **0.0394** | **0.1160** |
| **m[5R][5L]** | 0.0414 | 0.0272 | 0.0556 | **m[5R][3L]** | **0.0930** | **0.0551** | **0.1309** |
| **m[6R][5L]** | 0.0413 | 0.0272 | 0.0554 | **m[6R][3L]** | **0.0857** | **0.0480** | **0.1234** |
| **m[7R][5L]** | 0.0407 | 0.0257 | 0.0557 | **m[7R][3L]** | **0.0928** | **0.0547** | **0.1309** |
| **m[7L][5L]** | 0.0370 | 0.0221 | 0.0519 | **m[7L][3L]** | 0.0686 | 0.0300 | 0.1072 |
| **m[6L][5L]** | 0.0374 | 0.0229 | 0.0519 | **m[6L][3L]** | **0.0731** | **0.0347** | **0.1115** |
| **m[4L][5L]** | 0.0415 | 0.0264 | 0.0566 | **m[5L][3L]** | 0.0827 | 0.0432 | 0.1222 |
| **m[3L][5L]** | 0.0425 | 0.0260 | 0.0590 | **m[4L][3L]** | **0.0850** | **0.0467** | **0.1233** |
| **m[2L][5L]** | 0.0355 | 0.0186 | 0.0524 | **m[2L][3L]** | 0.0629 | 0.0219 | 0.1039 |
| **m[1R][4L]** | 0.0154 | 0.0007 | 0.0301 | **m[1R][2L]** | 0.0151 | 0.0005 | 0.0297 |
| **m[2R][4L]** | 0.0157 | 0.0008 | 0.0306 | **m[2R][2L]** | 0.0155 | 0.0008 | 0.0302 |
| **m[3R][4L]** | **0.0157** | **0.0014** | **0.0300** | **m[3R][2L]** | 0.0158 | 0.0012 | 0.0304 |
| **m[4R][4L]** | 0.0155 | 0.0010 | 0.0300 | **m[4R][2L]** | 0.0155 | 0.0010 | 0.0300 |
| **m[5R][4L]** | 0.0152 | 0.0005 | 0.0299 | **m[5R][2L]** | 0.0151 | 0.0008 | 0.0294 |
| **m[6R][4L]** | 0.0151 | 0.0008 | 0.0294 | **m[6R][2L]** | 0.0154 | 0.0008 | 0.0300 |
| **m[7R][4L]** | 0.0154 | 0.0009 | 0.0299 | **m[7R][2L]** | 0.0153 | 0.0011 | 0.0295 |
| **m[7L][4L]** | 0.0157 | 0.0009 | 0.0305 | **m[7L][2L]** | 0.0157 | 0.0011 | 0.0303 |
| **m[6L][4L]** | 0.0155 | 0.0005 | 0.0305 | **m[6L][2L]** | 0.0155 | 0.0010 | 0.0300 |
| **m[5L][4L]** | 0.0168 | 0.0013 | 0.0323 | **m[5L][2L]** | 0.0167 | 0.0011 | 0.0323 |
| **m[3L][4L]** | **0.0163** | **0.0004** | **0.0322** | **m[4L][2L]** | 0.0154 | 0.0010 | 0.0298 |
| **m[2L][4L]** | 0.0185 | 0.0015 | 0.0355 | **m[3L][2L]** | 0.0164 | -0.0004 | 0.0332 |

*From Nazareno et al. 2019a

**TABLE S8** Pearson's correlation coefficient between contemporary migration rates and geographic distances of pairs of locations sampled along the Rio Branco (Amazon Basin, Brazil). *P-values* are shown in parenthesis.

| **Plant species** | **Datasets** | | | |
| --- | --- | --- | --- | --- |
|  | **1** | **2** | **3** | **4** |
| 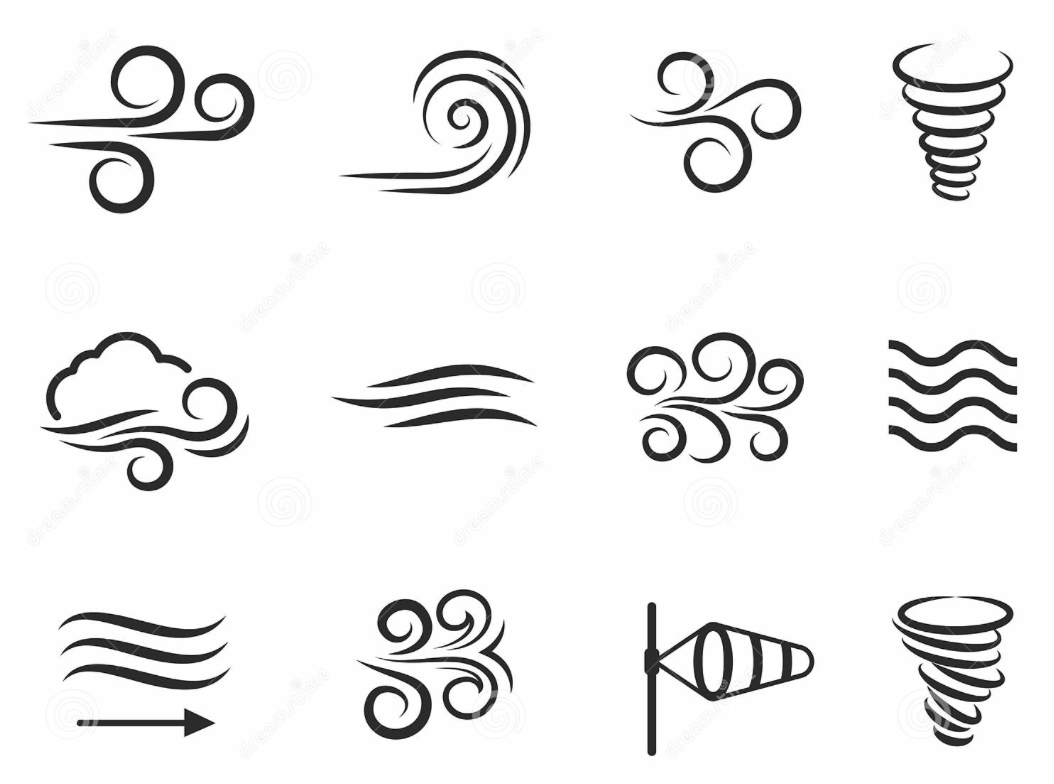  *Adenocalymma schomburgkii* | 0.07(0.53) | -0.28(0.07) | -0.07(0.02) | -0.17(0.10) |
| 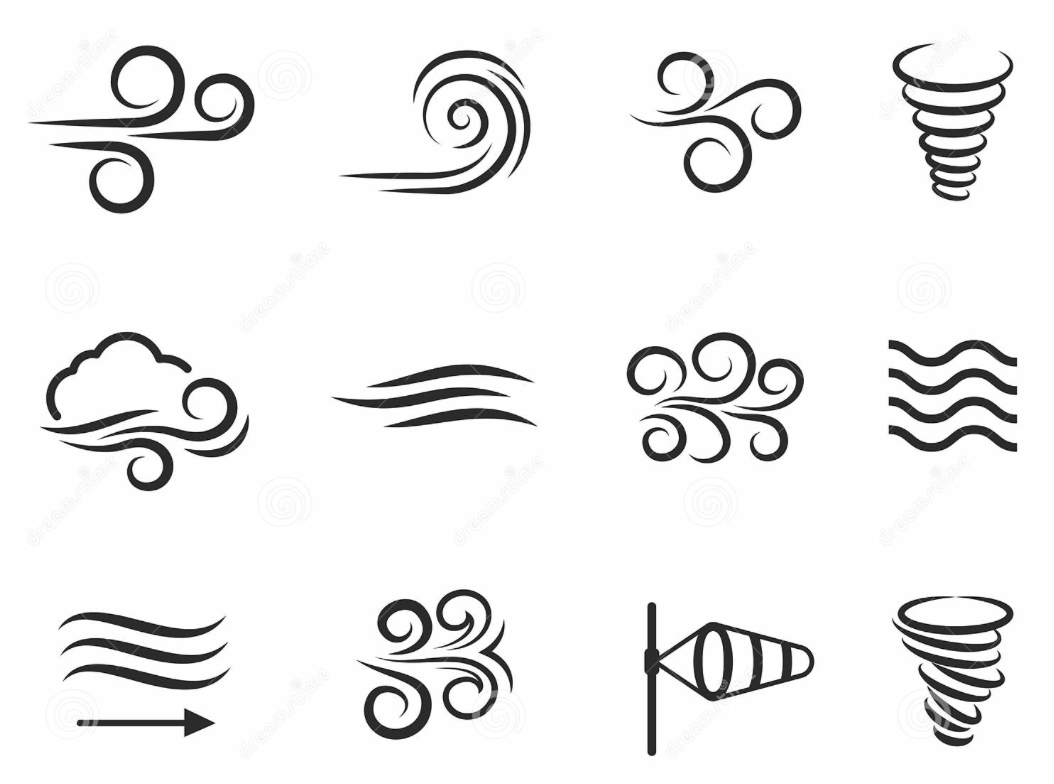*Bignonia aequinoctialis* | -0.13(0.28) | -0.29(0.11) | -0.18(0.23) | -0.14(0.21) |
| 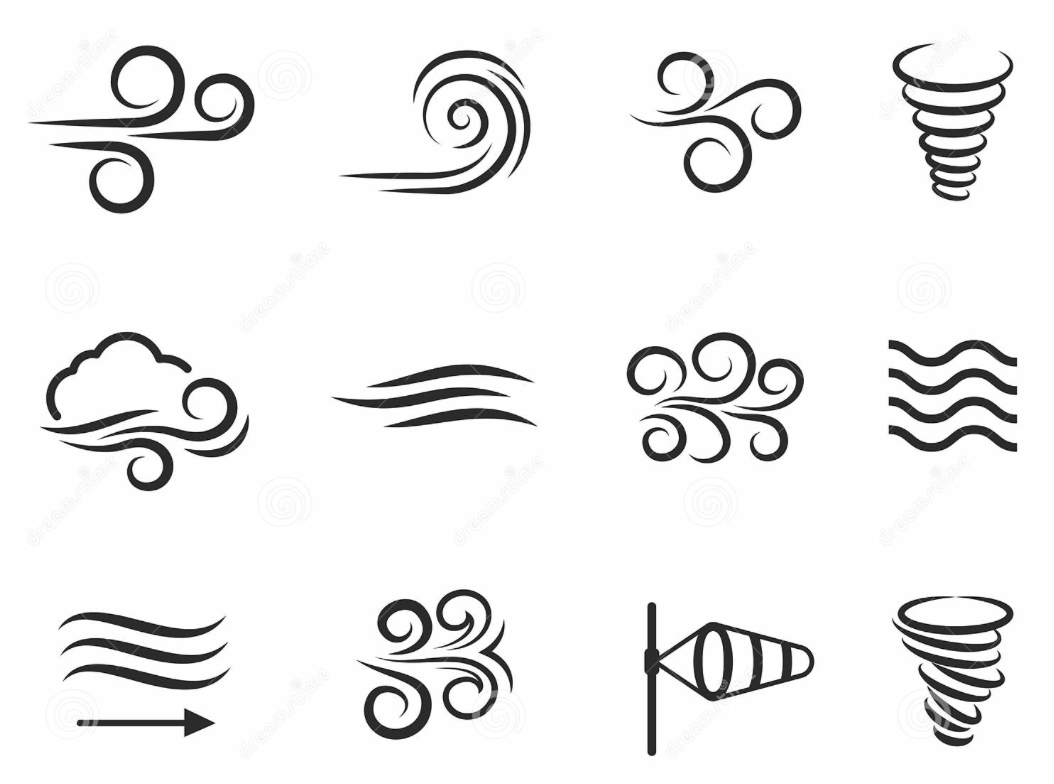*Tanaecium pyramidatum* | -0.13(0.26) | -0.16(0.38) | -0.15(0.33) | -0.15(0.21) |
| 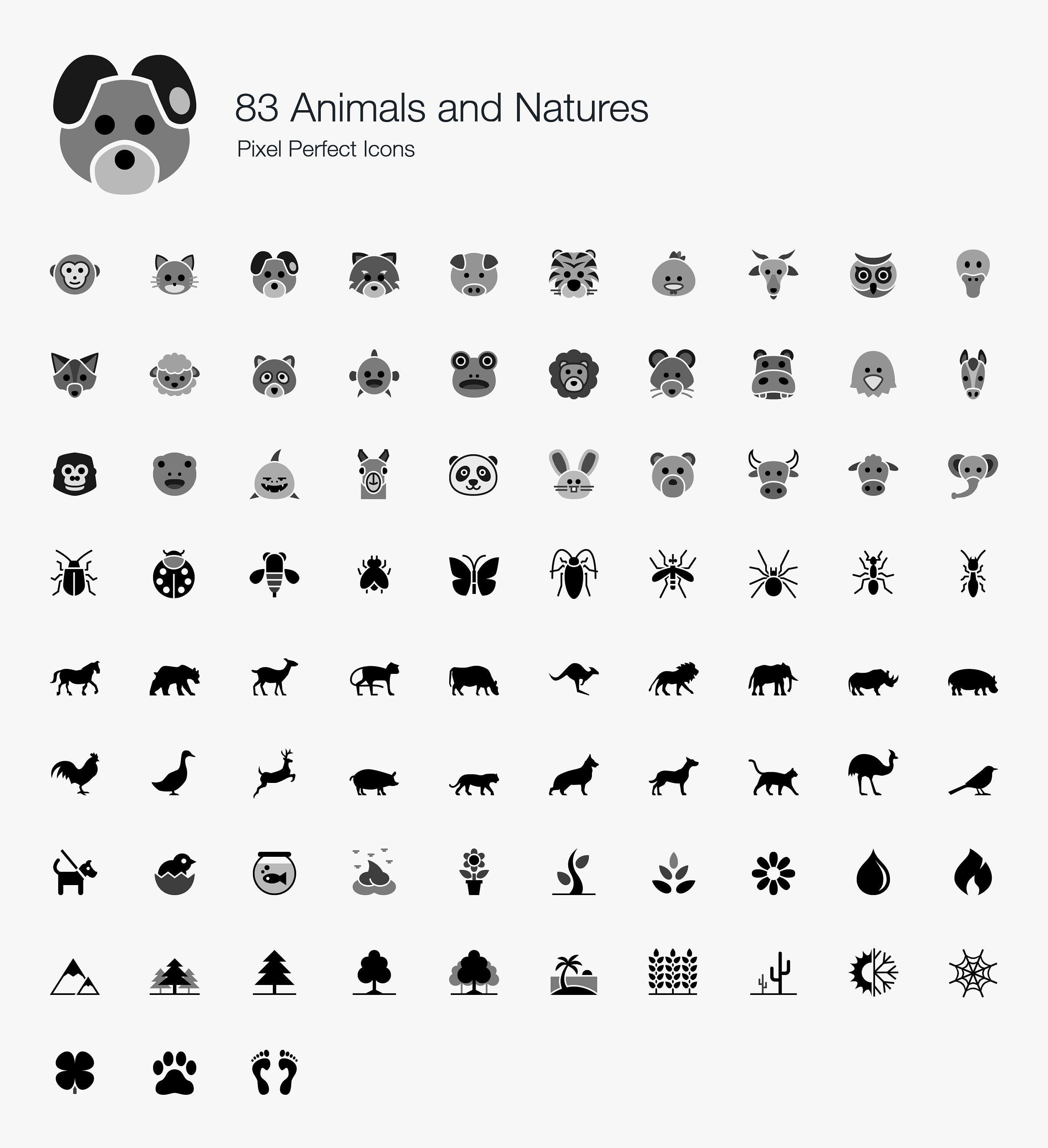  *Anemopaegma paraense* | -0.06(0.56) | -0.05(0.75) | -0.12(0.43) | -0.09(0.37) |
| 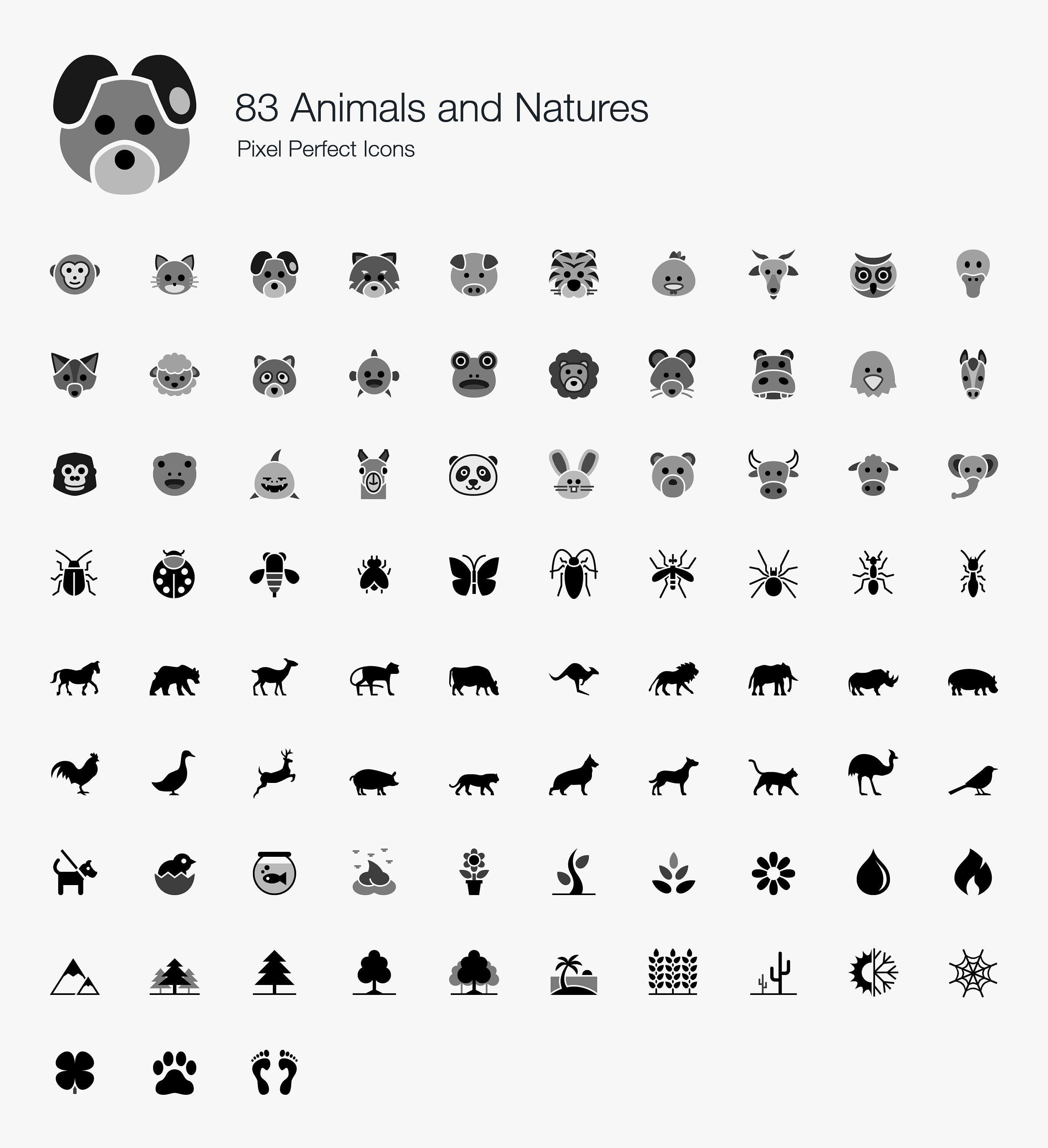  *Pachypetra kerere* | -0.06(0.55) | 0.44(0.00) | 0.06(0.68) | 0.22(0.04) |
| *Amphirrhox longifolia* | -0.10(0.39) | 0.03(0.88) | -0.17(0.27) | -0.15(0.21) |
| 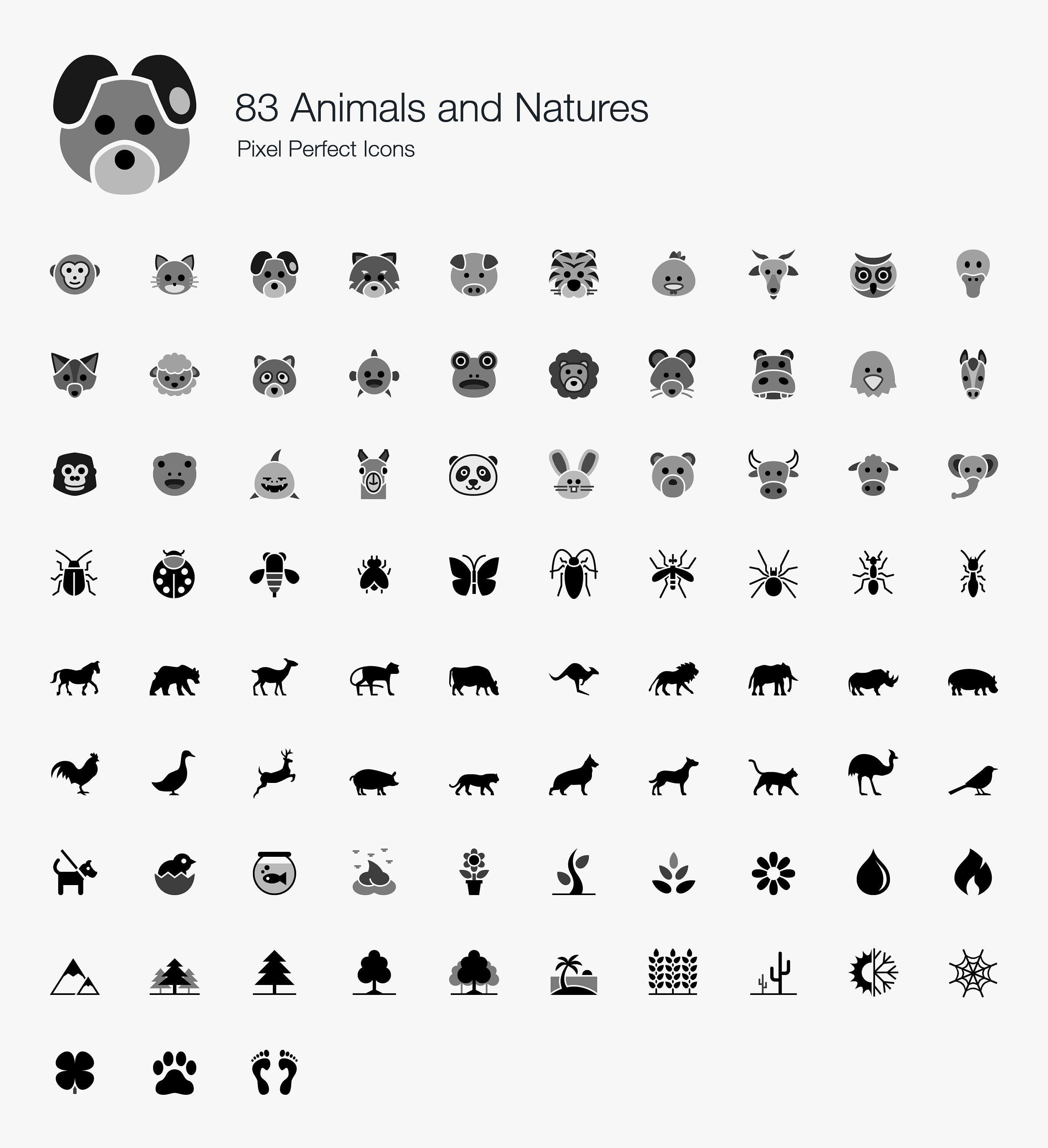*Passiflora spinosa* | -0.06(0.62) | -0.18(0.31) | -0.16(0.31) | -0.16(0.17) |
| 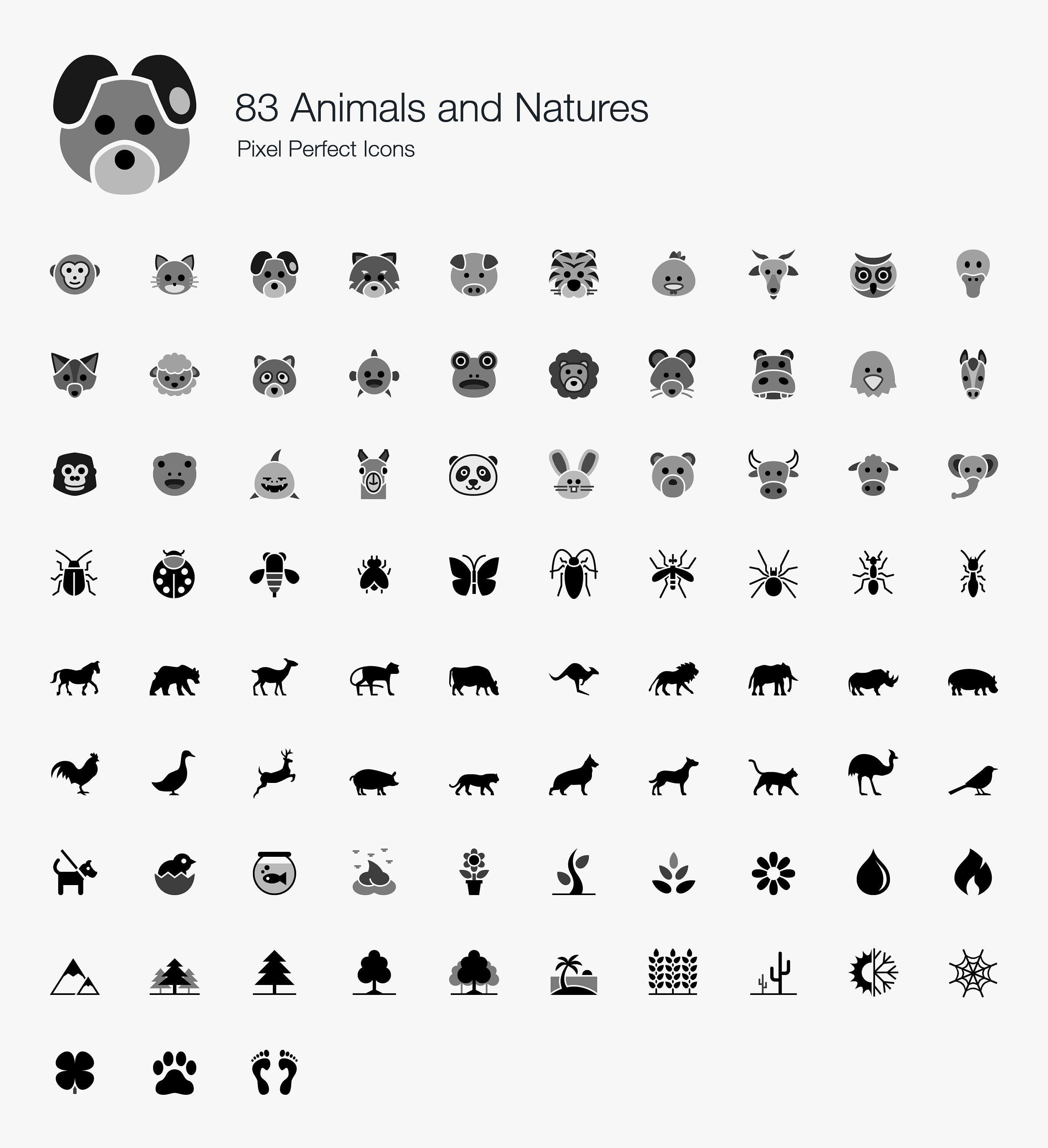  *Psychotria lupulina* | -0.09(0.41) | -0.14(0.37) | -0.27(0.13) | -0.23(0.07) |

**1** – Sampling locations located across banks of the Rio Branco.

**2** – Sampling locations located in the right bank of the Rio Branco.

**3** – Sampling locations located in the left bank of the Rio Branco.

**4** – Sampling locations located in both banks of the Rio Branco.

**TABLE S9** Pearson's correlation coefficient between contemporary migration rates and geographic distances of pairs of sampling locations in different directions (from upstream to downstream and contrariwise) along the Rio Branco (Amazon Basin, Brazil). *P-values* are shown in parenthesis.

(A) *Upstream to downstream*

| **Plant species** | **Datasets** | | | | |
| --- | --- | --- | --- | --- | --- |
|  | **1** | **2** | **3** | **4** | **5** |
| 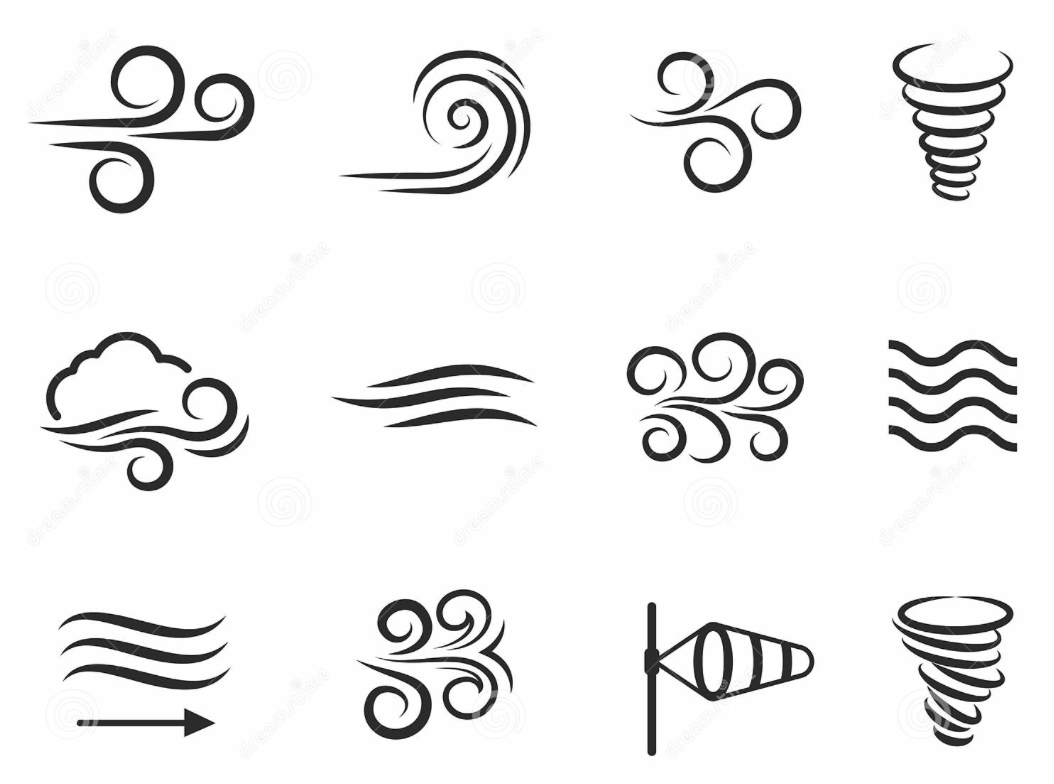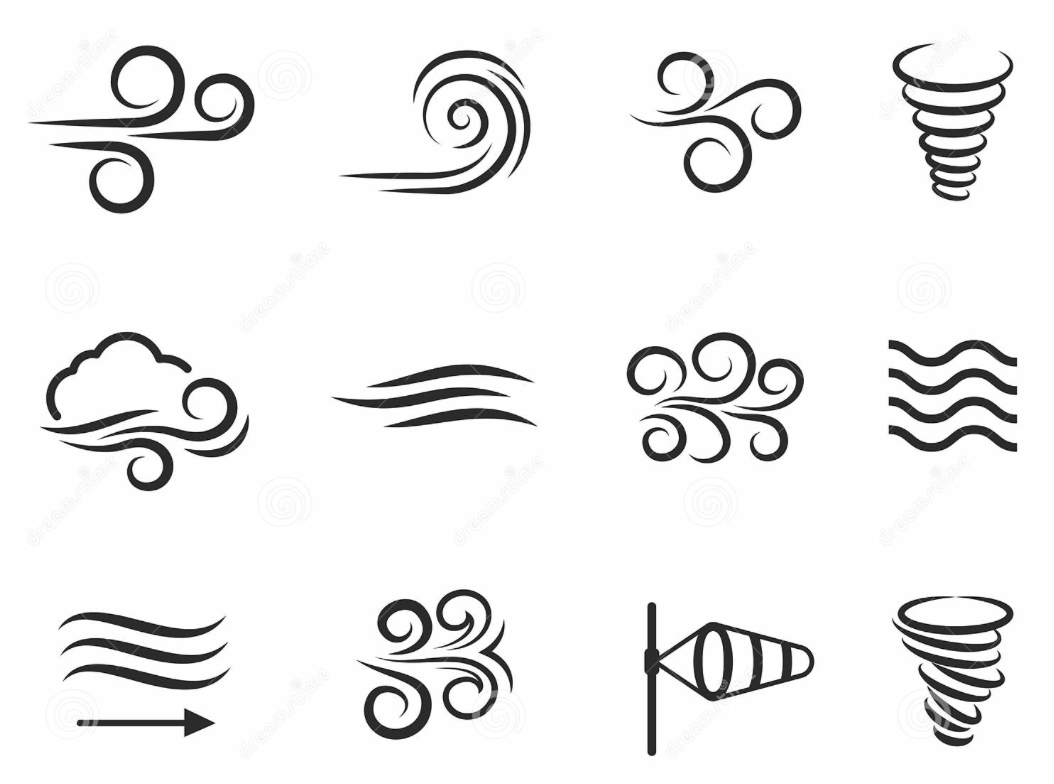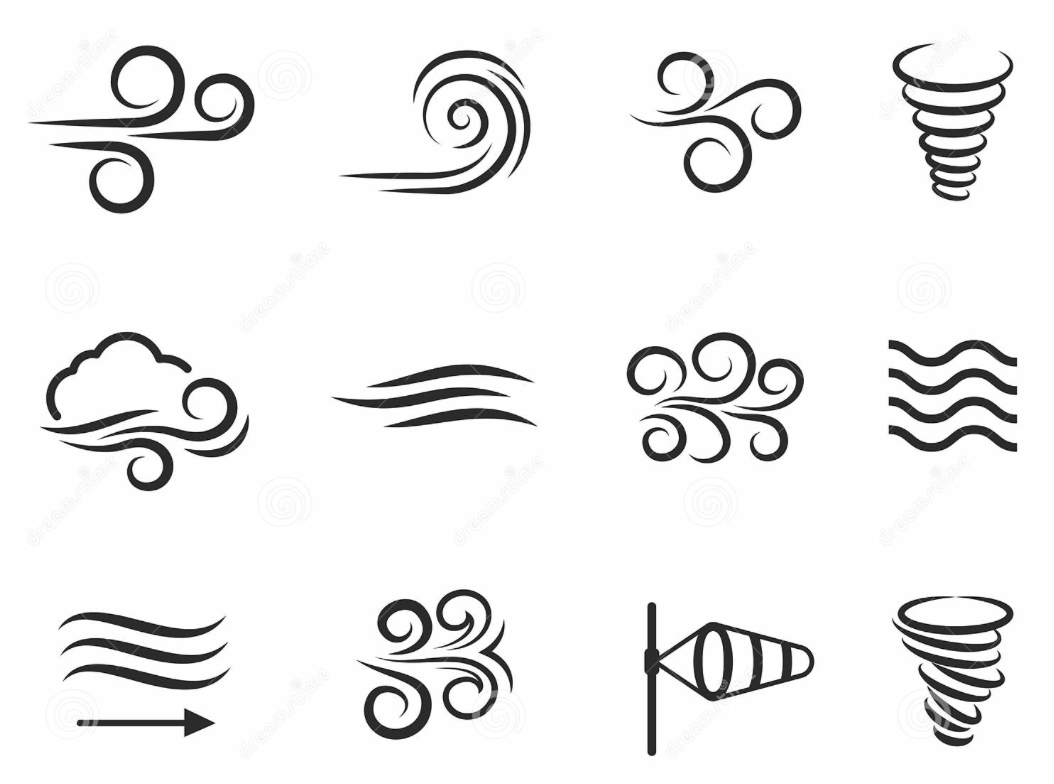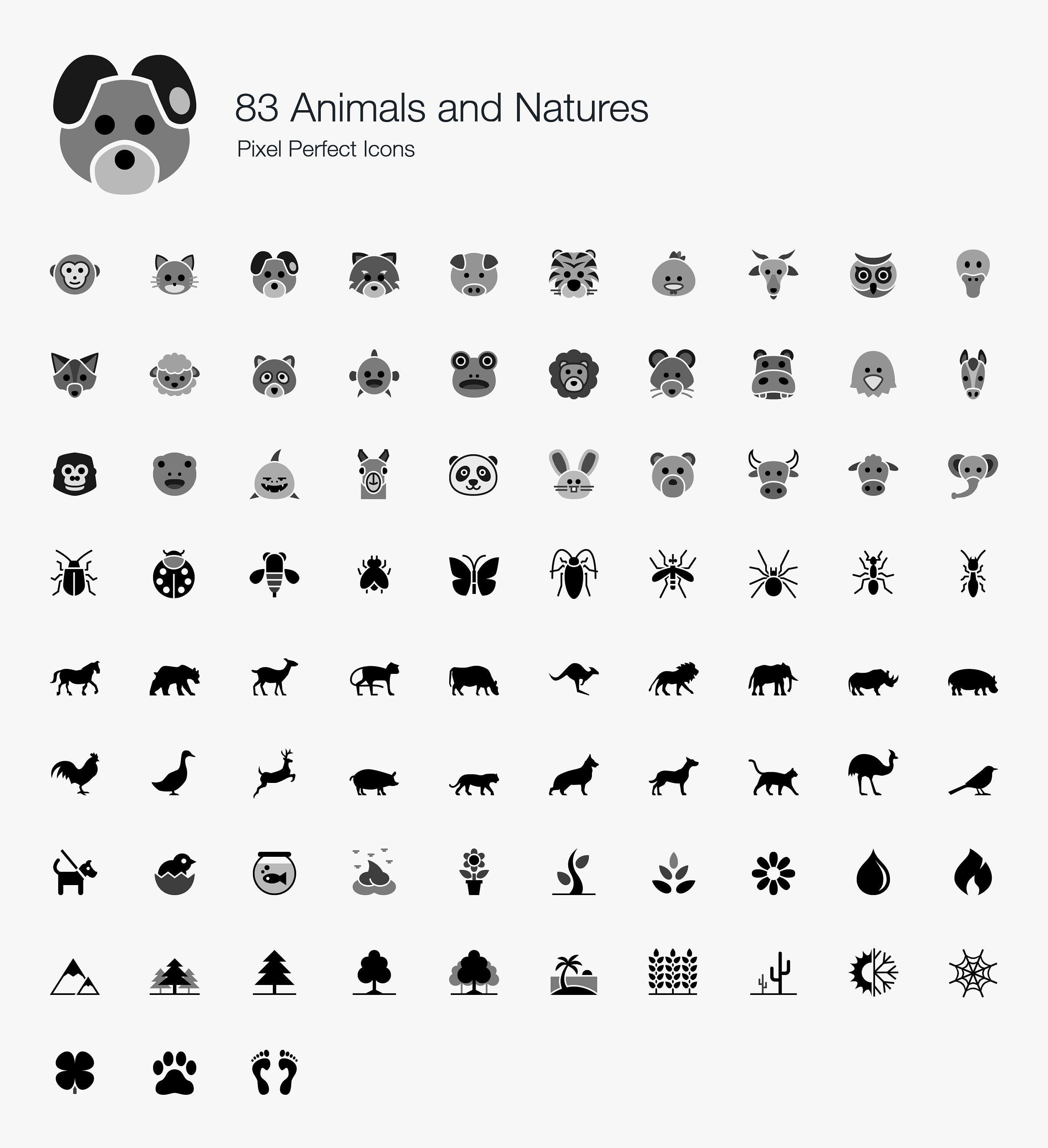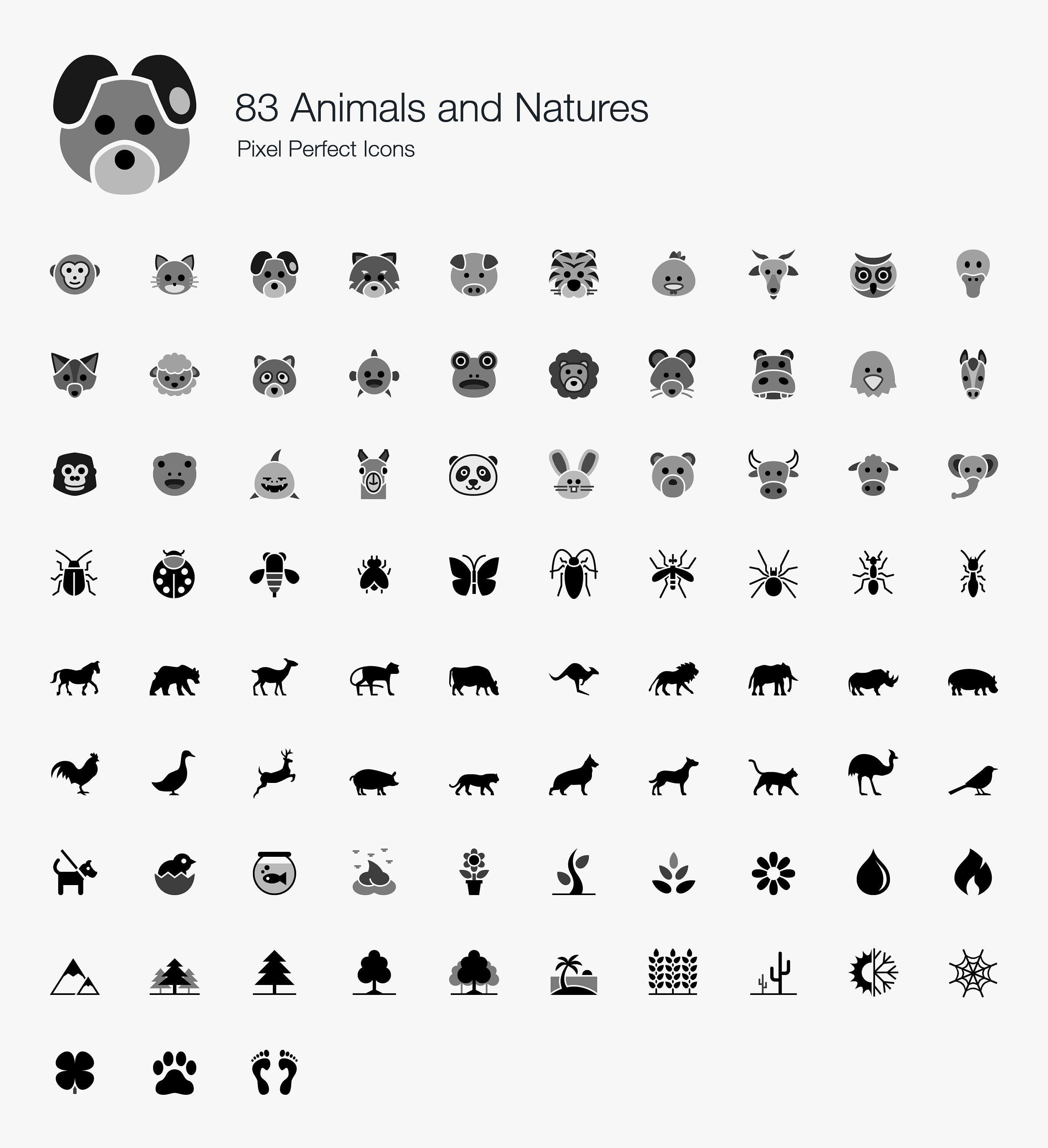*Adenocalymma schomburgkii* | -0.01(0.88) | -0.07(0.74) | 0.10(0.66) | 0.01(0.93) | -0.03(0.85) |
| *Bignonia aequinoctialis* | -0.17(0.14) | -0.27(0.32) | -0.22(0.32) | -0.17(0.30) | -0.18(0.31) |
| *Tanaecium pyramidatum* | -0.11(0.37) | 0.19(0.48) | -0.02(0.91) | 0.06(0.74) | -0.18(0.30) |
| 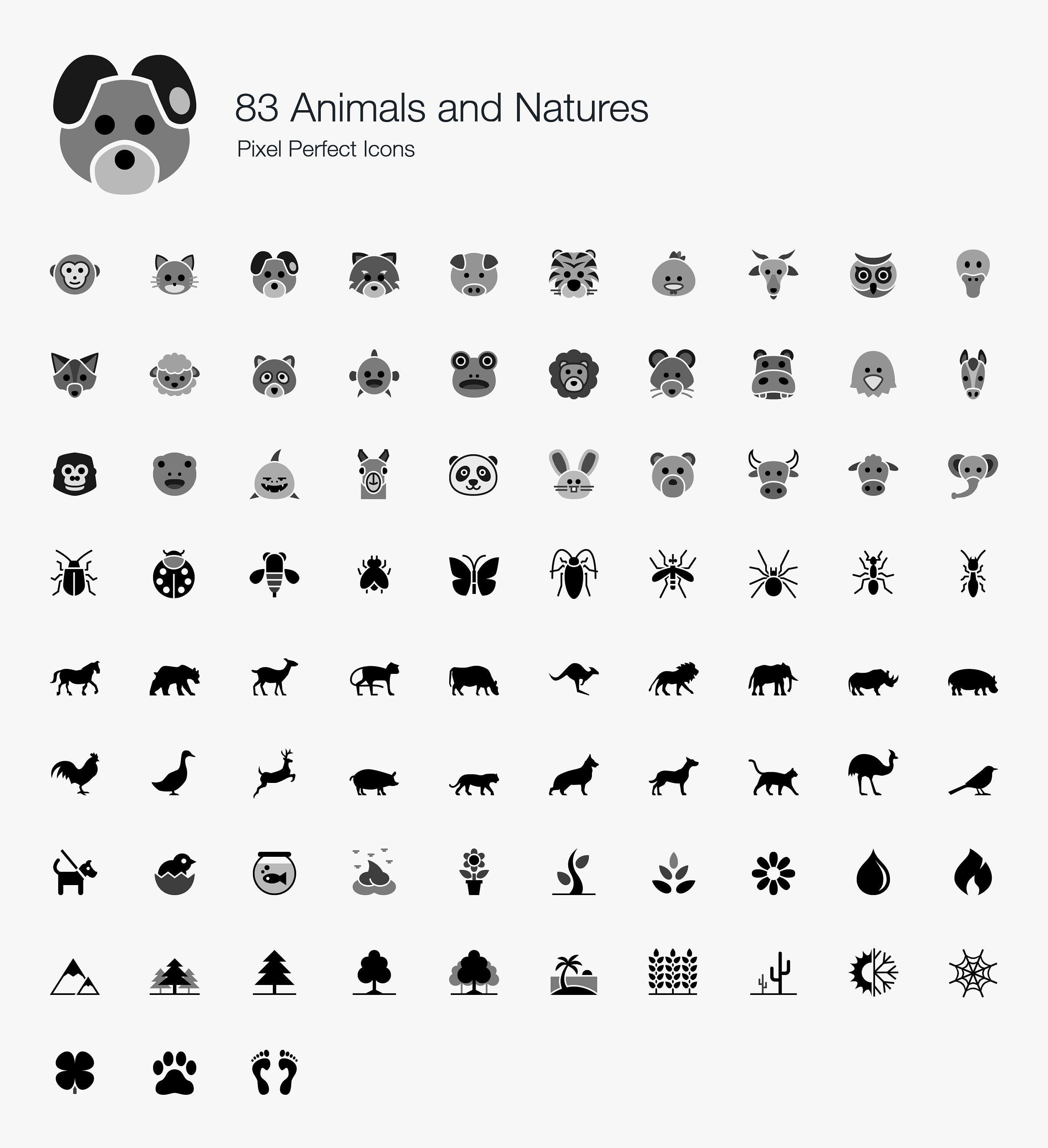  *Anemopaegma paraense* | -0.13(0.21) | -0.32(0.16) | -0.18(0.43) | -0.21(0.18) | -0.04(0.78) |
| 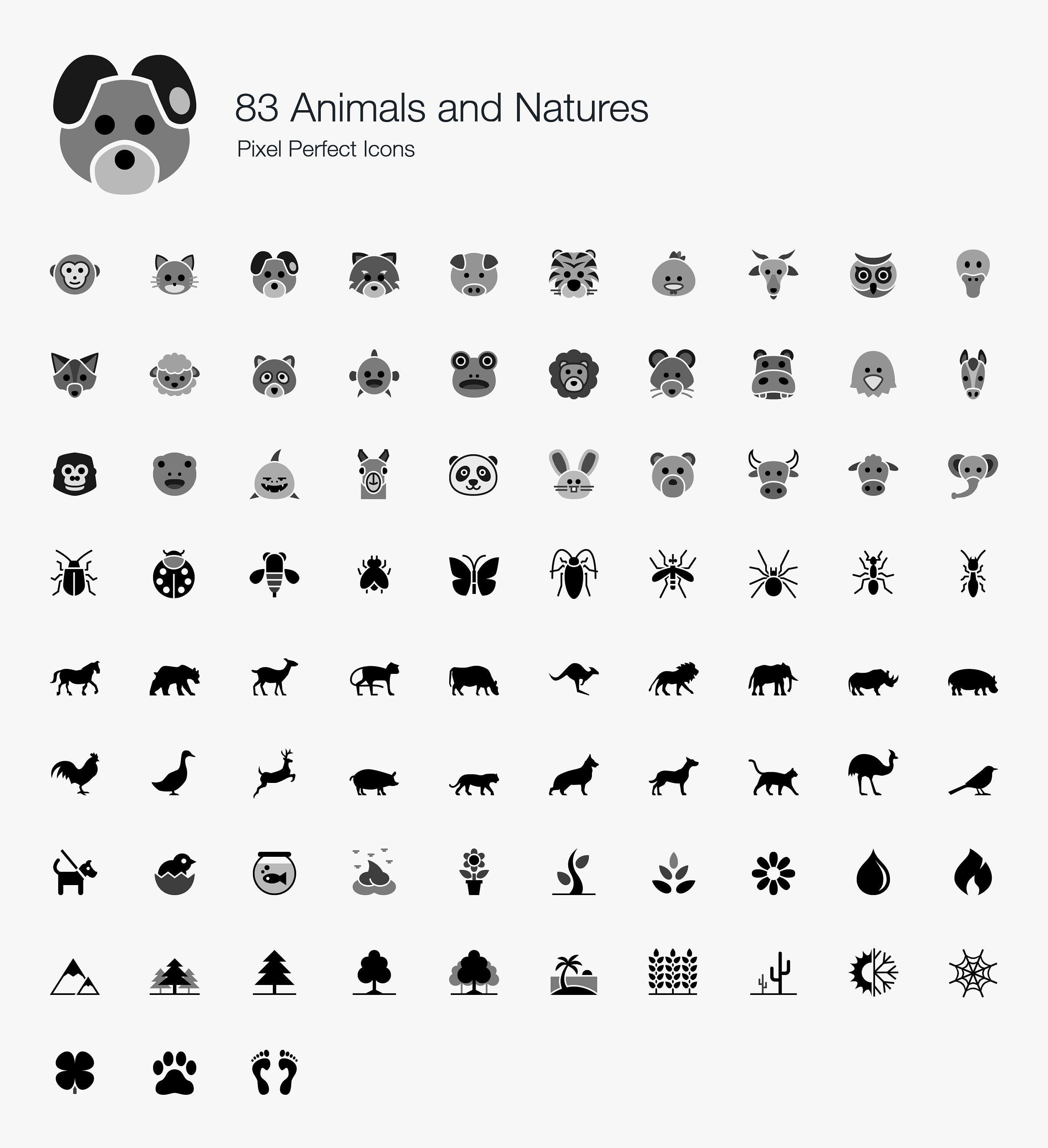  *Pachypetra kerere* | 0.07(0.53) | 0.57(0.00) | 0.40(0.05) | 0.48(0.00) | -0.26(0.09) |
| *Amphirrhox longifolia* | -0.18(0.12) | 0.14(0.62) | -0.21(0.35) | -0.17(0.32) | -0.19(0.25) |
| *Passiflora spinosa* | 0.05(0.63) | -0.39(0.13) | -0.23(0.32) | -0.29(0.07) | -0.15(0.39) |
| *Psychotria lupulina* | -0.16(0.17) | -0.07(0.76) | -0.29(0.29) | -0.21(0.20) | -0.12(0.49) |

(B) *Downstream to upstream*

| **Plant species** | **Datasets** | | | | |
| --- | --- | --- | --- | --- | --- |
|  | **1** | **2** | **3** | **4** | **5** |
| 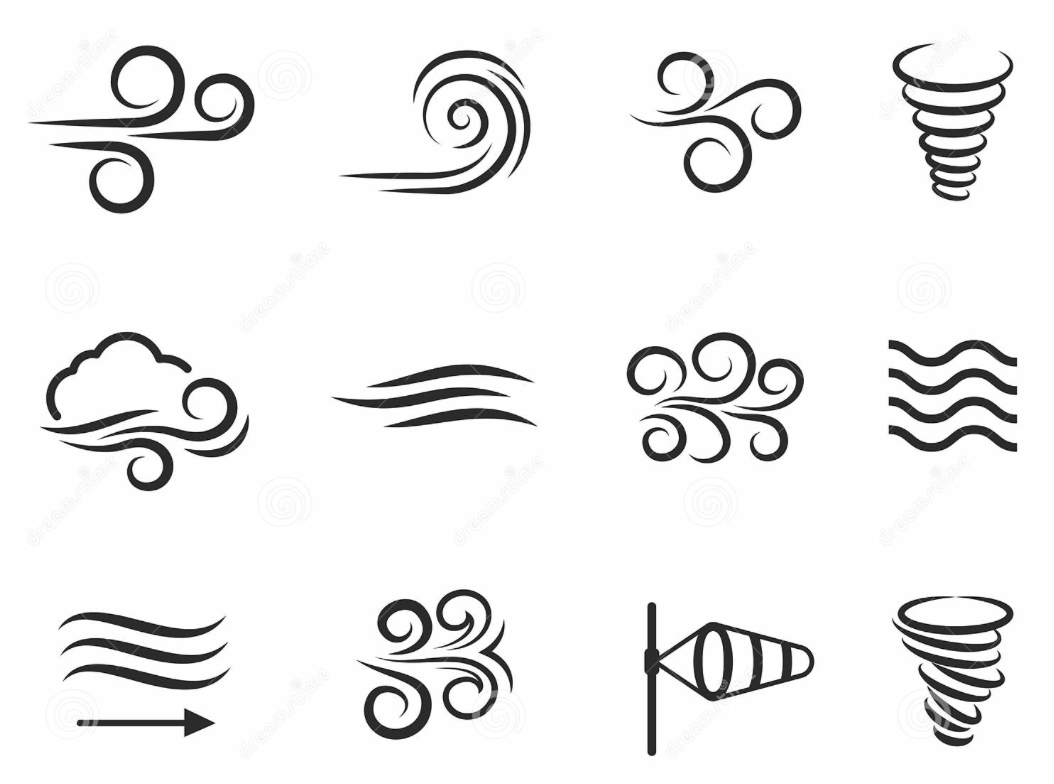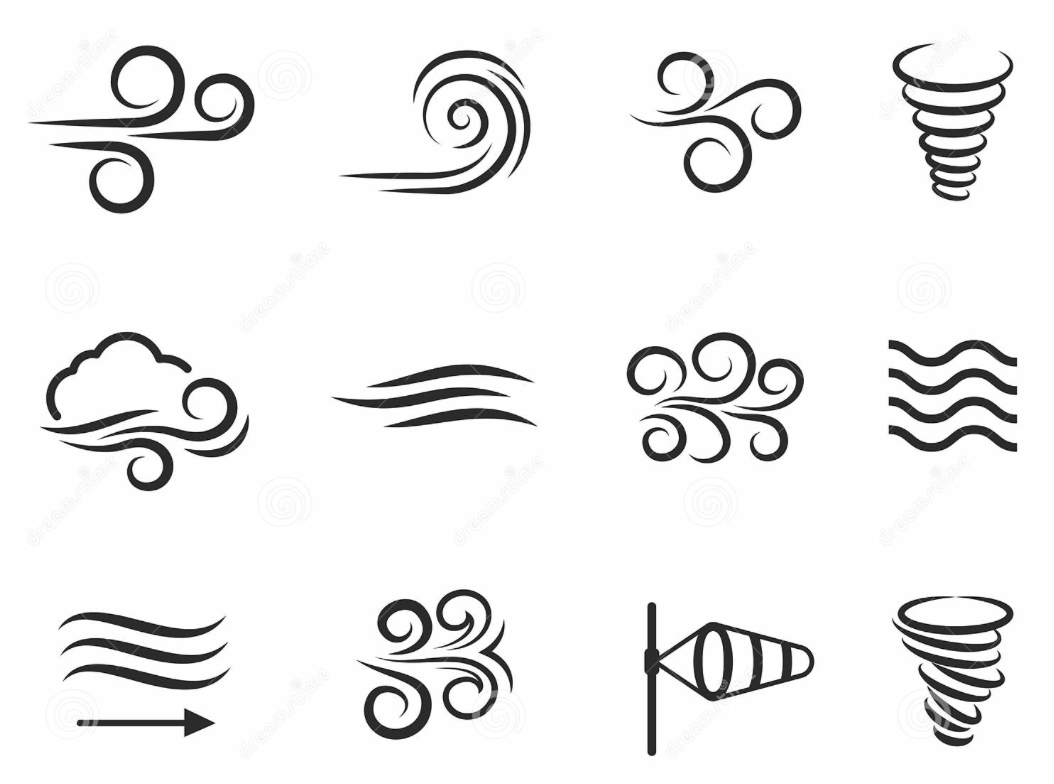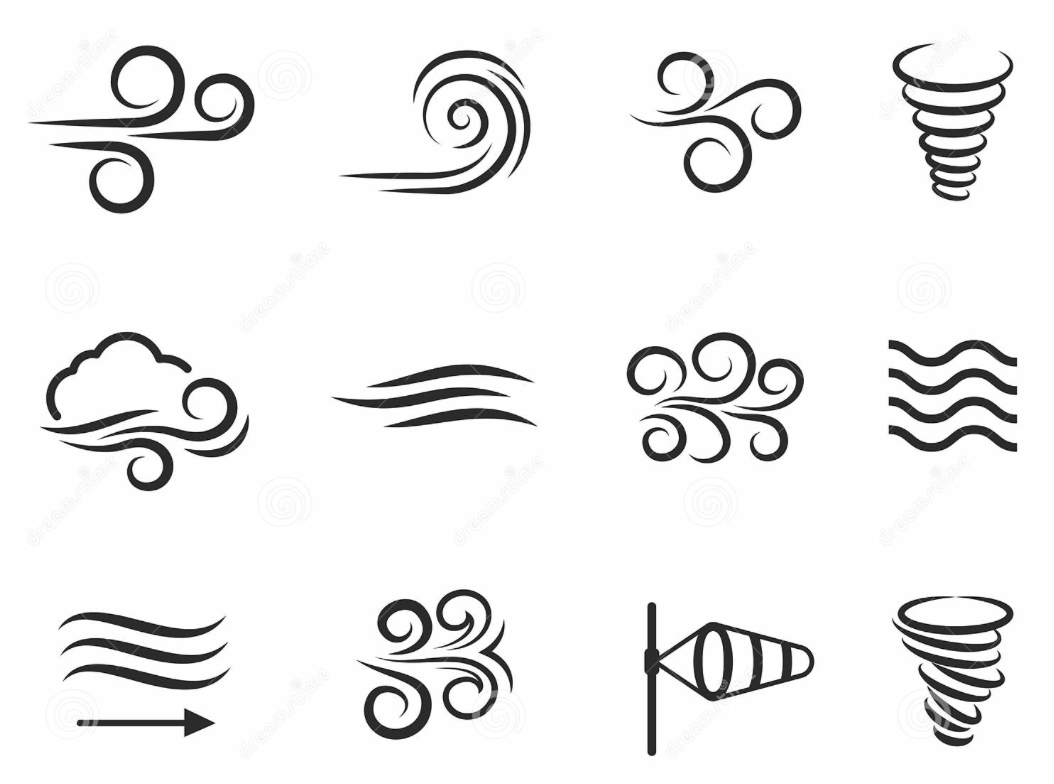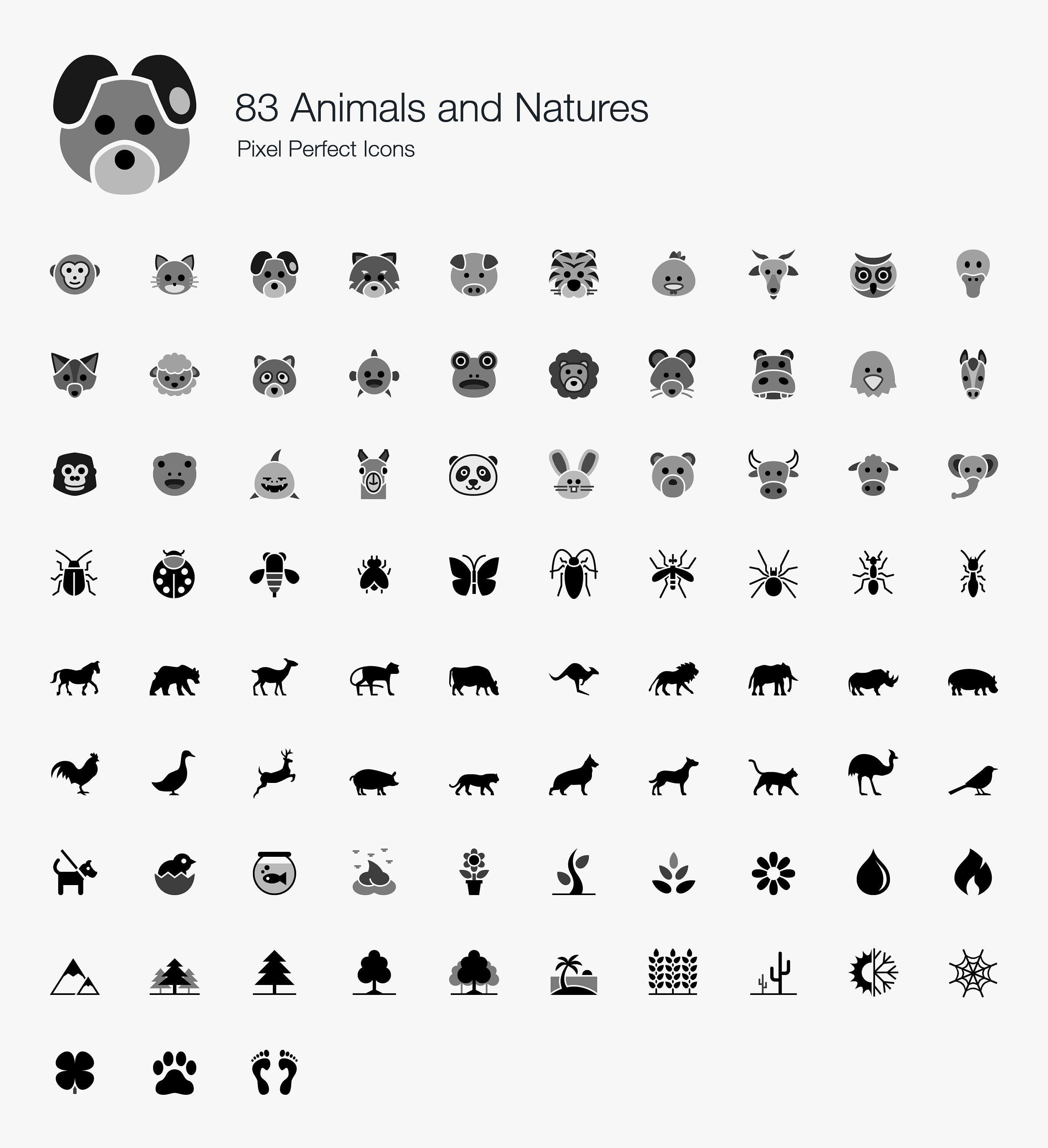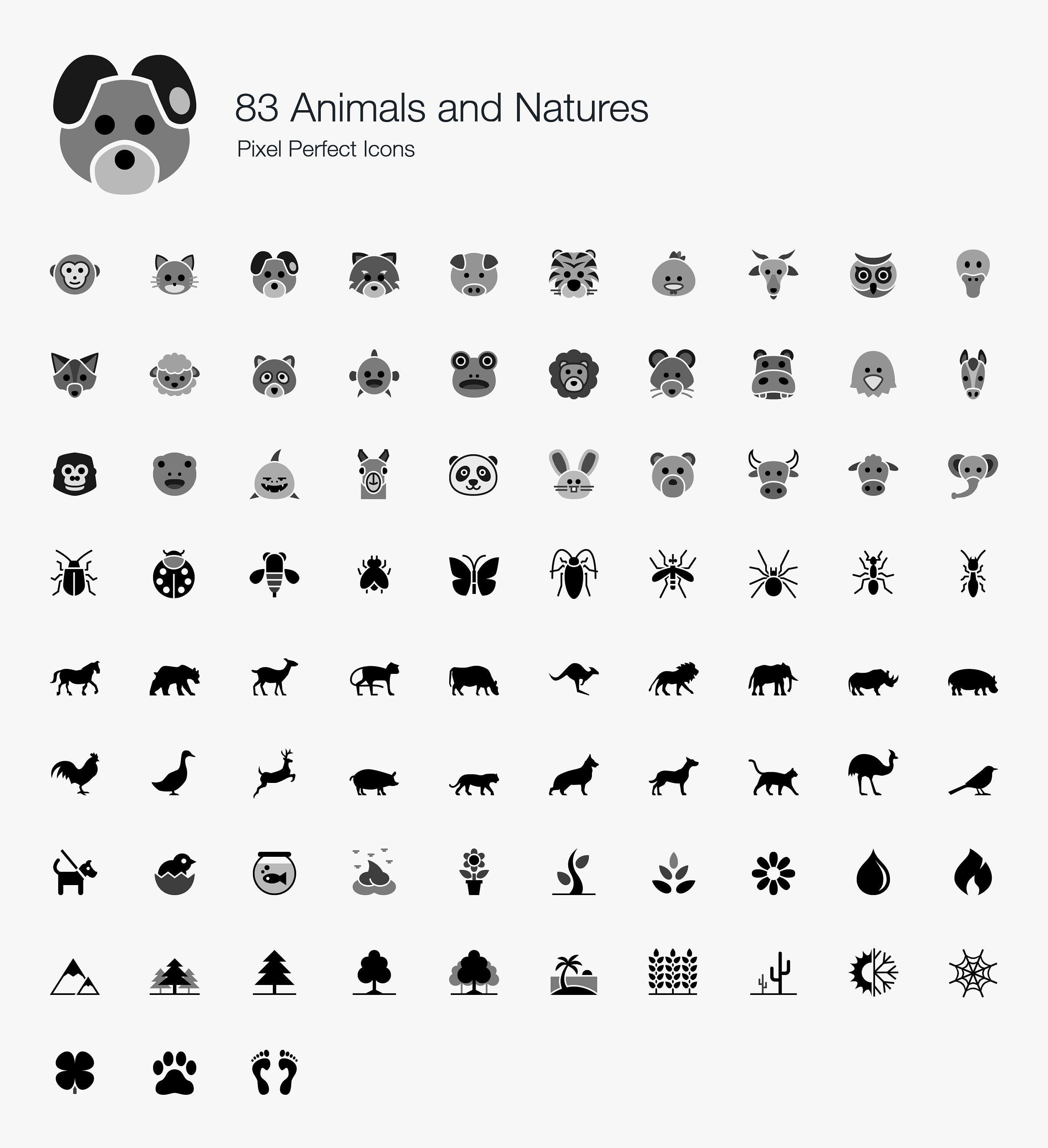*Adenocalymma schomburgkii* | -0.06(0.61) | -0.41(0.06) | -0.15(0.49) | -0.29(0.06) | 0.30(0.06) |
| *Bignonia aequinoctialis* | -0.10(0.39) | -0.33(0.23) | -0.15(0.51) | -0.12(0.47) | -0.07(0.67) |
| *Tanaecium pyramidatum* | -0.17(0.15) | -0.27(0.33) | -0.22(0.33) | -0.23(0.17) | -0.07(0.66) |
| 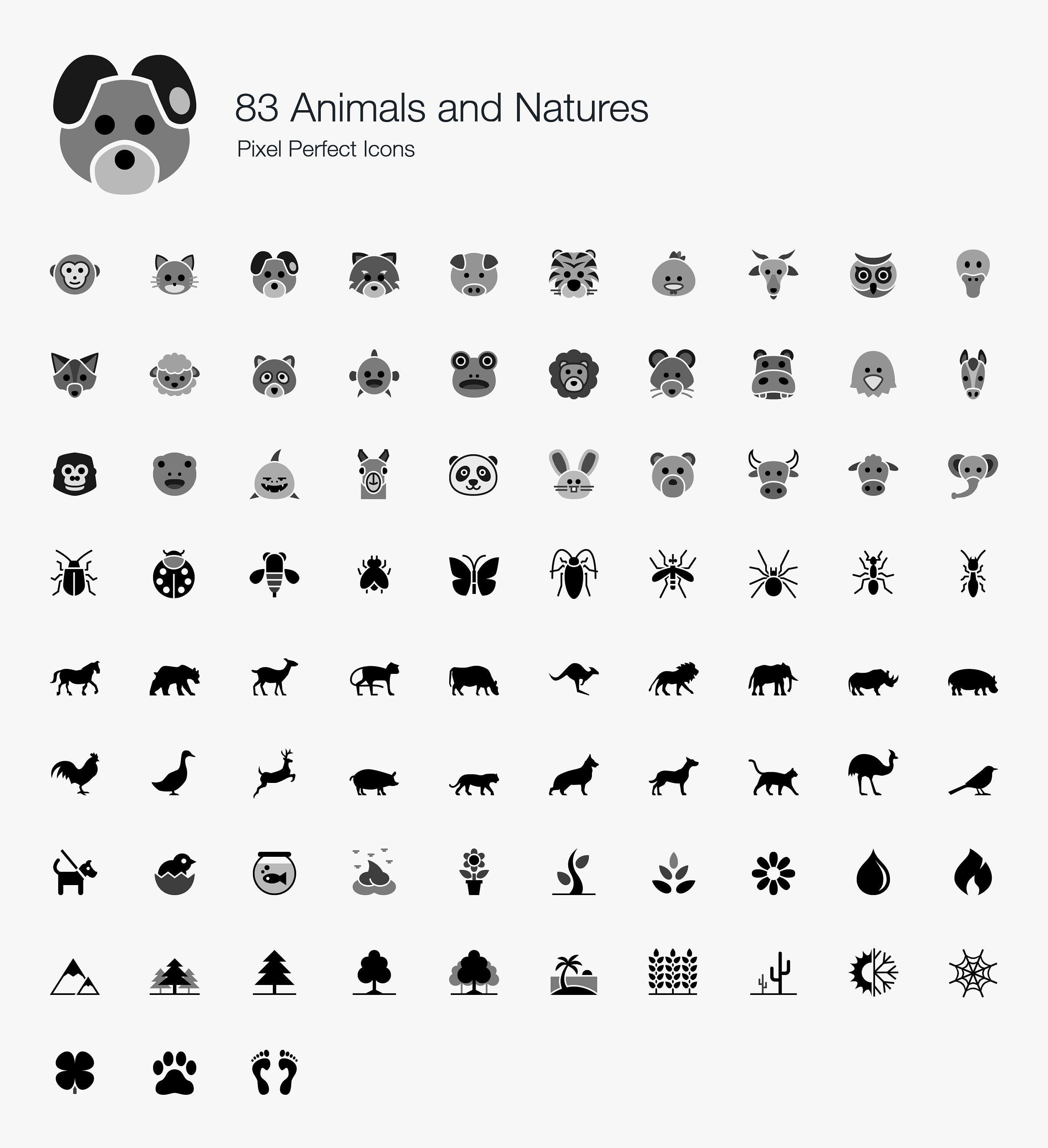  *Anemopaegma paraense* | -0.00(0.97) | 0.20(0.37) | -0.04(0.86) | 0.08(0.60) | -0.09(0.54) |
| 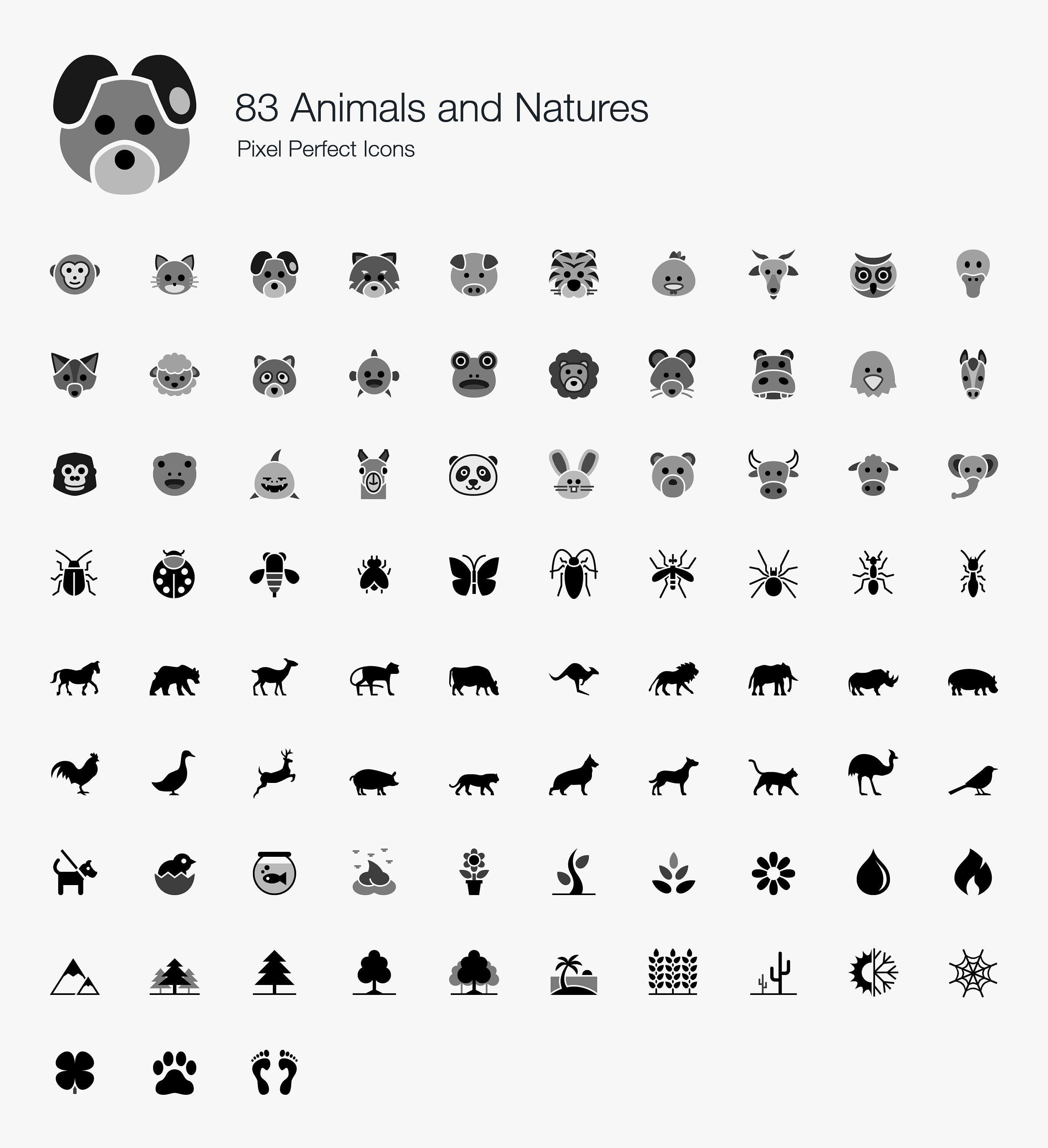  *Pachypetra kerere* | -0.23(0.07) | 0.29(0.19) | -0.23(0.31) | 0.08(0.96) | 0.11(0.49) |
| *Amphirrhox longifolia* | -0.08(0.47) | -0.21(0.45) | -0.15(0.51) | -0.13(0.43) | -0.03(0.85) |
| *Passiflora spinosa* | -0.04(0.72) | -0.04(0.87) | -0.08(0.72) | -0.06(0.71) | -0.02(0.89) |
| *Psychotria lupulina* | -0.17(0.15) | -0.22(0.32) | -0.31(0.26) | -0.29(0.08) | -0.07(0.65) |

**1** – All sampling locations; **2** – Sampling locations located in the right bank of the Rio Branco; **3** – Sampling locations located in the left bank of the Rio Branco; **4** – Sampling locations located in both banks of the Rio Branco, **5** – Sampling locations located across banks of the Rio Branco.

**FIGURE S1** Outlier loci test, implemented in the program Bayescan 2.1, on individual SNP markers for (A) *Adenocalymma schomburgkii*, (B) *Anemopaegma paraense*, (C) *Bignonia aequinoctialis*, (D) *Pachyptera kerere,* and (E) *Tanaecium pyramidatum*. Points displayed after the vertical line represent SNP markers potentially under diversifying selection.

**FIGURE S2** Correlations between genetic diversity parameters (*uH*_E_: unbiased expected genetic diversity; *H*_O_: observed heterozygosity) and geographic distances along the Rio Branco (Amazon Basin, Brazil) for *Pachyptera kerere* sampling locations.

**FIGURE S3** Unrooted maximum parsimony trees for eight riverine plant species using RAxML.

(A) *Adenocalymma schomburgkii*

(B) *Amphirrhox longifolia*

**

(C) *Anemopaegma paraense*

**

(D) *Bignonia aequinoctialis*

**

(E) *Pachyptera kerere*

(F) *Passiflora spinosa*

**

(G) *Psychotria lupulina*

**

(H) *Tanaecium pyramidatum*

**

**FIGURE S4** For each plant species, patterns of geographical structure as revealed by multidimensional scaling (MDS) of the matrix of genetic distances are showed in A. The Shepard diagram (inside A) shows the quality of the MDS representation. Population clustering analysis as calculated by GENELAND shows in B the density of the estimate of *k* along the Markov chain when spatial data are used (after a burn‐in of 5,000 × 100 iterations).

(A) *Adenocalymma schomburgkii*

(B) *Amphirrhox longifolia**

*Modified from Nazareno et al. 2019a

(C) *Anemopaegma paraense*

(D) *Bignonia aequinoctialis*

(E) *Pachyptera kerere*

(F) *Passiflora spinosa**

*Modified from Nazareno et al. 2019a

(G) *Psychotria lupulina**

*Modified from Nazareno et al. 2019a

(H) *Tanaecium pyramidatum*
